# Identification of chemical features for improved outer membrane permeation in mycobacteria using machine learning

**DOI:** 10.1038/s41564-026-02412-5

**Published:** 2026-06-30

**Authors:** Irene Lepori, Zichen Liu, Nelson Evbarunegbe, Shasha Feng, Turner P. Brown, Kishor Mane, Rachita Dash, Shivangi, Mitchell Wong, Ananya Naick, Amir George, Taijie Guo, Anil M. Shelke, K. Barry Sharpless, Jiajia Dong, Joel S. Freundlich, Wonpil Im, Anna G Green, Marcos M. Pires, M. Sloan Siegrist

**Affiliations:** 1 Department of Microbiology, University of Massachusetts Amherst, Amherst, MA USA; 2 Department of Chemistry, University of Virginia, Charlottesville, VA USA; 3 Manning College of Information and Computer Sciences, University of Massachusetts Amherst, Amherst, MA USA; 4 Department of Biological Sciences, Lehigh University, Bethlehem, PA USA; 5 Department of Bioengineering, Lehigh University, Bethlehem, PA USA; 6 Department of Pharmacology, Physiology and Neuroscience, Rutgers University-New Jersey Medical School, Newark, NJ USA; 7 Institute of Translational Medicine, Zhangjiang Institute for Advanced Study, Shanghai Jiao Tong University, Shanghai, China; 8 Department of Chemistry, The Scripps Research Institute, La Jolla, CA USA; 9 Molecular and Cellular Biology Graduate Program, University of Massachusetts Amherst, Amherst, MA USA; 10 Present Address: Merck Research Laboratories, Boston, MA USA

**Keywords:** Tuberculosis, Bacterial techniques and applications, Machine learning, Membranes, High-throughput screening

## Abstract

The ability of compounds to permeate and accumulate in bacterial cells is a critical determinant of antibiotic efficacy. Better therapeutics are urgently needed for the human pathogen *Mycobacterium tuberculosis*, yet the cell envelope, including the mycobacterial outer membrane, represents a significant barrier for drug entry, and the chemical features governing permeation remain poorly understood. Here we used the bioorthogonal click chemistry-based PAC-MAN assay to profile mycomembrane permeation of 1,572 azide-tagged compounds in *M. tuberculosis* and the model organism *M. smegmatis*. Cheminformatics and machine learning identified chemical features associated with mycomembrane permeation, which in turn had predictive value in three molecule series. Chemical predictors of mycomembrane permeation include nitrogen-containing aromatic scaffolds, such as indole, which in some cases were associated with increased anti-*M. tuberculosis* activity. Our data suggest a rational framework for improving mycomembrane permeation and whole-cell activity of antibiotics targeting *M. tuberculosis*.

## Abstract (web summary)

Screening 1,572 compounds for mycomembrane permeation plus cheminformatic and machine learning analyses reveals features associated with improved permeation in the pathogen *Mycobacterium tuberculosis* and model organism *M. smegmatis*.

## Main

Tuberculosis (TB) caused by *Mycobacterium tuberculosis* (Mtb) consistently claims over one million lives each year^1^. Antibiotic treatment is lengthy, complex and variably effective. Better drugs and drug regimens to target TB are urgent public health priorities.

An outstanding challenge in developing or improving drugs for TB is ensuring that drug candidates accumulate within Mtb cells^2,3^. To achieve whole-cell activity, a molecule must generally overcome several barriers, including membranes, efflux and metabolism, to accumulate with the kinetics and at the concentrations required to effectively engage its target. The features that enable a molecule to overcome individual accumulation barriers are divergent and sometimes at odds^4^. Moreover, even within bacteria there are interspecies and genera differences in molecule accumulation^5–12^. Together, these findings indicate that molecule accumulation in Mtb is likely to be a complex phenotype.

**Fig. 1:**
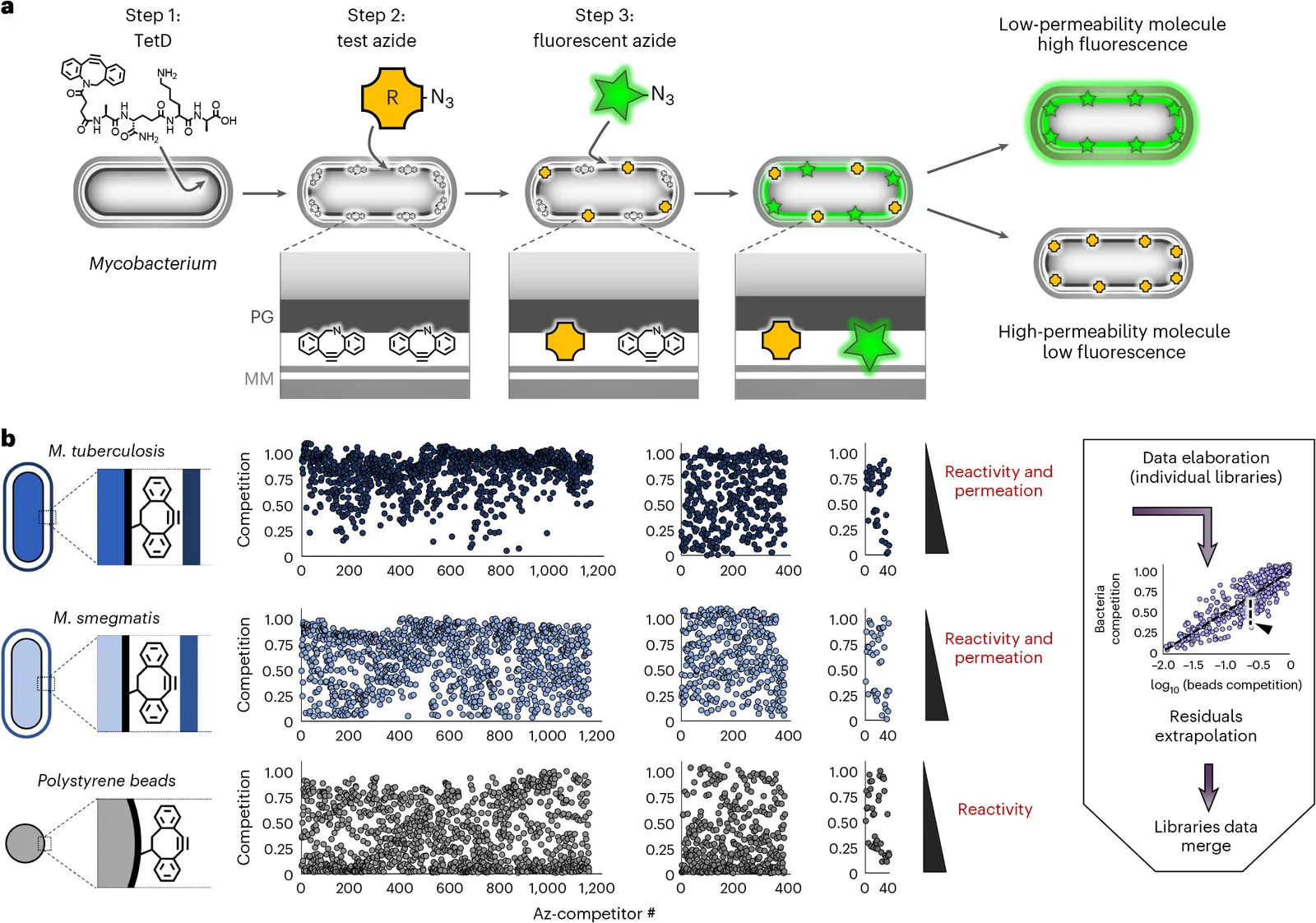
High-throughput screening of mycomembrane permeability. **a**, Schematic of PAC-MAN assay (adapted from ref. ^7^). In Step 1, Mtb or Msm is incubated with the DBCO-bearing TetD probe to tag cell wall peptidoglycan (PG). Mycobacteria are then exposed to an azide-modified test molecule (R, residue) (Step 2), followed by an azide fluorophore (Step 3). Test azides that do not permeate the mycomembrane (MM) do not access cell wall-embedded DBCO. DBCO that do not react in Step 2 are free to react with azide fluorophores in Step 3, resulting in high fluorescence. Conversely, test azides that permeate the mycomembrane can access and react with DBCO in Step 2, preventing DBCO from reacting with azide fluorophores in Step 3 and resulting in low fluorescence. **b**, PAC-MAN screening of three test azide libraries: Sharpless/Dong (1152), Enamine (380) or other commercial sources (40). Screening was performed on Mtb, Msm and DBCO-polystyrene beads to normalize for test azide reactivity. Standardized residuals were extracted from log-linear regression analyses for individual libraries as shown (see Extended Data Fig. 2a). Values for standardized residuals are inversely proportional to mycomembrane permeability of test azide. See Methods for screening details. Source data

Identifying chemical features that promote Mtb cell accumulation may enable more rapid development of new TB treatments. One way to address the complexity of accumulation is to focus on chemical features that enable a molecule to overcome a single barrier. For example, it has long been assumed that Mtb and related pathogens are intrinsically resistant to certain drugs in part because of the impermeability of the mycobacterial outer membrane^6,13,14^. This assumption is predicated on studies showing that mycomembrane disruptions sensitize mycobacteria to a subset of antibacterials^6,7,13–16^. Activity is an indirect proxy for uptake^17^; collateral metabolic dysfunction from cell envelope perturbation^18–21^ may also impact drug sensitivity. To clarify, we recently tested activity in parallel with Peptidoglycan Accessibility Click-Mediated AssessmeNt (PAC-MAN^7,22^; Fig. 1a)—an assay that measures the arrival of azide-modified molecules to the periplasm independent of their activity—to show that the mycomembrane is a selective permeability barrier and differentially restricts access of drugs. The chemical features that underlie mycomembrane selectivity, however, were unknown.

Here we addressed the complexity of Mtb accumulation by using PAC-MAN to screen the mycomembrane permeation of over 1,500 azide-tagged small molecules and deploying cheminformatics and machine learning to discover chemical features associated with this phenotype. De novo chemical synthesis and mycomembrane permeation testing of three molecule series confirmed that chemical correlates can also be predictive. Scaffolds such as aromatic nitrogen heterocycles, especially indole, and other chemical predictors of mycomembrane permeation are associated with anti-Mtb activity in different molecular contexts. Our work demonstrates that the mycomembrane is a major, albeit selective barrier to whole-cell activity in Mtb and suggests that improving the ability of a molecule to permeate the mycomembrane may aid the design or redesign of some antituberculars.

## Results

### High-throughput screening of mycomembrane permeability

**Extended Data Fig. 1:**
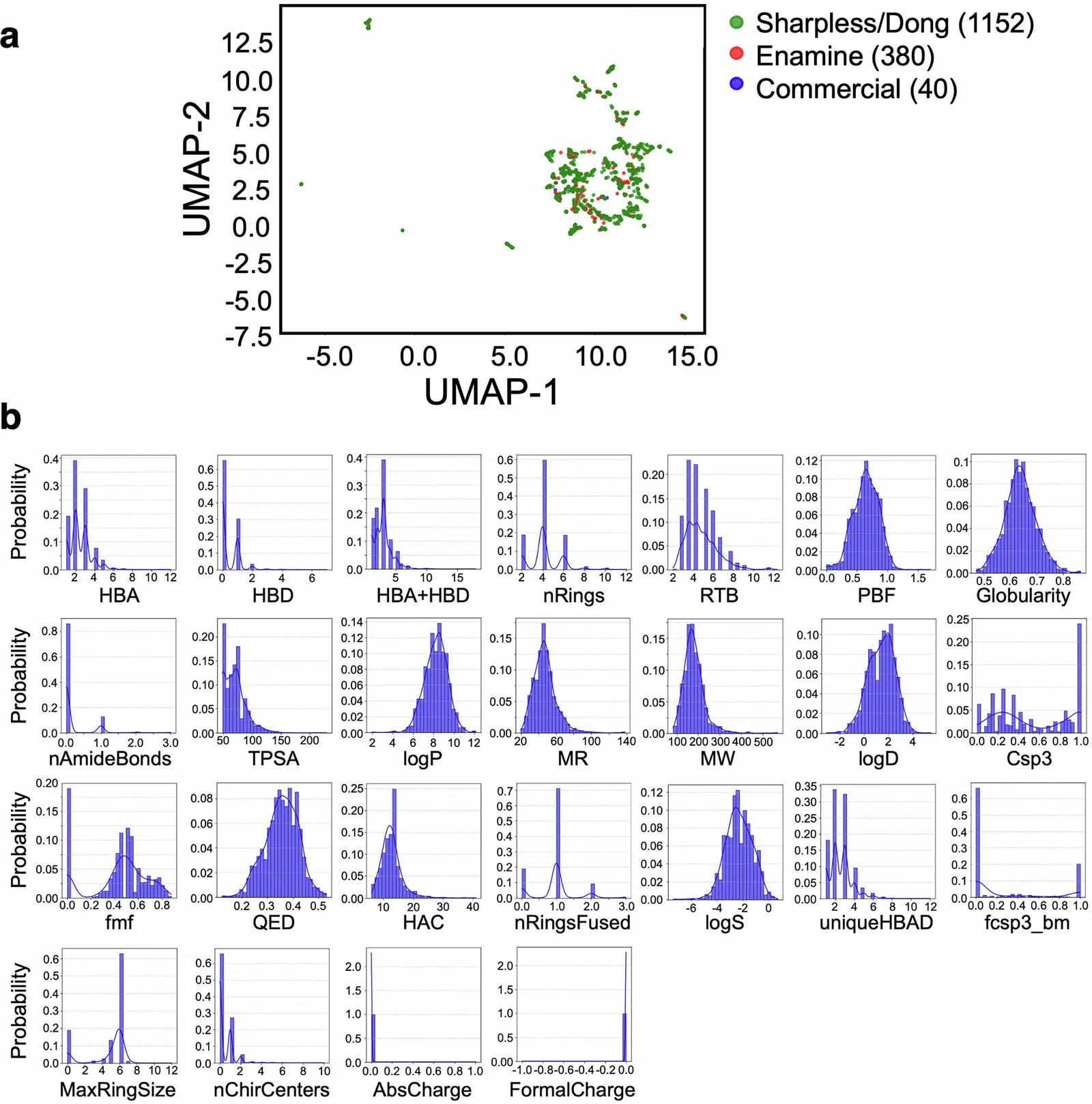
Chemical features of azide-tagged molecules screened by PAC-MAN. (**a**) 2D graph representing the chemical space of the three libraries (Sharpless/Dong, Enamine, and commercial) was generated by using the Uniform Manifold Approximation and Projection (UMAP) algorithm. (**b**) Distribution of major physicochemical properties of the libraries. HBA: hydrogen bond acceptor; HBD: hydrogen bond donor; nRing: number of rings; RTB: number of rotatable bonds; TPSA: topological polar surface area (Å^2^); Log P: *n*-Octanol/water partition coefficient (log); MR: molar refractivity (Å^3^); MW: molecular weight (Da); Csp3: fraction of sp^3^ carbons; fmf: fraction of heavy atoms in the molecular scaffold; QED: quantitative estimation of drug-likeness; HAC: heavy atom count; nFusedRings: number of fused rings; uniqueHBAD: number of unique hydrogen bond donors and acceptors; MaxRingSize: maximum ring size; fcsp3_bm: fraction of Csp^3^ hybridized carbons in the molecular scaffold; PBF: plane of best fit; AbsCharge: absolute charge; FormalCharge: formal charge; Log D: *n*-octanol/water distribution coefficient at pH 7.4 (log); Log S: water solubility (log). Physicochemical properties were calculated using RDKit except for LogD and LogS that were calculated with Collaborative Drug Discovery (CDD).

**Extended Data Fig. 2:**
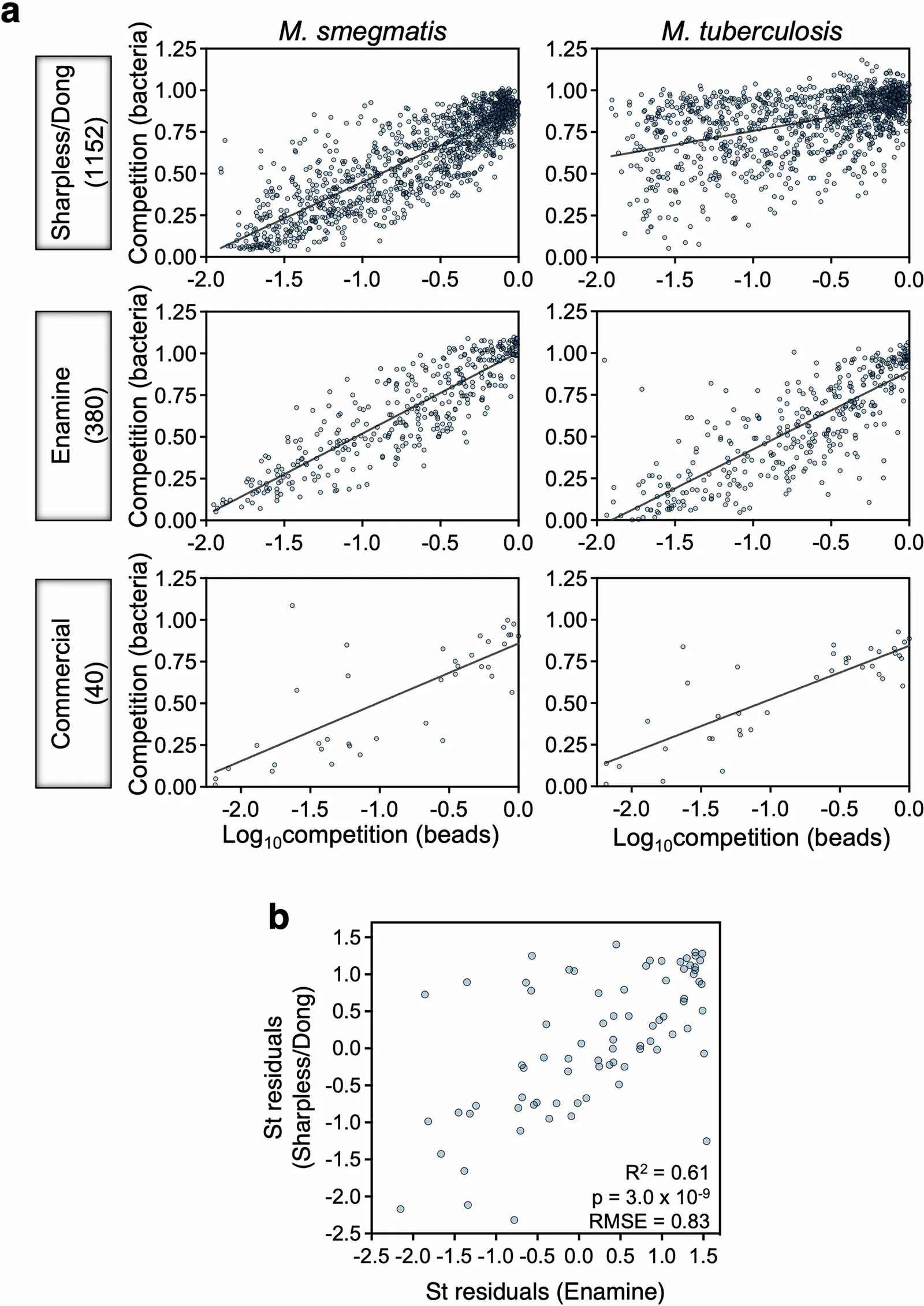
Log-linear correlation between test azide competition on DBCO-beads vs. DBCO-labeled bacteria and consistency of standardized residuals across libraries. (**a**) Screenings in *M. smegmatis* (*left*) and *M. tuberculosis* (*right*) were performed using test azides from Sharpless/Dong (1152), Enamine (380), and other commercial sources (40). Standardized residuals were generated for each library separately to account for potential differences in library quality. (**b**) Comparison of standardized residuals for test azides that are shared between the Sharpless/Dong (1152) and Enamine (380) libraries. Two-sided Spearman correlation test. RMSE: root mean square error. Source data

We aimed to understand the molecular basis of mycomembrane selectivity. To this end, we employed PAC-MAN (Fig. 1a), an assay that first proceeds via metabolic labelling of peptidoglycan, the cell wall polymer immediately beneath the arabinogalactan-mycomembrane layer of the cell envelope, with a dibenzocyclooctyne (DBCO) probe. A portion of the peptidoglycan-embedded DBCO is captured via bioorthogonal, strain-promoted alkyne-azide cycloaddition (SPAAC^23–25^) with azide-tagged test molecules. The remaining DBCO groups are revealed via SPAAC with a fluorescent azide. After controlling for azide reactivity, the level of mycobacterial cell fluorescence, measured by flow cytometry, inversely correlates with permeation of the azide-test molecule across the mycomembrane^7,22^. We used PAC-MAN to screen Mtb mc^2^6206 (H37Rv Δ*panCD* Δ*leuCD*)^26^ and the model organism *M. smegmatis* (Msm) mc^2^155 (ref. ^27^) with 1,572 azide-test molecules from three sources: 1,152 synthesized via fluorosulfuryl azide chemistry from the corresponding primary amine (Sharpless/Dong^28^), 380 purchased from Enamine, and 40 purchased from various commercial sources (Fig. 1b and Extended Data Fig. 1). The latter two sources contain molecules with primary amines. To control for the intrinsic reactivities of azide-test molecules towards DBCO^7,22^, we also screened DBCO-functionalized polystyrene beads that have bacteria-like dimensions but are devoid of a permeability barrier (Fig. 1b). We then performed log-linear regression analyses for the competition data on bacteria and beads to calculate the differences between observed and expected fluorescence; the resulting standardized residuals (Fig. 1b and Extended Data Fig. 2a) were consistent across libraries (Extended Data Fig. 2b).

### Chemical features associated with mycomembrane permeation

**Fig. 2:**
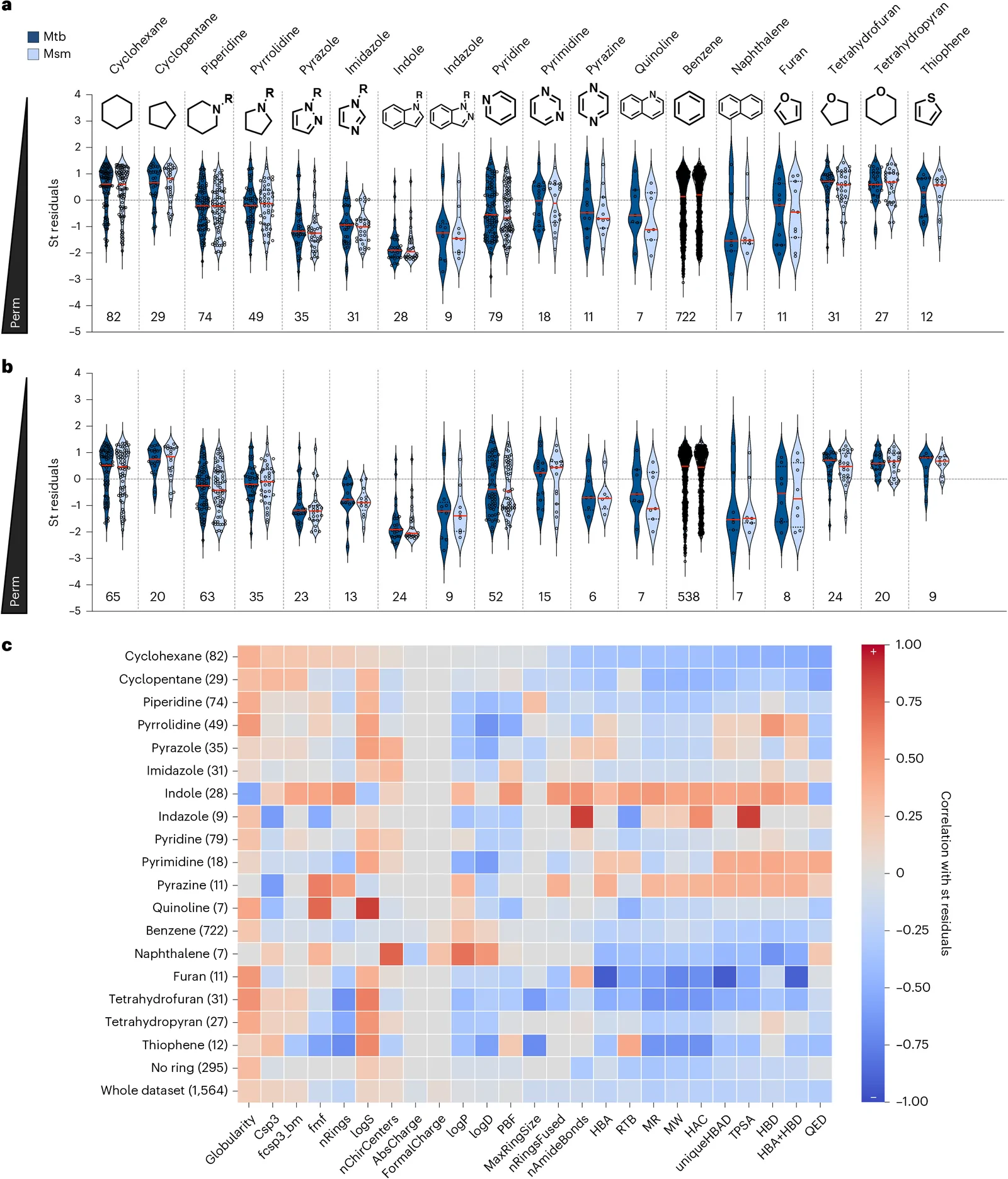
Chemical scaffolds and physicochemical properties that correlate with mycomembrane permeability. **a**, Mycomembrane permeability (standardized [st] residuals) for test azides grouped by the presence of a specific ring-containing scaffold, indicated on top (Perm, permeability; R, residue). **b**, ‘Greedy’ scaffold analysis excludes the fused ring structures. Numbers below the plots in **a** and **b** are the number of compounds in each group. Red line, median; dotted line, upper and lower quartiles. **c**, Correlation between mycomembrane permeability and selected physicochemical properties for all test azides (whole dataset, bottom row) and for subgroups bearing the scaffolds plotted in **a** and **b**. Numbers in brackets are the number of compounds in each group. Within each scaffold grouping, red denotes positive correlation between the indicated physicochemical properties and standardized residuals and thus negative correlation with mycomembrane permeability. Vice versa, blue denotes negative correlation between the indicated physicochemical properties and standardized residuals and thus positive correlation with mycomembrane permeability. For descriptor explanations, see Extended Data Fig. 1.

**Extended Data Fig. 3:**
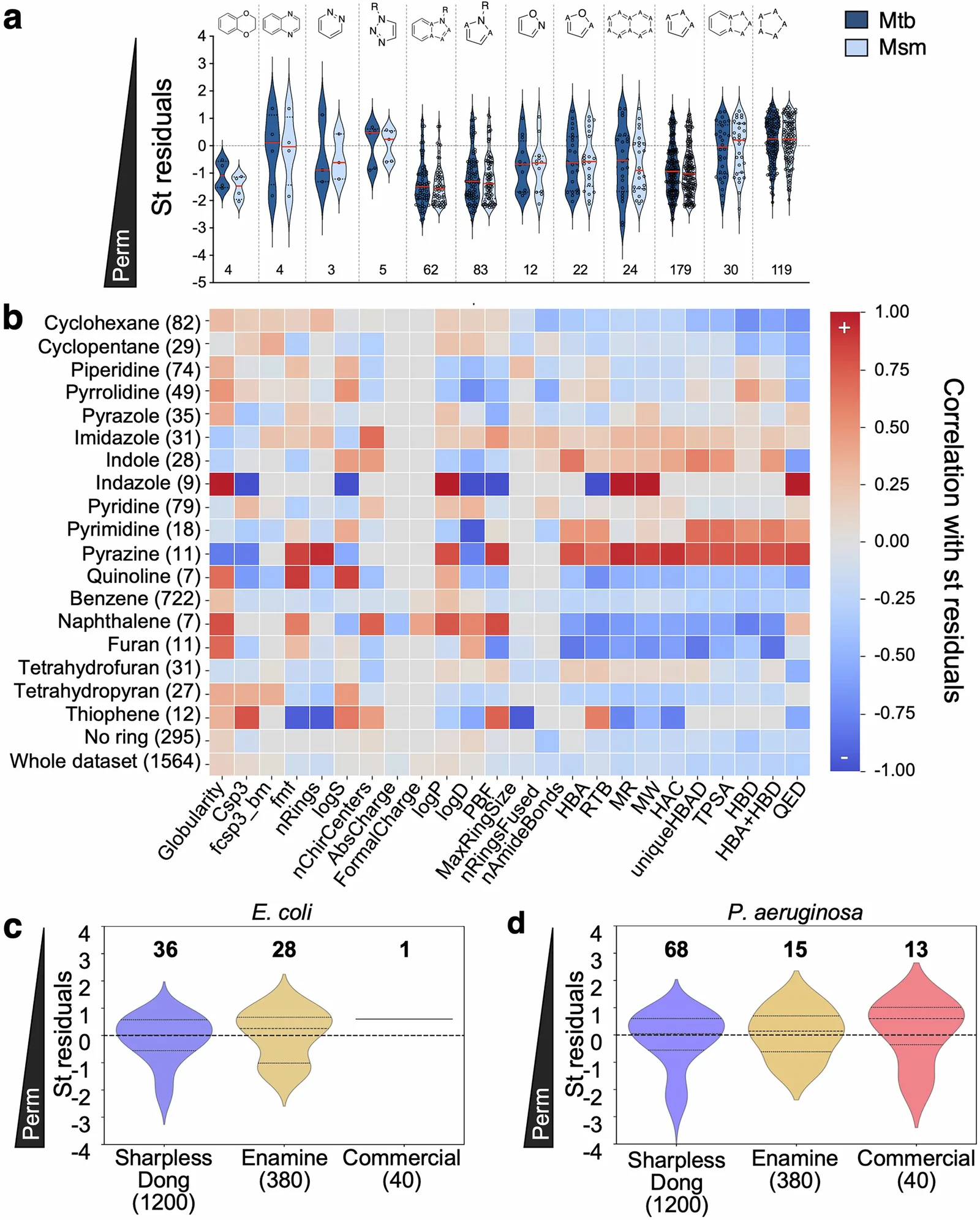
Extended cheminformatics analysis and mycobacteria permeability comparison with whole cell accumulation in Gram-negative bacteria. (**a**) Expanded set of chemical scaffolds analyzed as in Fig. 2a. The presence of aromatic heterocycles bearing more than one heteroatom positively correlates with mycomembrane permeability. Loss of aromaticity (last two scaffolds) negatively correlates with mycomembrane permeability. A: any atom. (**b**) Physicochemical properties analysis for Msm performed as in Fig. 2c. (**c-d**) Mycomembrane permeability scores for test azides that abide by (**c**) *E. coli* eNTRy rules^13^ (presence of an ionizable Nitrogen; low Three-dimensionality; low globularity (radius of gyration/molecular volume ≤ 0.25); and Rigid (≤ 5 rotatable bonds)) or (**d**) *P. aeruginosa* PASsagE rules (*Pseudomonas aeruginosa* Self-promoted Entry^12,27^ positive polar surface area (Q_VSA_PPOS ≥ 80); positive formal charge (FC ≥ .98); and high hydrogen bond donor surface area (HBDSA ≥ 23)). Dashed lines indicate median and upper and lower quartiles. Median values of the standardized residuals are ≥ 0, indicating low permeability in Mtb. Perm: permeability.

We first analysed the correlation between the presence of small, ring-containing scaffolds and mycomembrane permeability (Fig. 2a,b and Extended Data Fig. 3a). For both Msm and Mtb, we found that aromatic nitrogen-containing scaffolds such as indole, imidazole or pyrazole correlate positively with permeation (negative medians for standardized residuals), while scaffolds such as cyclopentane or cyclohexane correlate negatively (positive medians) (Fig. 2a,b and Extended Data Fig. 3a).

We next investigated the correlation between mycomembrane permeation and physicochemical properties (Fig. 2c for Mtb and Extended Data Fig. 3b for Msm). When analysed as a whole, our datasets revealed weak or no correlations. However, after grouping compounds by scaffold (Fig. 2a,b), we observed clear correlations. For example, topological polar surface area (TPSA) has a strong negative correlation with mycomembrane permeation when a molecule contains an indazole, and log partition coefficient (logP; Crippen logP calculation from RDKit^29^) has a strong negative correlation with mycomembrane permeation when a molecule contains an naphthalene. However, in the context of most other scaffolds, TPSA and logP have weak and/or positive correlations with mycomembrane permeation (Fig. 2c). These observations are striking as lipophilicity is generally viewed as a positive attribute for antitubercular drugs^30^. Notably, the physicochemical properties previously shown to correlate with Gram-negative accumulation^9,10,31^ were not associated with mycomembrane permeation (Extended Data Fig. 3c,d).

### Machine learning model to predict mycomembrane permeation

**Fig. 3:**
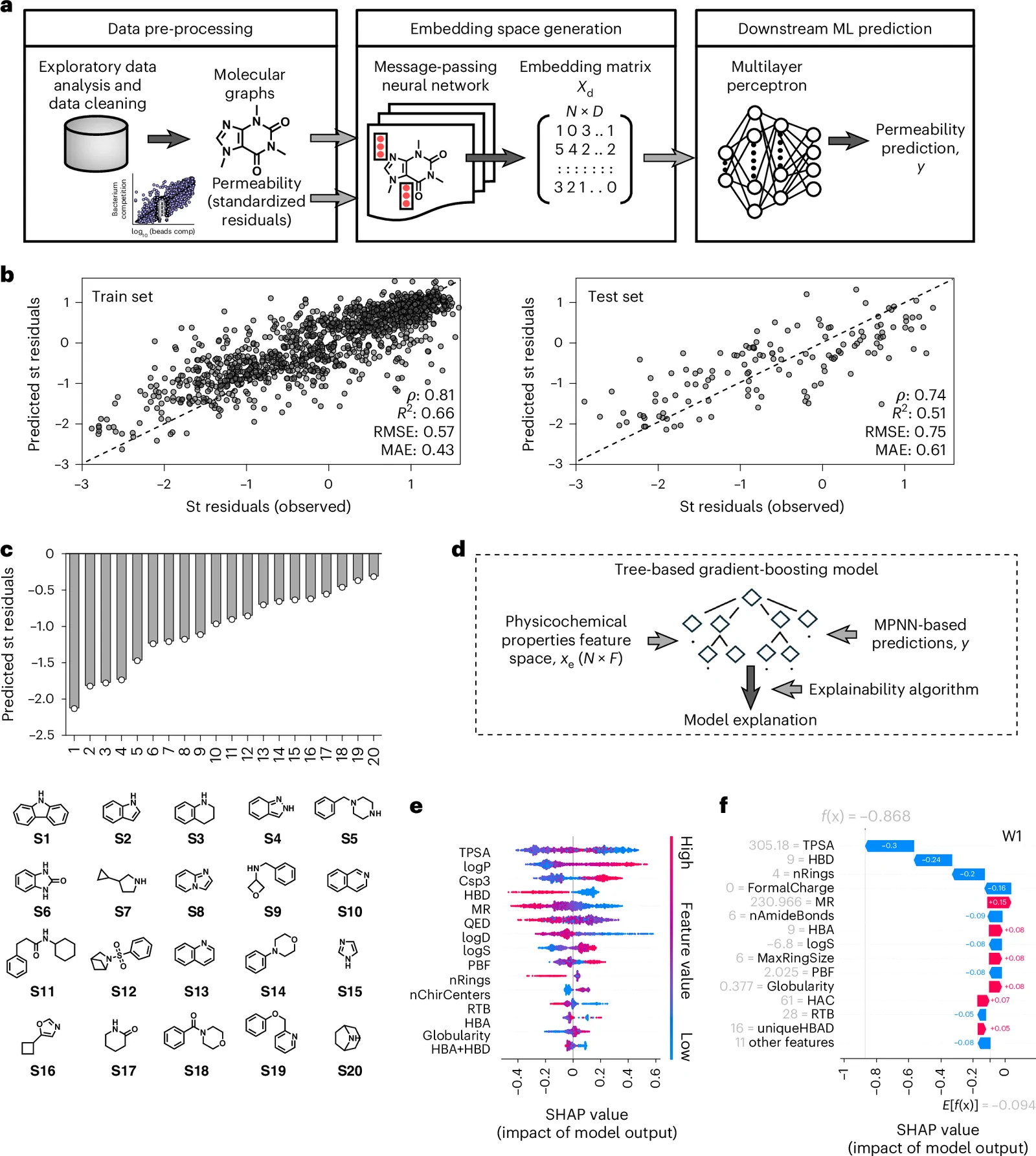
Machine learning model development and interpretation. **a**, Pipeline for machine learning (ML) model MycoPermeNet development. **b**, ML-predicted versus experimentally observed mycomembrane permeation data (standardized (St) residuals) for both the training (left) and held-out, scaffold-based test (right) sets. Train set: mean *R*^2^ 0.66 ± 0.051, RMSE 0.57 ± 0.039, MAE 0.43 ± 0.040. Test set: mean *R*^2^ 0.51 ± 0.055, RMSE 0.75 ± 0.041, MAE 0.61 ± 0.037. **c**, The 20 most-permeable chemical scaffolds predicted from the ML model. **d**, Pipeline for surrogate XGBoost ML model SHAP-based interpretability studies. XGBoost performance: *R*^2^ 0.86, RMSE 0.30 and MAE 0.23 for the interpretability fidelity on the full set, where the *R*^2^ explains 86% of the variance in the relationship between the features and MycoPermeNet. **e**, Ranking of top-15 (out of 25) physicochemical properties prioritized by the ML model to drive its prediction across all compounds. Molecular descriptors calculated using RDKit and Collaborative Drug Discovery (CDD) Vault. **f**, Interpretation of the ML predictions for a specific molecule (**W1** peptide, see Fig. 4e) from the perspective of physicochemical properties (descriptors). Rationalization and quantification of the positive or negative effect of the top-15 out of 25 descriptors (23 calculated with RDKit and 2 calculated with CDD Vault on molecule permeability across the mycomembrane. For results relative to the full list of descriptors, see Extended Data Fig. 4. For descriptor explanations, see Extended Data Fig. 1. RMSE, squared root of mean squared error; MAE, mean absolute error; *N*, number of compounds; *F*, number of descriptors; *D*, dimension of embeddings.

**Extended Data Fig. 4:**
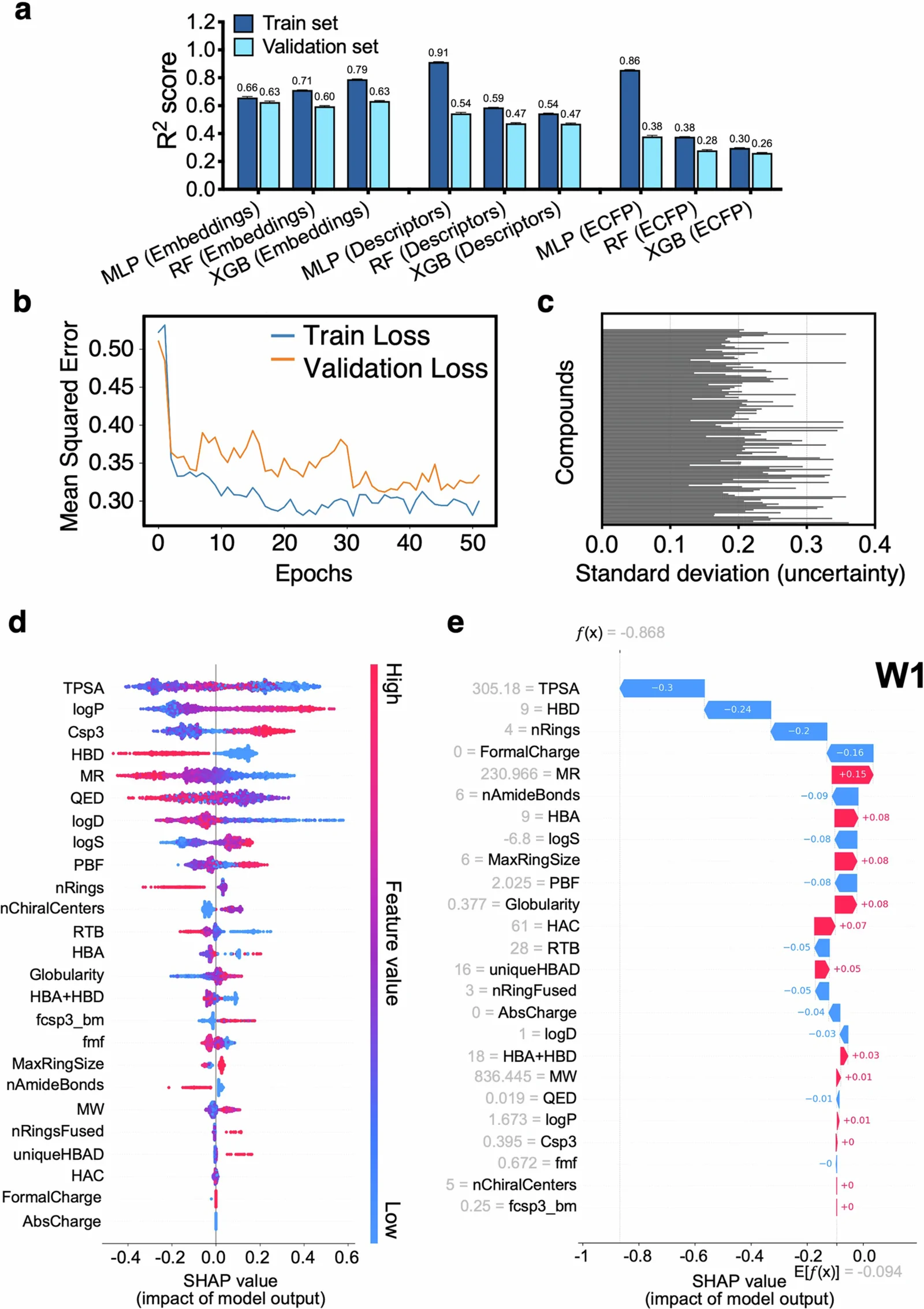
Development, validation, and interpretability studies of MycoPermeNet. (**a**) Performance across different ML models. Multilayer perceptron was chosen because it shows the highest median validation performance (R^2^) and closest performance in the train and validation sets. ECFP is length 1024 and has bit 3. MLP: Multilayer Perceptron; RF: Random Forest Regressor; XGB: XGBoost Regressor; ECFP: Extended-connectivity fingerprints. See the methods section for more details. Data are shown as means and s.e.m. for 49 states. (**b**) Loss curve for MLP model training. The model with median validation performance is shown. (**c**) Uncertainty of the median two-stage ML model prediction for the test set. (**d**) Global SHAP (SHapley Additive exPlanations) analysis for the entire azide library; ranks from the top-most impacting feature to the model’s prediction from top to bottom. TPSA has the largest impact on the model’s prediction. Each dot represents a compound within the library. Blue indicates low values of the feature, and red indicates high values of the feature. Points on the lefthand side of the plot show positive impact on Mtb’s permeability globally and vice versa for point on the right. For example, a red dot on the left side of the graph indicates that high values of that descriptor are positively contributing to the permeability (low shap values). (**e**) Local SHAP analysis for an individual compound’s prediction (shown here is the compound **W1**, Fig. 4e). The waterfall plot shows the model contributions of each of the 25 features by pushing the mean of the complex model’s predictions (*that is*, E[*f*(x)]), either negatively (blue) or positively (red), to the final permeability prediction of that compound (*that is*, *f*(x)). Blue indicates a positive contribution to high permeability and red indicates a negative contribution. The numbers attached to the features are the exact descriptor values for the **W1** compound.

We next built a machine learning (ML) model, called Mycobacterial Permeability neural Network (MycoPermeNet), to capture the complex relationships between chemical structure and mycomembrane permeability (Fig. 3a and Extended Data Fig. 4). Inspired by recent successes in deep learning for antibacterial discovery^32,33^, the model takes Simplified Molecular Input Line Entry System (SMILES) strings and Mtb screening data (that is, standardized residuals) as inputs, then uses a two-stage, deep-learning process to predict mycomembrane permeability. MycoPermeNet first generates vector representations of chemical compounds, called embeddings, then uses a type of neural network called multilayer perceptron to convert embeddings into a permeability prediction (that is, predicted standardized residuals).

MycoPermeNet was trained on 80% of the permeability data, while 10% of the data was used as validation set, and another 10% as test set (never observed by the model and used to assess performance). We selected the test set using the Bemis–Murcko scaffold split (see Methods section for details). Among several model architectures tested, the multilayer perceptron performed the best on the validation set (Extended Data Fig. 4a). It was trained until early stopping criteria were met, to minimize overfitting (Extended Data Fig. 4b), and achieved low uncertainty of predictions measured for the test set (Extended Data Fig. 4c). The final version of our model achieved good performance on train and test sets (Fig. 3b), indicating a strong relationship between observed and predicted mycomembrane permeability. Furthermore, the Spearman rank correlation coefficient (*ρ*) of 0.81 ± 0.019 on the train set and 0.74 ± 0.020 on the test set suggested that our model correctly ranks the relative permeability of compounds even better than it predicts their absolute permeability scores. After optimization and performance evaluation, we retrained a final version of MycoPermeNet on the entire dataset to maximize its predictive capability on unseen data.

To test whether MycoPermeNet was learning reasonable relationships between molecular structure and mycomembrane permeability, we asked the model to predict permeability scores for every chemical scaffold (for Bemis–Murcko definition) found in the dataset (*n* = 217). Among the 20 scaffolds predicted as most permeable (Fig. 3c), we found various indole-, imidazole- and pyrazole-like scaffolds. The concordance between the ML- and cheminformatics-based (Fig. 2) analyses serves as a validation of the ML approach and further reinforces the positive correlation between nitrogen-containing aromatic heterocycles and mycomembrane permeability.

To gain additional insights into the physicochemical properties associated with mycomembrane permeation, we performed interpretability studies of MycoPermeNet (Fig. 3d–f and Extended Data Fig. 4d,e). To make predictions, MycoPermeNet uses a large and complex set of parameters that is not human interpretable. Therefore, we built a surrogate model^34^ to determine which human-interpretable physicochemical properties have the most influence on MycoPermeNet predictions. Specifically, we used a surrogate XGBoost^35^ algorithm to predict the outputs of MycoPermeNet. XGBoost was trained using 25 interpretable, hand-selected input features previously shown to be important for molecule accumulation in Gram-negative bacteria^5,9–11^ (Fig. 3 and Methods).

After training our surrogate XGBoost model, we interpreted its features using Shapley Additive exPlanations (SHAP)^36^ (Fig. 3d). In this context, SHAP quantifies the contribution of each physicochemical property to the surrogate model predictions. SHAP identified TPSA and logP as the two most influential physicochemical properties in the generation of predictions (Fig. 3e and Extended Data Fig. 4d), suggesting that these physicochemical properties modulate mycomembrane permeation (although the direction of modulation varies). While SHAP returns properties that drive predictions across the dataset, it can also predict which features are responsible for the permeability prediction for individual compounds (Fig. 3f and Extended Data Fig. 4e). This analysis provides both a qualitative and quantitative interpretation of the impact of each physicochemical property on mycomembrane permeability.

### Scaffolds correlate with molecules’ mycomembrane permeability

**Fig. 4:**
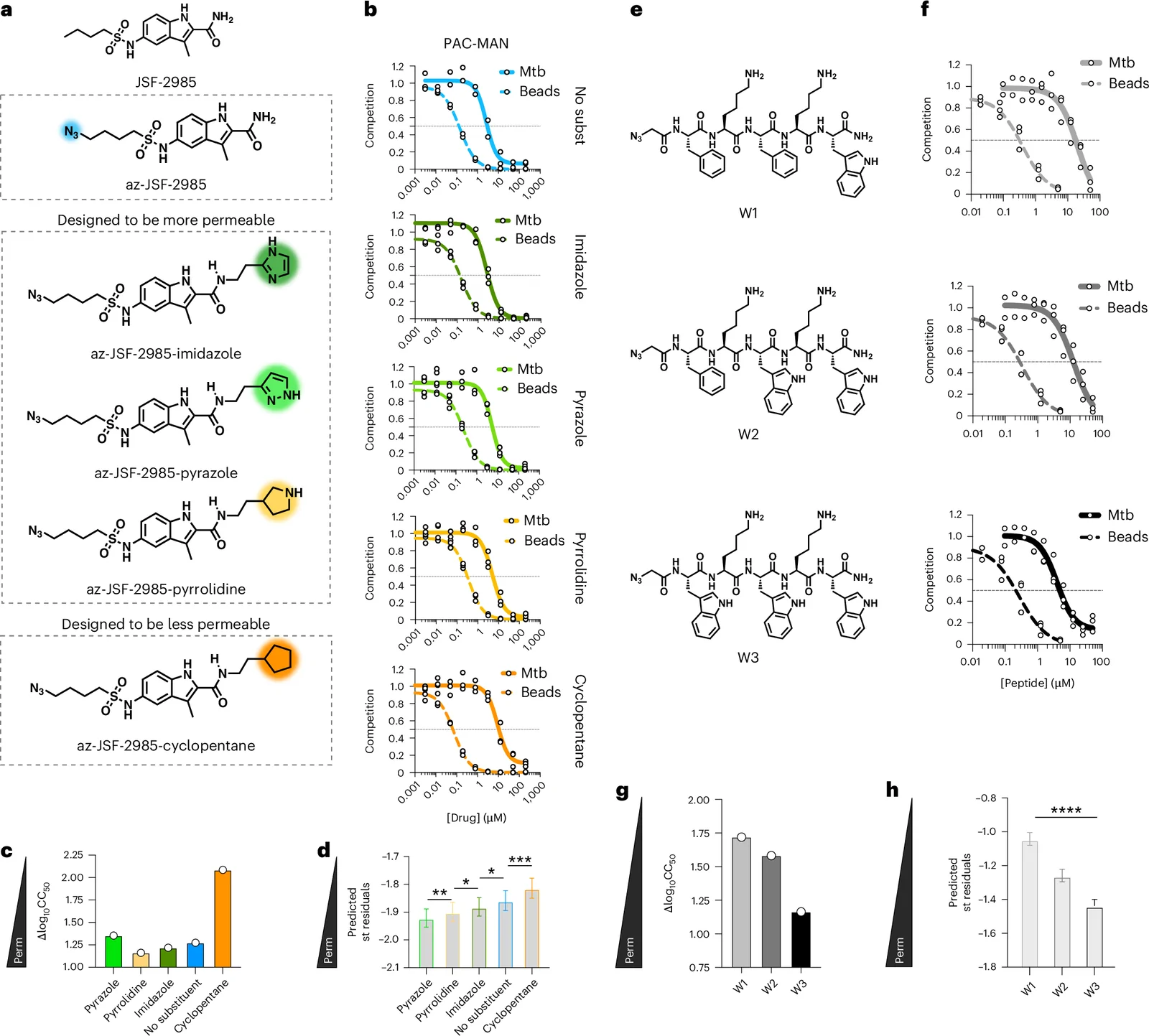
Scaffolds associated with mycomembrane permeation are predictive for JSF-2985 and the W peptide series. **a**–**d**, Analysis of an antitubercular candidate series. **JSF-2985** (**a**, top) was derivatized to bear an azide and chemical scaffolds that are associated with more (imidazole, pyrazole, pyrrolidine) or less (cyclopentane) mycomembrane permeability (Fig. 2a,b). **b**, PAC-MAN results for Mtb and beads. **c**, Observed (∆log_10_CC_50_) and **d**, ML-predicted mycomembrane permeability. *P* values from left to right: 0.00198; 0.0169; 0.0483; 0.000148. **e**–**h**, Analysis of a pentapeptide series in which benzene-containing phenylalanines are systematically substituted with indole-containing tryptophans (**W1–3**). The presence of indole or benzene scaffolds are respectively associated with higher or lower mycomembrane permeability (Fig. 2a,b). **e**, Peptide chemical structures. **f**, PAC-MAN results for Mtb and beads. **g**, Observed (∆log_10_CC_50_) and **h**, ML-predicted mycomembrane permeability. All *P* values are <10^−14^. For **b** and **f**, points represent biological replicates (*n* = 3) except for **az-JSF-2985** (no substituent) where *n* = 2. For **d** and **h**, data are mean ± s.e.m. generated by 50 random states of the model. Two-sided pairwise Wilcoxon signed-rank test. *P* values reported as adjusted *P* values across multiple comparisons. *P < 0.05, **P < 0.01, ***P < 0.001, ****P < 0.0001. Source data

**Extended Data Fig. 5:**
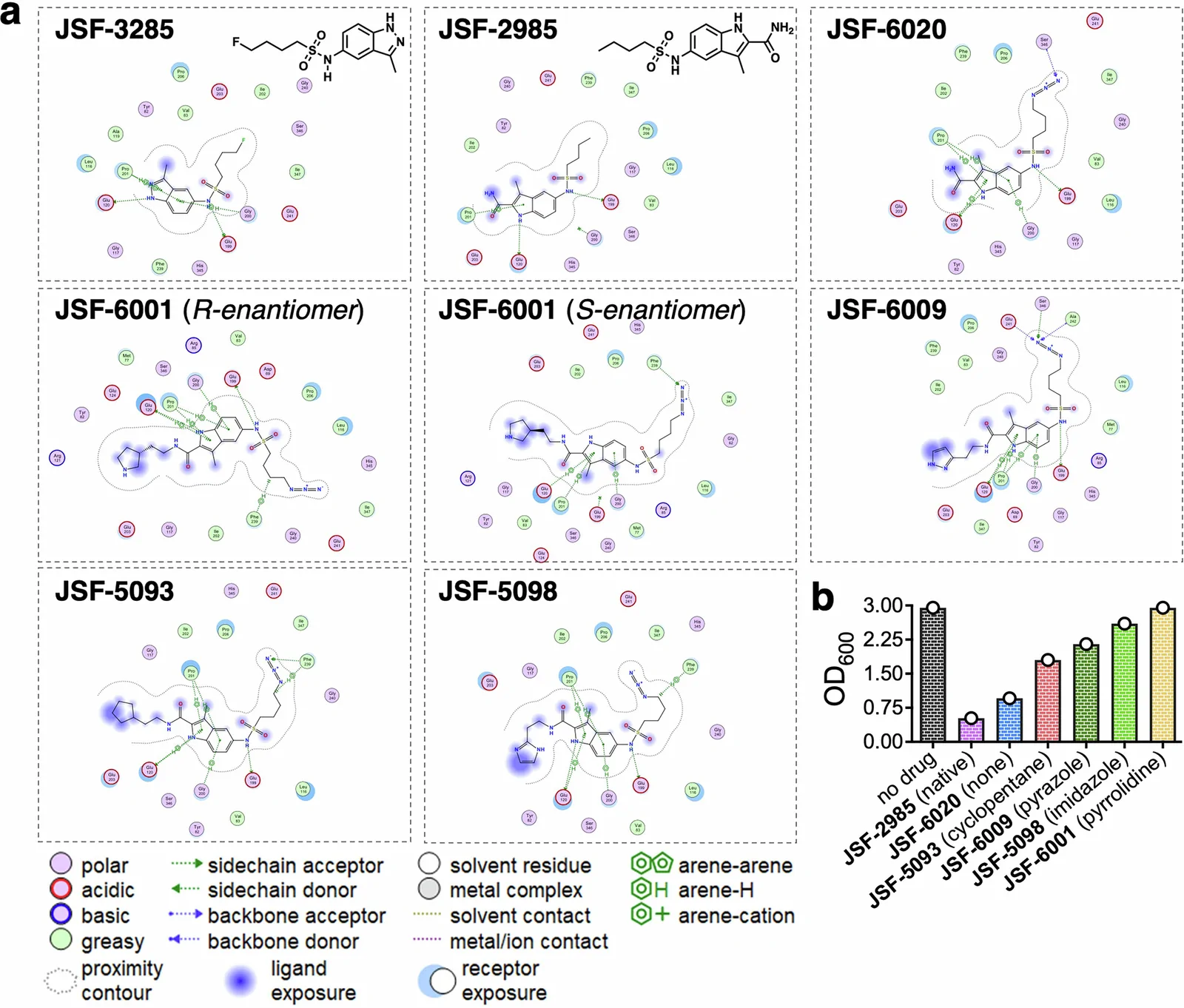
Docking studies of JSF-2985 series with KasA. (**a**) To evaluate potential target interactions of **JSF-2985** azide analogs, molecular docking studies were performed using the X-ray crystal structure of the mycobacterial beta-ketoacyl-ACP synthase I (KasA) with bound inhibitor **JSF-3285** (PDB ID: 6P9L), as the X-ray crystal structure for the KasA- **JSF-2985** complex is not available. **JSF-2985** is a **JSF-3285** analog which has been shown to inhibit *M. tuberculosis* growth (MIC = 3.1 μM). Informed by this X-ray crystal structure, we prioritized three interactions in evaluating the docked poses of prepared azide compounds that are derivative of **JSF-2985**. The first two proposed interactions involve hydrogen bonding of the sulfonamide N-H and the indazole/indole N-H with Glu199 and Glu120, respectively. The third proposed interaction is an arene-H interaction between the indazole/indole ring and Pro201. To further validate the docking poses of the analogs, they were overlaid with **JSF-3285** and the root mean square deviation (RMSD) was calculated. Binding poses that deviated greater than 2.5 Å from the **JSF-3285** X-crystal structure pose and did not participate in the three interactions mentioned above were predicted to be less likely to bind the protein. Such studies suggest that all the investigated **JSF-2985** derivatives have a high probability of retaining the ability to bind their target, although the analysis doesn’t allow to provide details of the strength of target binding, and thus, activity on the enzyme. See Extended Data Table 1 for the exact values of the mentioned parameters. (**b**) Mtb whole cell activity of **JSF-2985** derivatives at 30 μM expressed as optical density (OD_600_) after 5 days of treatment. N = 1 technical replicate representative of 3 biological replicates.

We hypothesized that molecular features identified by cheminformatics or predicted by MycoPermeNet to correlate with mycomembrane permeability are causative. We first tested this hypothesis with a small-molecule series based on **JSF-2985**, an antitubercular molecule we previously reported (Fig. 4a)^37^. We synthesized azide-bearing **JSF-2985** analogues of similar sizes (Fig. 4a) via replacement of the 2-position primary amide’s N-H with 5-membered heterocycles that correlate positively (imidazole, pyrazole and pyrrolidine) or negatively (cyclopentane) with mycomembrane permeation (Fig. 2a,b). We chose the 2-position amide N-H as the location of the new substituents based on docking studies that suggest that the amide group is not directly involved in the engagement of the target but instead lies outside the binding pocket and is thus exposed to the solvent (Extended Data Fig. 5 and Extended Data Table 1). To control for azide reactivity, we exposed DBCO-labelled beads and mycobacteria to different concentrations of azide-test molecules, then calculated the difference in concentration of the test azide molecule that competes fluorescence by 50% in the two systems (Δlog_10_CC_50_). We previously developed Δlog_10_CC_50_ as a relative measurement that ranged from ~0.8 to 2 for more- and less-permeable azide-antibiotics, respectively^7^. Here we found that **JSF-2985** derivatives with imidazole, pyrazole and pyrrolidine scaffolds (Δlog_10_CC_50_ = 1.1–1.3) permeate the mycomembrane better than the **JSF-2985** derivative bearing cyclopentane (Δlog_10_CC_50_ = 2; Fig. 4b,c), consistent with both cheminformatics analyses (Fig. 2a,b) and MycoPermeNet predictions (Fig. 4d).

**Extended Data Fig. 6:**
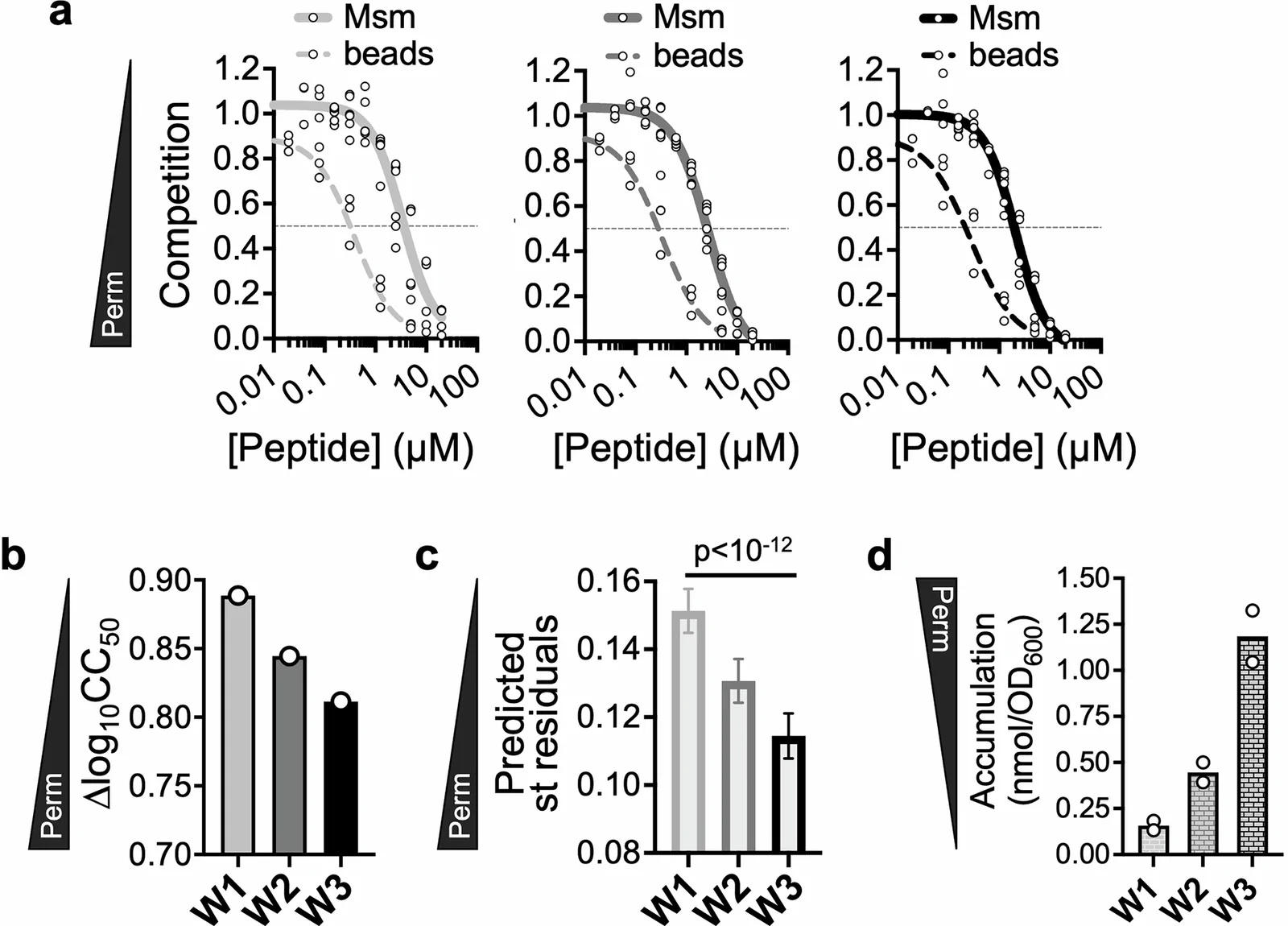
W1-3 pentapeptide permeability in *M. smegmatis*. (**a**) PAC-MAN assay for *M. smegmatis* (Msm) and DBCO-bead and (**b**) related ∆log_10_CC_50_ as measure of mycomembrane permeability. For (**a**) dots represent biological replicates; n = 3 for beads; n = 5 for Msm. (**c**) Predicted values for **W1-3** peptides generated MycoPermeNet trained with Msm permeability data (standard residuals). Data shown as mean and s.e.m. generated by 50 random states of the model. Two-sided pairwise Wilcoxon signed-rank test. Adjustment made for multiple comparisons. (**d**) Whole cell accumulation for **W1-3** peptides determined by Liquid Chromatography in tandem with Mass Spectrometry (LC-MS). Data points represent two biological replicates. Perm: permeability. Source data

We next tested for causation by synthesizing a second small-molecule series based on a pentapeptide (Phe-Lys-Phe-Lys-Phe) in which we systematically substituted phenylalanines for tryptophans (‘**W** peptides’; Fig. 4e). The side chains of tryptophan and phenylalanine respectively bear indole, which is strongly and positively associated with mycomembrane permeability, and benzene, which is weakly and negatively associated with mycomembrane permeability (Fig. 2a,b), and was chosen for its structural similarity to indole. Consistent with both cheminformatics analyses (Fig. 2a,b) and MycoPermeNet predictions (Fig. 4h and Extended Data Fig. 6c), we found that the substitutions enhanced mycomembrane permeation in both Mtb (Fig. 4f,g) and Msm (Extended Data Fig. 6a,b). The benzene to indole swap also resulted in higher overall Msm cell accumulation, as measured by liquid chromatography in tandem with mass spectrometry (LC–MS) (Extended Data Fig. 6d)^38^.

**Fig. 5:**
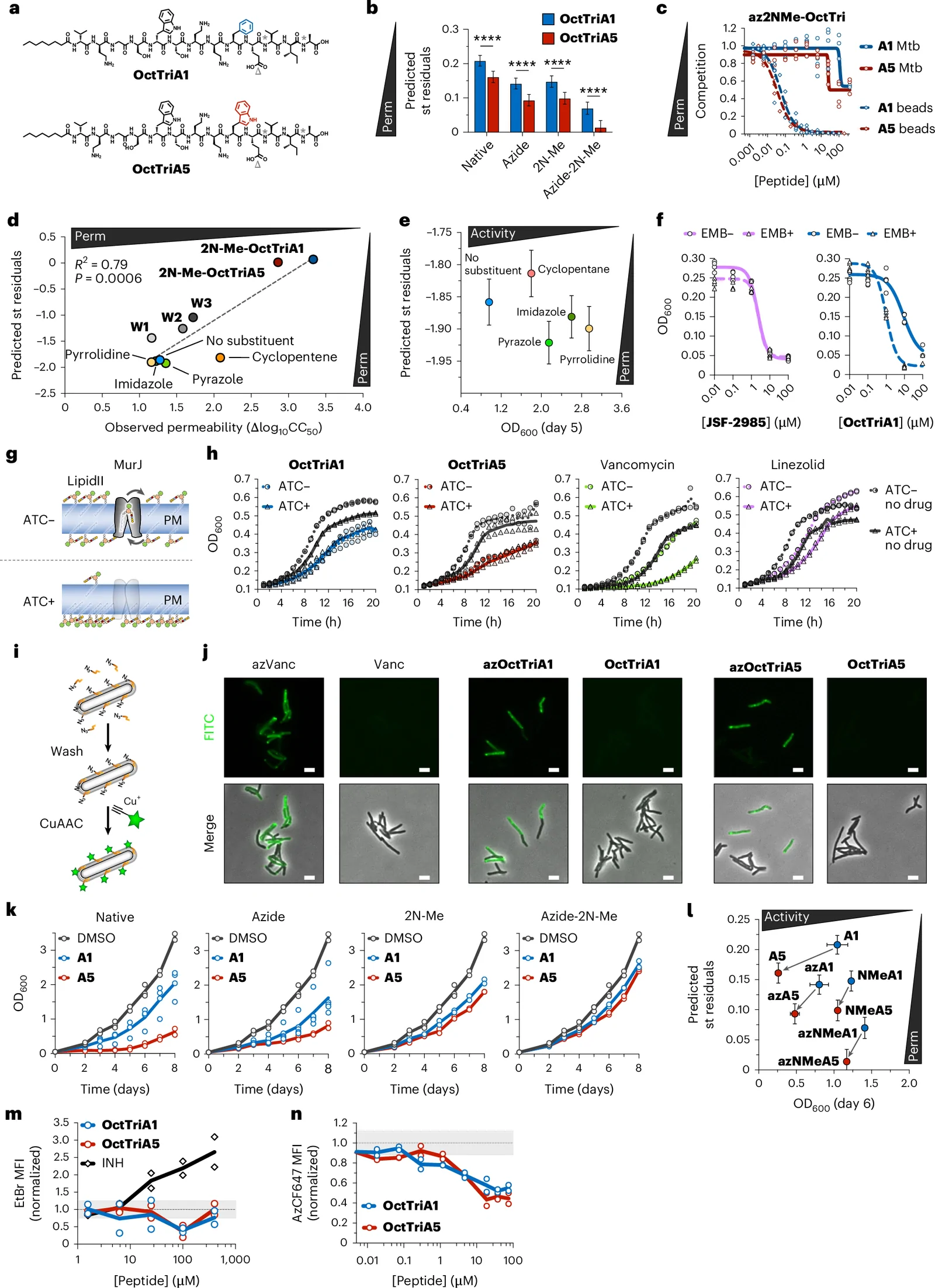
Benzene can promote mycomembrane permeability and anti-Mtb activity of octyl tridecaptins. **a**, Chemical structures of **OctTriA1** and **OctTriA5**. Grey asterisks, *N*-methylation sites; grey triangles, azide derivatization sites (see Extended Data Fig. 7a). **b**, MycoPermeNet predictions (standard residuals) for the **OctTriA1** and **OctTriA5** derivative pairs. Data are mean ± s.e.m. generated by 50 random states of the model. Two-sided pairwise Wilcoxon signed-rank test. *P* values reported as adjusted *P* values across multiple comparisons. All *P*< 10^−10^. ****P < 0.0001. **c**, PAC-MAN results for methylated, azide-modified octyl-tridecaptin derivatives for Mtb and beads (unmethylated derivatives in Extended Data Fig. 7b). Dots represent biological replicates (*n* = 4). **d**, Correlation between observed and ML-predicted mycomembrane permeability across three compound series. Observed permeability values for **OctTriA1** and **OctTriA5** are estimated (see Methods for details). **e**, Predicted permeability does not correlate with anti-Mtb activity (OD_600_ after 5 days of growth) for **JSF-2985** derivatives. *X* axis: individual technical replicate representative of 3 biological replicates (Extended Data Fig. 5b). *Y* axis: data from Fig. 4d. **f**, Mtb growth inhibition curves for **JSF-2985** and **OctTriA1** +/− sub-inhibitory ethambutol (EMB) co-treatment. Dots are technical replicates (*n* = 4) representative of 3 biological replicates. **g**, MurJ depletion (upon anhydrotetracycline [ATC] treatment) and its effect on lipid II availability in the outer leaflet of the plasma membrane (PM). **h**, MurJ was depleted (+ATC) or not (−ATC) in the presence of absence of **OctTriA1** (37.5 μM), **OctTriA5** (12.5 μM), vancomycin (3.1 μM) and linezolid (0.125 μM). Drug concentrations were titrated to achieve similar levels of Msm growth inhibition. For comparison of **OctTriA1** and **OctTriA5** at the same concentration, see Extended Data Fig. 7c. Dots are technical replicates (*n* = 2) representative of 3 biological replicates. **i**, Schematic representation of the copper(I)-catalysed azide-alkyne cycloaddition (CuAAC)-based assay to visualize azide-vancomycin, -**OctTriA5** and -**OctTriA5** and **j**, related fluorescence micrographs from Msm. Scale bars, 3 μm. **k**, Mtb growth curves upon treatment with the four pairs of octyl-tridecaptin derivatives. Dots represent technical replicates (*n* = 2 or *n* = 4 for **OctTriA1** and azide-**OctTriA1**) representative of 3 biological replicates. **l**, Increased predicted permeability from benzene to indole substitutions correlates with enhanced anti-Mtb activity (OD_600_ after 6 days of growth) for OctTriA peptides. MIC: 80 μM for **OctTriA1** and 2.5 μM for **OctTriA5**. Arrows highlight matched **A1** (blue)–**A5** (red) pairs. *X* axis: data from Fig. 5k. *Y*axis: data from Fig. 5b. **m**,**n**, Mtb cell envelope permeabilization by octyl tridecaptins: ethidium bromide (EtBr) uptake (**m**) and accessibility of DBCO-labelled peptidoglycan to an azide fluorophore (**n**) performed as in ref. ^7^. Dots are technical replicates (*n* = 2 for **m** and *n* = 3 for **n**) representative of 2 biological replicates. Source data

**Extended Data Fig. 7:**
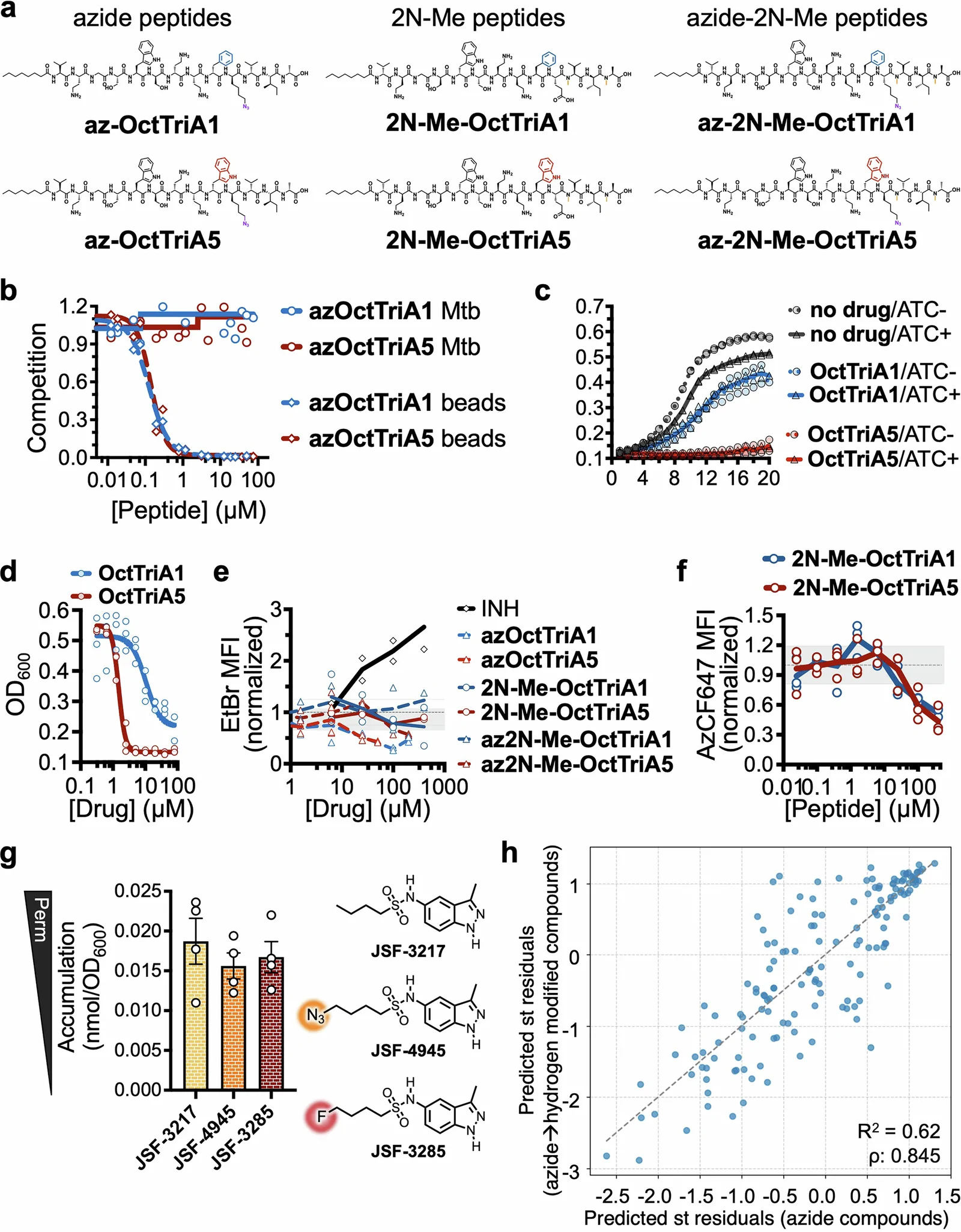
Extended data related to Main Fig. 5. (**a**) Chemical structure of the **azide**-, **2N-methyl-** and **azide-2N-methyl-** versions of **OctTriA1** and **OctTriA5**, antibacterial peptides. (**b**) PAC-MAN results for **azide-OctTriA1** and **azide-OctTriA5** peptides for both Mtb and DBCO-beads. Dots represent biological replicates, n = 2. (**c**) Msm MurJ−mutant growth curves +/- ATC upon treatment with **OctTriA1** and **OctTriA5** at 37.5 mM. Dots are technical replicates (n = 2) representative of three biological replicates. (**d**) Mtb growth (OD_600_) after 7 days of incubation with indicated concentrations of **OctTriA1** and **OctTriA5**. Dots are technical replicates (n = 3) representative of three biological replicates. (**e**) Mtb envelope permeabilization of **azOctTriA1**, **azOctTriA5**, and their 2N-methylated version measured by ethidium bromide (EtBr) assay. Dots are technical replicates (n = 2) representative of two biological replicates. (**f**) Mycomembrane permeabilization of **2N-Me-OctTriA1** and **2N-Me-OctTriA5** assessed by accessibility of DBCO-labeled peptidoglycan to an azide-fluorophore as in (^7^). Dots are technical replicates (n = 3) representative of three biological replicates. MFI: mean fluorescence intensity. (**g-h**) Effect of azide group on compound accumulation and mycomembrane permeation. (**g**) LC-MS whole cell accumulation for the anti-Mtb drug candidate **JSF-3285** and analogs that have either an azide group (**JSF-4945**) or a hydrogen (**JSF-3217**) replacing the fluorine atom. Dots are technical replicates (n = 4) representative of two biological replicates. Error bars: s.e.m. (**h**) Correlation between predicted standard residuals for azide-modified compounds (X-axis) and computationally-derived derivatives in which the azide is replaced with a hydrogen atom (Y-axis). Datapoints represent the compounds in the test set. Perm: permeability. Source data

We next asked whether the promotion of mycomembrane permeation by benzene to indole substitution was generalizable. To test this, we synthesized a third molecule series based on octyl tridecaptin A1 (**OctTriA1**), an antimicrobial peptide^39–41^ that we have shown to have activity against Msm^42^ (Fig. 5a). We replaced the phenylalanine at position 9 with a tryptophan, generating **OctTriA5** (ref. ^40^) (Fig. 5a). Previously, we were unable to detect mycomembrane permeation of azide-**OctTriA1** in Msm unless the backbone of the peptide was *N*-methylated^42^. Thus, we also synthesized derivatives of parent and azide-modified **OctTriA1** and **OctTriA5** with two *N*-methyl groups each (Extended Data Fig. 7a). Across the four pairs, MycoPermeNet predicts better mycomembrane permeation for **OctTriA5** derivatives compared with **OctTriA1** derivatives and for methylated derivatives compared with unmethylated derivatives (Fig. 5b). We were unable to detect the unmethylated azide derivatives via PAC-MAN in Mtb (Extended Data Fig. 7b), as expected from Msm^42^, but found that methylated azide-**OctTriA5** was indeed better able to permeate the Mtb mycomembrane compared with methylated azide-**OctTriA1** (Fig. 5c).

Although we do not yet know the mechanism, our data indicate that benzene to indole substitution can promote ML-predicted and observed mycomembrane permeation in the two molecule series. More broadly, the agreement between ML-predicted and observed permeation across azide derivatives of **JSF-2985,** **W** peptides and methylated octyl tridecaptins (Fig. 5d) suggests that small, ring-containing scaffolds and other chemical features that correlate with mycomembrane permeation (Fig. 2) can be causative.

### Indole association with whole-cell activity

We next asked whether modulating the ability of a molecule to permeate the mycomembrane can likewise impact its whole-cell anti-Mtb activity. For the first series we investigated, **JSF-2985** analogues, we hypothesized that the less-permeable, cyclopentane-bearing derivative (Fig. 4a–d) would inhibit Mtb growth less than the more-permeable imidazole-, pyrazole- and pyrrolidine-bearing derivatives (Fig. 4a–d). Our docking studies (Extended Data Fig. 5 and Extended Data Table 1) suggested that the molecules all have a high probability of target binding. However, we found that Mtb growth inhibition by the **JSF-2985** derivatives did not reflect their mycomembrane permeation profiles (Fig. 5e, Extended Data Fig. 5b and Extended Data Table 1). We and others have found that the cell envelope, including the mycomembrane, is a differential barrier that restricts permeation and whole-cell activity for some antibacterials (for example, vancomycin and moxifloxacin) more than others (for example, linezolid and ciprofloxacin)^7,15,43^. The lack of correlation between mycomembrane permeation and growth inhibition for **JSF-2985** suggested that the mycomembrane might not be an important barrier to its whole-cell activity. Indeed, we found that a sub-inhibitory dose of ethambutol, an antibiotic that indirectly disrupts the mycomembrane^44,45^, does not potentiate **JSF-2985** activity against Mtb (Fig. 5f). Alternatively, or additionally, the small size of **JSF-2985** (309 Da) may have complicated our efforts to modify the molecule with scaffolds, which are relatively large permeability-tuning moieties, without impacting target engagement.

Both MycoPermeNet predictions and experimental data suggest that derivatives of **OctTriA1** are less mycomembrane-permeable than those of **JSF-2985** (Fig. 5d). In *Escherichia coli*, resistance to **OctTriA1** can arise via mutations predicted to impact outer membrane assembly^46^. We hypothesized that the larger size of **OctTriA1** (1,521 Da) might render the molecule more sensitive to membrane barriers than **JSF-2985** as well as enable structure–activity relationship-informed chemical modifications that do not directly interfere with on-target activity^39,41^. In support of the first conjecture, we found that sub-MIC ethambutol sensitizes Mtb to **OctTriA1**, unlike **JSF-2985** (Fig. 5f). **OctTriA1** kills *E. coli* by using the peptidoglycan precursor lipid II in the inner membrane as a receptor from which to dissipate the proton motive force^47^. We therefore addressed the second conjecture by assessing the effect of restricted lipid II availability on **OctTriA1** and **OctTriA5** activities (Fig. 5g,h). We limited the accessibility of the lipid II in Msm via depletion of the essential lipid flippase MurJ^48–50^. MurJ depletion did not impact sensitivity to linezolid, which targets the ribosome, and enhanced sensitivity to vancomycin, which kills bacteria by blocking lipid II incorporation into peptidoglycan. By contrast, MurJ depletion decreased the sensitivity of Msm to both **OctTriA1** and **OctTriA5** (Fig. 5h). Azide-**OctTriA** peptides detected directly by copper(I)-catalysed azide-alkyne cycloaddition (CuAAC; Fig. 5i)^51^ localized similarly to vancomycin, which in mycobacterial species is enriched at cell poles (Fig. 5j)^52^. These data suggest that **OctTriA1** and **OctTriA5** both use mycobacterial lipid II as a receptor and inhibit Msm growth via a mechanism distinct from that of vancomycin.

Given that benzene to indole substitution can promote ML-predicted and observed mycomembrane permeation (Figs. 4g,h and 5b,c), albeit via an unknown mechanism, we wondered whether the modification might also enhance whole-cell activity. Indeed, in the four matched pairs of **OctTriA1** and **OctTriA5** derivatives (with and without azide or methylation modifications), we observed that **OctTriA5** analogues better inhibit the growth of Msm (Extended Data Fig. 7c) and Mtb (Fig. 5k and Extended Data Fig. 7d), albeit to different degrees within the pairs (Fig. 5k,l).

**OctTriA5** permeabilizes red blood cells and *Staphylococcus aureus*, a phenotype neither observed for **OctTriA1** nor for *E. coli*^40^. Surprisingly, in Mtb we found that octyl tridecaptin derivatives decreased general cell envelope permeability, measured by ethidium bromide uptake (Fig. 5m and Extended Data Fig. 7e), as well as mycomembrane permeability (Fig. 5n and Extended Data Fig. 7f), measured by the accessibility of DBCO-labelled peptidoglycan to an azide fluorophore^7^. While we do not yet understand the mechanistic basis for this phenotype, the lack of difference between the matched **OctTriA1** and **OctTriA5** pairs indicates that the benzene to indole substitution is not causative. Thus, this scaffold swap simultaneously promotes mycomembrane permeation and, in the context of the octyl tridecaptins, enhances whole-cell anti-Mtb activity (Fig. 5k,l).

**Fig. 6:**
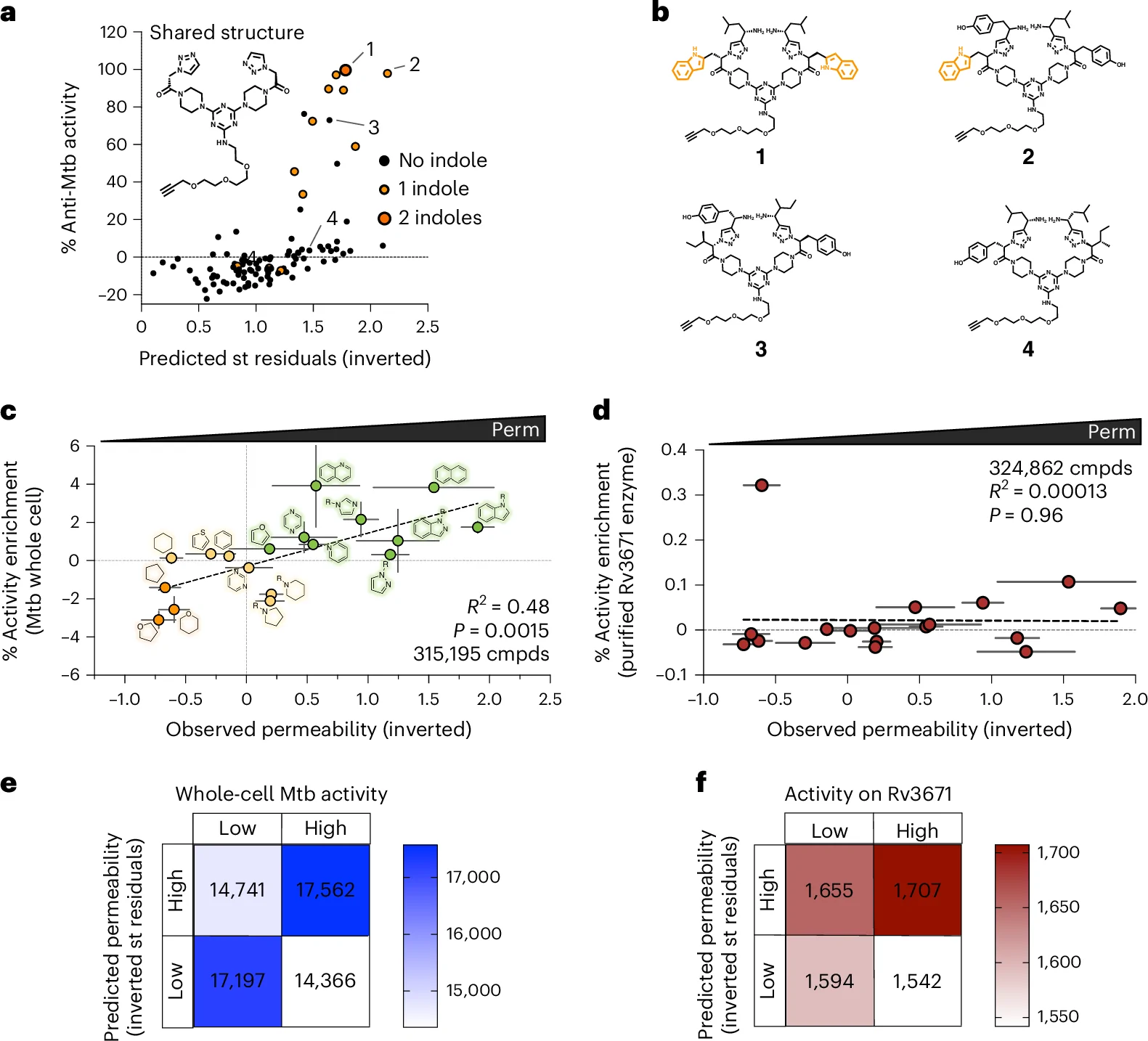
Scaffolds and other chemical predictors of mycomembrane permeability also predict whole-cell activity. **a**, Relationship between whole-cell anti-Mtb activity and ML-predicted mycomembrane permeability (inverted values for more intuitive comparison) for 101 MLSMR compounds that share the indicated structure. The presence of an indole is highlighted in orange. **b**, Four examples of structures from **a**, one for each combination +/− indole and +/− activity. **c**,**d**, Scaffold-specific relationship between activity against whole Mtb cells (**c**) or against a purified Mtb enzyme (serine protease Rv3671c (**d**)) and observed mycomembrane permeation (inverted standardized residuals). *X* axis for both **c** and **d** is median ± s.e.m. of (inverted) observed standard residuals for test azides that share indicated scaffolds (datapoints from Fig. 2a,b). *Y* axis is median ± s.d. of percentage of activity enrichment for MLSMR and TAACF molecules that share the same scaffold relative to all the molecules in the respective datasets (Extended Data Fig. 9). *Y* axis data available from CDD (**c**) or publicly available from PubChem (AID 2606) (**d**). Dashed black line, linear regression (two-sided comparison). **e**,**f**, Relationship between (**e**) whole-cell anti-Mtb activity (MLSMR) or (**f**) purified Rv3671c enzyme inhibition and (inverted) ML-predicted mycomembrane permeation. Each library was divided into quadrants (threshold for permeability: median; threshold for activity: bottom and top 15% of the total values of the libraries (MLSMR) or bottom and top 1% values (Rv3671c) (see Extended Data Fig. 9 for further analyses) and the number of values per quadrant are plotted in **e** and **f**. **e**, Activity range: ≥7.84% (31,928 compounds (cmpds)) and ≤−6.74% (31,938 compounds). *χ*^2^ = 500; *P* < 1.00 × 10^−11^; Cramér’s *V* = 0.0885. **f**, Activity range: ≥13.10% (3,249 compounds) and ≤−45.21% (3,249 compounds). *χ*^2^ = 1.60; *P* = 0.205; Cramér’s *V* = 0.0157. *χ*^2^, Pearson’s chi-squared test of independence. The association in the whole-cell screening is significantly stronger than the association on Rv3671 enzyme using Cramer’s *V*. Source data

**Extended Data Fig. 8:**
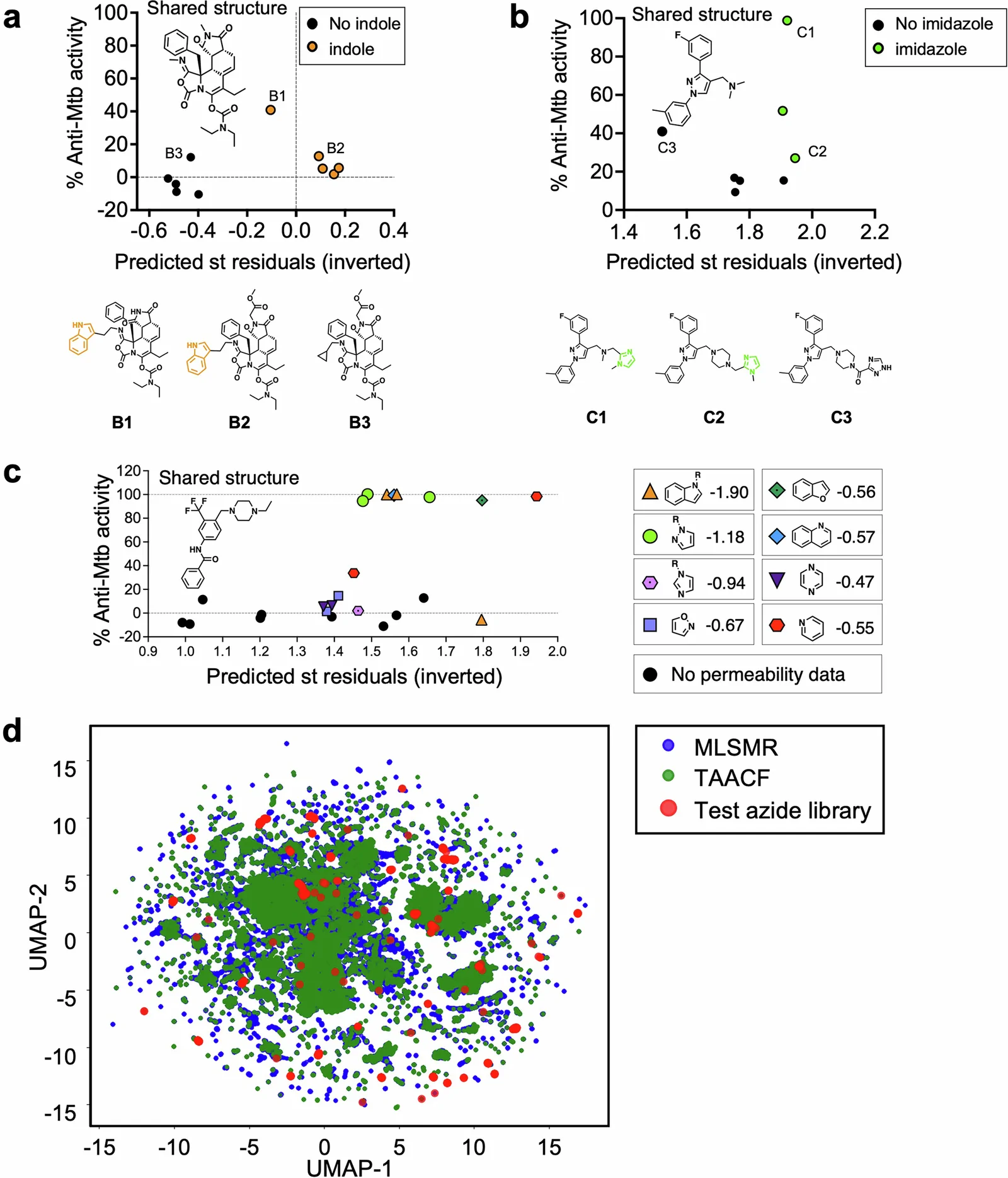
Scaffolds that influence mycomembrane permeation correlate with whole cell anti-Mtb activity. (**a-c**) Three additional sets of compounds subjected to the same analysis as in Fig. 6a. (**a-b**) The presence of indole (orange, (**a**)), or imidazole (green, (**b**)) which are associated with higher mycomembrane permeability (Fig. 2a, b), correlates with higher ML-predicted mycomembrane permeability and whole cell activity. (**c**) The presence of scaffolds (various colors) that are associated with higher mycomembrane permeability (Fig. 2a, b) correlates with higher ML-predicted mycomembrane permeability and whole cell activity. The median mycomembrane permeability for each scaffold (standardized residuals) is shown to the right; lower values indicate higher mycomembrane permeability. (**d**) 2D graph representing the chemical space of the libraries was generated by using Uniform Manifold Approximation and Projection (UMAP) algorithm. Source data

**Extended Data Fig. 9:**
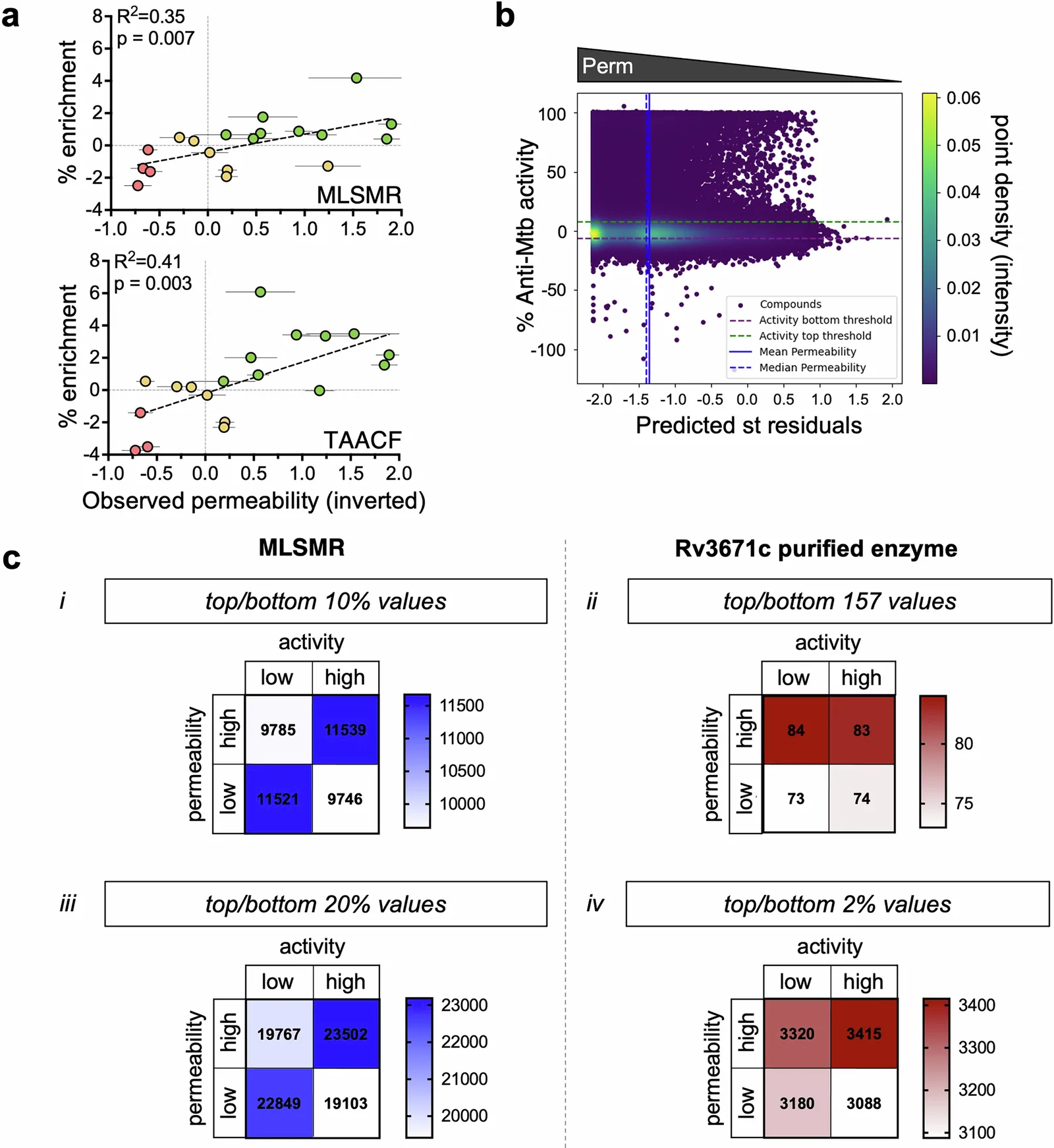
Correlation between predicted mycomembrane permeation and whole cell anti-Mtb activity. (**a**) Scaffold-specific relationship between whole cell activity and observed mycomembrane permeation (inverted standardized residuals) shown separately for MLSMR and TAACF screens (merged in Fig. 6c). X axis is median and s.e.m. of mycomembrane permeability for test azides that share indicated scaffolds (from Fig. 2a). Dashed line: linear regression (two-sided comparison). (**b**) Graphic representation of the analysis performed on MLSMR in Fig. 6e and Extended Data Fig. 9c. MLSMR library data were graphed as ML-predicted permeability (standardized residuals) vs. anti-Mtb activity then divided in quadrants. Threshold for permeability: median. Threshold for activity: 15% top value and 15% bottom values. (**c**) Extended analysis of Fig. 6e, f with additional thresholds as specified in the panels. For the screening on Rv3671c the threshold of activity 37.42% (157 compounds) was defined by the screening’s guidelines (PubChem AID 2606). *χ*^2^ = Pearson’s chi-squared test of independence. Threshold for permeability: median; threshold for activity: bottom and top 15% of the total values of the libraries (MLSMR) or bottom and top 1% values (Rv3671c. (**c**, *i*) Activity range: ≤−8.29% (21306 compounds) and ≥ 11.01%: (21285 compounds). *χ*^2^ = 292.07; p = 1.75 × 10^−65^. (**c**, *ii*) Activity range: ≤−205% (157 compounds) and ≥ 37.42% (157 compounds). *χ*^2^ < 0.0001; p = 1. (**c**, *iii*) Activity range: ≤ −5.51% (42616 compounds) and ≥ 6.00% (42605 compounds). *χ*^2^ = 656.54; p = 8.43 × 10^−145^. (**c**, *iv*) Activity range: ≤ −26.28% (6503 compounds) and ≥ 11.15% (6500 compounds). *χ*^2^ = 2.63; p = 0.105. *χ*^2^ = Pearson’s chi-squared test of independence. The association in the whole cell screening is significantly stronger than the association on Rv3671 enzyme by Cramer’s V. Perm: permeability. Source data

We hypothesized that indole could promote mycomembrane permeation and whole-cell activity in other molecular contexts. To test this, we performed a retrospective analysis on a small-molecule library previously screened for anti-Mtb activity^53,54^. We looked for sets of molecules within the ~200,000 Molecular Libraries Small Molecule Repository (MLSMR; data obtained from the Collaborative Drug Discovery (CDD) Vault database^55^) collection that have similar structures but differ in the presence or absence of indole in peripheral positions, to avoid disrupting the drug core. Because these molecules lack azide tags, we first tested by LC–MS how an azide might impact accumulation of a compound. We found that for **JSF-3285**, an antitubercular molecule related to **JSF-2985** (ref. ^37^), neither removal of fluorine nor substitution with an azide in the same location impacted whole-cell accumulation (Extended Data Fig. 7g). Since the effects of an azide tag may be molecule specific, we next tested the performance of MycoPermeNet on matched compounds with and without the modification (-N_3_ versus -H). The concordance between predictions (Extended Data Fig. 7h) suggested the utility of using a model trained on azide-modified compounds to predict the behaviour of unmodified compounds. In the two distinct molecule sets that met our criteria (Fig. 6a,b and Extended Data Fig. 8a), compound activity correlated with the presence of indole and with ML-predicted mycomembrane permeation (inverted standardized residuals; Fig. 6, and Extended Data Figs. 8 and 9). Together with cheminformatics and ML-based analyses, and **W** peptide and octyl-tridecaptin experiments (Figs. 2a,b, 3c, 4e–h and 5b,c,j), these data indicate that the presence of an indole can predict mycomembrane permeability and whole-cell anti-Mtb activity in a variety of molecular contexts.

### Permeability predictors correlate with whole-cell activity

Finally, we wished to understand more broadly how small, ring-containing scaffolds and other predictors of mycomembrane permeability influence whole-cell activity. We examined two additional MLSMR molecule sets, this time differing in the presence or absence of various scaffolds that predict mycomembrane permeability (Fig. 2a,b). MycoPermeNet predictions and compound activity correlated with the presence of these scaffolds (Extended Data Fig. 8b,c). We then widened our analysis to three different screens: the MLSMR (above) and Tuberculosis Antimicrobial Acquisition and Coordinating Facility (TAACF; data obtained from CDD Vault^55^) small-molecule collections, which were screened against whole Mtb cells, and a third screen against the purified Mtb enzyme Rv3671c (PubChem AID 2606). As in the matched pairs analyses (Fig. 6a,b and Extended Data Fig. 8a–c), the libraries enabled us to examine molecules that lack azide tags and occupy wider chemical space than that covered by our screening libraries (Extended Data Fig. 8d). We found that molecule activity in the whole-cell screens correlates with both observed permeability of different scaffolds (Fig. 6c and Extended Data Fig. 9a) and more generally with MycoPermeNet predictions, which account for physicochemical properties (Fig. 6e and Extended Data Fig. 9b,c). Notably, these correlations were absent in the enzyme screen, a negative control for the mycomembrane barrier (Figs. 6f and Extended Data Fig. 9c). These data suggest that scaffolds and other chemical features that influence mycomembrane permeation also predict whole-cell anti-Mtb activity in large datasets.

## Discussion

Here we find that the effects of physicochemical properties on mycomembrane permeation are scaffold dependent. Furthermore, scaffolds alone, particularly aromatic nitrogen heterocycles such as indole, associate with and/or promote mycomembrane permeation in different molecular contexts. In pharmacology, indole is considered a privileged scaffold, in part because it is highly represented across drug classes and can effectively engage with a variety of targets^56^. Together with the fact that several current antitubercular candidates are indole-based^57^, our findings suggest that indole may also be a privileged scaffold for mycomembrane permeation.

The identification of chemical features that influence mycomembrane permeation may benefit drug discovery efforts in several ways. On the small scale, MycoPermeNet and our interpretability studies on individual molecules (Fig. 3f) may help guide derivatization of lead compounds, although their utility will depend on several factors. First, the mycomembrane is not always a significant barrier for anti-Mtb antimicrobials^7,15,43^ (Fig. 5f). Second, even for molecules for which the mycomembrane poses a major barrier to uptake, improving permeation via derivatization might compromise other aspects of whole-cell activity, for example, target engagement or susceptibility to metabolism and efflux. For example, *N*-methylation enhances observed and predicted mycomembrane permeation of the octyl tridecaptins in Msm and Mtb but activity only against Msm (ref. ^42^; Fig. 5 and Extended Data Fig. 7). By contrast, benzene to indole substitution can enhance permeation and activity of these derivatives in both species (Fig. 5 and Extended Data Fig. 7). We envision that interpretability studies of individual compounds (Fig. 3f) may help tailor permeability-promoting modifications. For example, smaller molecules may be more amenable to subtle changes in physicochemical properties such as TPSA and logP than to larger modifications such as addition or subtraction of ring-containing scaffolds. Beyond derivatization of individual compounds, on the large scale, MycoPermeNet could be applied to molecule collections before or after activity screening to prioritize candidates. In this work, for example, the model enabled us to identify robust correlation between chemical predictors of mycomembrane permeation and whole-cell activity in large datasets.

Mass spectrometry studies that assess whole-cell accumulation have found variability across *E. coli, P. aeruginosa* and *Acinetobacter baumannii*, probably reflecting species-specific differences in molecule transit via porins, diffusion across the outer membrane, and transit via efflux pumps^9^^,10,58,38^. Given that the Mtb mycomembrane has a structure^6,13,14^ and porin-like proteins^59,60^ that are distinct from those of the Gram-negative outer membrane, it is not surprising that the predictors of mycomembrane permeation that we identify here are different from those of Gram-negative bacteria accumulation^9,10,31^. Future use of Mtb strains with targeted disruptions to mycomembrane transporters or structural integrity may enable the identification of pathway-specific predictors of permeation.

By chemically trapping molecules in the periplasm, PAC-MAN enables subcellular localization while avoiding technical problems such as non-specific binding with the mycomembrane and loss of the molecule during sample processing. However, a caveat to the method is the requirement for an azide tag. Azides are widely recognized as ideal bioorthogonal tags because they are small and have minimal known impacts on the physicochemical properties of the parent compound^23,25,61^. While the accumulation of an azide-tagged molecule was similar to that of unmodified compounds by LC–MS (Extended Data Fig. 7g), we do not rule out potential effects of the azide on mycomembrane permeation or activity for other molecules. Indeed, azide functionalization of **JSF-2985** and octyl-tridecaptin derivatives modestly decreased activity (Extended Data Fig. 5b, Extended Data Table 1 and Fig. 5k,l), as we have observed for other antibiotics^62^. However, we note that the chemical features that we identified as predictors of this phenotype from azide-tagged libraries are also correlates of whole-cell activity for untagged compounds (Fig. 6, and Extended Data Figs. 8 and 9). These data are consistent with the concordance of MycoPermeNet predictions for matched compounds modified or not with an azide (Extended Data Fig. 7h), and with our previous finding that sensitivity of azide-modified drug permeation to envelope perturbation tracks closely with that of unmodified parent drug activity^7^. The constancy of the azide across all the test molecules, the large number of test molecules, and the redundancy of some test molecules (that is, compounds that differ only in the position of the azide) may contribute to our ability to predict mycomembrane permeation.

Intrabacterial metabolism presents a challenge for any method measuring molecule accumulation in live cells. For PAC-MAN, we speculate that the 2-h window of molecule exposure may limit intrabacterial metabolism, whether of the azide or of other parts of the molecule, as we have observed that transformation of other molecules in Mtb begins after 2 h^59^.

Intracellular accumulation in Mtb depends on the ability of a molecule to overcome membrane, efflux and metabolism barriers^2,3^. Because PAC-MAN covalently traps test molecules in the periplasm and is sensitive to genetic and chemical perturbations to the mycomembrane^7,15^, we speculate that the assay primarily reports one aspect of accumulation, mycomembrane permeation, although the slow reaction kinetics of SPAAC (second order rate constant of 0.2–0.5 M^−1^ s^−1^ (refs. ^63,64^)) may also permit contributions from inner membrane permeation and efflux processes. As well, our test molecules occupy limited chemical space as they are small and generally abide by the Lipinski’s rule of five^65^. The restrictions on cell compartment and chemical space queried are both a strength and limitation of our work. On one hand, we were able to identify clear structure–function relationships for a well-defined aspect of intracellular accumulation. On the other hand, the structure–function relationships that we identified may need refinement for more complex molecules or for whole-cell accumulation. Expansion of the chemical space covered by our azide libraries, along with whole-cell mass spectrometry^4,5,9–12,58,66–69^, adaptation of PAC-MAN for the cytoplasm^70^, and the use of strains with defined membrane, efflux or metabolism defects^4^^,71,70^ will collectively enable the generation of more comprehensive accumulation models. These, in turn, may be integrated with models for target inhibition, pharmacokinetics and synthetic feasibility to build comprehensive, biologically and chemically interpretable pipelines that aid antibacterial development.

## Methods

### Experimental procedures

#### Bacterial strains and growth conditions

The double auxotroph *M. tuberculosis* (Mtb) mc^2^6206 strain (H37Rv Δ*panCD* Δ*leuCD*^72^), which was provided by Dr William Jacobs, was used for all experiments except the **JSF-2985** analogue MICs, which were performed on the parental H37Rv strain. The mc^2^6206 strain was cultured in Middlebrook 7H9 supplemented with 0.4% glycerol, 10% Middlebrook Oleic Albumin Dextrose Catalase (OADC; BD), and either 0.05% tyloxapol, for strain maintenance, or 0.05% Tween-80, for experimental manipulation. Growth media were additionally supplemented with 50 μg ml^−1^ l-leucine and 50 µg ml^−1^ pantothenic acid. Bacterial stocks were prepared by directly freezing at −80 °C for Mtb culture at optical density at 600 nm (OD_600_) ∼0.5.

*Mycobacterium smegmatis* (Msm) mc^2^155 (refs. ^27,73^) were provided by Dr Anil Ojha and cultured in Middlebrook 7H9 supplemented with 0.5% glycerol (Fisher), 0.05% Tween-80 (Sigma), 10% ADC (ADC: 5% bovine serum albumin, 2% dextrose, 0.003% catalase in water; alternatively, catalase was not included but NaCl 0.9% was added). All bacteria were grown at 37 °C with shaking.

#### High-throughput permeability assay (PAC-MAN) on live Mtb

Mtb was diluted to OD_600_ ∼0.015 and grown in 7H9 +/− 25 μM dibenzocyclooctyne (DBCO)-tetrapeptide (**TetD**^22^, WuXi AppTec) for 4 days until OD_600_ ∼0.25, washed twice with the same medium, then incubated with 50 μM of azide-test molecule in 7H9 in 96-well plates for 2 h at 37 °C with shaking. Azide-test molecules were removed by spinning and supernatant removal, and bacteria were then resuspended in 7H9 containing 17 μM of the fluorogenic label CalFluor647-azide (APC flow cytometry channel) for 1 h at 37 °C with shaking. The fluorophore was removed by spinning and supernatant removal, and bacteria were fixed with freshly prepared 4% paraformaldehyde (PFA, Ted Pella) in polyphosphate buffer (PBS, Genesee) for at least 2 h at room temperature. Then bacteria were centrifuged and resuspended in physiological solution (0.9% NaCl in water) and analysed using a BD DUAL LSRFortessa flow cytometer. The screening of the 40 commercially available azide compounds was performed three times and the mean was used for the subsequent in silico analysis. The screening of the Enamine library was performed two times and a panel of ~60 compounds equally distributed across the library plates was tested a third time. The mean was used for the subsequent in silico analysis. The screening of the Dong/Sharpless azide library was performed once and a panel of ~150 compounds equally distributed across the library plates was re-tested and found to reproduce the first replicate. When applicable, the mean was used for the subsequent in silico analysis.

#### PAC-MAN on Msm

Msm was inoculated from the glycerol stock at a 1:1,000 dilution to 50 ml fresh 7H9 media with ADC in 250 ml Erlenmeyer flasks each day and grown for 24 h until 0.5–0.6 OD. **TetD** (25 μM) were added to the media, and the cells were grown overnight to achieve stationary phase. The cells were collected and spun down for 10 min at 3,000 *g*, washed with 50 ml PBST two times and resuspended in 50 ml PBST. Each day, the azido compounds were prepared at 50 mM in PBST from their dimethyl sulfoxide (DMSO) stocks. To four 96-well plates were added 90 μl of cells, which were then spun down at 2,700 *g* for 2 min with the supernatant decanted to obtain the cell pellets in each well. For each azido compound, 100 μl (50 μM) were added to each well with the cell pellet. The plates were incubated at 37 °C for 2 h. The cells were spun down for 2 min at 2,700 *g*. The supernatant was decanted, and 100 μl 50 μM Az-6-FAM (Lumiprobe, FITC flow cytometry channel) was then added to each well, followed by incubation at 37 °C for 1 h. The cells were then spun down again, and the pellets were washed with 200 μl PBST once and fixed with 100 μl 4% formaldehyde for 15 min. The samples were then analysed using the Attune NxT Acoustic Focusing cytometer.

Alternatively, Msm was grown in 7H9 +/− 25 μM **TetD** for 2 days until OD_600_ ∼0.4, washed twice with PBST and incubated with 50 μM of azide-test molecule in PBST in 96-well plates for 2 h at 37 °C with shaking. Azide-test molecules were removed by spinning and supernatant removal, and bacteria were then resuspended in PBST containing 1 μM of the fluorogenic label CalFluor647-azide (Click Chemistry Tools) for 1 h at 37 °C with shaking. The fluorophore was removed by spinning and supernatant removal, and bacteria were fixed with freshly prepared 2% PFA in PBS, then resuspended in physiological solution (0.9% NaCl in water) and analysed using the BD DUAL LSRFortessa flow cytometer. For values normalization: first, fluorescence values of no-**TetD** controls were subtracted from all the samples. The adjusted fluorescence values were then divided by the fluorescence values from the no-test-azide (vehicle, DMSO) control bacteria. Results were concordant between protocols. The screening of all the libraries was performed three times and the mean was used for the subsequent in silico analysis.

#### PAC-MAN competition curves of W1–3 peptides and JSF-2985 analogues on Mtb

Mtb was diluted to OD_600_ ∼0.015 and grown in 7H9 +/− 25 μM **TetD** for 4 days until OD_600_ ∼0.25, washed twice with 7H9, then incubated with various concentrations of azide-test molecule (or native **JSF-2985**) in 7H9 in 96-well plates for 2 h at 37 °C with shaking. Azide-test molecules were removed by spinning and supernatant removal, and bacteria were then resuspended in 7H9 containing 17 μM of the fluorogenic label CalFluor647-azide for 1 h at 37 °C with shaking. The fluorophore was removed by spinning and supernatant removal, and bacteria were fixed with freshly prepared 4% PFA for at least 2 h at r.t. Then bacteria were resuspended in physiological solution (0.9% NaCl in water) and analysed using the BD DUAL LSRFortessa flow cytometer. Fluorescence values for **W1–3** peptides were normalized as above. Given the permeabilization property of **JSF-2985**, the fluorescence for **JSF-2985** analogues was normalized to that of bacteria treated with the native (azide-free) **JSF-2985** compound (instead of just the vehicle) for each concentration.

#### PAC-MAN competition curves of OctTriA peptides on Mtb

Mtb was diluted to OD_600_ ∼0.015 and grown in 7H9 +/− 25 μM **TetD** for 4 days until OD_600_ ∼0.25, washed twice with PBST, then incubated with various concentrations of the 8 **OctTriA** peptides (**OctTriA1** and **OctTriA5** +/− azide and +/− 2*N*-methylation; Fig. 5c and Extended Data Fig. 7b) in PBST in 96-well plates for 2 h at 37 °C with shaking. Azide-test molecules were removed by spinning and supernatant removal, and bacteria were then resuspended in PBST containing 17 μM of the fluorogenic label CalFluor647-azide for 1 h at 37 °C with shaking. The fluorophore was removed by spinning and supernatant removal, and bacteria were fixed with freshly prepared 4% PFA for at least 2 h at r.t. Then bacteria were resuspended in physiological solution (0.9% NaCl in water) and analysed with the BD DUAL LSRFortessa flow cytometer. The fluorescence for all **azide-OctTriA** analogues was normalized to the fluorescence obtained by bacteria treated with their native, azide-free version for each tested concentration. Since the competition curves on Mtb did not reach 0 for **az2N-Me-OctTriA1** and **az2N-Me-OctTriA5**, to extrapolate and estimate the ∆log_10_CC_50_ between the DBCO-beads curves and the Mtb curves, we renormalized the Mtb data for the curves to reach 0. The estimate is graphed in Fig. 5d.

#### PAC-MAN competition curves of W1–3 peptides and small molecules in Msm

DBCO-modified Msm was generated after incubation with **TetD** as described above. Compounds in different concentrations were prepared on separate 96-well plates. To each well of a 96-well plate was added 90 μl of DBCO-modified Msm followed by spinning down for 2 min at 2,700 *g*, after which the supernatant from each well was decanted to obtain the cell pellets. Compounds (100 µl) in desired concentrations were added to the cell pellets in the working 96-well plate. Each test azide was assayed in technical triplicate. The plates were incubated in 37 °C for 2 h. Bacteria were spun down for 2 min at 2,700 *g*. The supernatant was decanted, and 100 μl 50 μM Az-6-FAM was then added to each well, followed by incubation at 37 °C for 1 h. Bacteria were then spun down again, and the pellets were washed once with 200 μl PBST, then fixed with 100 μl 4% formaldehyde for 15 min. Samples were analysed with the Attune NxT Acoustic Focusing cytometer.

#### OctTriA peptides ethidium bromide full envelope permeabilization assay in Mtb

Mtb was cultured until OD_600_ 0.4–0.6, arrayed in a 96-well plate with the drug of interest at various concentrations, and incubated for 2 h at 37 °C. Bacteria were washed twice with PBS and 0.5% dextrose, and then 5 μg ml^−1^ ethidium bromide (EtBr, Biorad) in PBS and 0.5% dextrose was added. After 1 h of incubation at 37 °C, EtBr solution was removed by spinning and bacteria were fixed as above. Bacteria were resuspended in physiological solution (0.9% NaCl in water) and analysed with the BD DUAL LSRFortessa flow cytometer (fluorescence channel: PE-Texas Red).

#### OctTriA peptides mycomembrane permeabilization assay by peptidoglycan accessibility of an azide fluorophore in Mtb

Mtb was diluted to OD_600_ ∼0.015 and grown in 7H9 +/− 25 μM **TetD** for 4 days until OD_600_ ∼0.25, washed twice with PBST, then incubated with various concentrations of the 4 **OctTriA** peptides lacking azide (**OctTriA1** and **OctTriA5** +/− 2*N*-methylation) in PBST in 96-well plates for 2 h at 37 °C with shaking. The drug molecules were removed by spinning and supernatant removal, and bacteria were then resuspended in PBST containing 17 μM of the fluorogenic label CalFluor647-azide for 1 h at 37 °C with shaking. The fluorophore was removed by spinning and supernatant removal, and bacteria were fixed with freshly prepared 4% PFA for at least 2 h at r.t. Then bacteria were resuspended in physiological solution (0.9% NaCl in water) and analysed with the BD DUAL LSRFortessa flow cytometer. The fluorescence for **OctTriA** analogues was normalized to that of the untreated control.

#### Azide-OctTriA peptide association with Msm envelope and detection via CuAAC

Msm was grown until OD_600_ ~0.6 and treated with 10 μM **azide-OctTriA1, azide-OctTriA5** or azide-vancomycin^7^, and their native counterpart as a control, for 2 h in 7H9. Bacteria were then washed twice in PBST and resuspended in the click reaction mix (1.5 mM CuSO4, 1.5 mM BTTP, 10 mM sodium ascorbate [60 mM stock solution freshly prepared in PBS], 50 μM AZDye488-alkyne [Vector laboratories], in PBS)^51^ and incubated with shaking at 37 °C for 30 min. The cells were washed twice with PBST and fixed for 10 min in 2% PFA. Bacteria were resuspended in physiological solution (0.9% NaCl in water) and imaged by fluorescence microscopy.

#### Fluorescence microscopy

Bacteria labelled as described above were prepared on agar pads and imaged with an inverted Nikon Ti2-E wide-field fluorescence microscope with a Nikon Plan Apo λ ×60 1.45 numerical aperture objective lens. Images were rendered in FIJI.

##### DBCO modification of polystyrene beads

Amino functionalized polystyrene beads (1 ml) (5% w/v, 5 mg, Spherotech) were spun down at 21,000 *g* for 15 min in a 1.7-ml microcentrifuge tube and washed with 1 ml deionized water before use. The beads were then spun down and resuspended in 10 ml 100 mM sodium borate buffer (pH 9) with 1 μg ml^−1^ DBCO-*n*-hydroxysuccinimide(NHS) ester (Vector laboratories) and reacted at 37 °C for 2 h with shaking. The resulting beads were spun down at 4,000 *g* for 10 min, washed once and resuspended in sodium borate buffer. Acetic anhydride (200 μl) was added to the suspension and reacted at 37 °C for 2 h with shaking. The resulting product was then spun down and washed twice with 10 ml sodium borate buffer and twice with 10 ml PBS and resuspended in 20 ml PBS for further use.

##### Competition with the test molecules on DBCO-modified polystyrene beads

DBCO-modified polystyrene beads were diluted at 1:20 in PBS before adding to the assay. To a MultiScreen HTS 96-well filter plate with 0.65 mm hydrophilic PVDF membrane, we added 100 μl of beads to each well, which was then filtered under vacuum to get the pellet of beads on the filter membrane. Test molecules (50 μM) in the library were added to the bead’s pellets in each well at a final volume of 100 μl, and the pellets were resuspended with volumetric pipettes. The plates were incubated at 37 °C for 2 h and then washed with 200 µl PBS, filtered under vacuum to remove the supernatant. Az-6-FAM (100 μl, 50 μM) was then added to each well. The plates were then incubated in 37 °C for 1 h. The beads were washed with 200 μl PBS twice by vacuum filtration and then resuspended in 200 μl PBS. The samples were then analysed using the Attune NxT Acoustic Focusing cytometer. Alternatively, DBCO-modified polystyrene beads were diluted at 1:20 in PBST and incubated with the proper concentration of azide-test molecule in 96-well plates for 2 h at 37 °C with shaking. Azide-test molecules were removed by spinning and supernatant removal, and beads were resuspended in PBST containing 1 μM of the fluorogenic label CalFluor647-azide for 1 h at 37 °C with shaking. The fluorophore was washed and resuspended in physiological solution and analysed with the BD DUAL LSRFortessa flow cytometer. Results were concordant between protocols. The screening of the Dong/Sharpless and Enamine libraries was performed once. The screening of the 40 commercially available azide compounds was performed three times and the mean was used for the subsequent in silico analysis.

#### JSF-2985 analogues growth inhibition against Mtb

Mtb was diluted to OD_600_ 0.01 and incubated with 30 μM **JSF-2985** analogues (or just vehicle, DMSO) at 37 °C for 5 days and OD_600_ was measured. A concentration of 30 μM was chosen because of the **JSF-5093** precipitation issue at higher concentrations.

#### OctTriA peptides growth inhibition against Mtb

Mtb was diluted to OD_600_ 0.01 and incubated with 5 μM of the 8 **OctTriA** peptides (**OctTriA1** and **OctTriA5** +/− azide and +/− 2*N*-methylation; Fig. 5c and Extended Data Fig. 7b) (or just vehicle, DMSO) at 37 °C for 6 days and OD_600_ was measured.

#### Dose–response growth inhibition curve of Mtb for OctTriA1 and OctTriA5 peptides

Mtb was grown until OD_600_ ~0.6, back diluted to OD_600_ 0.01, arrayed in 96-well plate in triplicate and treated with increasing concentrations of **OctTriA1** or **OctTriA5** at 37 °C for 7 days. Bacteria were then fixed by adding the necessary volume of 16% PFA to reach a final concentration of 4% and incubated at r.t. for at least 2 h. OD_600_ was measured using a BioTek Epoch plate reader and related BioTek Gen5 software. Minimum inhibitory concentration (MIC) was determined as the first concentration where no growth was observed.

#### Dose–response growth inhibition curve of Mtb for OctTriA1 peptides and JSF-2985 in combination with ethambutol

Mtb was grown until OD_600_ ~0.6, back diluted to OD_600_ 0.05, arrayed in 96-well plate in triplicate and treated with increasing concentrations of **OctTriA1** or **JSF-2985**, with or without co-treatment with 0.5 μg ml^−1^ ethambutol, and incubated at 37 °C for 5 days. Bacteria were then fixed by adding the necessary volume of 16% PFA to reach a final concentration of 4% and incubated at r.t. for at least 2 h. OD_600_ was measured using a BioTek Epoch plate reader using a BioTek Gen5 software.

#### Growth inhibition curves of Msm MurJ depletion strain upon treatment with OctTriA peptides or antibiotics

Msm MurJ depletion strain (inducible protein degradation system^49^) was grown up to OD_600_ ~0.2 and then treated or not with 50 ng ml^−1^ anhydrotetracycline (ATC) for 4 h. Bacteria were then diluted to OD_600_ 0.1, arrayed in 96-well plate, and treated with **OctTriA1** (37.5 μM), **OctTriA5** (12.5 or 37.5 μM), vancomycin (3.1 μM) or linezolid (0.125 μM) as specified in the Fig. 5 legend. The plate was analysed using a BioTek Synergy2 plate reader by incubating at 37 °C with mild shaking for 20 h, with an OD_600_ read every hour. Data were collected using BioTek Gen5 software.

#### JSF-2985 analogue MICs for *M. tuberculosis* H37Rv

Determination of MIC values was done using the microplate Alamar blue assay (MABA). Serial dilutions of test compounds in 100 µl were prepared in growth media (7H9-ADS (albumin-dextrose-sodium chloride)) in a 96-well microplate (Fisher, FB012932). Each well was added with 100 µl of Mtb culture (parental H37Rv strain diluted 1:1,000 from an initial OD_595_ of 0.2). After 7 days incubation at 37 °C, alamarBlue HS Cell Viability Reagent (Invitrogen, A50101) supplemented with 20% Tween-80 was added. Plates were incubated for an additional 24 h, followed by absorbance readings at 570 nm (normalized to 600 nm) to assess bacterial viability^74^.

#### Mass spectrometry-based intrabacterial accumulation in Msm

##### Sample preparation

Msm was grown in 7H9 broth supplemented with 10% OADC and 0.05% Tween-80 at 37 °C and shaken at 180 r.p.m. until an OD_600_ of 0.5–0.6 was reached. The bacterial culture was divided among a series of flasks, one for each compound, with a final concentration of 50 µM. The compound-treated cultures were incubated at 37 °C with shaking at 180 r.p.m. for 2 h, 4 × 1 ml samples were taken in 1.5-ml microcentrifuge tubes, and the culture OD_600_ was measured. Each sample was centrifuged at 5,000 × *g* for 10 min at 4 °C, and the pellet was washed with 1 ml 0.85% NaCl aqueous solution, followed by centrifugation at 9,000 × *g* for 10 min. The washed pellet was then quenched with an equal volume of 2:2:1 CH_3_CN/MeOH/H_2_O (MAW) and the resulting cell suspension was transferred to bead tubes (lysing matrix B, 1169110-CF), lysed by bead beating with a Mini-Beadbeater-96 (BioSpec) for six 30-s cycles at 2,400 r.p.m., incubating for 2 min on ice between cycles to prevent overheating of the samples. Following bead beating, samples were centrifuged at 9,000 × *g* for 10 min at 4 °C, after which 600 µl of each supernatant was transferred to a 0.22-µm filter tube (Costar SpinX tube, 8160) and further centrifuged at 16,000 × *g* at 4 °C for 10–15 min. Subsequently, the flow-through was kept at −80 °C until analysis by LC–MS.

##### LC–MS-based quantification of drug accumulation

The samples were analysed using an Agilent 1290 Infinity II HPLC system, coupled to an Agilent InfinityLab LC/MSD system, with a Poroshell 120 EC-C18 column (2.1 × 50 mm; 1.9 µm particle size). The solvent system utilized was water/acetonitrile acidified with 0.1% formic acid using the typical gradient programme (expressed as the percentage of acetonitrile): 0–0.4 min, 5%; 0.4–0.5 min 5%–30%; 0.5–3.0 min, 30%–95%; 3.0–3.3 min, 95%; 3.3–3.6 min 95%–5% at a flow rate of 0.5 ml min^−1^, with a post-run segment of 1 min. Before analysing the biological samples, we confirmed the robust peaks and retention times for the compounds and verapamil (the typical internal standard used in the study) in standard mixtures of the compound in MAW, and the lower limit of detection (LLOD) was determined. The LLOD for each compound was defined as the lowest concentration of the compound that could be detected and distinguished from the baseline such that the analyte peak height was ≥2× the height of the baseline. The peak area ratio of the compound to internal standard was plotted against the compound concentration to calculate the slope of the fitted line by the linear least squares method. Data processing and quantification of ions were conducted as previously published^75^.

### In silico analysis and experiments

#### Cheminformatics

##### Data cleaning

To reduce the effect of DBCO-beads reactivity on apparent accumulation, the residual of apparent accumulation against the logarithm of bead reactivity for each molecule was calculated. First, values above 1 were clipped to 1, and values lower than 0 were clipped to 0. Then, a simple linear regression was performed using NumPy.polyfit() with a degree of 1, and residuals were calculated according to equation (1):

1 $$r={y}_{i}-\hat{{y}_{i}}$$

In equation (1), *y*_*i*_ refers to the measured accumulation, and *ŷ*_*i*_ refers to the accumulation predicted by the line-of-best-fit from the linear regression. Next, residuals were standardized according to equation (2) to compare the residuals of different compound libraries.

2 $${r}_{\mathrm{std}}=(r-\mu )/\sigma$$

In equation (2), the standardized residual for a compound, *r*_std_, is equal to the original residual, *r*, minus the mean residual among all compounds of that compounds library, *µ*, divided by the standard deviation among all compounds of that compounds library, *σ*. This equation is also known as the *z*-score of the residual. To combine libraries, we saved the standard residuals in a Pandas data frame, and, looping through each library, added the molecules to a new data frame. If we came across a molecule common between two or more libraries, we took the standardized residual value from the 40-compound or 380-compound library.

##### Scaffold analysis

To analyse how the presence of various ring structures impacts the apparent accumulation, we binned compounds into groups on the basis of whether they had at least one instance of each ring in their structure. The ring structures chosen are shown in Fig. 2a and Extended Data Fig. 3a for which the analysis was performed using the CDD Vault database (www.collaborativedrug.com). For the analysis in Fig. 2b, to further classify compounds, a distinction was made between ring structures that were part of a larger ring structure (FUSED) and those that were not (NONFUSED). These groupings were then used to plot the distribution of standardized residuals in violin plots using Graphpad Prism.

##### Physicochemical property correlation analysis

To analyse how physicochemical properties of the compounds might affect the apparent accumulation, various properties were calculated with the rdMolDescriptors package within RDKit. The properties calculated were number of hydrogen bond acceptors (HBA), number of hydrogen bond donors (HBD), HBA + HBD, number of rings (nRings), number of rotatable bonds (RTB), number of amide bonds (nAmideBonds), globularity, plane of best fit (PBF), topological polar surface area (TPSA), ipophilicity (logP), molar refractivity (MR), molecular weight (MW), number of Csp^3^ hybridized carbons (Csp3), fraction of heavy atoms in the molecular scaffold (fmf), quantitative estimation of drug-likeness (QED), heavy atom count (HAC), number of fused rings (nRingsFused), number of unique hydrogen bond donors and acceptors (uniqueHBAD), maximum ring size (MaxRingSize), number of chiral centers (nChiralCenters), fraction of Csp^3^ hybridized carbons in the molecular scaffold (fcsp3_bm), formal charge (FormalCharge) and absolute charge (AbsoluteCharge). We further calculated 2 additional properties from the CDD Vault visualization tool: *n*-octanol/water distribution coefficient at log pH 7.4 (logD) and water solubility (logS), resulting in a total of 25 physicochemical properties. If any of the properties could not be calculated for a compound, that compound was discarded. The correlation matrix for the properties, along with raw apparent accumulation, logarithm of bead reactivity, original residuals and standardized residuals, was calculated using the ‘Pandas DataFrame.corr()’ function. Properties without valid correlation values were discarded. The correlation matrix was visualized as a heat map using Matplotlib.

##### Effect of a permeability-promoting scaffold within molecule sets with common minimal structure

The analyses were performed using the CDD Vault software in specific datasets (see below).

##### Indole

Indole-containing molecules were searched within the MLSMR^53^ library screened for anti-TB activity, applying a bottom MW threshold of 500 Da (~4–5× the indole MW of 117.15 Da). We performed a structural search for molecules where indole was present as a peripheral substituent instead of the main core. Groups where indole occupied a central position and, thus, could not be removed to perform a comparative analysis +/− indole, were not considered. We prioritized compounds that showed structural similarities (shared core structure) within the indole-containing subgroup. For each chosen molecule/group, the indole first, and subsequently, additional peripheral parts of the molecule, were removed from the structure to identify a ‘minimal structure’ which was then searched for again within the entire MLSMR dataset, defining the group for the +/− indole analysis. This was a reiterative process to obtain as many compounds as possible for each group, until the structure became too small to be unique. Given the lack of information about the molecular targets relevant to the library, sets of molecules were chosen (≥7 molecules) to inform structure–activity relationships and decrease the likelihood that indole was directly involved in the target engagement. For the same reason, we prioritized structures for which the scaffold of interest represented a small portion of the molecular structure (that is, one-fifth of the total MW or less). Around 20 minimal core structures were analysed and only the ones that met the abovementioned criteria were selected. All the chosen sets of molecules present examples of drug structures missing the scaffold of interest but showing activity, suggesting the role of the scaffold as an auxophore instead of a pharmacophore.

##### Imidazole

The same analysis described above for indole was performed for imidazole-containing compounds, but the bottom threshold of MW was set as 300 Da, proportional to imidazole MW of 68.8 Da. The results in Extended Data Fig. 9c were obtained using the threshold for imidazole.

##### Correlation between predicted permeability and activity in big published datasets

To investigate relationships between permeability and biological activity, we used previously published drug libraries screened for whole-cell anti-TB activity (MLSMR), where mycomembrane permeability can affect activity, and the screening against the purified enzyme Rv3671 (PubChem AID 2606) where mycomembrane permeability is not a factor in activity. This analysis aims to test the hypothesis that less-permeable compounds are also less active and vice versa. Since we do not know the level of correlation between mycomembrane permeability and activity, we were looking for major statistical differences between the dataset where mycomembrane permeability is a relevant factor for activity (MLSMR) versus the one for which it is not (purified Rv3671enzyme).

First, we predicted the permeability of the entire MLSMR and Rv3671 libraries using MycoPermeNet. We divided the libraries into quadrants for high versus low predicted permeability and high versus low activity. For predicted permeability, we categorized on the basis of the mean permeability for each dataset individually: values below the mean were classified as ‘high permeability’ and those above the mean as ‘low permeability’, due to the inverted nature of our permeability residuals (that is −3 indicates highest permeability and 3 indicates lowest permeability).

The activity thresholds were defined differently for the two libraries given the nature of the screening and the dataset features.

Screened libraries activity data distribution

MLSMR screening (percentage of growth inhibition at 10 μM): mean 3.525; standard deviation 18.485; minimum −117.650; maximum 105.380; median −0.130;

Purified Rv3671 enzyme screening (percentage of inhibition at 5.96 μM): mean −2.353; standard deviation 13.199; minimum −776.820; maximum 511.750; median −0.850.

MLSMR anti-TB screening was performed using the AlmarBlue reagent^74^, while for the in vitro enzyme screening, a competitive activity-based protein profiling assay based on fluorescence polarization was used^76^.

Both datasets show a large number of negative values for activity, which is probably due to experimental noise. To obtain a reasonable threshold without generating a strong imbalance between the amount of datapoints for the active and the inactive compounds, we chose a defined percentage of the total values, rather than selecting a percentage cut-off. Since the activity ranking among the negative values is meaningless, we chose percentage of values that provided balanced thresholds for high and low active compounds close to 0.

Both libraries’ values were ranked by activity, and the same numbers of high and low activity values (as a percentage with respect to the whole dataset) were selected: 10%, 15% and 20% for MLSMR; 157, 1% and 2% for the purified Rv3671 enzyme screening. The threshold that led to 157 compounds was chosen following the screening directions for the activity threshold (>37.42%).

We carried out a series of statistical tests on both datasets to ensure statistical significance. Statistical experiments were done using the SciPy package (v.1.13.1).

After classification into groups, we assessed the association between permeability and biological activity using a contingency table that compares the permeability category (rows) and the activity category (columns). A chi-squared test for independence was performed on this table to determine whether these categories were significantly associated, yielding a chi-squared statistic (*χ*^2^), *P* value and degrees of freedom for an all-around assessment (see Fig. 6e and Extended Data Fig. 9c for details).

For MLSMR, the groups ‘high permeability/high activity’ and ‘low permeability/low activity’ showed a strong enrichment compared to the other two, whereas for the purified Rv3671 enzyme, a small enrichment was observed for the high permeability/low activity group.

To further validate association strength to distinguish between the two libraries, we carried out Cramér’s *V* test to evaluate the statistical association strengths of the libraries. For the MLSMR library, we observed a very strong association relative to the purified enzyme library.

##### Docking studies of **JSF-2985** analogues with KasA

The X-ray crystal structure of **JSF-3285** bound to the KasA protein was retrieved from the RCSB Protein Data Bank (PDB 6P9L ref. ^37^). The protein structure was prepared for docking simulations using the QuickPrep function in the Molecular Operating Environment (MOE) software package^77^. Structure preparation was used to correct topological errors in protein residues. Hydrogen atoms were added using Protonate3D and water molecules greater than 4.5 Å away from ligand atoms were deleted. Tethers were installed on binding pocket, ligand and solvent atoms present in the binding pocket to limit their deviation from the experimentally determined coordinates during simulations. Atoms further away from the binding pocket were fixed to increase efficiency during simulation. Refinement and energy minimization of the protein were performed using the Amber:EHT force field. Ligands were minimized using the MMFF94× force field for small molecules while keeping the protein fixed. Analogues were built using the Builder function in MOE. For the docking calculations, 30 poses were generated for each ligand and docked using the Triangle Matcher placement function. A combination of the GBVI/WSA Δ*G* and the London Δ*G* scoring functions were used. The top five scoring conformations were evaluated by overlaying them with the lowest-energy docked **JSF-3285** conformation.

#### Machine learning

##### Dataset preparation

The 40, 380 and 1,152 azide libraries were standardized and merged using the standardized residual calculation described above. The compounds were then represented as SMILES strings^78^—a standard textual representation of the compounds used for cheminformatics. Defective SMILES that could not be properly parsed by the RDkit tool^29^ were removed, resulting in a final dataset of 1,558 samples with corresponding permeability residuals labelled as ‘Mtb standardized residuals’. Values range from −3 (most permeable) to 3 (least permeable). The dataset was then split into training, validation and test sets using the Bemis–Murcko scaffold-balanced strategy implemented using Chemprop^79,80^. This procedure ensures that compounds with the same molecular scaffold are not shared across data splits, which could result in overestimating model performance. This resulted in 217 unique scaffolds, with the test set containing 157 samples.

##### Embedding space generation

We employed a message-passing neural network (MPNN) architecture implemented in the Chemprop framework (v.1.6.1) to generate molecular embeddings. MPNN operates in two phases, the first being the message-passing phase which propagates information across the molecular graphs, ultimately building a neural representation of the whole graph. The other phase is the readout phase in which the neural representation generated from the message-passing phase is used to predict the property of interest, in our case, the permeability residuals. This readout phase ensures that the generated embedding space is useful for predicting downstream properties. Chemprop implements a directed message-passing neural network, a type of MPNN, which centres the message passing on bonds and gathers information on neighbouring bonds^81^.

Hyperparameter tuning was carried out after testing various architectures, and the following hyperparameters gave the best performance on the validation set. We selected an MPNN with a depth of 3, where each layer consisted of a series of graph convolutions followed by a rectified linear unit (ReLU activation function. The MPNN encoder was configured with a hidden size of 300, utilizing a linear layer without bias to map input features (147-dimensional) to hidden representations. The output of the MPNN encoder was further processed through a readout function, consisting of a series of linear layers with ReLU activations, to produce the final molecular embedding.

The model was trained for 30 epochs with a batch size of 50. The initial learning rate was set to 0.001 and a warm-up period of 2 epochs. The optimization was performed using the Adam optimizer, and the loss function was defined as the mean squared error (MSE) between the predicted and actual permeability residuals. Manual inspection of the loss curves was performed to prevent overfitting, with validation stability observed around the 13th epoch. We employed a single model ensemble, with no dropout regularization applied during training. No additional atom or bond descriptors were provided other than the input SMILES strings. This was done for multiple 90–10 splits of the train/validate datasets (7×) and model initialization states (7×), and the mean performance and standard error measurement on the validation set were reported across the 49 different runs.

Model performance was evaluated using root mean squared error (RMSE) as the primary metric. RMSE was calculated on the validation set at regular intervals, with model checkpoints saved after each epoch. The final model’s performance on the test set was used to report the predictive accuracy of the MPNN-generated embeddings for downstream permeability prediction tasks. The final model consisted of 355,201 parameters.

##### Downstream machine learning modelling

The embeddings generated from the MPNN were employed as input features for a downstream machine learning task to predict permeability residuals.

Three models were evaluated for their effectiveness at predicting permeability residuals from the MPNN-generated embeddings after hyperparameter tuning (Extended Data Fig. 4a). All downstream models were built within the Scikit-learn framework^80^ (v.1.3.2). A multilayer perceptron (MLP) demonstrated best performance on the validation set coefficient of determination (*R*^2^) score, hence this model was selected for downstream use (Extended Data Fig. 4b). While other models did achieve higher performance on the training set, their worse performance on the validation set indicates that they were overfit. To verify the need for embeddings, we also trained the three models on input features generated by RDKit and by extended-connectivity fingerprint (ECFP), which provide fixed representations of chemicals on the basis of pre-determined rules. Models trained on fixed representations performed worse than embeddings-based models (Extended Data Fig. 4a).

The MLP was configured with four hidden layers consisting of 300, 200, 32 and 16 neurons, respectively. The activation function used for all hidden layers was the ReLU function. To control overfitting, L2 regularization was applied with an alpha parameter set to 0.01. The learning rate was adaptive, with an initial value of 0.01, and the model’s optimization was performed using stochastic gradient descent.

The model was trained for a maximum of 400 epochs, with early stopping implemented at this stage to halt training when the validation loss did not improve for 10 consecutive epochs. The early stopping criterion was met for all models. During each epoch, the model was trained on the training set, and predictions were generated for both the training and validation sets (Extended Data Fig. 4b). The MSE was computed for both sets to monitor performance and detect the optimal stopping point. This was done for multiple 90–10 splits of the train/validate datasets (7×) and model initialization states (7×), and the mean performance and standard error measurement on the validation set were reported across the 49 different runs.

To identify the optimal model, the epoch with the lowest validation loss was selected. This model was then retrained from scratch for the corresponding number of epochs using the training set. The final model was evaluated on both the validation and test sets to ensure that its performance generalized well to unseen data. The training and validation loss values were recorded across epochs to visualize the training process and confirm the model’s convergence. Parity plots were generated to compare the predicted permeability residuals against the actual values. The *R*^2^ scores of all models were ranked and the median performing model was selected for further visualization. We show parity analysis between the actual experimental residuals and the model-predicted residuals (Fig. 3b). All machine learning plots provided in this study were made using Matplotlib (v.3.10.0).

We interrogated the uncertainty of our model by repeating model training with 30 different randomly selected model states and measured the standard deviation of the predicted residuals for all compounds in the test set. We observed only a few points with high variance (Extended Data Fig. 4c).

To maximize the model’s predictive capability on unseen data, we retrained it using the entire dataset, as is routinely done in cheminformatics studies^82–84^. This ‘full’ version of the model is used in Fig. 3c and onwards. Pairwise Wilcoxon statistical test *P* values were computed to confirm statistical significance of ranking of permeability predictions of the chemical compounds studied in this work.

##### Error bars of ML predictions

To be trained, neural network models require a starting point that is randomly generated. This ‘starting point’ is the set of initial values of the parameters of the neural network. A model that is well suited to the data and trained appropriately will reach virtually the same result (parameter values, and thus output prediction) regardless of the value of the random starting point. We therefore analysed the robustness of our model by accounting for the effect of different ‘starting points’ on the model output. To make final predictions on the test compounds in the figure, we trained the ML model 50 times, inputting 50 different random ‘starting points’, which are called random seeds. The fact that all the models trained starting from different random seeds provided a very similar result validates the robustness of the model. We then performed the Wilcoxon rank-sum test to generate *P* values and define the significance of the predictions for different compounds.

##### Model interpretability using a gradient boosted approach

We performed an interpretability analysis to determine which features of the input data lead the model to make its predictions. A fundamental issue with the black box nature of deep-learning models such as the MPNN is that our embeddings do not have human-interpretable meanings. To overcome this problem, an extreme gradient boosting (XGBoost) regressor (v.2.1.3) was employed to model the relationship between interpretable molecular descriptors and the predictions made by the MycoPermeNet model. The molecular descriptors used here were the 25 descriptors collected from RDkit (RDkit v.2024.9.5) and the CDD visualization tool, where each descriptor is an interpretable entry (for example, molecular weight, lipophilicity of the compound and so on). Features were hand selected via scientific intuition for relevance to Mtb permeability, resulting in an input matrix of 1,556 × 25 for our analysis.

The XGBoost model was configured with a maximum tree depth of 3 and a regularization parameter of 5 to control model complexity and prevent overfitting. The model was trained on the cleaned training set of descriptors. The trained XGBoost model was then used to predict the outcomes on the full set of interpretable descriptors. These predictions were compared against the predictions made by the original complex MLP model. The performance of the XGBoost model was evaluated by calculating the MSE and the *R*^2^ score between the XGBoost predictions and the complex model’s predictions on the full set. These metrics provided insight into how well the simpler, interpretable model could replicate the behaviour of the complex model.

To interpret the predictions made by the XGBoost model, SHapley Additive exPlanations (SHAP, v.0.5.6) was used. SHAP is a method that assigns each feature an importance value based on its contribution to the model’s predictions using Shapley values from cooperative game theory. SHAP values were computed to quantify the contribution of each feature (interpretable descriptor) to the predictions made by the XGBoost model (Extended Data Fig. 4d,e).

### Statistical analyses

Statistical analyses were performed with the SciPy package in Python (v.1.13.1), the scikit-learn package for evaluation metric calculations (Spearman correlations, performance evaluation metrics, that is, RMSE, *R*^2^, mean absolute error) (v.1.3.2) and GraphPad Prism 10.

### Reporting summary

Further information on research design is available in the Nature Portfolio Reporting Summary linked to this article.

## Supplementary information


Supplementary InformationChemical syntheses.
Reporting Summary
Peer Review File


## Source data


Source Data Figs. 1 and 4–6Statistical source data. Source Data Extended Data Fig. 2 Statistical source data. Source Data Extended Data Fig. 6 Mass spectrometry raw data.
Source Data Extended Data Fig. 7Statistical source data, mass spectrometry raw data. Source Data Extended Data Figs. 8 and 9 Statistical source data.


## Figures and Tables

**Extended Data Table 1:**
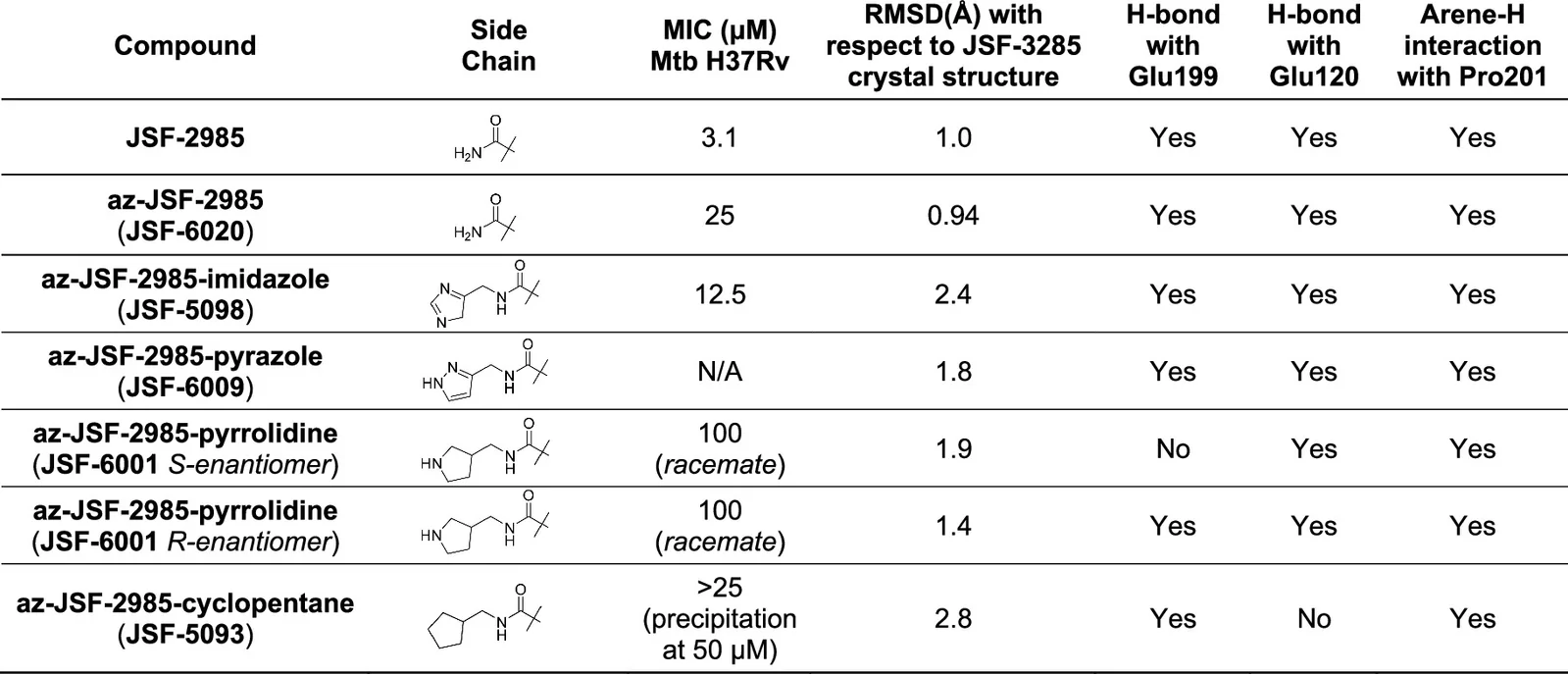
Parameters related to the docking studies of **JSF-2985** series with KasA and their antitubercular activity

Summary of docking studies features and activity against Mtb H37Rv of **JSF-2985** derivatives related to Extended Data Fig. 5. RMSD: root mean square deviation. MIC: minimum inhibitory concentration; ND: not determined.

## Data Availability

Data produced in this manuscript are available as Supplementary Information (Source Data). Mass spectrometry data are available as Source data provided with this paper.

## Code availability

All code and saved model weights for the cheminformatics analyses and MycoPermeNet model are publicly available on GitHub at https://github.com/Nevbarunegbe/Mycomembrane-permeability-project (ref. ^85^).
