# Supplementary material for: Identification of chemical features for improved outer membrane permeation in mycobacteria using machine learning
Source: Nat Microbiol. 2026 Jun 30;11(9):2647–65. doi: 10.1038/s41564-026-02412-5 (PMC13524814; doi:10.1038/s41564-026-02412-5)
Supplement: Supplementary file 1 [file 41564_2026_2412_MOESM1_ESM.pdf]

nature microbiology

Supplementary information https://doi.org/10.1038/s41564-026-02412-5

Identification of chemical features for improved outer membrane permeation in mycobacteria using machine learning

In the format provided by the authors and unedited

Molecules synthesis and characterization

Solid-phase synthesis of W1-3 peptides

W1-3 peptides were prepared by standard Fmoc-based solid-phase chemistry using the rink amide resin. Briefly, to a 25 mL peptide synthesis vessel, appropriate amount of resin was added, followed by 20% piperidine in *N, N*-Dimethylformamide (DMF, 15 mL). This was followed by shaking at room temperature for 30 min. The resin was then washed with methanol (CH_3OH) and dichloromethane (DCM) three times. After the last wash, 4 eqiv. of amino acid was added along with 4 eqiv. of ethyl cyanohydroxyiminoacetate (Oxyma) and 4 eqiv. of *N, N'*-Diisopropylcarbodiimide (DIC). The resin was shaken at room temperature for 2 h, then washed with CH_3OH/DCM. The remainder of the amino acids were coupled in the same manner. Peptides were cleaved from the resin using a TFA/TIPS/H_2O mixture (95:2.5:2.5, v/v/v) with shaking at room temperature for 2 h unless otherwise mentioned. The solution was filtered and concentrated prior to precipitation by the addition of cold diethyl ether to yield crude peptide. Crude peptides were purified by reverse-phased preparative high-performance liquid chromatography (RP-HPLC) equipped with Waters 1525 with 2489 UV/Visible Detector on a Phenomenex Luna 10 µm C8(2) 100 Å (250 x 21.2 mm) column using gradient elution with H_2O/MeOH with 0.1% TFA at 10 mL/min. The HPLC fractions of the desired purified compounds were first concentrated under reduced pressure using a rotary evaporator. The final concentrated aqueous solutions were lyophilized to dryness using Labconco Freezone 4.5 L (-84°C) lyophilizer. The peptides were analyzed for purity using Phenomenex Luna 5 µm C8(2) with gradient elution in H_2O/MeCN with 0.1% TFA at 1 mL/min. Peptide identities were confirmed via high resolution electrospray ionization mass spectrometry (HRMS, ESI/MS). Analyses were obtained on an Agilent 6545B Q-TOF LC/MS equipped with 1260 infinity II LC system with auto sampler.

Characterization of W1-3 peptides

Chemical structure of W1

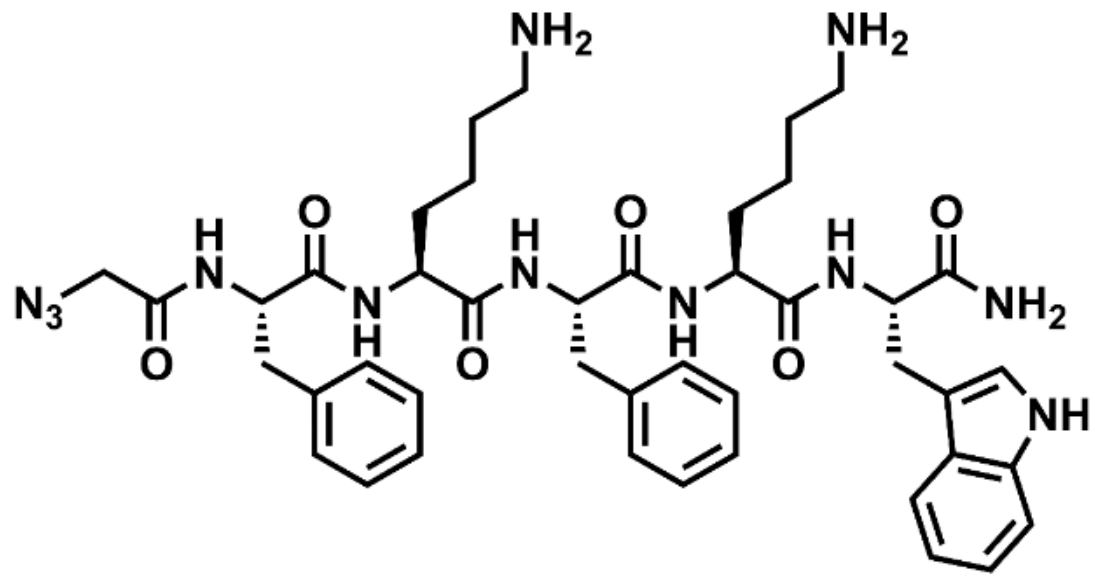

Analytical HPLC of W1

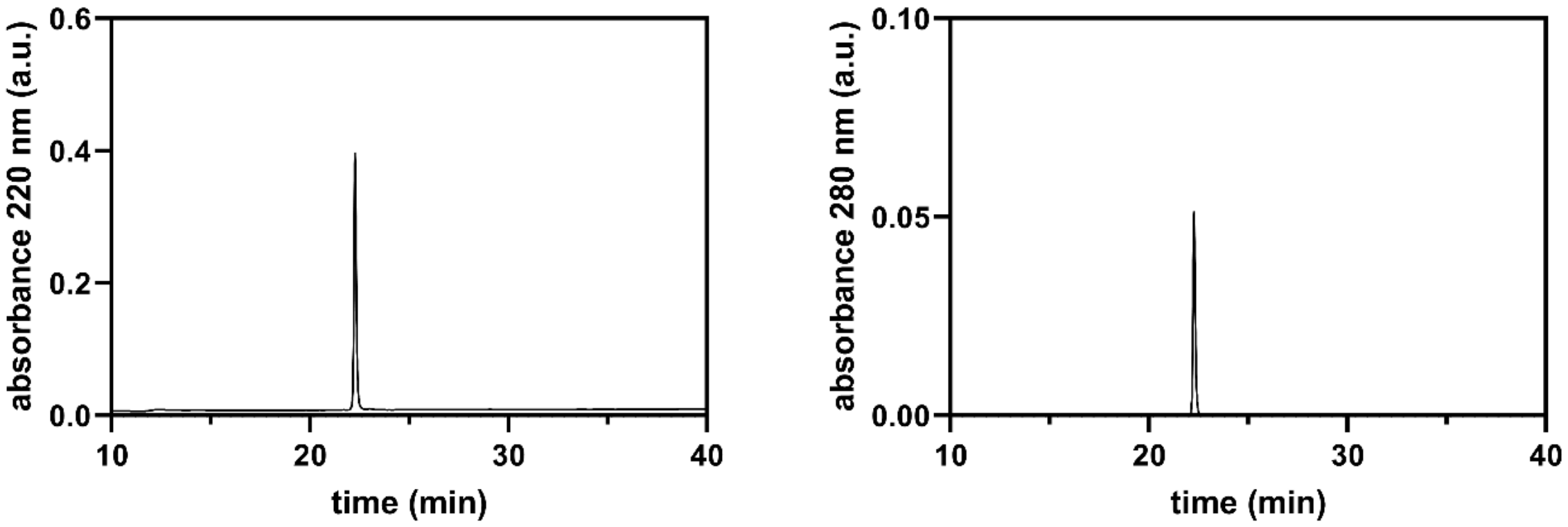

HRMS of W1

Expected m/z: $[M+H]^+$ 837.4519; found 837.4520

Chemical structure of W2

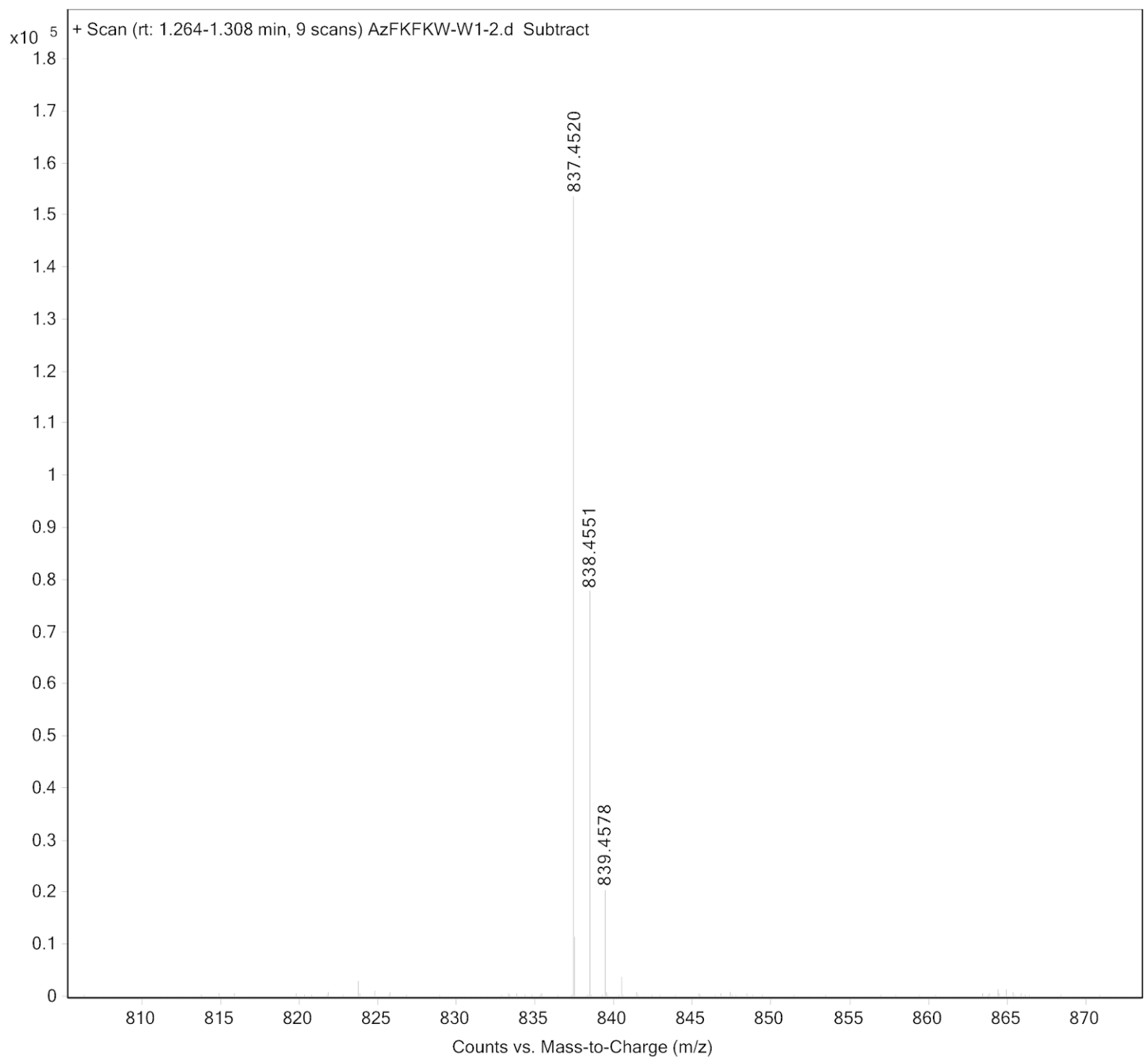

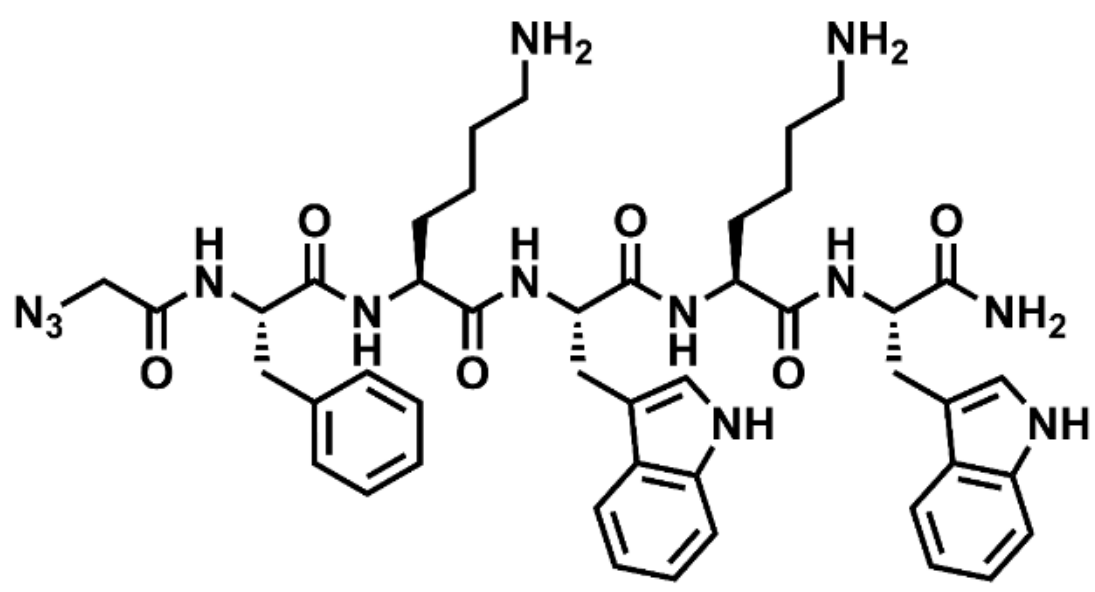

Analytical HPLC of W2

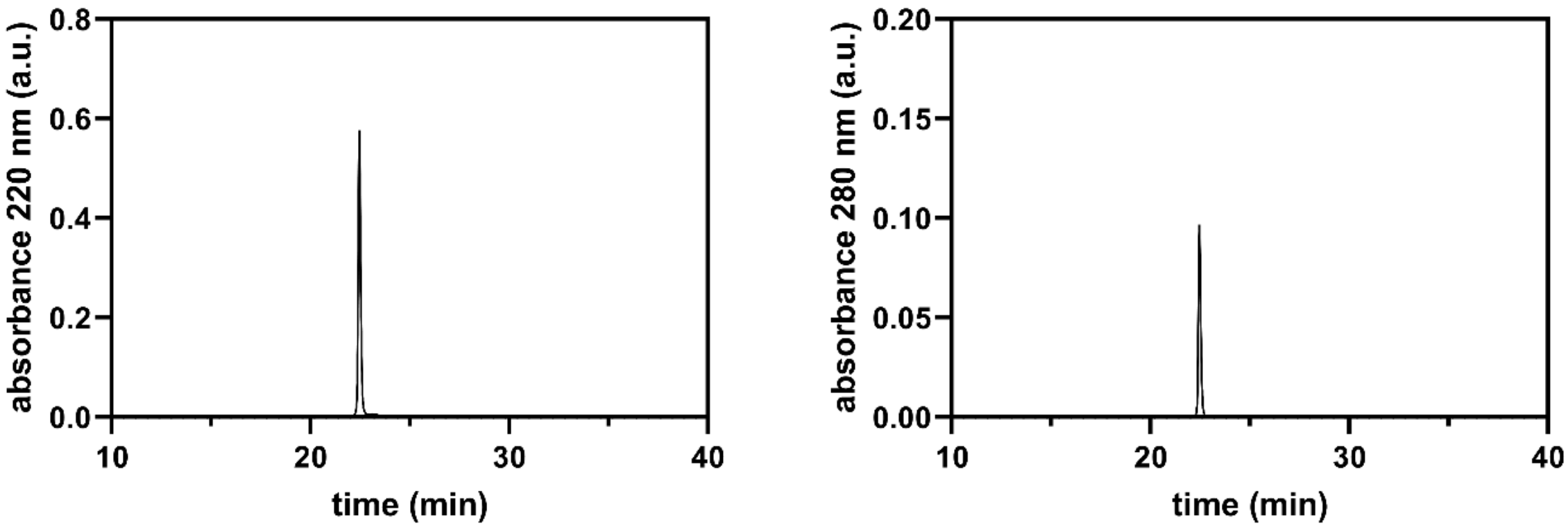

HRMS of W2

Expected m/z: $[M+H]^+$ 876.4628; found 876.4614

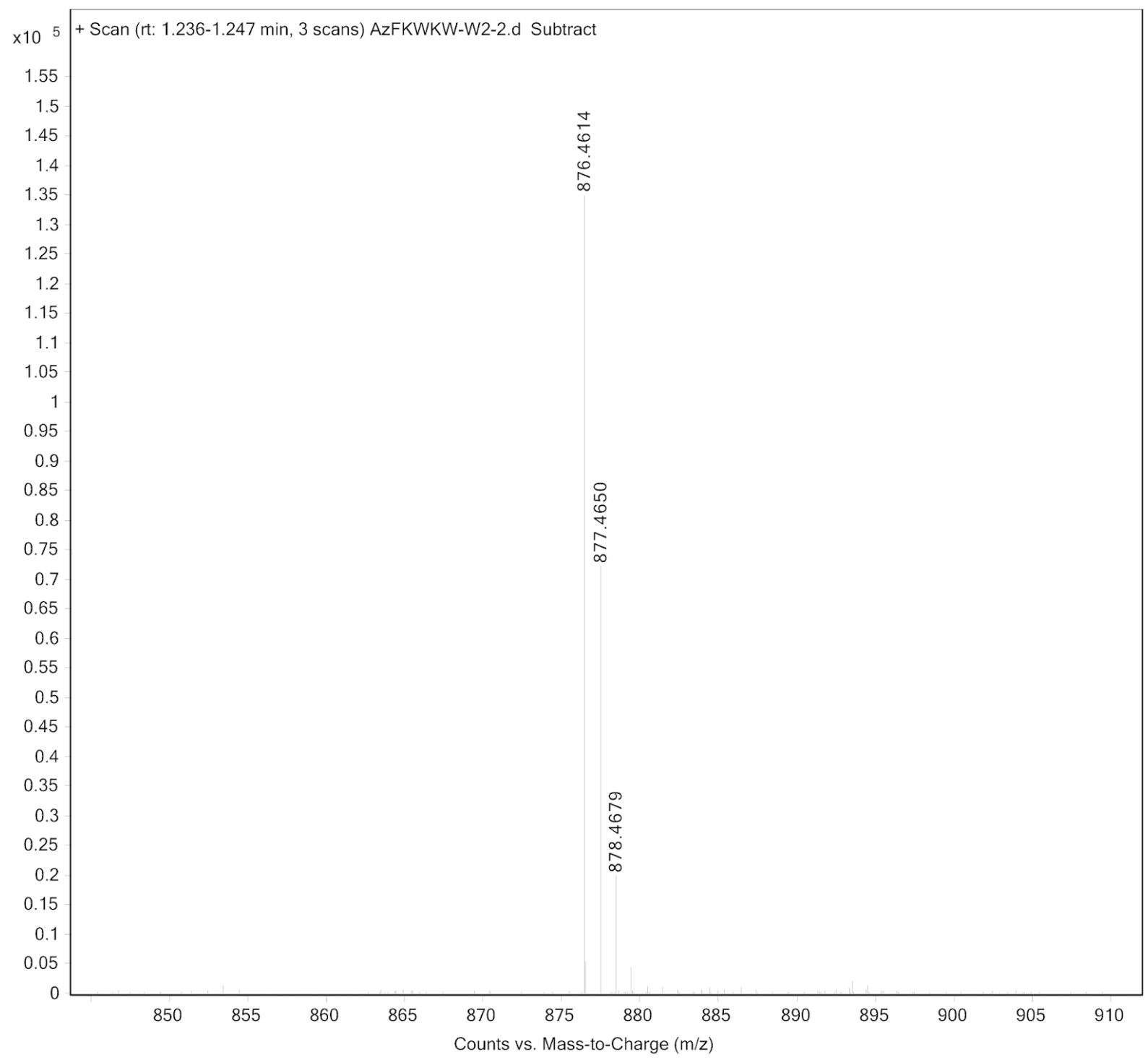

Chemical structure of W3

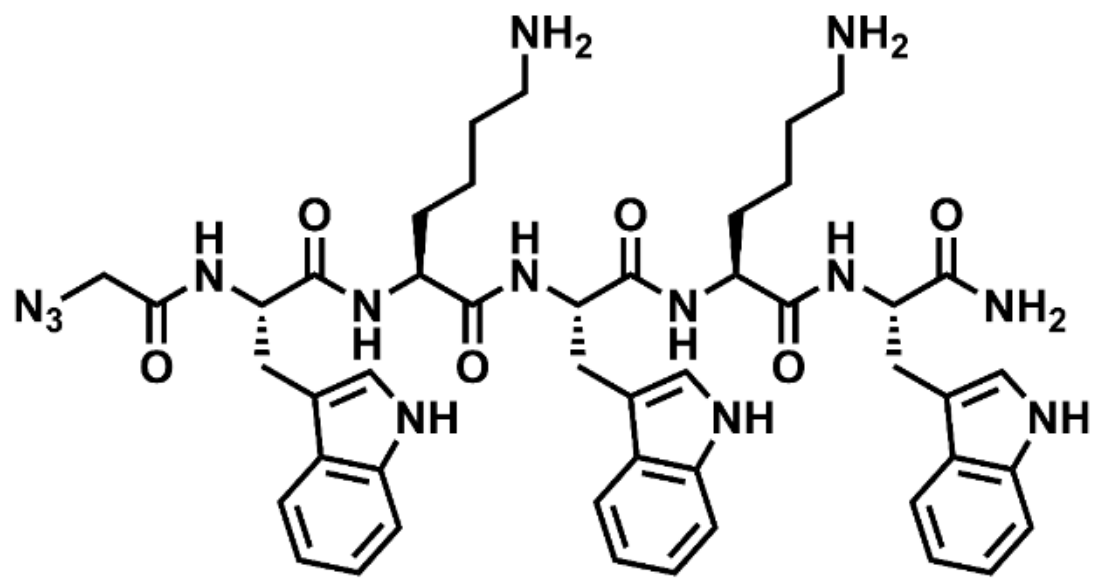

Analytical HPLC of W3

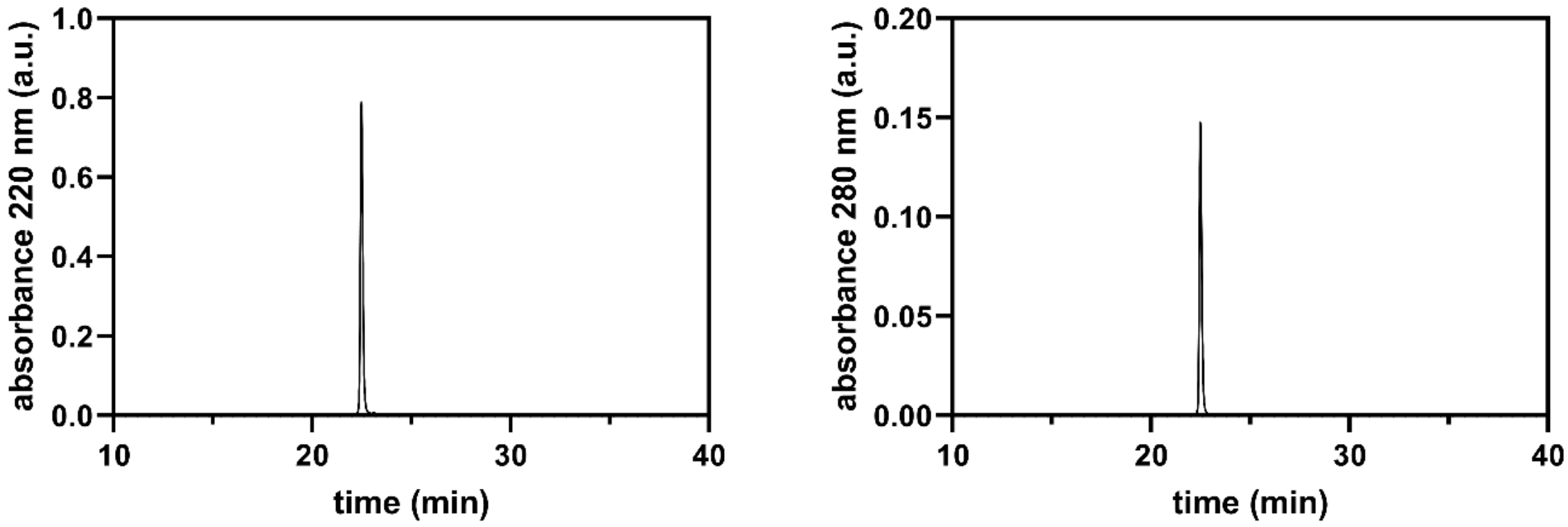

HRMS of W3

Expected m/z: $[M+H]^+$ 915.4737; found 915.4739

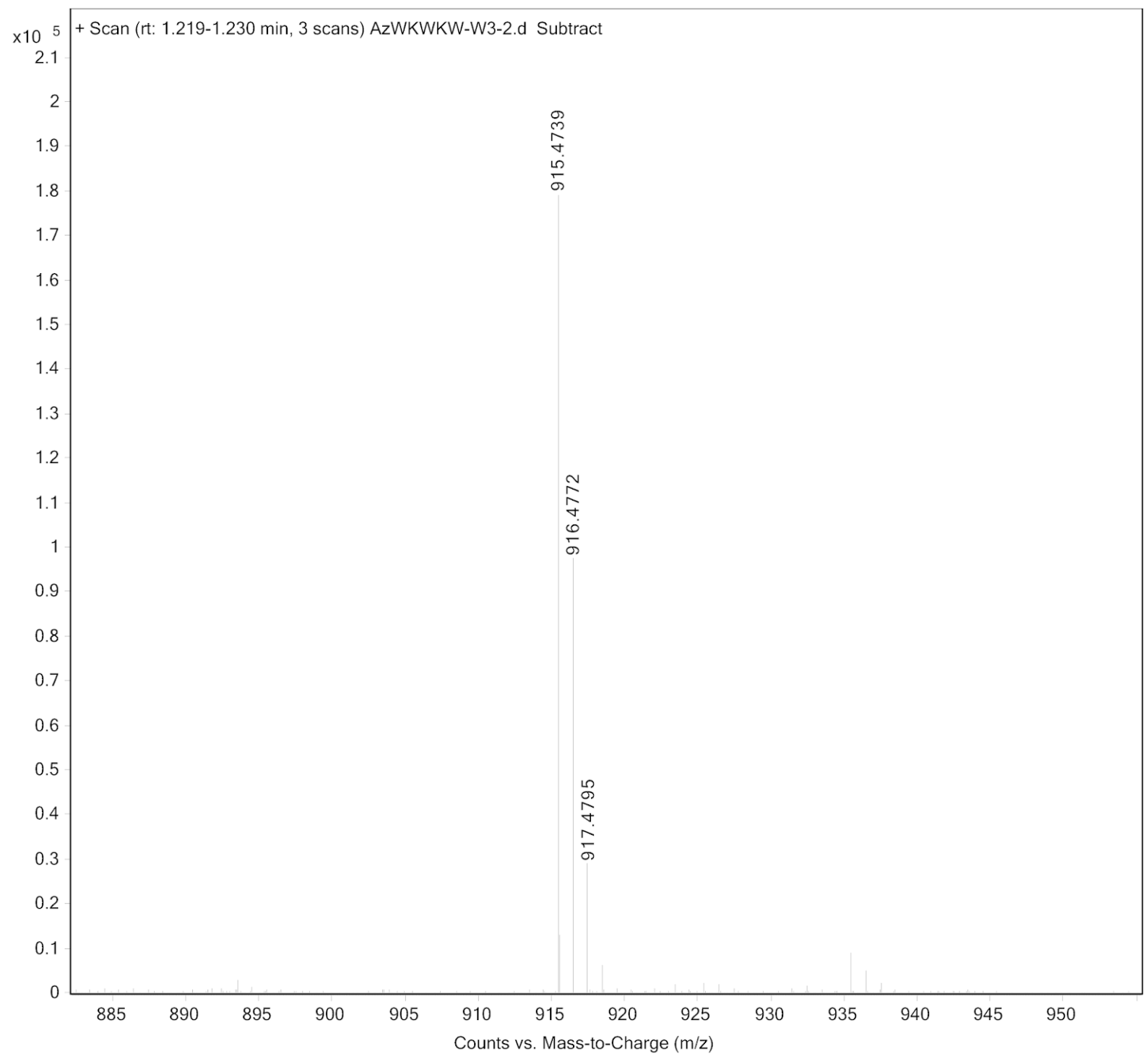

Solid-phase synthesis of W1-3 peptides

MATERIALS

REAGENTS	VENDOR SOURCE	CATALOG #
α-*N*-Fmoc-amino acids	Chem Impex	Various
2-Chlorotrityl chloride resin, (1.0 - 1.6 mmol/g, 100 - 200 mesh)	Chem Impex	03498
N, *N'*-Diisopropylethylamine (DIEA)	Chem Impex	00141
N, *N'*-Diisopropylcarbodiimide (DIC)	Chem Impex	00110
Ethyl Cyano(hydroxyimino)acetate (Oxyma)	TCI Chemicals	E0847
N, N-Dimethylformamide (DMF) ACS-grade, anhydrous	Millipore Sigma	319937, 589565
Dichloromethane (DCM) ACS-grade, anhydrous	Millipore Sigma	D65100, 270997
Methanol (CH_3OH) ACS-grade, HPLC-grade	Millipore Sigma	179337, 34860
Acetonitrile (CH_3CN) ACS-grade, HPLC-grade	Millipore Sigma	360457, 34851
Piperidine	Millipore Sigma	104094
Trifluoroacetic acid (TFA) HPLC grade	Millipore Sigma	302031
Trifluoroacetic acid (TFA) reagent-grade	Chem Impex	00289
Triisopropylsilane	Chem Impex	01966

METHODS

Tridecaptin peptides were prepared as described before[1] by standard Fmoc-based solid-phase chemistry using 2-chlorotrityl-chloride resin (0.142 mmol) such that the *C*-terminus would remain as a carboxylic acid upon cleavage. Briefly, a 25 mL peptide synthesis vessel was charged with 2-Chlorotrityl chloride resin, to which 1.1 equiv. of the first amino acid was added, along with 4.4 equiv. DIEA in 15 mL dry dichloromethane (DCM). The resin was agitated for 1 h at ambient temperature and washed with methanol (CH_3OH) and DCM (3 x 15 mL each). Deprotection of the Fmoc groups was carried out with 20% piperidine in *N*, *N*-Dimethylformamide (DMF, 15 mL) and shaking at room temperature for 30 min. The resin was then washed with CH_3OH and DCM three times. After the last wash, 3 eqiv. of amino acid was added along with 3 eqiv. of ethyl cyanohydroxyiminoacetate (Oxyma) and 3 eqiv. of *N*, *N'*-Diisopropylcarbodiimide (DIC). The resin was shaken at room temperature for 2 h, then washed with CH_3OH/DCM. The remainder of the amino acids were coupled in the same manner. Commercially available *N*-methyl amino acids were used accordingly for the *N*-methyl residues on the peptide. n-Octanoic acid was coupled on the *N*-terminus of the tridecaptin peptide derivatives on resin. Peptides were cleaved from the resin using a TFA/TIPS/H_2O mixture (95:2.5:2.5, v/v/v) with shaking at room temperature for 2 h unless otherwise mentioned. The solution was filtered and

concentrated prior to precipitation by the addition of cold diethyl ether to yield crude peptide. Crude peptides were purified by reverse-phased preparative high-performance liquid chromatography (RP-HPLC) equipped with Waters 1525 with 2489 UV/Visible Detector on a Phenomenex Luna 10 μm C8(2) 100 Å (250 x 21.2 mm) column using gradient elution with H2O/MeOH with 0.1% TFA at 10 mL/min. The HPLC fractions of the desired purified compounds were concentrated under reduced pressure using a rotary evaporator. The final concentrated aqueous solutions were lyophilized to dryness using Labconco Freezone 4.5 L (-84oC) yielding the purified compounds as solid powders. Peptide stock solutions were prepared by dissolving the lyophilized powders in either DMSO (**OctTriA1**, **OctTriA5**, **2N-Me-OctTriA1**, **2N-Me-OctTriA5**, **az2N-Me-OctTriA1**, **az2N-Me-OctTriA5**) or water (**azOctTriA1**, **azOctTriA5**), depending on peptide solubility. Owing to variability in the solubility profiles of azide-Tridecaptin peptides, initial stock solutions were prepared at low concentration in water when possible. Solubility was assessed by visual inspection, and additional dilution was performed to obtain a clear, homogeneous solution. Peptide stock solutions were passed through a 0.22 μm PTFE or PVDF syringe filter to remove insoluble particulates and ensure compatibility with downstream analytical methods. Final concentration of peptide stocks were determined using UV-Vis absorbance of the tryptophan residues in their sequence at 280 nm (ε = 5690 cm-1M-1).The peptides were analyzed for purity using Phenomenex Luna 5 μm C8(2) with gradient elution in H_2O/MeCN with 0.1% TFA at 1 mL/min. Peptide identities were confirmed via high resolution electrospray ionization mass spectrometry (HRMS, ESI/MS). Analyses were obtained on an Agilent 6545B Q-TOF LC/MS equipped with 1260 infinity II LC system with auto sampler. Samples were dissolved in CH_3CN and eluted with a CH_3CN/H_2O solution containing 0.1% formic acid. Characterization data are provided below.

OctTriA1

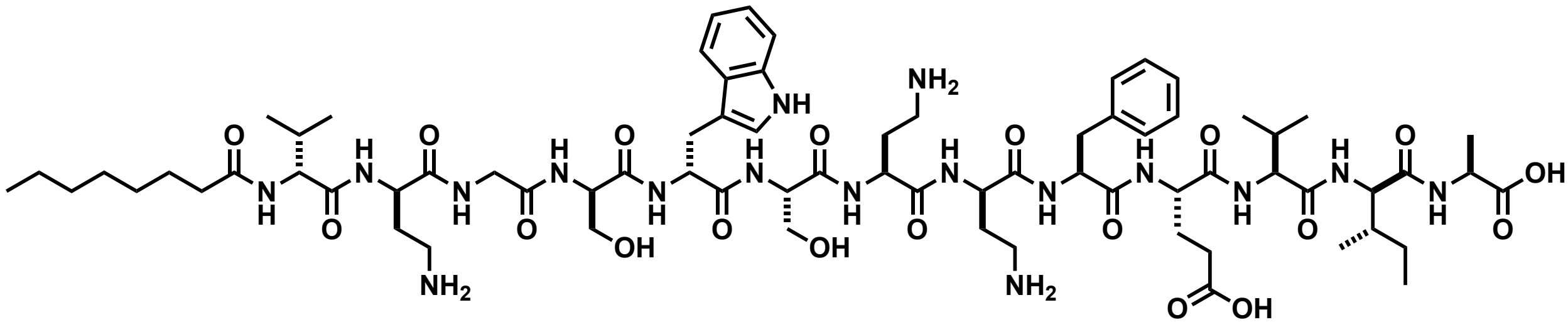

Chemical structure of **OctTriA1**.

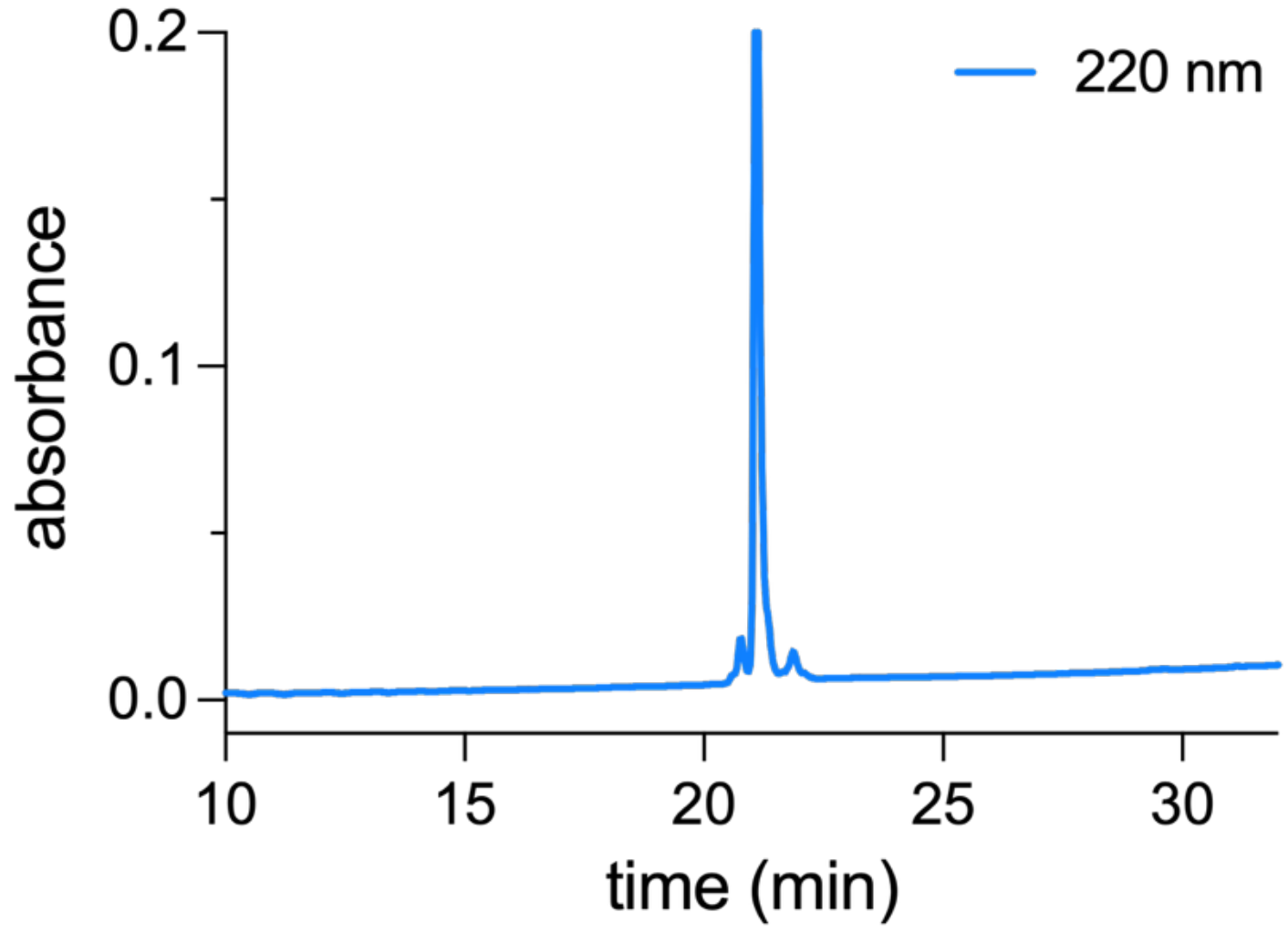

Analytical HPLC chromatogram of **OctTriA1**.

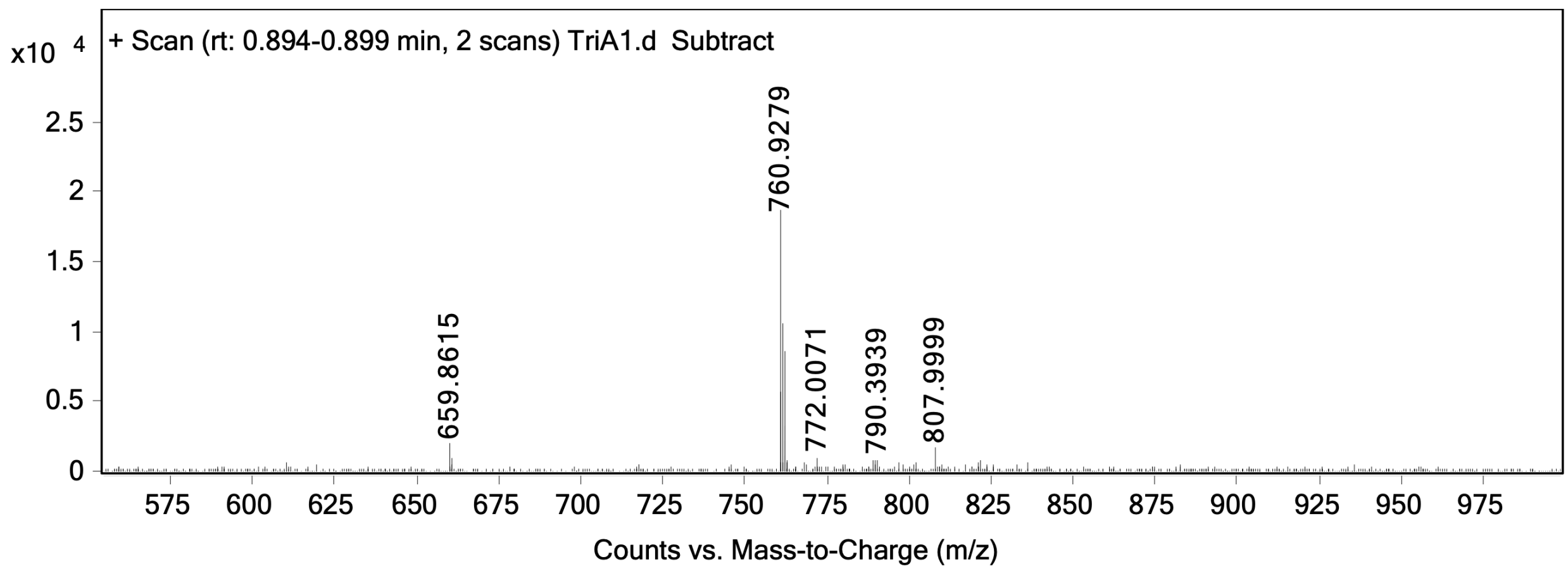

QTOF high resolution mass spectrum of **OctTriA1**(expected m/z 760.9272 for $[M+2H]^{2+}$).

azOctTriA1

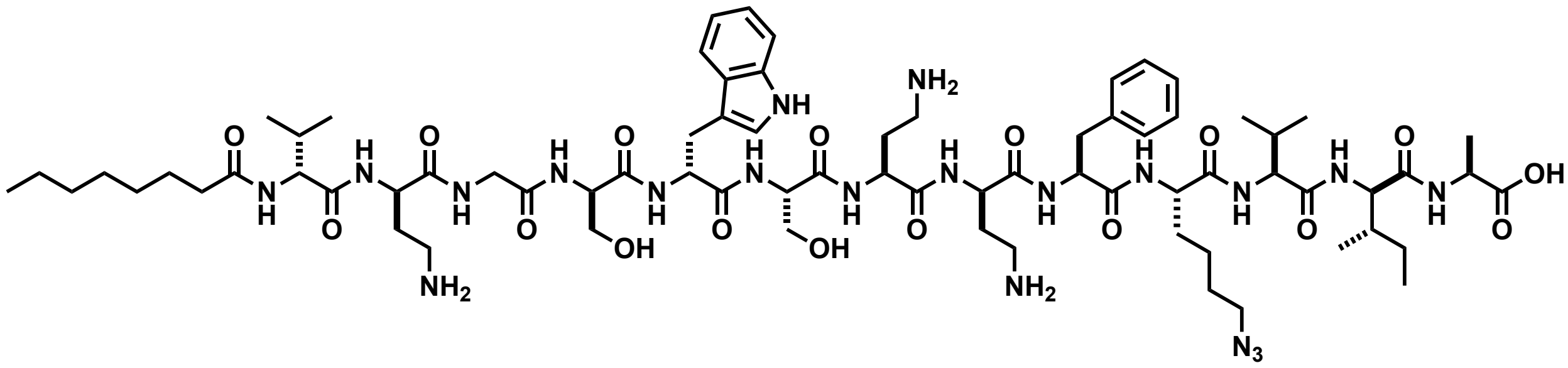

Chemical structure of **azOctTriA1**.

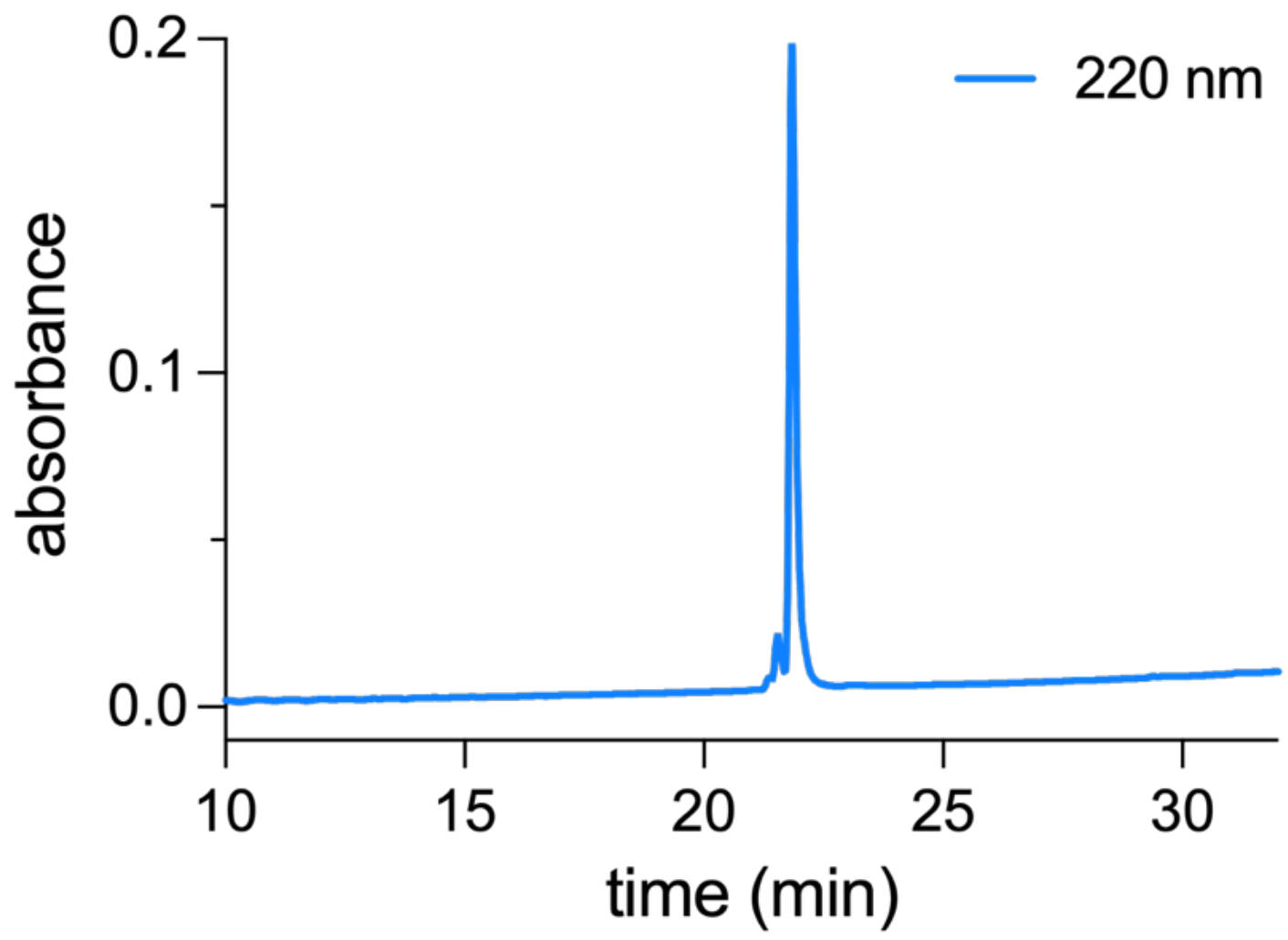

Analytical HPLC chromatogram of **azOctTriA1**.

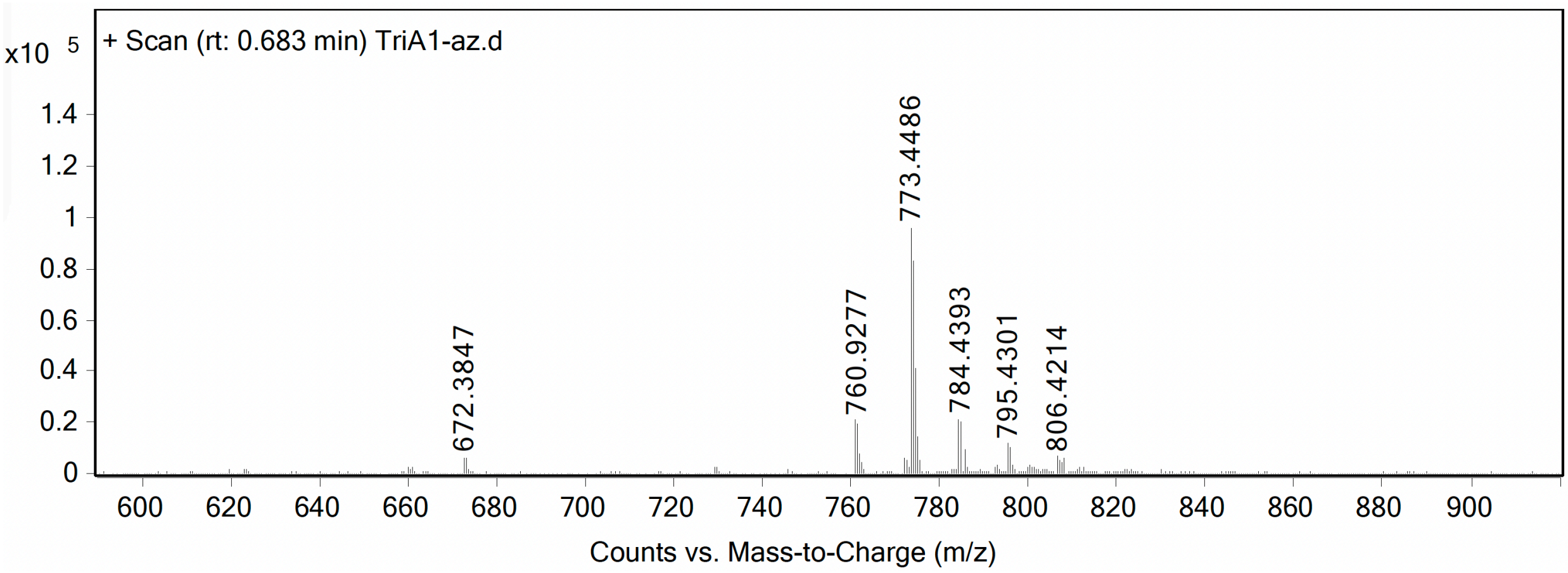

QTOF high resolution mass spectrum of **azOctTriA1**(expected m/z 773.4487 for $[M+2H]^{2+}$).

2N-Me-OctTriA1

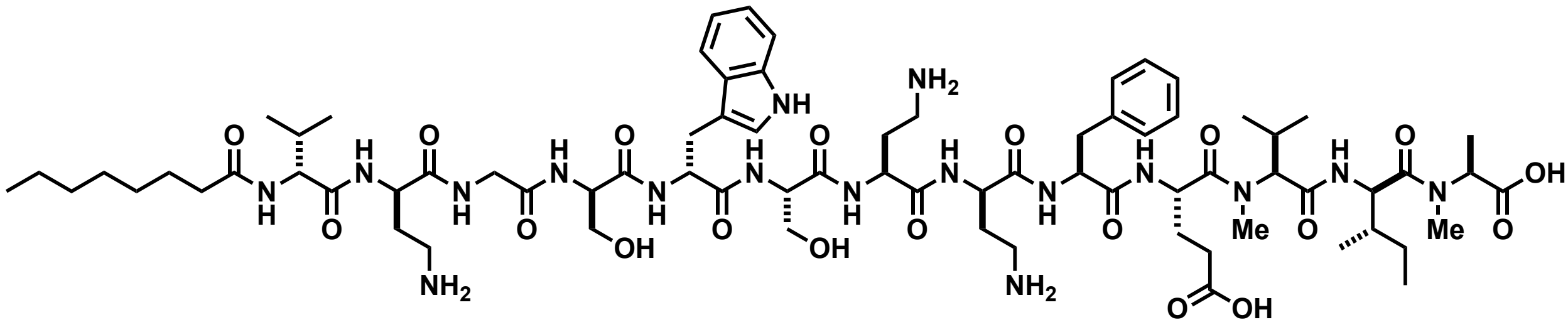

Chemical structure of **2N-Me-OctTriA1**.

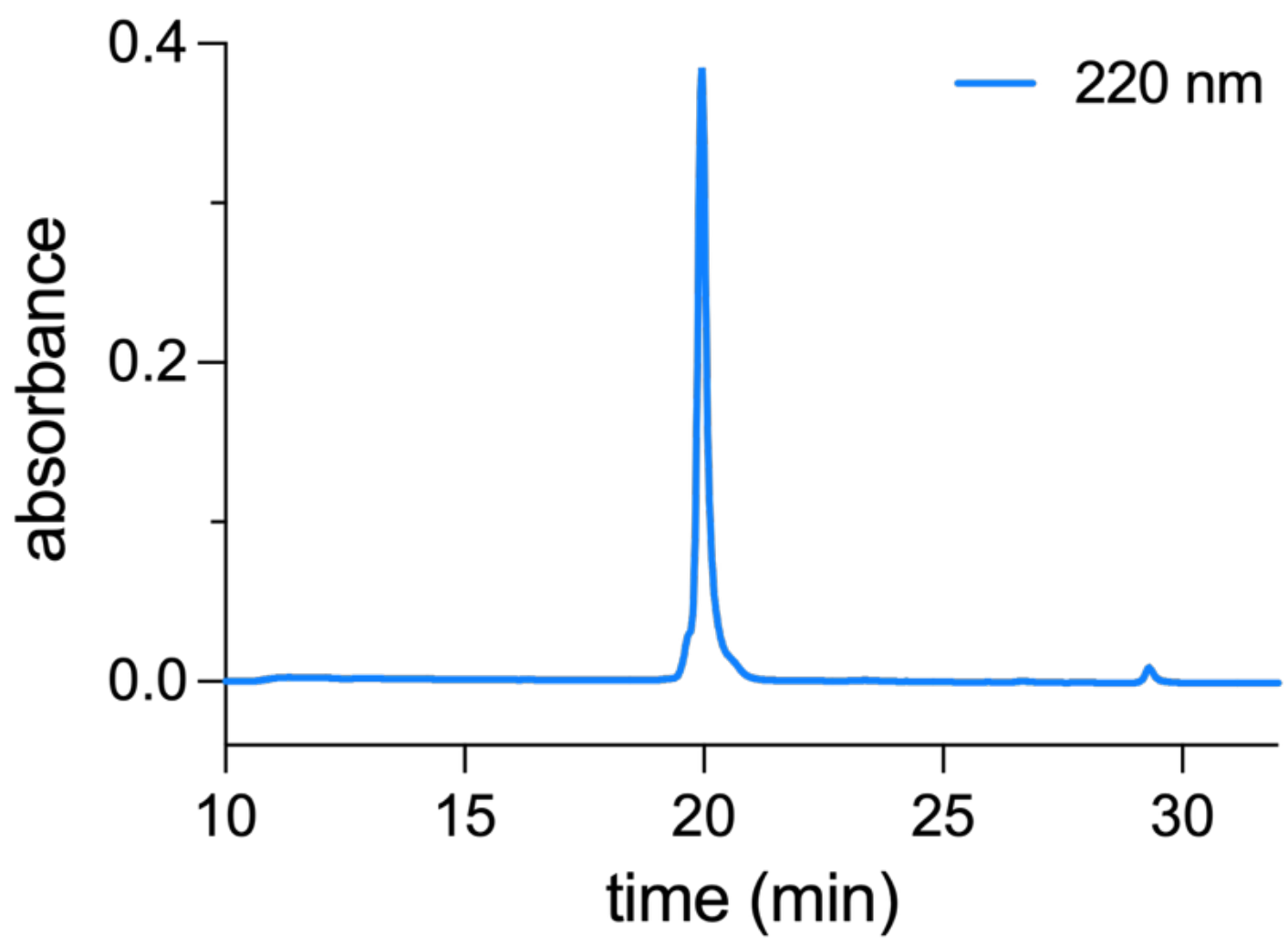

Analytical HPLC chromatogram of **2N-Me-OctTriA1**.

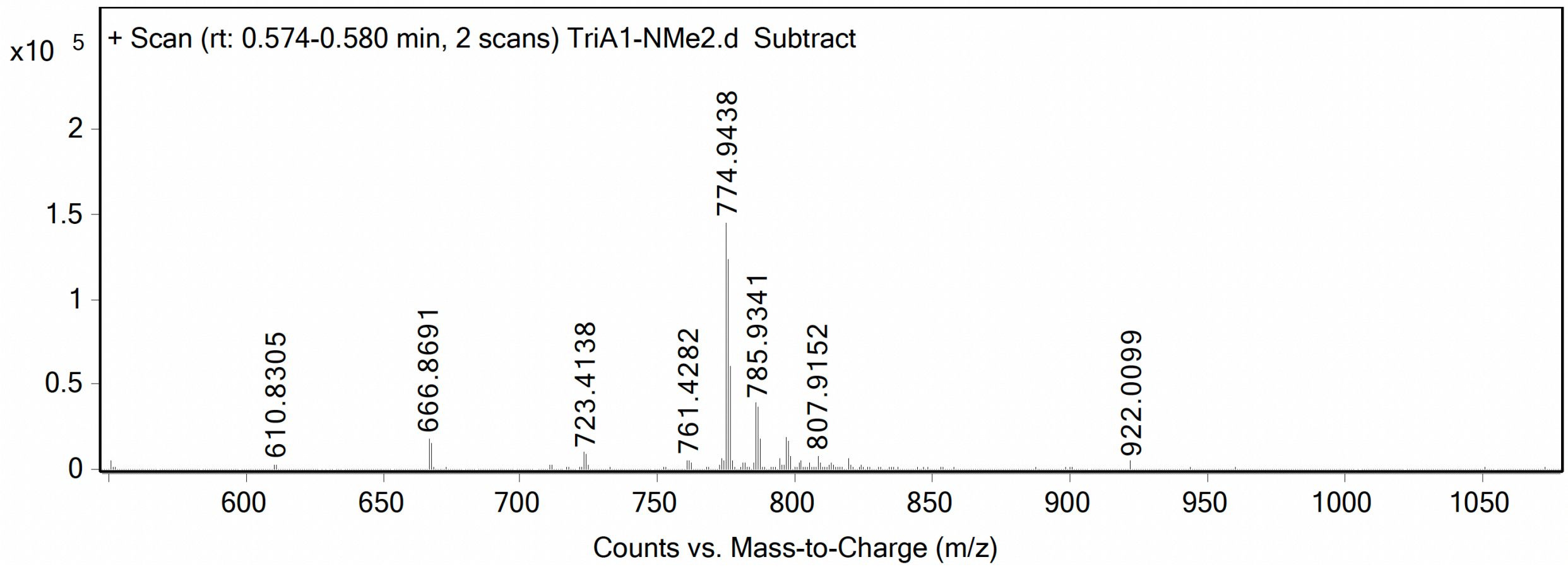

QTOF high resolution mass spectrum of **2N-Me-OctTriA1** (expected m/z 774.9429 for $[M+2H]^{2+}$).

az2N-Me-OctTriA1

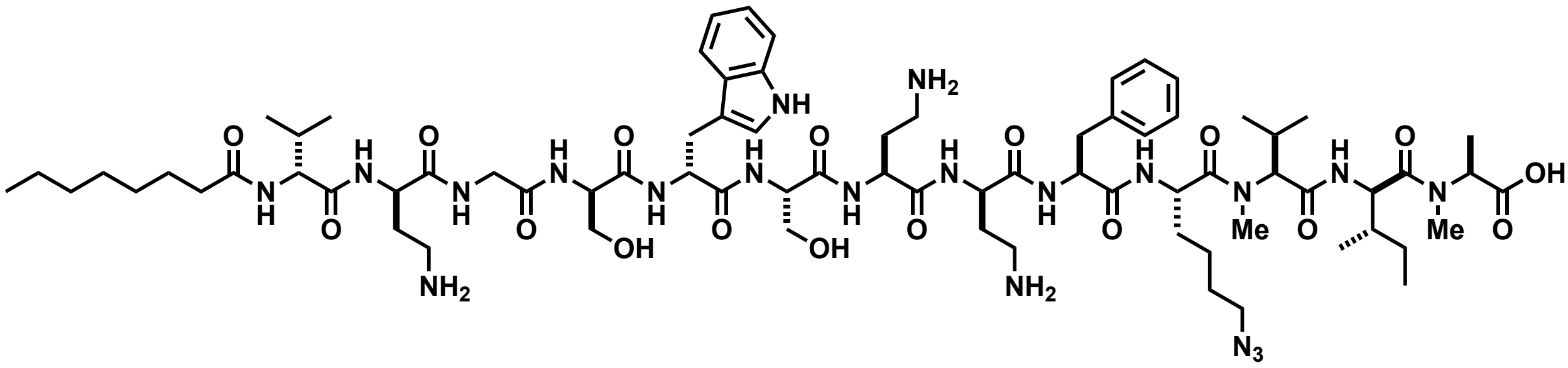

Chemical structure of **az2N-Me-OctTriA1**.

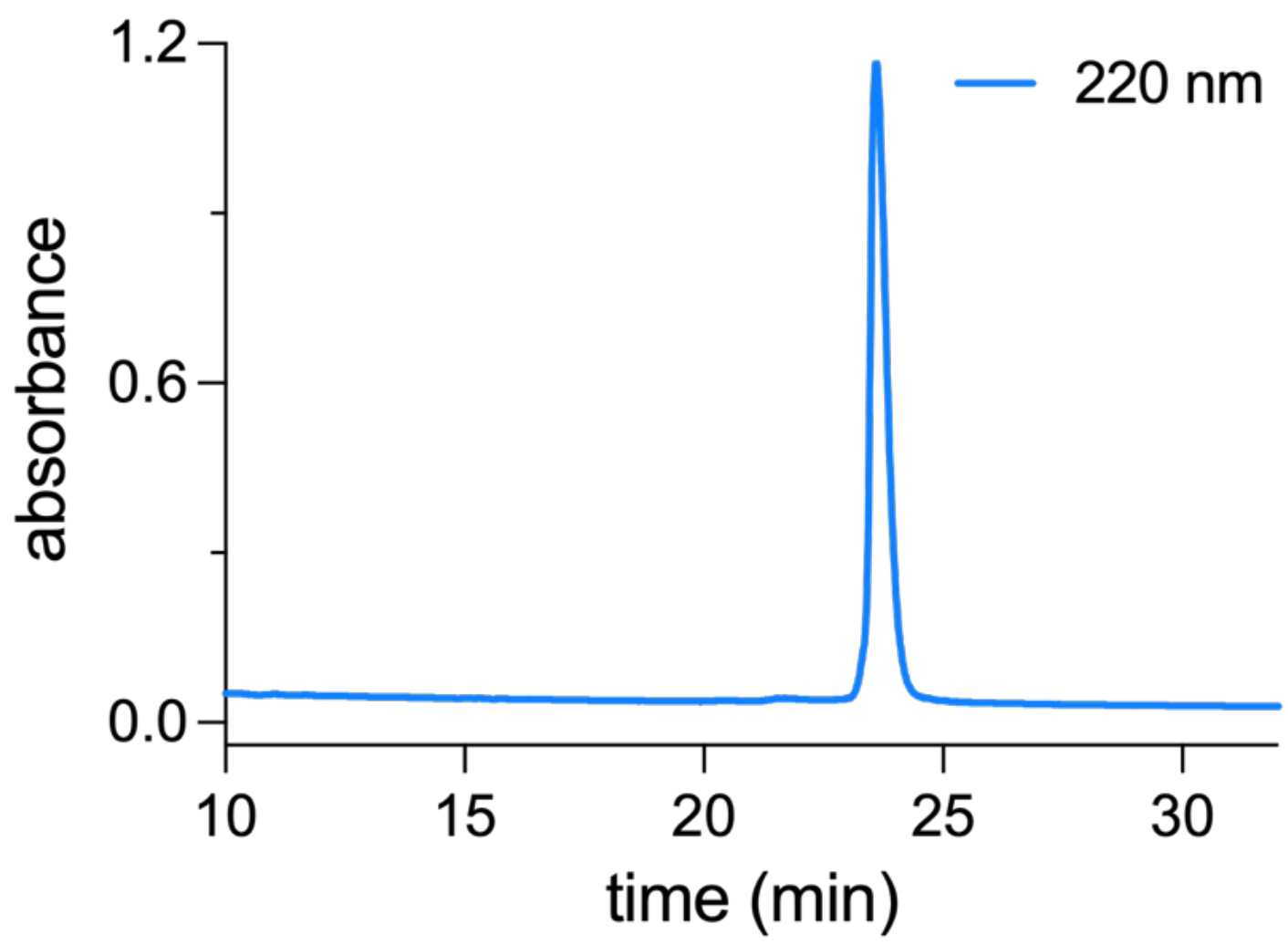

Analytical HPLC chromatogram of **az2N-Me-OctTriA1**.

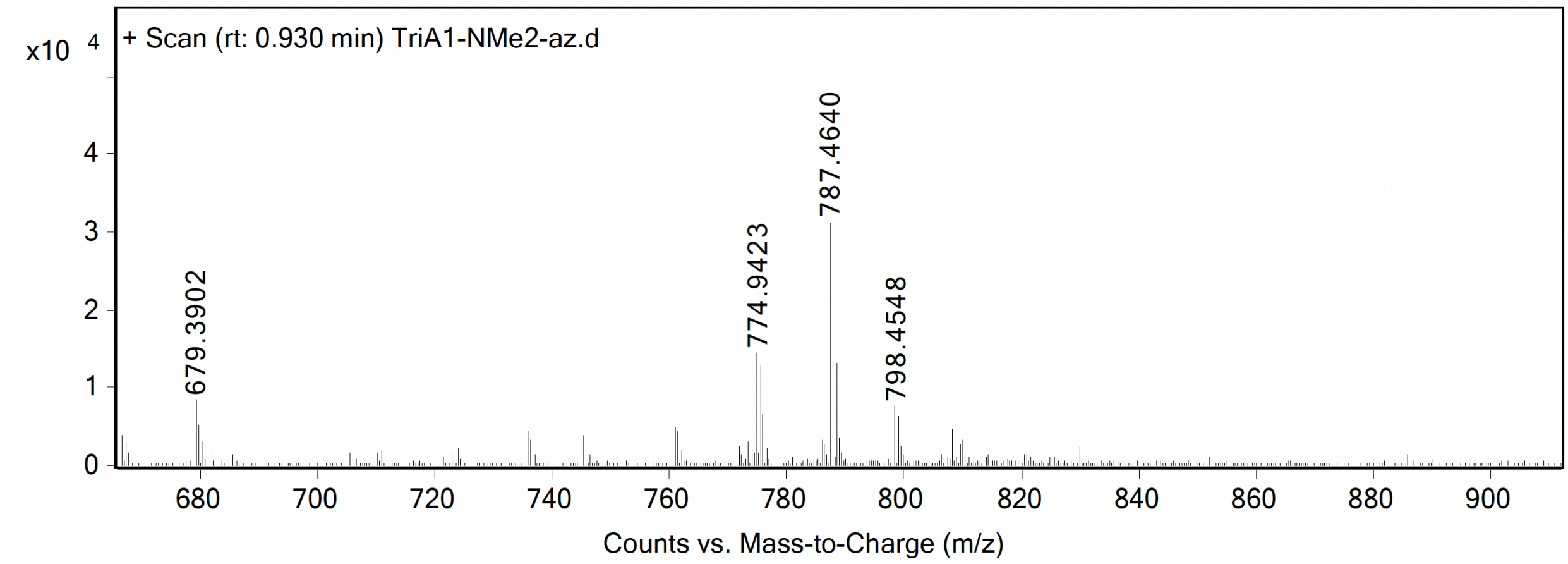

QTOF high resolution mass spectrum of **az2N-Me-OctTriA1** (expected m/z 787.4643 for $[M+2H]^{2+}$).

OctTriA5

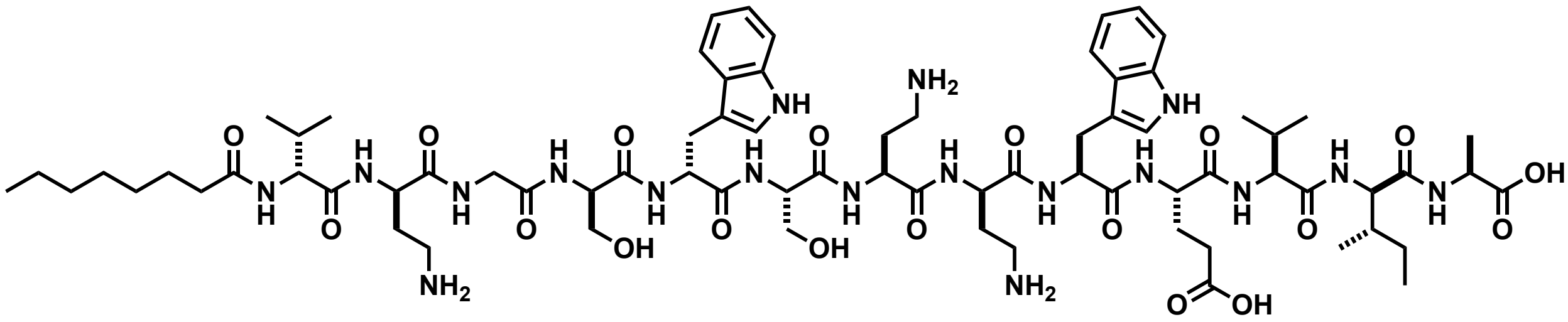

Chemical structure of **OctTriA5**.

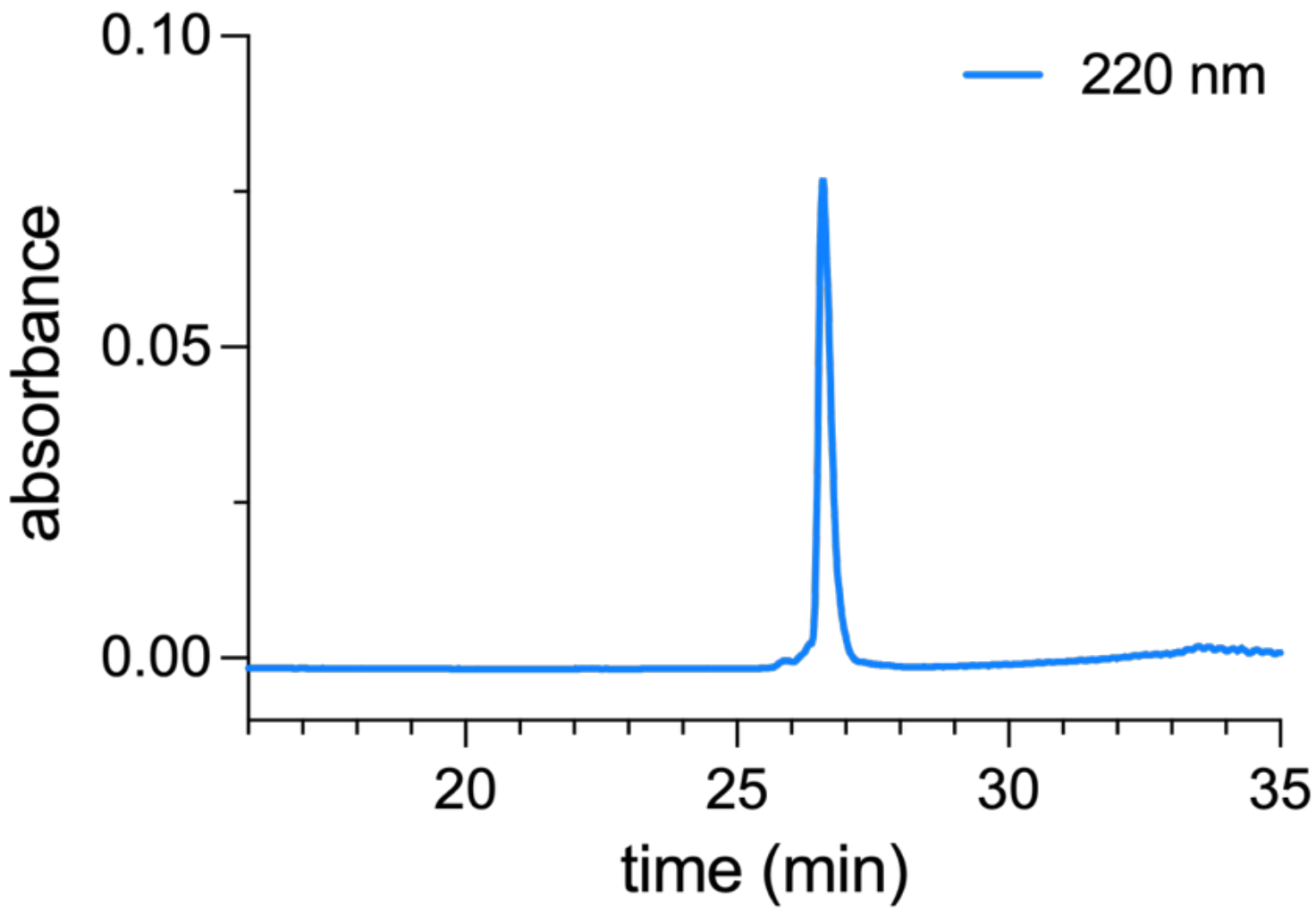

Analytical HPLC chromatogram of **OctTriA5**.

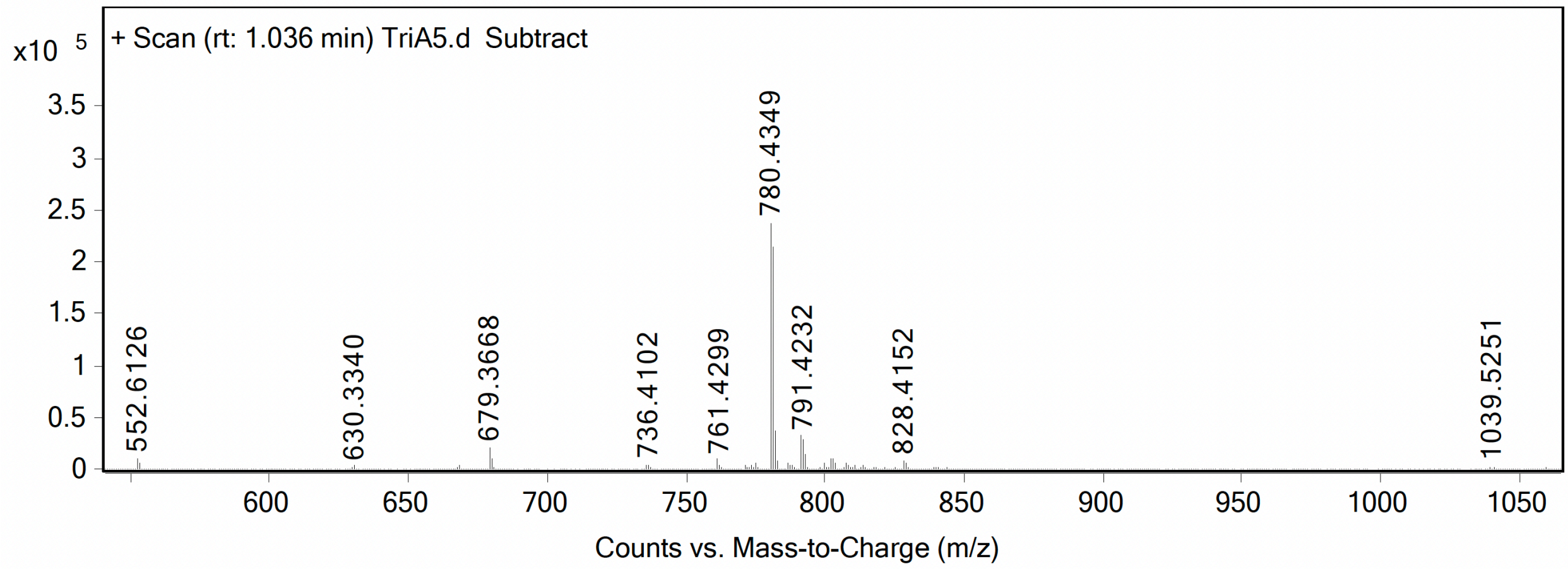

QTOF high resolution mass spectrum of **OctTriA5** (expected m/z 780.4327 for $[M+2H]^{2+}$).

azOctTriA5

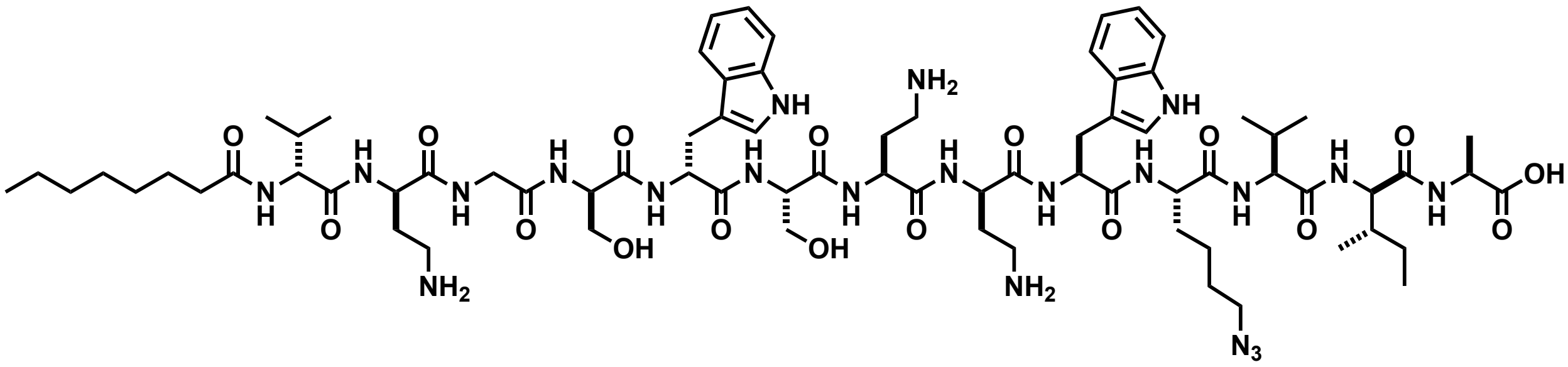

Chemical structure of **azOctTriA5**.

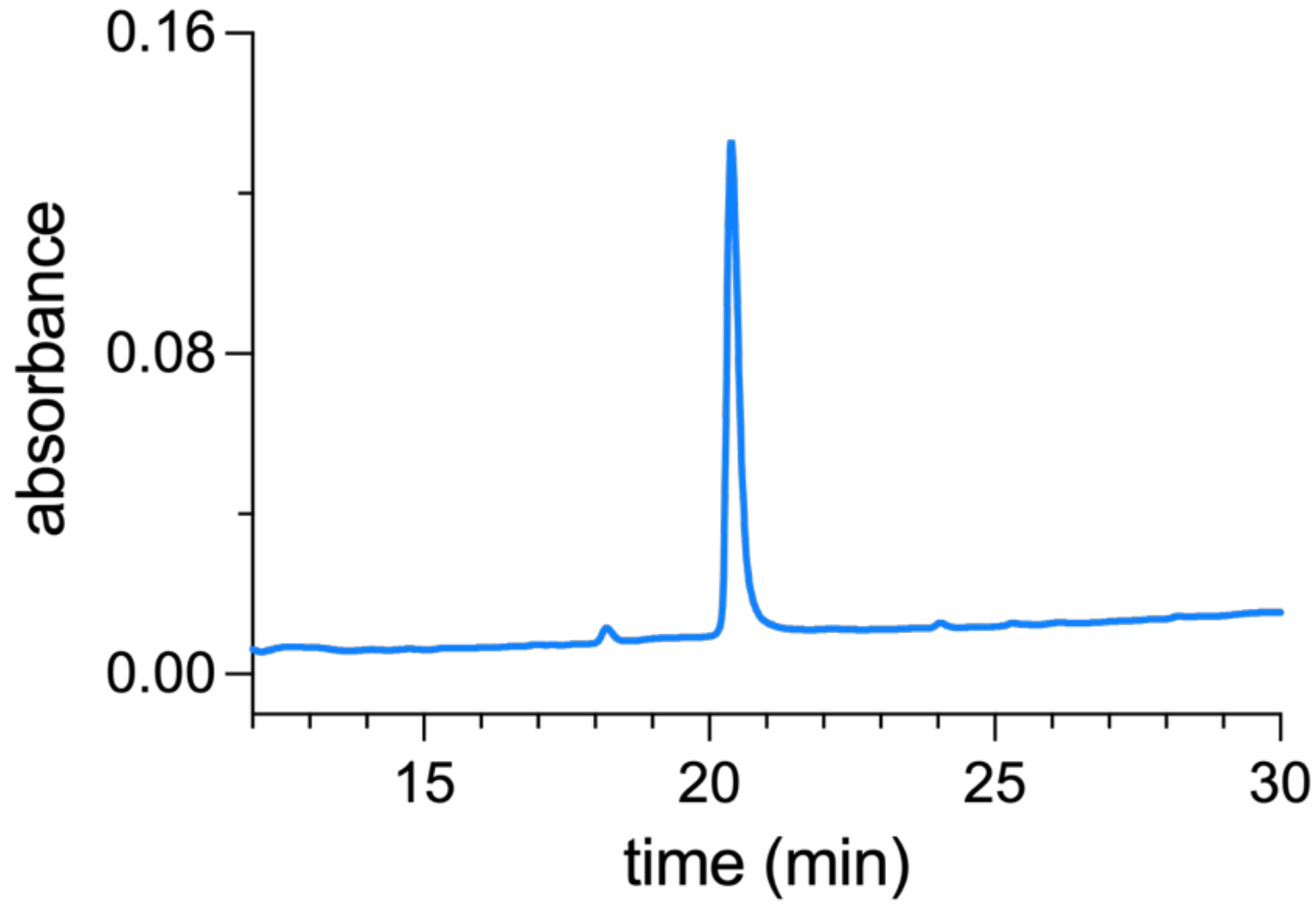

Analytical HPLC chromatogram of **azOctTriA5**.

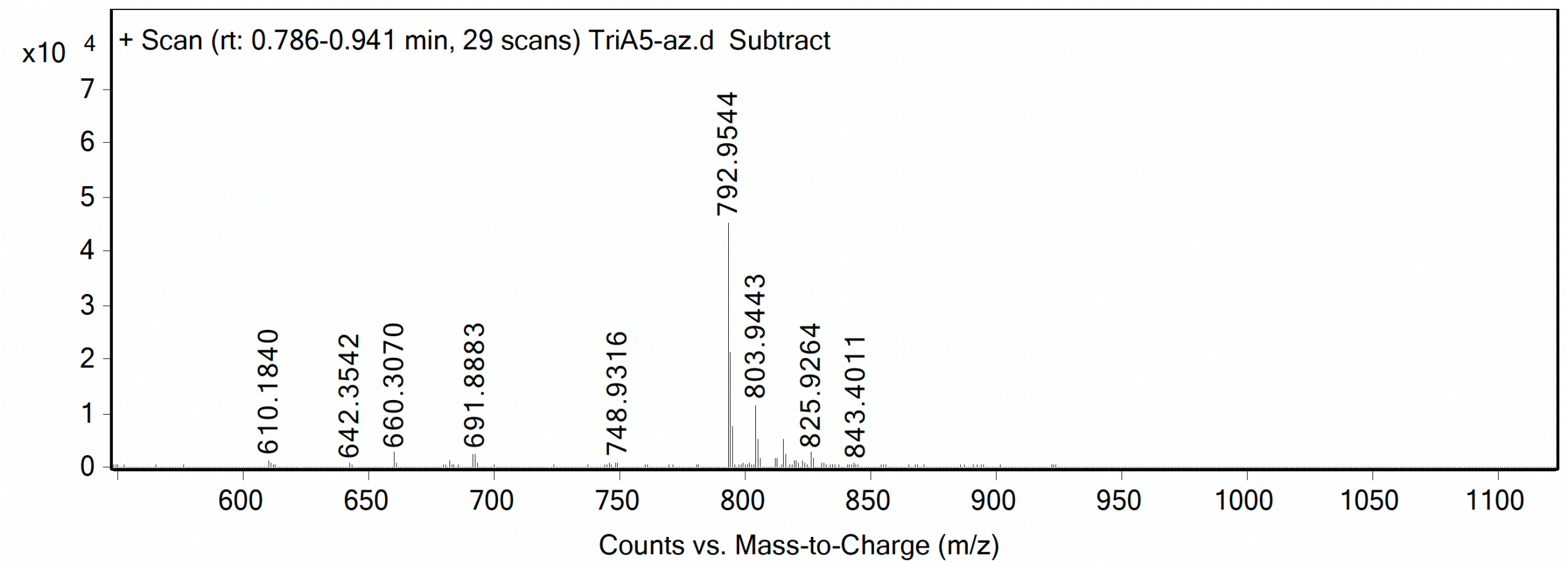

QTOF high resolution mass spectrum of **azOctTriA5** (expected m/z 792.9541 for $[M+2H]^{2+}$).

2N-Me-OctTriA5

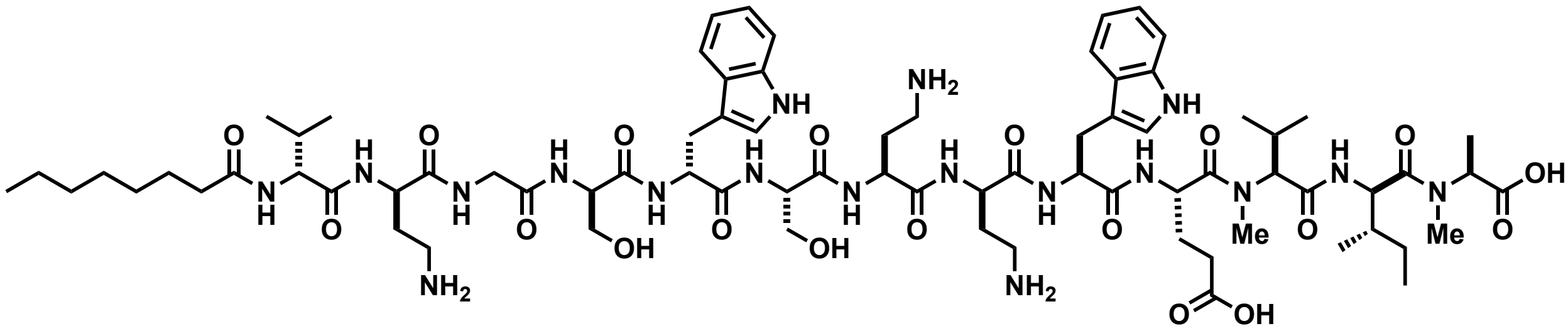

Chemical structure of **2N-Me-OctTriA5**.

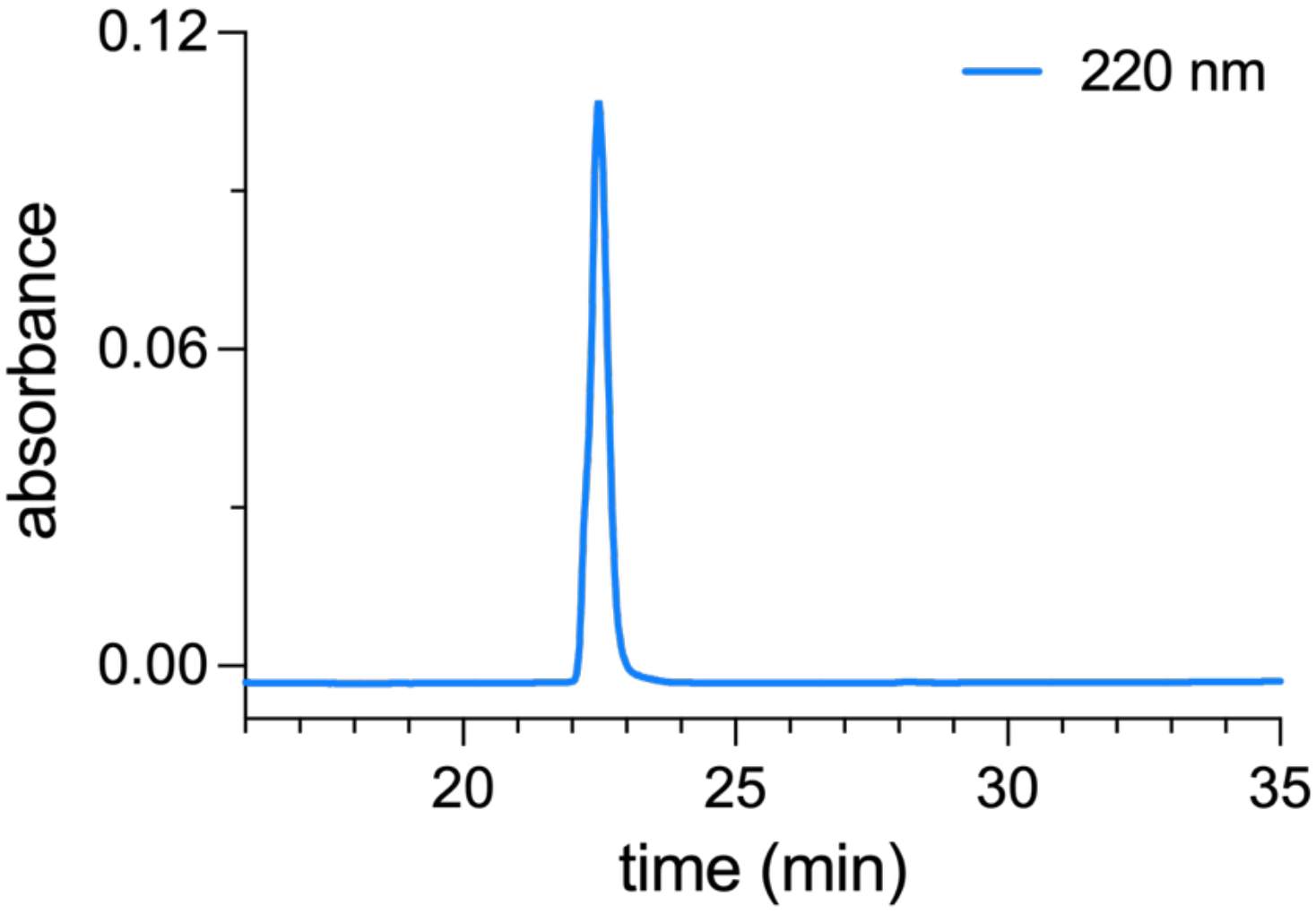

Analytical HPLC chromatogram of **2N-Me-OctTriA5**.

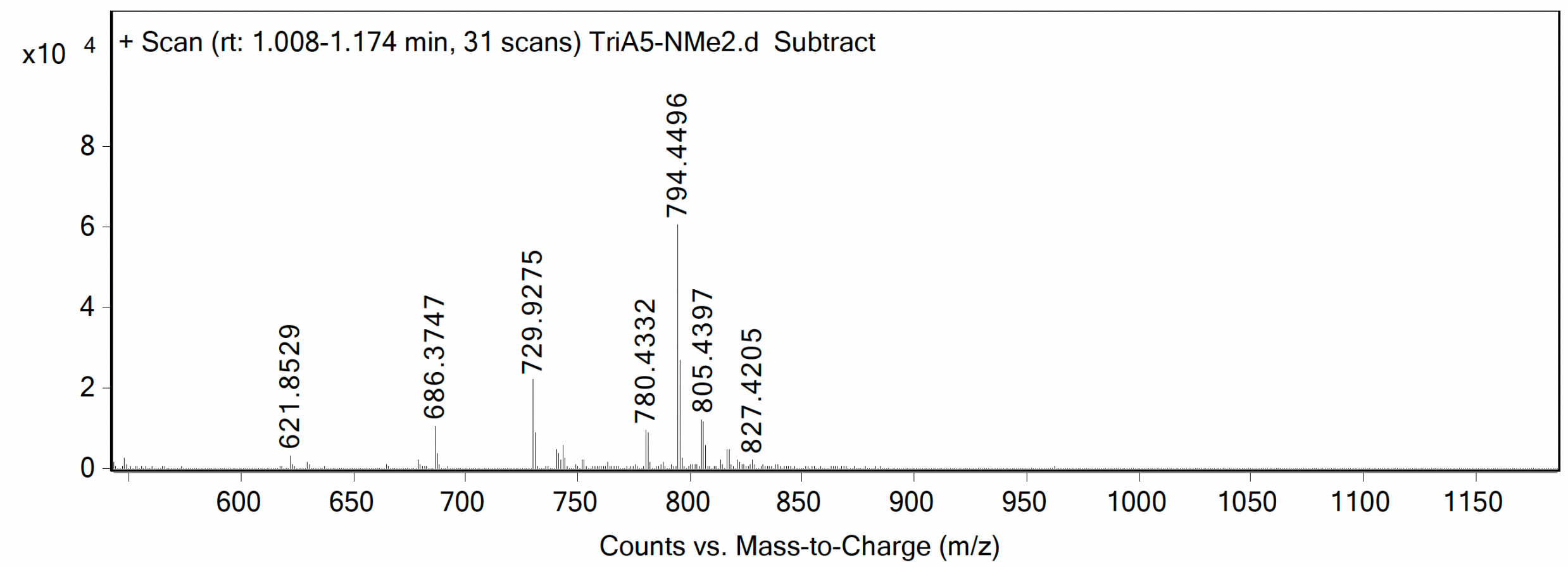

QTOF high resolution mass spectrum of **2N-Me-OctTriA5**. (expected m/z 794.4483 for $[M+2H]^{2+}$).

az2N-Me-OctTriA5

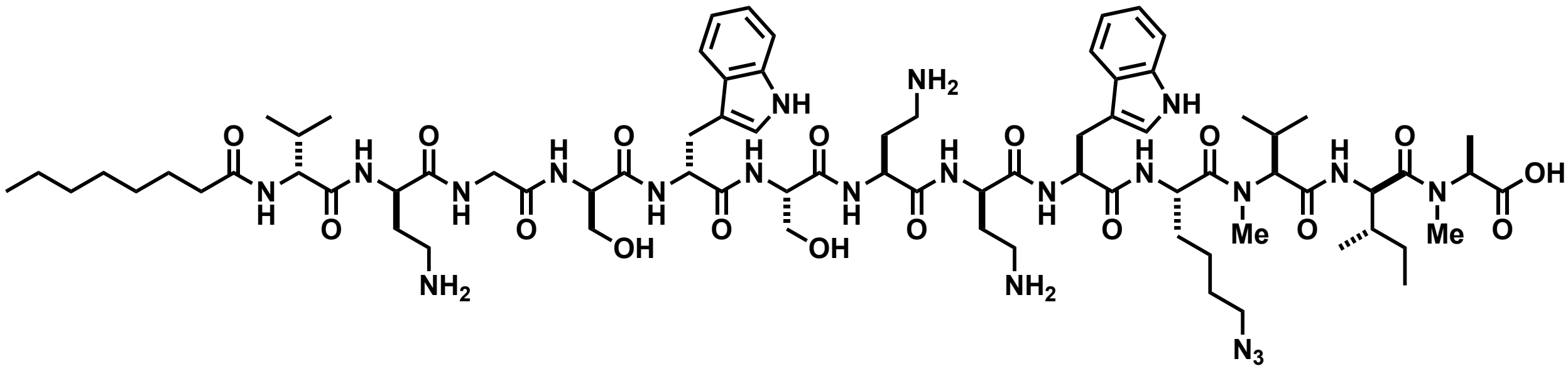

Chemical structure of **az2N-Me-OctTriA5**.

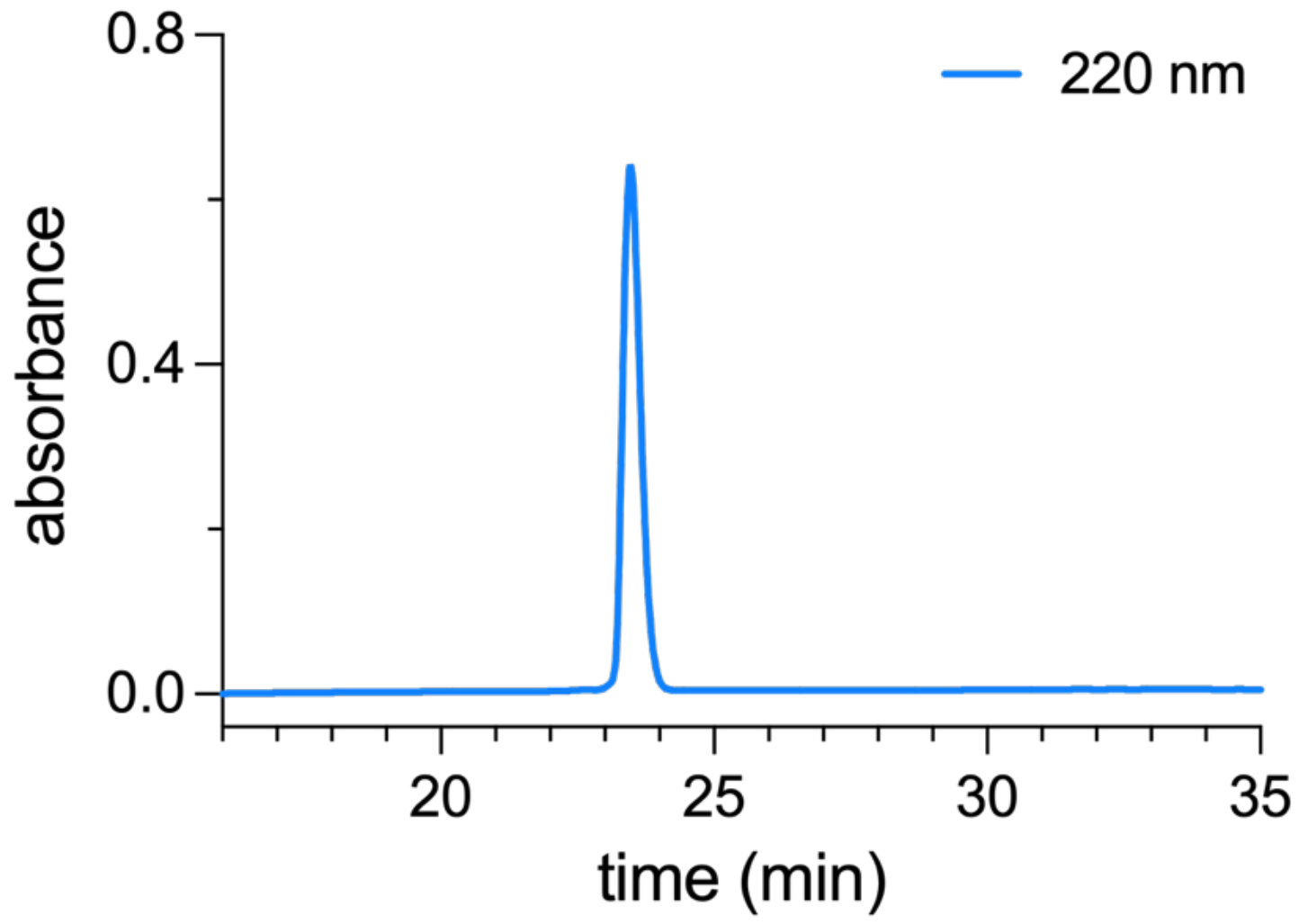

Analytical HPLC chromatogram of **az2N-Me-OctTriA5**.

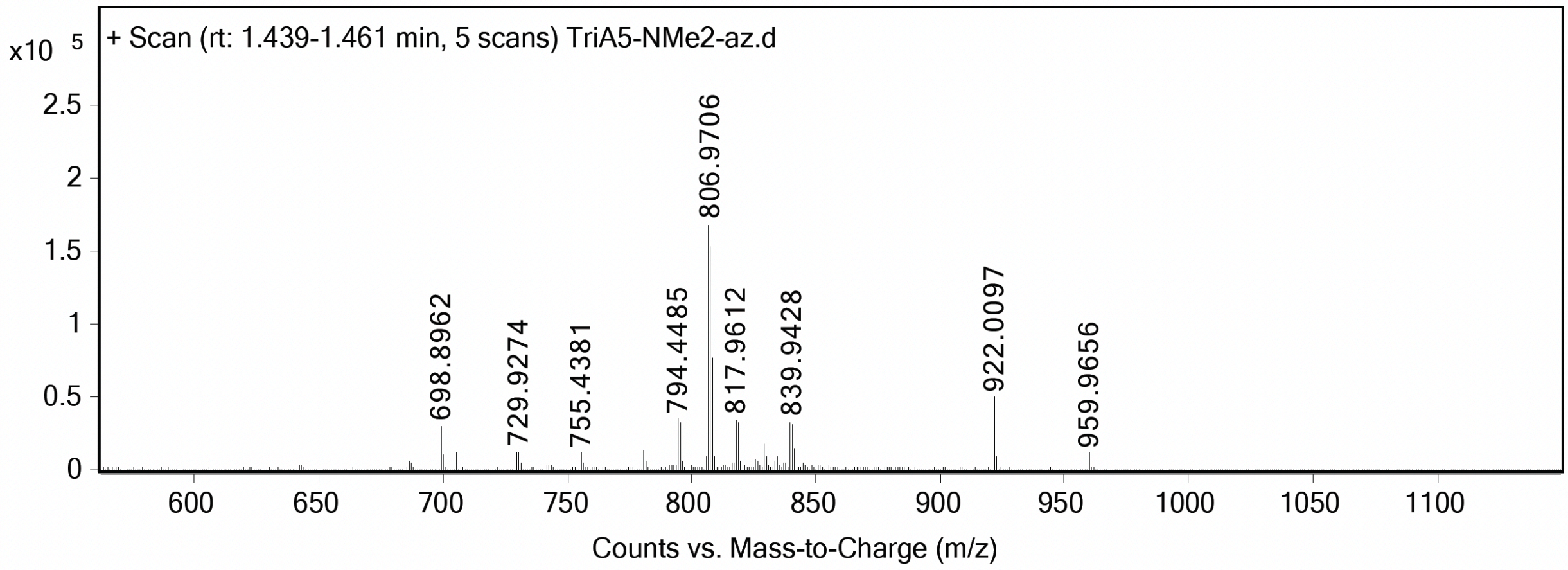

QTOF high resolution mass spectrum of **az2N-Me-OctTriA5** (expected m/z 806.9698 for $[M+2H]^{2+}$).

Synthesis of JSF-2985 analogues

All reagents were purchased from commercial suppliers and used without further purification unless noted otherwise. All chemical reactions occurring solely in an anhydrous organic solvent were executed under an inert atmosphere of nitrogen or argon unless noted otherwise. Reactions conducted at rt were usually at an ambient temperature of 21 – 24 °C. Analytical TLC was carried out with Merck silica gel 60 F_{254} plates. Silica gel column chromatography utilized Teledyne Isco CombiFlash Companion or Rf+ systems. ^{1}H NMR spectra were obtained on Bruker 500 and 600 MHz instruments and are listed in parts per million downfield from TMS. ^{1}H NMR and ^{13}C NMR spectra were recorded on a Bruker 500 MHz instrument. Liquid chromatography-mass spectrometry (LC-MS) was conducted on an Agilent 1260 HPLC coupled to an Agilent 6120 MS. All synthesized compounds were at least 95% pure as judged by their HPLC trace at 220 or 250 nm and were characterized by the expected parent ion/s in the MS. HRMS was executed on an Agilent 6230B Accurate Mass TOF MS.

JSF-2985, JSF-3217, and the key intermediate ethyl 5-((*tert*-butoxycarbonyl)amino)-3-methyl-1*H*-indole-2-carboxylate were synthesized as reported previously.[2, 3]

5-((4-azidobutyl)sulfonamido)-3-methyl-1*H*-indole-2-carboxamide (az-JSF-2985; JSF-6020):

Ethyl 5-amino-3-methyl-1*H*-indole-2-carboxylate trifluoroacetic acid salt: Trifluoroacetic acid (10 mL) was added dropwise to a vigorously stirring solution of ethyl 5-((*tert*-butoxycarbonyl)amino)-3-methyl-1*H*-indole-2-carboxylate (400 mg, 1.25 mmol) in 10 mL DCM. The reaction was stirred at rt for 12 h when at that point no starting material was remaining based on LC/MS analysis. The reaction mixture was concentrated *in vacuo* to give the desired product as a white, gummy semi-solid (170 mg, 0.511 mmol, 40.7%): Calculated for $C_{12}H_{15}N_2O_2$ $[M+H]^+$ = 219.1; found 219.1.

Ethyl 5-((4-chlorobutyl)sulfonamido)-3-methyl-1*H*-indole-2-carboxylate: Ethyl 5-amino-3-methyl-1*H*-indole-2-carboxylate trifluoroacetic acid salt (170 mg, 0.511 mmol) and 4-chlorobutanesulfonyl chloride (98 mg, 0.511 mmol) were all dissolved in 5 mL pyridine at -10 °C. The reaction was stirred at -10 °C for 5 h. The reaction was diluted 10-fold with ethyl acetate and washed with 1N $HCl_{(aq)}$ solution. The organic phase was dried over anhydrous Na_2SO_4 and concentrated *in vacuo* to afford the desired product

as a brown gummy liquid (159 mg, 0.427 mmol, 83.6%): Calculated for $C_{16}H_{22}ClN_2O_4S$ $[M+H]^+$ = 373.1; found 373.1.

Ethyl 5-((4-azidobutyl)sulfonamido)-3-methyl-1*H*-indole-2-carboxylate: To the mixture of ethyl 5-((4-chlorobutyl)sulfonamido)-3-methyl-1*H*-indole-2-carboxylate (159 mg, 0.427 mmol) in 5 mL DMSO was added sodium azide (83.0 mg, 1.28 mmol, 3 equiv). The reaction was heated to 80 °C and stirred overnight. The mixture was subjected to the addition of ice-cold water and extracted with ethyl acetate. The organic phase was dried over anhydrous Na_2SO_4, concentrated *in vacuo*, and purified by silica gel flash column chromatography using a gradient of 0 – 5% MeOH/DCM to afford the product as a brown solid (101 mg, 0.266 mmol, 62.3%): Calculated for $C_{16}H_{22}N_5O_4S$ $[M+H]^+$ = 380.1; found 380.1.

5-((4-azidobutyl)sulfonamido)-3-methyl-1*H*-indole-2-carboxylic acid: To a solution of ethyl 5-((4-azidobutyl)sulfonamido)-3-methyl-1*H*-indole-2-carboxylate (101 mg, 0.266 mmol) in 7 mL 1,4-dioxane was added 3 mL of an aqueous solution of LiOH (56 mg, 1.33 mmol, 5 equiv). The reaction was heated to 70 °C and stirred overnight. The mixture was cooled in an ice bath and 6N $HCl_{(aq)}$ was added slowly. The solution was extracted with ethyl acetate. The organic phase was dried over anhydrous Na_2SO_4 and concentrated *in vacuo* to afford a chromatographically inseparable mixture containing the desired product and two major impurities (m/z = 312.1 and m/z = 366.1) (61 mg): Calculated for $C_{14}H_{18}N_5O_4S$ $[M+H]^+$ = 352.1; found 352.0. The inseparable mixture was used in the next step without further purification.

Perfluorophenyl 5-((4-azidobutyl)sulfonamido)-3-methyl-1*H*-indole-2-carboxylate: The impure mixture containing 5-((4-azidobutyl)sulfonamido)-3-methyl-1*H*-indole-2-carboxylic acid (61 mg, ≤0.173 mmol), 1-ethyl-3-(3-dimethylaminopropyl)carbodiimide hydrochloride (50.0 mg, 0.260 mmol, ≥1.5 equiv), and pentafluorophenol (48.0 mg, 0.260 mmol, ≥1.5 equiv) were dissolved in 15 mL dichloromethane. The reaction was stirred at rt overnight. The reaction was diluted 10-fold with dichloromethane and washed with saturated $NH_4Cl_{(aq)}$ and then saturated aqueous brine solution. The organic phase was separated, dried over anhydrous Na_2SO_4, and concentrated *in vacuo* to afford the product as a brown solid (54 mg, 0.10 mmol, ≥60%): Calculated for $C_{20}H_{17}F_5N_5O_4S$ $[M + H]^+$ = 518.1; found 518.1.

To a solution of perfluorophenyl 5-((4-azidobutyl)sulfonamido)-3-methyl-1H-indole-2-carboxylate (54 mg, 0.104 mmol) in 5 mL acetonitrile was added ammonium hydroxide (1 mL). The reaction was stirred at rt for 12 h. After completion of the reaction as

determined by TLC and LC/MS, the crude reaction mixture was taken up into EtOAc and washed with saturated $NH_4Cl_{(aq)}$ solution and a saturated aqueous brine solution. The organic phase was dried over anhydrous Na_2SO_4 and purified by reverse phase column chromatography using a gradient of 0 – 60% H_2O/ACN to afford the title product as a white solid (16 mg, 0.044 mmol, 42% over 2 steps): ^{1}H NMR (500 MHz, d_6-DMSO) δ 11.2 (s, 1H), 9.42 (s, 1H), 7.37 (m, 4H), 7.11 (dd, *J* = 8.7, 2.0 Hz, 1H), 3.31 (s, 2H), 3.00 (m, 2H), 2.45 (s, 3H), 1.75 (p, *J* = 7.6 Hz, 2H), 1.59 (p, *J* = 7.0 Hz, 2H). Also noted, 3.3 (s, H_2O). Calculated for $C_{14}H_{19}N_6O_3S$ $[M+H]^+$ = 351.1239; found 351.1238.

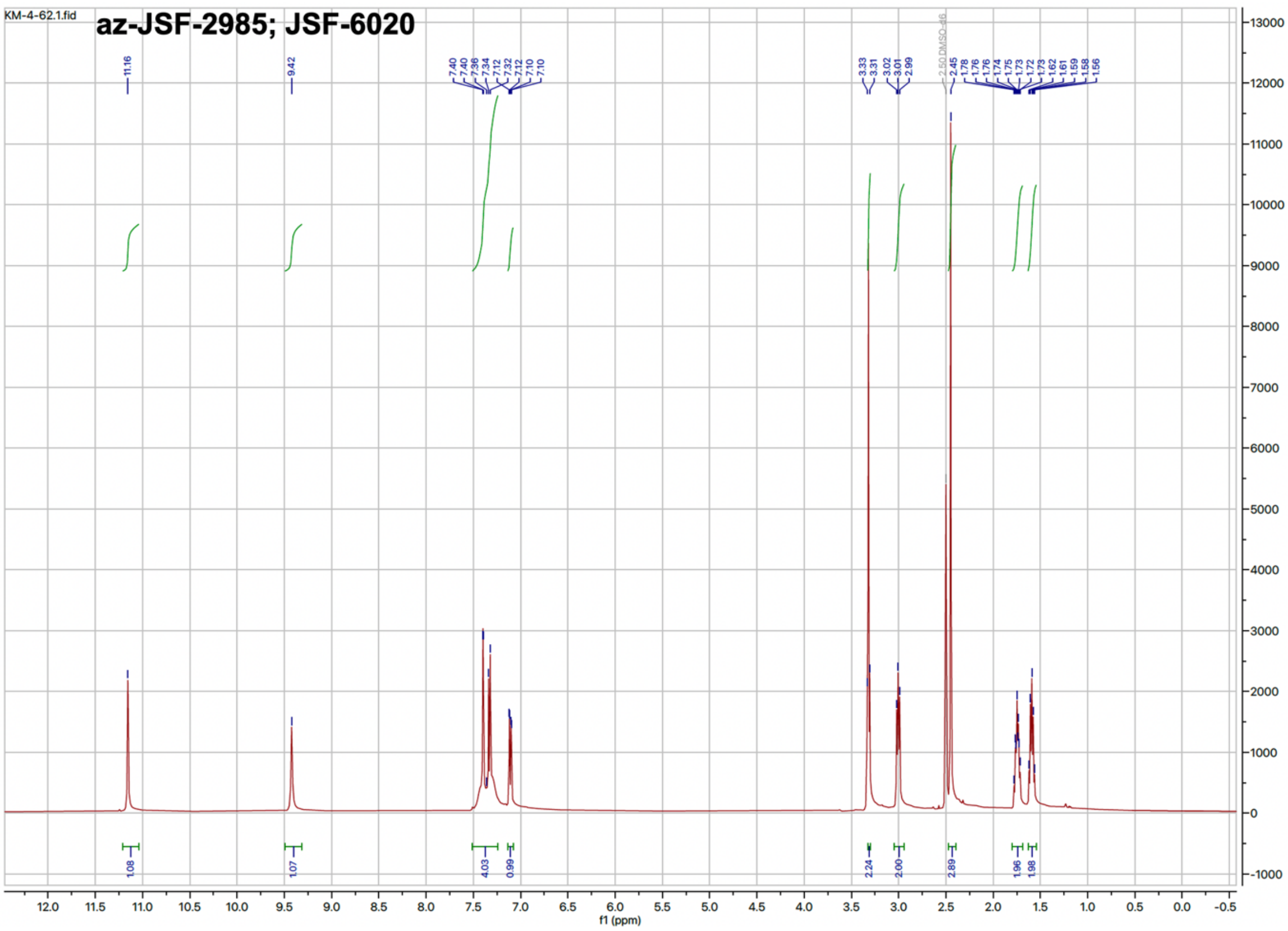

N-(2-(1*H*-pyrazol-3-yl)ethyl)-5-((4-azidobutyl)sulfonamido)-3-methyl-1*H*-indole-2-carboxamide (az-JSF-2985-pyrazole; JSF-6009):

Ethyl 5-((4-azidobutyl)sulfonamido)-3-methyl-1*H*-indole-2-carboxylate: Ethyl 5-amino-3-methyl-1H-indazole-2-carboxylate (100 mg, 0.465 mmol), and 4-azidobutanesulphonyl chloride (110 mg, 0.588 mmol, 1.2 equiv) were all dissolved in 5 mL pyridine at 0 ℃. The reaction was stirred at rt for 12 h. The reaction was diluted 10-fold with ethyl acetate and washed with 1N $HCl_{(aq)}$ solution. The organic phase was

dried over anhydrous Na_2SO_4 and concentrated in *vacuo* to obtain a faint yellow liquid. The crude reaction product was purified via flash chromatography over silica gel eluting with MeOH/DCM (0 – 5%) to obtain the desired product as a brown solid (97.0 mg, 0.255 mmol, 56.0%): ^{1}H NMR (500 MHz, d_6-DMSO) δ 11.5 (s, 1H), 9.5 (s, 1H), 7.45 (s, 1H), 7.37 (d, *J* = 8.7 Hz, 1H), 7.18 (dd, *J* = 8.8, 2.1 Hz, 1H), 4.33 (m, 2H), 3.33 (d, *J* = 6.6 Hz, 2H), 3.03 (t, *J* = 7.8 Hz, 2H), 2.5 (s, 3H), 1.75 (m, 2H), 1.60 (m, 2H), 1.36 (t, *J* = 7.0 Hz, 3H). Calculated for $C_{16}H_{22}N_5O_4S$ [M+H]+ = 380.1, found 380.0.

5-((4-azidobutyl)sulfonamido)-3-methyl-1*H*-indole-2-carboxylic acid: To a solution of ethyl 5-((4-azidobutyl)sulfonamido)-3-methyl-1*H*-indole-2-carboxylate (97.0 mg, 0.255 mmol) in 7 mL 1,4-dioxane was added 3 mL of an aqueous solution of LiOH (31.0 mg, 1.27 mmol, 5 equiv). The reaction was heated to 70 °C and stirred overnight. The mixture was cooled and 1N $HCl_{(aq)}$ was added slowly until the solution reached pH 5 – 6. The reaction mixture was extracted with ethyl acetate. The organic phase was dried over anhydrous Na_2SO_4 and concentrated *in vacuo* to afford the desired product as a brown solid (40.0 mg, 0.113 mmol, 44.0 %): Calculated for $C_{14}H_{18}N_5O_4S$ $[M+H]^+$: 352.1, found 352.1.

Perfluorophenyl 5-((4-azidobutyl)sulfonamido)-3-methyl-1*H*-indole-2-carboxylate: 5-((4-azidobutyl)sulfonamido)-3-methyl-1H-indole-2-carboxylic acid (40.0 mg, 0.113 mmol), 1-ethyl-3-(-3-dimethylaminopropyl)carbodiimide hydrochloride (33.0 mg, 0.170 mmol, 1.5 equiv), and pentafluorophenol (31.0 mg, 0.170 mmol, 1.5 equiv) were dissolved in 5 mL dichloromethane. The reaction was stirred at rt overnight. The reaction was diluted 10-fold with dichloromethane and washed with saturated $NH_4Cl_{(aq)}$ and saturated aqueous brine solution. The organic phase was separated, dried over anhydrous Na_2SO_4, and concentrated *in vacuo* to afford the product as a yellow solid (47.0 mg, 0.090 mmol, 80.0%): Calculated for $C_{20}H_{17}F_5N_5O_4S$ $[M + H]^+$ = 518.1; found 518.1.

To a solution of perfluorophenyl 5-((4-azidobutyl) sulfonamido)-3-methyl-1*H*-indole-2-carboxylate (47.0 mg, 0.090 mmol) in 5 mL acetonitrile was added 2-(1*H*-pyrazol-3-yl)ethan-1-amine dihydrochloride (25.0 mg, 0.135 mmol, 1.5 equiv). The reaction was stirred at rt for 2 h. Upon completion, the reaction was concentrated *in vacuo* to obtain a brown solid. The crude reaction product was purified via flash chromatography over silica gel eluting with MeOH/DCM (0 – 7%) to obtain the desired product as a brown solid (39.0 mg, 0.087 mmol, 98.0%): ^{1}H NMR (500 MHz, d_6-DMSO) δ 12.5 (br s, 1H),

11.2 (s, 1H), 9.45 (s, 1H), 7.91 (s, 1H), 7.61 (br s, 1H), 7.40 (d, J = 2.1 Hz, 1H), 7.34 (d, J = 8.6 Hz, 1H), 7.11 (dd, J = 8.7, 2.0 Hz, 1H), 6.13 (s, 1H), 3.56 (q, J = 6.9 Hz, 2H), 3.32 (d, J = 8.0 Hz, 2H), 3.01 (m, 2H), 2.89 (d, J = 7.7 Hz, 2H), 2.43 (s, 3H), 1.76 (m, 2H), 1.60 (m, 2H). Also noted 3.3 (s, H_2O) and 1.2 (s). Calculated for $C_{19}H_{25}N_8O_3S$ $[M+H]^+$ = 445.1770; found 445.1760.

az-JSF-2985-pyrazole; JSF-6009

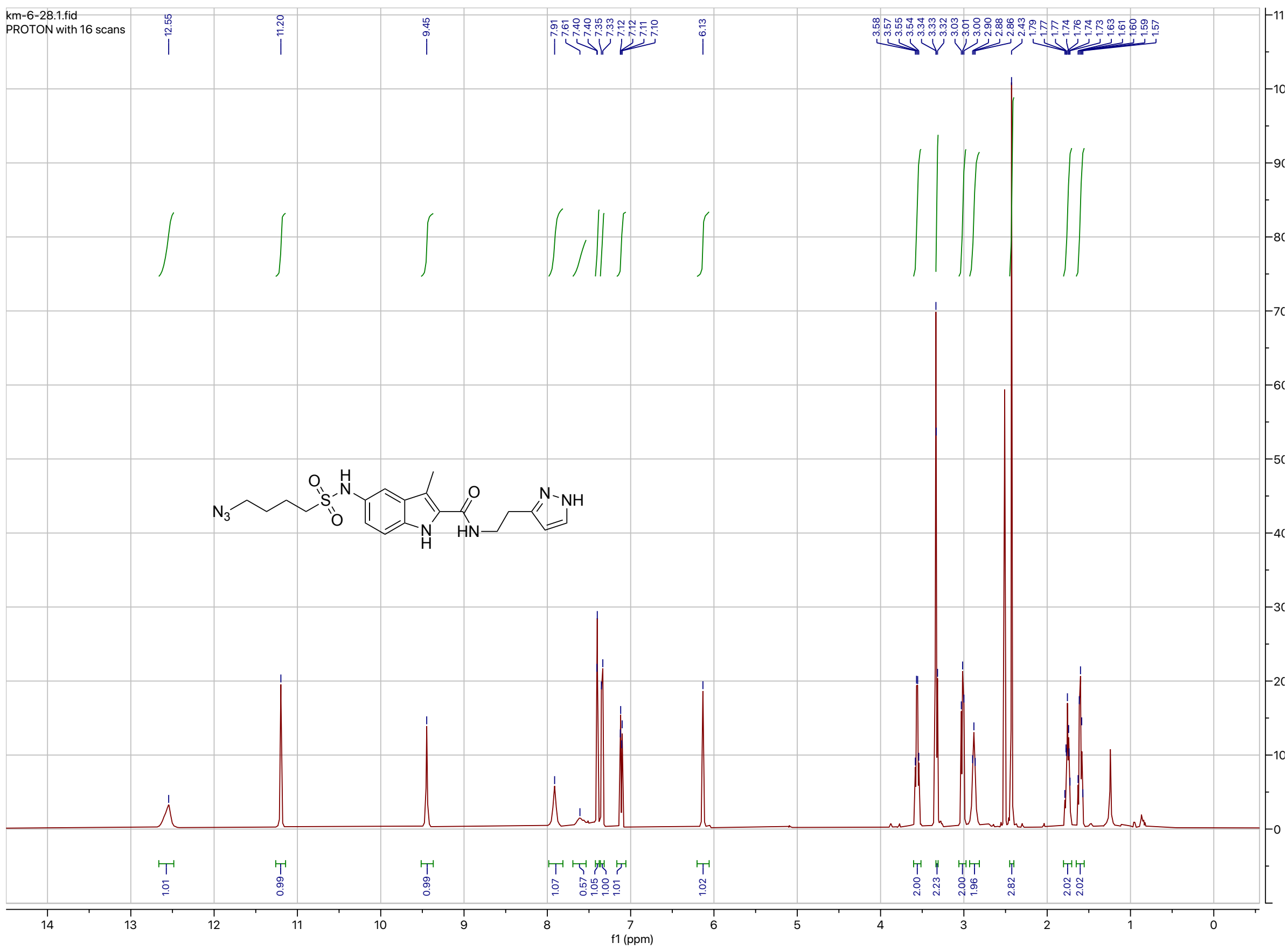

N-(2-(1H-imidazol-2-yl)ethyl)-5-((4-azidobutyl)sulfonamido)-3-methyl-1H-indole-2-carboxamide (az-JSF-2985-imidazole; JSF-5098):

To a solution of perfluorophenyl 5-((4-azidobutyl) sulfonamido)-3-methyl-1*H*-indole-2-carboxylate (250 mg, 0.483 mmol) in 5 mL acetonitrile was added 2-(1*H*-imidazol-2-yl)ethan-1-amine dihydrochloride (133 mg, 0.725 mmol) and triethylamine (336 µL, 2.41 mmol, 5.0 equiv). The reaction was stirred at rt for 2 h. Upon completion, the reaction was concentrated *in vacuo* to obtain a brown solid. The crude reaction product was purified via flash chromatography over silica gel eluting with MeOH/DCM (0

– 10%) to obtain the desired product as a yellow solid (8.0 mg, 0.018 mmol, 4.0%): ^{1}H NMR (500 MHz, MeOD) δ 7.50 (d, *J* = 2.1 Hz, 1H), 7.35 (d, *J* = 8.7 Hz, 1H), 7.18 (dd, *J* = 8.7, 2.0 Hz, 1H), 7.0 (s, 2H), 3.75 (t, *J* = 6.9 Hz, 2H), 3.29 (d, *J* = 6.6 Hz, 2H), 3.06 (m, 4H), 2.46 (s, 3H), 1.89 (m, 2H), 1.66 (m, 2H). Also noted 4.89 (s, H_2O), and 1.28 (s). 4 NHs were unaccounted for, presumably due to H/D exchange with the NMR solvent. Calculated for $C_{19}H_{25}N_8O_3S$ $[M+H]^+$ = 445.1770; found 445.1762.

az-JSF-2985-imidazole; JSF-5098

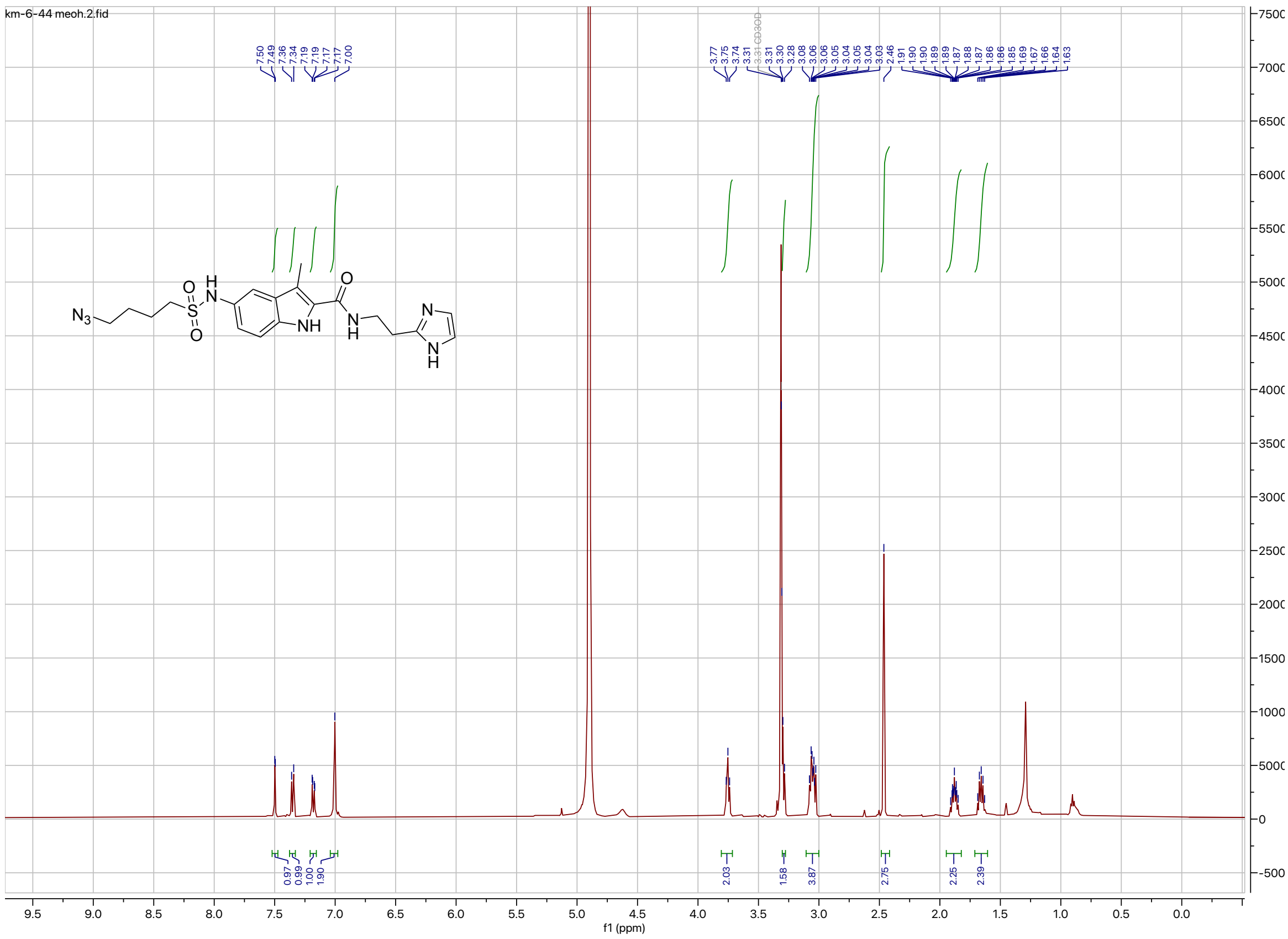

5-((4-azidobutyl)sulfonamido)-3-methyl-*N*-(2-(pyrrolidin-3-yl)ethyl)-1*H*-indole-2-carboxamide (az-JSF-2985-pyrrolidine; JSF-6001):

tert-butyl 3-(2-(5-((4-azidobutyl)sulfonamido)-3-methyl-1*H*-indole-2-carboxamido)ethyl)pyrrolidine-1-carboxylate: To a solution of perfluorophenyl 5-((4-azidobutyl) sulfonamido)-3-methyl-1*H*-indole-2-carboxylate (241 mg, 0.466 mmol) in 5 mL acetonitrile was added *tert*-butyl 3-(2-aminoethyl)pyrrolidine-1-carboxylate (100 mg, 0.466 mmol). The reaction was stirred at rt for 2 h. Upon completion, the reaction was concentrated *in vacuo* to obtain a brown solid. The crude reaction product was purified via flash chromatography over silica gel eluting with MeOH/DCM (0 – 5%) to obtain

the desired product as a yellow solid (71.0 mg, 0.129 mmol, 28.0%): ^{1}H NMR (500 MHz, d_6-DMSO) δ 11.2 (s, 1H), 9.45 (s, 1H), 7.88 (d, *J* = 6.8 Hz, 1H), 7.39 (d, *J* = 2.0 Hz, 1H), 7.33 (d, *J* = 8.7 Hz, 1H), 7.10 (dd, *J* = 8.7, 2.1 Hz, 1H), 3.49 (m, 2H), 3.31 (m, 4H), 3.15 (m, 1H), 3.0 (dd, *J* = 8.9, 6.6 Hz, 2H), 2.82 (q, *J* = 10.1 Hz, 1H), 2.44 (s, 3H), 2.17 (m, 1H), 1.99 (s, 1H), 1.74 (m, 2H), 1.60 (m, 4H), 1.48 (m, 1H), 1.38 (d, 9H). Also noted 3.3 (s, H_2O), 2.1 (s, acetone) and 1.2 (s). Calculated for $C_{20}H_{30}N_7O_3S$ $[M+H-100]^+$ 448.3, found 448.2.

Trifluoroacetic acid (1 mL) was added dropwise to a vigorously stirring solution of *tert*-butyl 3-(2-(5-((4-azidobutyl)sulfonamido)-3-methyl-1*H*-indole-2-carboxamido)ethyl) pyrrolidine-1-carboxylate (70.0 mg, 0.129 mmol) in 5 mL DCM. The reaction was stirred at rt for 2 h when no starting material was observed based on LC/MS analysis. The reaction mixture was diluted in 10 mL DCM and washed with saturated aqueous $NaHCO_3$ solution. The organic layer was concentrated *in vacuo* and purified via flash chromatography over silica gel eluting with MeOH/DCM (0 – 30%) to obtain the desired product as a yellow solid (26.0 mg, 0.058 mmol, 46.0%): ^{1}H NMR (500 MHz, d_6-DMSO) δ 11.6 (s, 1H), 9.13 (br s, 1H), 8.29 (t, *J* = 5.5 Hz, 1H), 7.39 (d, *J* = 2.0 Hz, 1H), 7.32 (d, *J* = 8.6 Hz, 1H), 7.10 (dd, *J* = 8.7, 2.1 Hz, 1H), 3.32 (t, *J* = 6.8 Hz, 5H), 3.20 (m, 1H), 3.06 (m, 1H), 3.01 (m, 2H), 2.72 (dd, *J* = 11.3, 9.0 Hz, 1H), 2.46 (s, 3H), 2.26 (m, 1H), 2.12 (m, 1H), 1.74 (m, 2H), 1.66 (m, 2H), 1.59 (m, 2H), 1.51 (m, 1H). Also noted 3.3 (s, H_2O). One H was unaccounted for. Calculated for $C_{20}H_{30}N_7O_3S$ $[M+H]^+$ 448.2131, found 448.2133.

az-JSF-2985-pyrrolidine; JSF-6001

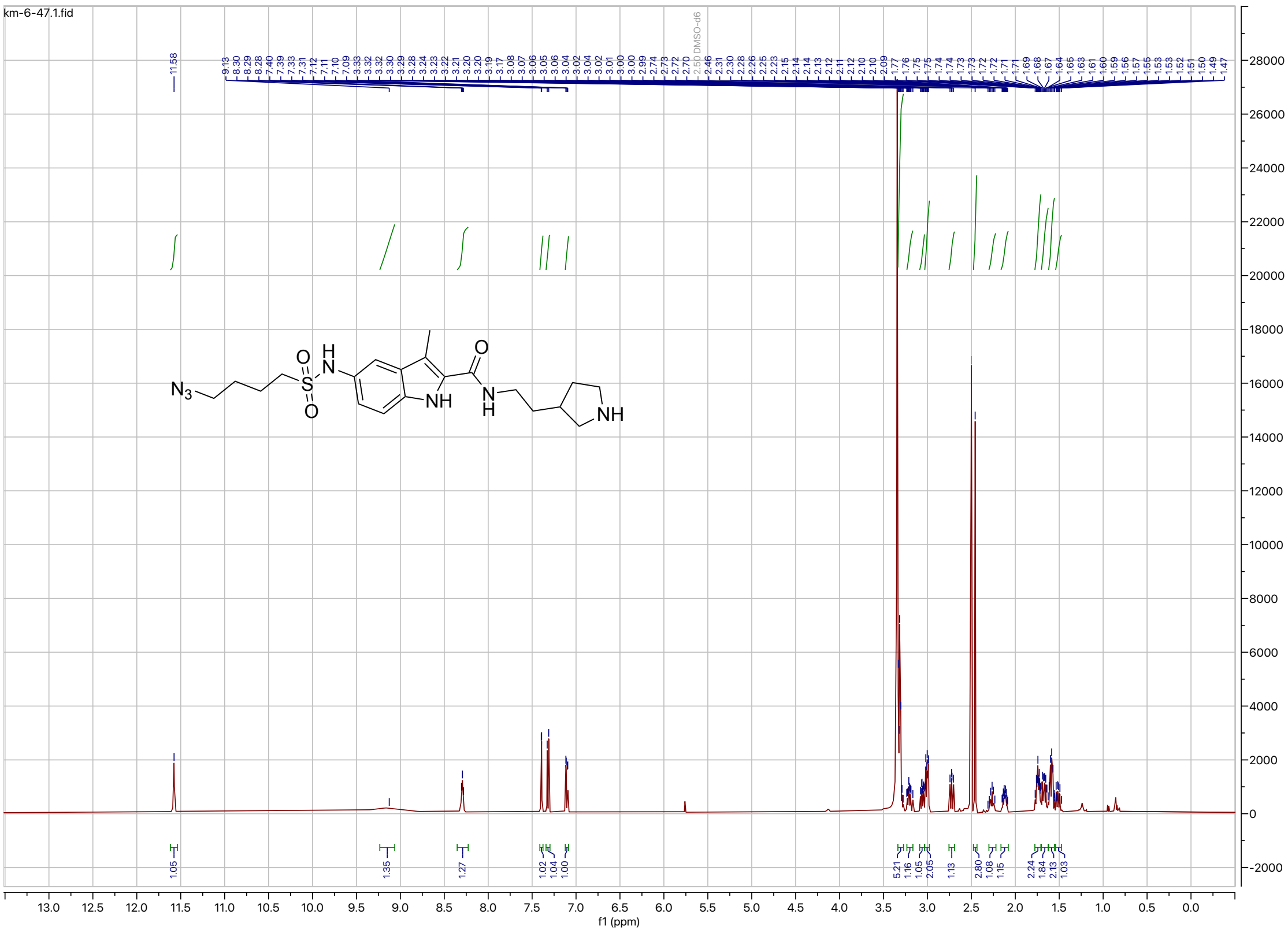

5-((4-azidobutyl)sulfonamido)-*N*-(2-cyclopentylethyl)-3-methyl-1*H*-indole-2-carboxamide (az-JSF-2985-cyclopentane; JSF-5093):

Perfluorophenyl 5-((*tert*-butoxycarbonyl)amino)-3-methyl-1*H*-indole-2-carboxylate: 5-((*tert*-butoxycarbonyl)amino)-3-methyl-1*H*-indole-2-carboxylic acid (400 mg, 1.37 mmol), 1-ethyl-3-(3-dimethylaminopropyl)carbodiimide hydrochloride (395 mg, 2.06 mmol), and pentafluorophenol (379 mg, 2.06 mmol) were dissolved in 30 mL dichloromethane. The reaction was stirred at rt overnight. The reaction was diluted 10-fold with dichloromethane and washed with saturated $NH_4Cl_{(aq)}$ and then saturated aqueous brine solution. The organic phase was separated, dried over anhydrous Na_2SO_4, and concentrated *in vacuo* to afford the desired product as an off-white solid (310 mg, 0.679 mmol, 49.3%): Calculated for $C_{21}H_{18}F_5N_2O_4$ $[M + H - 56]^+ = 401.0$; found 401.0.

tert-butyl (2-((2-cyclopentylethyl)carbamoyl)-3-methyl-1*H*-indol-5-yl)carbamate: To the pentafluoro ester (310 mg, 0.679 mmol) in 10 mL acetonitrile was added 2-cyclopentylethan-1-amine (153 mg, 1.35 mmol, 2 equiv). The reaction was stirred at rt for 4 h. After completion of reaction as determined by TLC and LC/MS, the crude

reaction mixture was taken up into EtOAc and washed with saturated $NH_4Cl_{(aq)}$ solution and then a saturated aqueous brine solution. The organic phase was dried over anhydrous Na_2SO_4 and purified by silica gel flash column chromatography using a gradient of 0 – 10% MeOH/DCM to afford the desired product as a yellow solid (209 mg, 0.542 mmol, 80.0%): Calculated for $C_{22}H_{32}N_3O_3$ $[M+H]^+$ = 386.2; found 386.2.

5-amino-*N*-(2-cyclopentylethyl)-3-methyl-1*H*-indole-2-carboxamide: Trifluoroacetic acid (3 mL) was added dropwise to a vigorously stirring solution of *tert*-butyl (2-((2-cyclopentylethyl) carbamoyl)-3-methyl-1*H*-indol-5-yl)carbamate (300 mg, 1.05 mmol) in 15 mL DCM. The reaction was stirred at rt for 12 h after which no starting material was remaining based on LC/MS analysis. The reaction was neutralized with saturated aqueous $NaHCO_3$ solution until the pH of the aqueous layer was approximately 8. The DCM layer was collected, and the remaining aqueous phase was extracted twice with EtOAc. The organic fractions were pooled and washed with saturated aqueous brine solution. The organic phase was dried over anhydrous Na_2SO_4 and concentrated *in vacuo* to give the desired product as a brown solid (210 mg, 0.736 mmol, 94.5%): Calculated for $C_{17}H_{24}N_3O$ $[M+H]^+$ = 286.2; found 286.1.

5-((4-chlorobutyl)sulfonamido)-*N*-(2-cyclopentylethyl)-3-methyl-1*H*-indole-2-carboxamide: 5-amino-*N*-(2-cyclopentylethyl)-3-methyl-1*H*-indole-2-carboxamide (210 mg, 0.736 mmol), and 4-chlorobutanesulfonyl chloride (155 mg, 0.810 mmol,1.1 equiv) were dissolved in 10 mL pyridine at 0 °C. The reaction was stirred at rt overnight. The reaction was diluted 10-fold with ethyl acetate and washed with 1N $HCl_{(aq)}$ solution. The organic phase was dried over anhydrous Na_2SO_4, concentrated *in vacuo*, and purified by silica gel flash column chromatography using a gradient of 0 – 5% MeOH/DCM to afford the desired product as a yellow solid (136 mg, 0.309 mmol, 42.1%): Calculated for $C_{21}H_{31}ClN_3O_3S$ $[M+H]^+$ = 440.2; found 440.1.

To a solution of 5-((4-chlorobutyl)sulfonamido)-*N*-(2-cyclopentylethyl)-3-methyl-1*H*-indole-2-carboxamide (125 mg, 0.284 mmol) in 10 mL DMSO was added sodium azide (46.0 mg, 0.711 mmol). The reaction was heated to 80 °C and stirred overnight. The mixture was subjected to the addition of ice-cold water and extracted with ethyl acetate. The organic phase was dried over anhydrous Na_2SO_4, concentrated *in vacuo*, and purified by silica gel flash column chromatography using a gradient of 0 – 30% EtOAc/hexanes to afford the title product as a white solid (21 mg, 0.047 mmol, 16%): ^{1}H NMR (500 MHz, d_6-DMSO) δ 11.1 (s, 1H), 9.43 (s, 1H), 7.81 (t, *J* = 5.8 Hz, 1H), 7.39 (s, 1H), 7.33 (d, *J* = 8.7 Hz, 1H), 7.10 (dd, *J* = 8.6, 2.1 Hz, 1H), 3.30 (d, *J* = 8.9

Hz, 2H), 3.00 (m, 2H), 2.43 (s, 3H), 1.76 (dtd, *J* = 18.0, 9.7, 5.9 Hz, 5H), 1.57 (m, 6H), 1.49 (dd, *J* = 7.5, 4.7 Hz, 3H), 1.12 (q, *J* = 8.6 Hz, 3H): Also noted, 3.3 (s, H_2O) and 1.3 (s). Calculated for $C_{21}H_{31}N_6O_3S$ $[M+H]^+$ = 447.2178; found 447.2156.

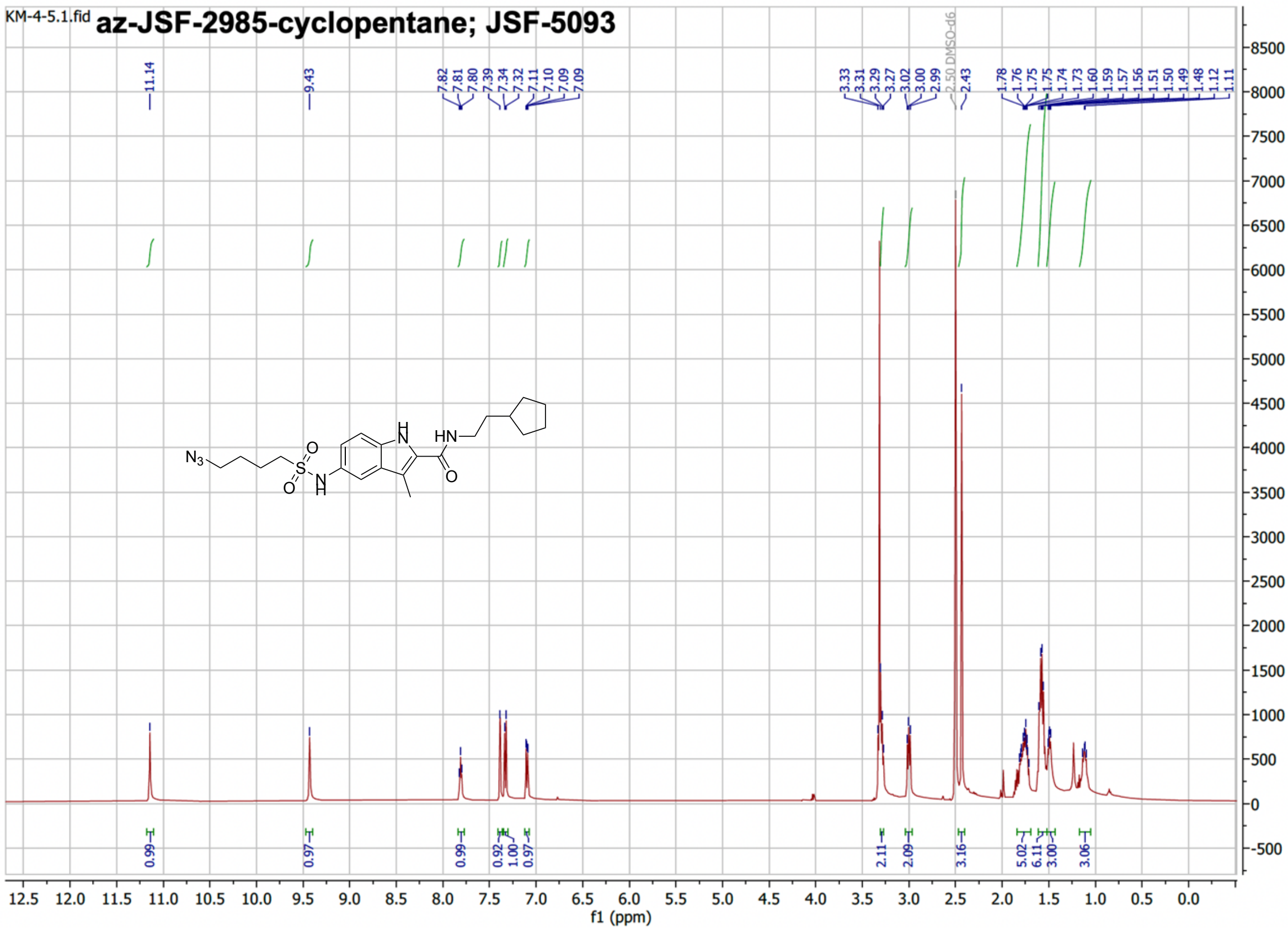

4-azido-*N*-(3-methyl-1*H*-indazol-5-yl) butane-1-sulfonamide (JSF-4945):

4-chloro-*N*-(3-methyl-1*H*-indazol-5-yl) butane-1-sulfonamide: To a solution of 3-methyl-1H-indazol-5-amine (0.500 g, 3.39 mmol) in pyridine (15 mL), 4-chlorobutane-1-sulfonyl chloride (0.584 g, 3.05 mmol) was added dropwise at -10 °C and the mixture was stirred at rt for 12 h. The solvent was removed *in vacuo* and the crude was partitioned between EtOAc (25 mL) and 0.1N $HCl_{(aq)}$ (15 mL). The organic phase was dried over anhydrous Na_2SO_4 and then concentrated *in vacuo*. The crude product mixture was purified by silica gel chromatography using 0 – 100% EtOAc/hexanes to afford the desired product as white solid (0.720 g, 2.38 mmol, 70.2%): ^{1}H NMR (500 MHz, d_6-DMSO) δ 12.6 (s, 1H), 9.58 (s, 1H), 7.48 (d, *J* = 1.3 Hz, 1H), 7.42 (d, *J* = 8.8 Hz, 1H), 7.22 (dd, *J* = 8.8, 1.9 Hz, 1H), 3.62 (t, *J* = 6.0 Hz, 2H), 3.04 (m, 2H), 2.45 (s, 3H), 1.86 - 1.75 (m, 4H). Also noted 3.32 (s, H_2O). Calculated for $C_{12}H_{17}ClN_3O_2S$ $[M+H]^+$ = 302.0730; found 302.0738.

To a stirred solution of 4-chloro-*N*-(3-methyl-1*H*-indazol-5-yl) butane-1-sulfonamide (100 mg, 0.331 mmol) in 1 mL of dry DMSO at rt was added sodium azide (21 mg, 0.331 mmol). The reaction was then heated at 80 °C for 3 h. After completion of the reaction as judged by TLC, the crude reaction mixture was then dissolved in ethyl acetate and washed with water followed by saturated aqueous brine solution. The organic layer was dried over anhydrous sodium sulfate and concentrated *in vacuo.* The solid residue was purified by flash column chromatography (0 - 100% EtOAc in hexanes) to afford the title compound as a white solid (51 mg, 0.16 mmol, 50%): ^{1}H NMR (500 MHz, d$_6$-DMSO) δ 12.63 (s, 1H), 9.55 (s, 1H), 7.47 (d, *J* = 1.5 Hz, 1H), 7.42 (d, *J* = 8.8 Hz, 1H), 7.22 (dd, *J* = 8.8, 1.9 Hz, 1H), 3.33 (d, *J* = 6.6 Hz, 2H), 3.06 - 3.00 (m, 2H), 2.45 (s, 3H), 1.78 - 1.71 (m, 2H), 1.63 - 1.56 (m, 2H). Also noted δ 5.75 (s, CH_2Cl_2), 3.32 (s). Calculated for $C_{12}H_{17}N_6O_2S$ [M+H]$^+$ = 309.1134; found 309.1141.

JSF-4945

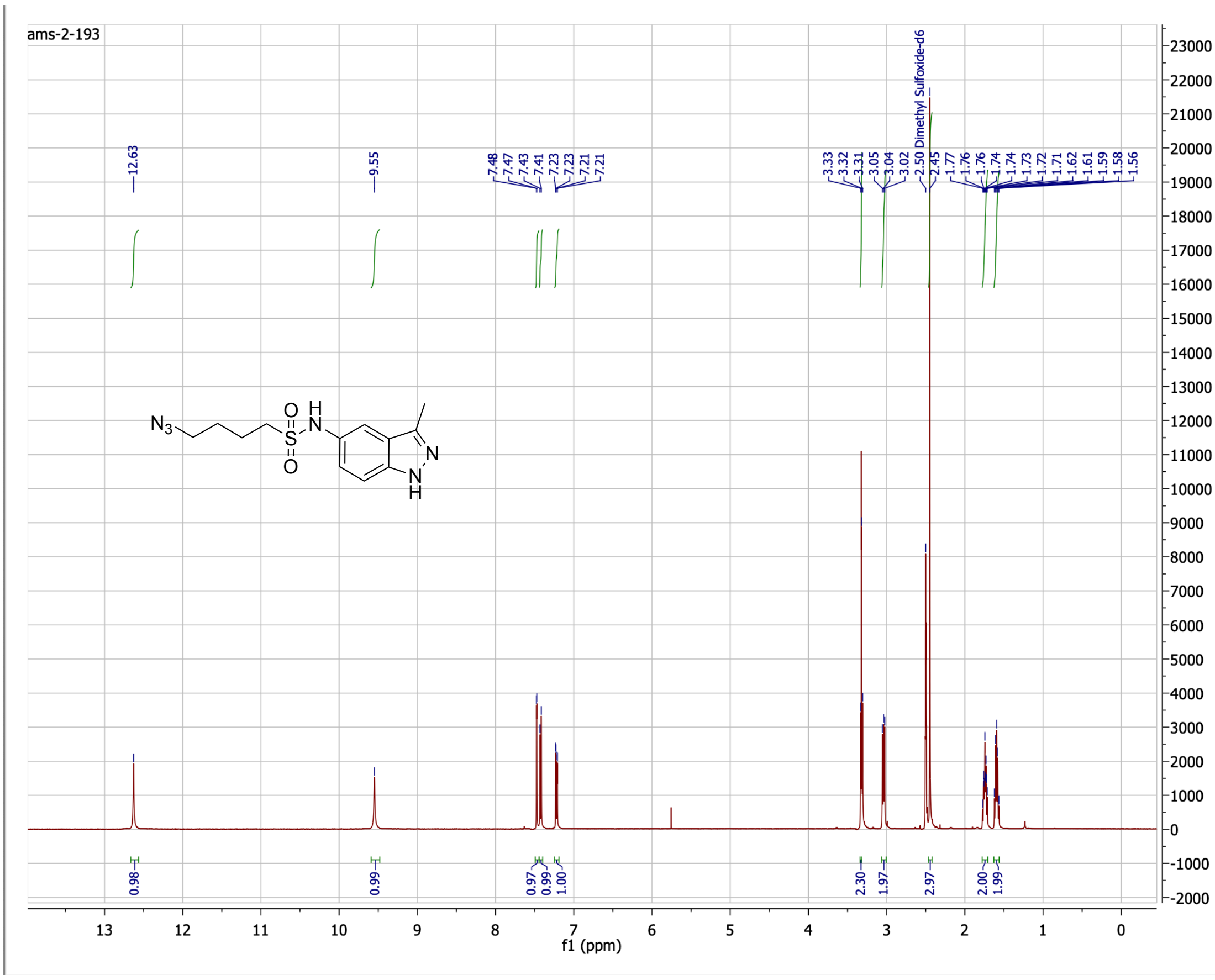

References

1. Dash, R.; Liu, Z.; Lepori, I.; Chordia, M. D.; Ocius, K.; Holsinger, K.; Zhang, H.; Kenyon, R.; Im, W.; Siegrist, M. S.; Pires, M. M., Systematic Determination of the Impact of Structural Edits on Peptide Accumulation into Mycobacteria. *ACS Chem Biol* **2025,** *20* (8), 1962-1979.
2. Kumar, P.; Capodagli, G. C.; Awasthi, D.; Shrestha, R.; Maharaja, K.; Sukheja, P.; Li, S. G.; Inoyama, D.; Zimmerman, M.; Ho Liang, H. P.; Sarathy, J.; Mina, M.; Rasic, G.; Russo, R.; Perryman, A. L.; Richmann, T.; Gupta, A.; Singleton, E.; Verma, S.; Husain, S.; Soteropoulos, P.; Wang, Z.; Morris, R.; Porter, G.; Agnihotri, G.; Salgame, P.; Ekins, S.; Rhee, K. Y.; Connell, N.; Dartois, V.; Neiditch, M. B.; Freundlich, J. S.; Alland, D., Synergistic Lethality of a Binary Inhibitor of Mycobacterium tuberculosis KasA. *mBio* **2018,** *9* (6).
3. Inoyama, D.; Awasthi, D.; Capodagli, G. C.; Tsotetsi, K.; Sukheja, P.; Zimmerman, M.; Li, S. G.; Jadhav, R.; Russo, R.; Wang, X.; Grady, C.; Richmann, T.; Shrestha, R.; Li, L.; Ahn, Y. M.; Ho Liang, H. P.; Mina, M.; Park, S.; Perlin, D. S.; Connell, N.; Dartois, V.; Alland, D.; Neiditch, M. B.; Kumar, P.; Freundlich, J. S., A Preclinical Candidate Targeting Mycobacterium tuberculosis KasA. *Cell Chem Biol* **2020,** *27* (5), 560-570.e10.
