# Supplementary material for: Identification of chemical features for improved outer membrane permeation in mycobacteria using machine learning
Source: Nat Microbiol. 2026 Jun 30;11(9):2647–65. doi: 10.1038/s41564-026-02412-5 (PMC13524814; doi:10.1038/s41564-026-02412-5)
Supplement: Supplementary file 2 [file 41564_2026_2412_MOESM2_ESM.pdf]

nature portfolio

Corresponding author(s): M. Sloan Siegrist

Last updated by author(s): May 27, 2026

Reporting Summary

Nature Portfolio wishes to improve the reproducibility of the work that we publish. This form provides structure for consistency and transparency in reporting. For further information on Nature Portfolio policies, see our Editorial Policies and the Editorial Policy Checklist.

Statistics

For all statistical analyses, confirm that the following items are present in the figure legend, table legend, main text, or Methods section.

n/a	Confirmed	
☐	☒	The exact sample size (*n*) for each experimental group/condition, given as a discrete number and unit of measurement
☐	☒	A statement on whether measurements were taken from distinct samples or whether the same sample was measured repeatedly
☐	☒	The statistical test(s) used AND whether they are one- or two-sided<br>*Only common tests should be described solely by name; describe more complex techniques in the Methods section.*
☐	☒	A description of all covariates tested
☐	☒	A description of any assumptions or corrections, such as tests of normality and adjustment for multiple comparisons
☐	☒	A full description of the statistical parameters including central tendency (e.g. means) or other basic estimates (e.g. regression coefficient) AND variation (e.g. standard deviation) or associated estimates of uncertainty (e.g. confidence intervals)
☐	☒	For null hypothesis testing, the test statistic (e.g. *F*, *t*, *r*) with confidence intervals, effect sizes, degrees of freedom and *P* value noted<br>*Give P values as exact values whenever suitable.*
☐	☒	For Bayesian analysis, information on the choice of priors and Markov chain Monte Carlo settings
☐	☒	For hierarchical and complex designs, identification of the appropriate level for tests and full reporting of outcomes
☐	☒	Estimates of effect sizes (e.g. Cohen's *d*, Pearson's *r*), indicating how they were calculated

Our web collection on statistics for biologists contains articles on many of the points above.

Software and code

Policy information about availability of computer code

Data collection

For the activity data relative to MSLMR and TAACF we used Collaborative Drug Discovery software, as specified in the methods section and figure legends. The activity data for the activity screening against Rv3671c Mtb purified enzyme publicly available from PubChem (AID 2606).

Data analysis

For cheminformatics and machine learning analyses, we used Google Colab for our coding notebooks and the Visual Studio Code software (version 1.99.0) for scripting. The coding language used is Python (version 3.10). All codes are publicly available on GitHub: https://github.com/Nevbarunegbe/Mycomembrane-permeability-project.
As specify in the methods section, we used the Collaborative Drug Discovery software to perform some scaffold analysis.
Software packages used for stastical analyses:
For all statistical analyses, we used the
- scipy package on python (version: 1.13.1)
- scikit-learn package for evaluation metric calculations. Used for calculations of Spearman correlations, performance evaluation metrics i.e root mean squared error (RMSE), coefficient of determination (R2), mean absolute error (MAE) (version 1.3.2).
- GraphPad Prism 10

Other software packages:
For machine learning modeling
- PyTorch (version 2.5.1+cu124)
- Chemprop (version 1.6.1)
- Visual Code Studio (version 1.121.0) to write python scripts
- Scikit learn package (version 1.3.2)
- RDKit package for cheminformatic workflows (version 2024.9.5)

- SHAP (version version 0.5.6)
- UMAP (version 0.5.7)
Data processing
- Pandas (version 2.2.2)
- NumPy (version 1.26.4)

Data Plotting:
- Matplotlib for machine learning and cheminformatics plots (version 3.10.0)
- GraphPad Prism 10

FACS Diva for flow cytometry data collection and analysis
Microsoft Excel 365 for data elaboration
BioTek Gen5 for spectrophotometer plate reader data collection

For manuscripts utilizing custom algorithms or software that are central to the research but not yet described in published literature, software must be made available to editors and reviewers. We strongly encourage code deposition in a community repository (e.g. GitHub). See the Nature Portfolio guidelines for submitting code & software for further information.

Data

Policy information about availability of data

All manuscripts must include a data availability statement. This statement should provide the following information, where applicable:

- Accession codes, unique identifiers, or web links for publicly available datasets
- A description of any restrictions on data availability
- For clinical datasets or third party data, please ensure that the statement adheres to our policy

Data Availability

Data produced in this manuscript are available as Supplemental material (Supplemental Files). MLSMR and TAAC antitubercular drug activity data are available from Collaborative Drug Discovery (CDD). Activity data against the purified Mtb enzyme Rv3671c are available from PubChem (AID 2606).

Code Availability

All code and saved model weights for the cheminformatics analyses and MycoPermeNet model are publicly available on github: https://github.com/Nevbarunegbe/Mycomembrane-permeability-project.

Research involving human participants, their data, or biological material

Policy information about studies with human participants or human data. See also policy information about sex, gender (identity/presentation), and sexual orientation and race, ethnicity and racism.

Reporting on sex and gender	N/A
Reporting on race, ethnicity, or other socially relevant groupings	N/A
Population characteristics	N/A
Recruitment	N/A
Ethics oversight	N/A

Note that full information on the approval of the study protocol must also be provided in the manuscript.

Field-specific reporting

Please select the one below that is the best fit for your research. If you are not sure, read the appropriate sections before making your selection.

☒ Life sciences ☐ Behavioural & social sciences ☐ Ecological, evolutionary & environmental sciences

For a reference copy of the document with all sections, see nature.com/documents/nr-reporting-summary-flat.pdf

Life sciences study design

All studies must disclose on these points even when the disclosure is negative.

Sample size	Experiments were performed in technical singlicate, duplicate, or triplicate and repeated 2-3 times. The full screening of the Dong/Sharpless azide library in Mtb was performed once; however, a panel of ~150 compounds, equally distributed across the library plates, were re-tested and found to reproduce the first replicate. See Methods for details.

Data exclusions	No data were excluded
Replication	All the experiments in the manuscript have been performed at least 2-3 times.
Randomization	N/A
Blinding	N/A

Reporting for specific materials, systems and methods

We require information from authors about some types of materials, experimental systems and methods used in many studies. Here, indicate whether each material, system or method listed is relevant to your study. If you are not sure if a list item applies to your research, read the appropriate section before selecting a response.

Materials & experimental systems

n/a	Involved in the study
☐	☐ Antibodies
☐	☐ Eukaryotic cell lines
☐	☐ Palaeontology and archaeology
☐	☐ Animals and other organisms
☐	☐ Clinical data
☐	☐ Dual use research of concern
☐	☐ Plants

Methods

n/a	Involved in the study
☐	☐ ChIP-seq
☐	☒ Flow cytometry
☐	☐ MRI-based neuroimaging

Antibodies

Antibodies used	N/A
Validation	N/A

Eukaryotic cell lines

Policy information about cell lines and Sex and Gender in Research

Cell line source(s)	N/A
Authentication	N/A
Mycoplasma contamination	N/A
Commonly misidentified lines (See ICLAC register)	N/A

Palaeontology and Archaeology

Specimen provenance	N/A
Specimen deposition	N/A
Dating methods	N/A

☐ Tick this box to confirm that the raw and calibrated dates are available in the paper or in Supplementary Information.

Ethics oversight	N/A

Note that full information on the approval of the study protocol must also be provided in the manuscript.

Animals and other research organisms

Policy information about studies involving animals; ARRIVE guidelines recommended for reporting animal research, and Sex and Gender in Research

Laboratory animals	N/A
Wild animals	N/A
Reporting on sex	N/A
Field-collected samples	N/A
Ethics oversight	N/A

Note that full information on the approval of the study protocol must also be provided in the manuscript.

Clinical data

Policy information about clinical studies

All manuscripts should comply with the ICMJE guidelines for publication of clinical research and a completed CONSORT checklist must be included with all submissions.

Clinical trial registration	N/A
Study protocol	N/A
Data collection	N/A
Outcomes	N/A

Dual use research of concern

Policy information about dual use research of concern

Hazards

Could the accidental, deliberate or reckless misuse of agents or technologies generated in the work, or the application of information presented in the manuscript, pose a threat to:

No	Yes	
☒	☐	Public health
☒	☐	National security
☒	☐	Crops and/or livestock
☒	☐	Ecosystems
☒	☐	Any other significant area

Experiments of concern

Does the work involve any of these experiments of concern:

No	Yes	
☒	☐	Demonstrate how to render a vaccine ineffective
☒	☐	Confer resistance to therapeutically useful antibiotics or antiviral agents
☒	☐	Enhance the virulence of a pathogen or render a nonpathogen virulent
☒	☐	Increase transmissibility of a pathogen
☒	☐	Alter the host range of a pathogen
☒	☐	Enable evasion of diagnostic/detection modalities
☒	☐	Enable the weaponization of a biological agent or toxin
☒	☐	Any other potentially harmful combination of experiments and agents

Plants

Seed stocks	N/A
Novel plant genotypes	N/A
Authentication	N/A

ChIP-seq

Data deposition

☐ Confirm that both raw and final processed data have been deposited in a public database such as GEO.

☐ Confirm that you have deposited or provided access to graph files (e.g. BED files) for the called peaks.

Data access links<br>*May remain private before publication.*	N/A
Files in database submission	N/A
Genome browser session<br>(e.g. UCSC)	N/A

Methodology

Replicates	N/A
Sequencing depth	N/A
Antibodies	N/A
Peak calling parameters	N/A
Data quality	N/A
Software	N/A

Flow Cytometry

Plots

Confirm that:

☒ The axis labels state the marker and fluorochrome used (e.g. CD4-FITC).

☒ The axis scales are clearly visible. Include numbers along axes only for bottom left plot of group (a 'group' is an analysis of identical markers).

☒ All plots are contour plots with outliers or pseudocolor plots.

☒ A numerical value for number of cells or percentage (with statistics) is provided.

Methodology

Sample preparation	Bacteria were labeled with a cyclooctyne probe and subjected to one or two rounds of strain-promoted azide-alkyne cycloaddition. After fixation, bacteria were then washed in PBST and resuspended either in saline solution (NaCl 0.9%) or in PBS and directly subjected to flow cytometry.
Instrument	BD DUAL LSRFortessa, 5 excitation lasers (355nm, 405nm, 488nm, 561nm, and 640nm), 18 color analysis capabilities. Optical configuration details: https://www.umass.edu/ials/sites/default/files/facilities/2021-Fortessa-Oplical-Configuration.pdf
Software	BD FACSDiva software.

Cell population abundance	We acquired from 10,000 to 30,000 events per sample.
Gating strategy	Bacteria were gated based on FSC-A vs SSC-A using the lab's standard settings for mycobacteria.

☒ Tick this box to confirm that a figure exemplifying the gating strategy is provided in the Supplementary Information.

Magnetic resonance imaging

Experimental design

Design type	N/A
Design specifications	N/A
Behavioral performance measures	N/A

Acquisition

Imaging type(s)	N/A
Field strength	N/A
Sequence & imaging parameters	N/A
Area of acquisition	N/A

Diffusion MRI ☐ Used ☐ Not used

Preprocessing

Preprocessing software	N/A
Normalization	N/A
Normalization template	N/A
Noise and artifact removal	N/A
Volume censoring	*Define your software and/or method and criteria for volume censoring, and state the extent of such censoring.*

Statistical modeling & inference

Model type and settings	N/A
Effect(s) tested	N/A

Specify type of analysis: ☐ Whole brain ☐ ROI-based ☐ Both

Statistic type for inference (See Eklund et al. 2016)	N/A
Correction	N/A

Models & analysis

n/a	Involved in the study
☒	☐ Functional and/or effective connectivity
☒	☐ Graph analysis
☒	☐ Multivariate modeling or predictive analysis
