# Supplementary material for: Identification of chemical features for improved outer membrane permeation in mycobacteria using machine learning
Source: Nat Microbiol. 2026 Jun 30;11(9):2647–65. doi: 10.1038/s41564-026-02412-5 (PMC13524814; doi:10.1038/s41564-026-02412-5)
Supplement: Supplementary file 3 [file 41564_2026_2412_MOESM3_ESM.pdf]

nature portfolio

Peer Review File

Identification of chemical features for improved outer membrane permeation in mycobacteria using machine learning

Corresponding Author: Professor M. Sloan Siegrist

Version 0:

Reviewer comments:

Reviewer #1

(Remarks to the Author)
The manuscript is well written and focused on an important problem. I enjoyed reading it. The authors leverage a previously developed assay to assess the permeability of ~1500 small molecules through the cell wall of Gram-positive mycobacteria. Their analysis identifies chemical moieties that correlate with permeability, which are then used to optimize a previously reported compound.

Acceptance of the manuscript should be contingent upon addressing the following points.

MAJOR COMMENTS
1. I would like to better understand the rationale behind selecting imidazole, pyrazole, pyrrolidine, and cyclopentane for the optimization of JSF-2985. While Figure 2A highlights these groups, other moieties (such as indole) appear to show even stronger positive correlation with permeability but were not chosen. Similarly, many groups exhibit strong negative correlation. Please clarify how the final selection was made, and why certain promising scaffolds were excluded.

2. On line 246, the manuscript states that a molecule with a positively correlated group permeates better than the cyclopentane derivative. However, for a meaningful comparison, one would expect the derivative to be compared to the parent (unmodified) compound. If the cyclopentane group correlates negatively with permeability, it should perform worse. Am I misinterpreting the statement? I recommend showing dose–response curves for each derivative directly compared to the original molecule in Figure 4F. The current comparison against beads is informative, but this data could be moved to the Supplementary Information.

3. Figure 3B suggests the ML model predicts residuals well, but I am concerned it may simply be memorizing the data (i.e., overfitting) rather than learning meaningful structure–activity relationships. To better evaluate generalization, I recommend the authors repeat the ML analysis using their 80-10-10 train-validation-test splits, with the test set consisting of compounds that are significantly dissimilar (e.g., by Tanimoto distance) from those in the training set. One way to achieve this is by scaffold-based splitting. The performance (e.g., ρ, R^2) should be reported as mean ± SEM across multiple splits. If predictive performance under these conditions is poor, Figures 3C, 3E, and 3F should be toned down (or removed). That said, even in the absence of predictive value, the ML model may still serve as a useful tool to summarize structure–permeability relationships within the dataset. However, in this case, its role would overlap substantially with the chemoinformatics analysis, and this redundancy should be acknowledged.

MINOR COMMENTS
- In Figure 1A, the test molecule is represented by a large “R” (yes?) labeled “test azide,” presumably indicating it is bound to an N_3 group for TetD labeling. I assume “R” stands for “residue,” but this is not clearly stated in the figure or legend, and took some effort to deduce. Please clarify this notation explicitly.

- The assay readout is “competition,” which reflects both permeability and reactivity. The manuscript would benefit from a more explicit discussion of whether these two components can be disentangled. Are the authors primarily interested in permeability? If so, how do they control for reactivity effects?

- I would appreciate efforts to improve the clarity of Figure 2. The caption states that the scaffolds shown in panels A and B correspond to those listed on the y-axis of panel C, but there is no clear mapping between scaffold names and their chemical structures—making it difficult to identify, for example, which structure represents indole. One might assume the scaffolds are

presented in the same order across panels, but this is made more confusing by inconsistencies in the compound counts. For instance, indole is reported to have 28 compounds in panel A but only 26 in panel C. I understand that panel B may contain fewer compounds due to selection criteria, but shouldn't panels A and C include the same number of compounds per scaffold? Please clarify both the ordering and the source of these discrepancies.

- A natural question arises as to whether the azide modification affects antibacterial activity. Azides can be reactive and potentially alter compound potency. The discussion touches on this, but further clarification would be valuable. Ideally, the authors could provide dose–response curves for the azide-tagged versus parent compounds in at least one Mycobacterium strain. MIC or IC_{50} values would help clarify this point.

(Remarks on code availability)

Reviewer #2

(Remarks to the Author)
In this work Lepori, Liu, Evbarunegbe, Feng et al. present the most comprehensive report to date on the application of their permeability assay PAC-MAN, which uses covalent trapping of azide test compounds by alkynes metabolically incorporated into peptidoglycan, to the determination of small molecule accumulation in mycobacteria, with a focus on M. tuberculosis (Mtb). Building on their recent work examining chemical features that modulate dye, antibiotic and peptide accumulation in the cell wall, the authors here present the screening of a far larger library of azide test compounds (>1500) and use these results to develop a predictive model.
This work stands apart from very recently reported complementary work on antibiotic accumulation in M. abscessus due to its far higher degree of generalizability and applicability. Crucially, the authors identify and validate chemical features that (dis)favor accumulation in their assay and show via the analysis of existing (non-azide-containing) datasets and their own test compound SAR series that these relationships correlate with whole-cell activity. This is a revolutionary first for a field that has a history of target-based inhibitors failing to yield whole-cell activity and inhibitor optimization based on unvalidated assumptions regarding chemical features that promote permeation. This study provides among the first guidance that is based on experimental evidence; importantly, it also introduces and emphasizes accessible tools in the publicly available MycoPermeNet predictive model and the PACMAN assay, which, although it requires azide-modified compounds, is still on the whole more tractable than more widely reported but far more expensive mass spectrometry methods.
The study is especially commendable for its comprehensiveness and rigor and the strong dialogue between experiment and modeling. For example, the authors provide quantitative comparisons to analogous studies in E. coli and P. aeruginosa (showing that they do not predict accumulation in Mtb) and a negative control for their predictions by using data from not only from whole-cell screens, but also enzyme target-based screens where permeation is irrelevant. They also have the crucial insight to segment their results by chemical context (i.e., scaffold) rather than only analyzing the entire set for physicochemical correlates, as is more widely done. This makes their findings far more specific and useful with respect to chemical changes that promote permeation, as demonstrated in their two small molecule series highlighting Trp-Phe substitution and the introduction of nitrogen-containing heterocycles. Overall, the combination of informed (myco)bacterial perspective and deep chemical understanding makes this study an exemplar in this burgeoning area of predictive modeling for antibiotic development and optimization; we can only hope that similar teams come together to tackle related areas. Only a very few, very minor comments on a well written and presented manuscript that is set to be landmark study in the field.
MINOR COMMENTS
General comment: For the non-expert the coverage of the computation analysis and model building may be especially difficult to navigate and could benefit from more explanatory context, although we recognize that there may not be space to explain terms and provide background, in terms of norms that dictate methodological choices.
Line 152: "When analyzed as a whole, our Mtb dataset did not reveal obvious correlations." The authors generally also summarize their results on Msm throughout, but not here. Was the same true for the Msm dataset?
Line 317-2138: "PAC-MAN primarily measures mycomembrane permeation, e.g., passive diffusion and facilitated transport" It is not clear from the cited studies what the evidence is that other processes (plasma membrane transport, as yet unknown mechanisms of mycomembrane export) do not also contribute or, at least, what assumptions are made by this statement (e.g., that chemical reactivity is appreciably faster than any process by which the test compound would exit the cell wall). Could the authors please clarify?

(Remarks on code availability)

Reviewer #3

(Remarks to the Author)
This manuscript by Lepori et al takes on an important problem, that of trying to discern what chemical features might allow a would-be antibiotic to permeate into Mtb. Given the many differences between the membranes of Mtb and other bacteria, it is critical to determine Mtb-specific permeation traits.

I have read this manuscript closely and carefully considered its message. I do think the long-term goal is highly meritorious, and the work could potentially be of high interest. As of right now, however, there are questions about the method and what it is reading out. And more critically, there is no real conclusion about chemical traits and permeation in Mtb (for example, neither the abstract or discussion mentions anything that could guide drug development). The manuscript indeed is much more computationally oriented and in its present form would be better suited to a computational journal. Modification for Nature Microbiology would require significant re-thinking and re-casting of this work, and further experiments. Specific comments:

1) The hypothesis that Mtb permeation is a key determinant of whole cell anti-Mtb activity (mentioned a couple times) is self-evident and strikes as a straw man.
2) Mtb is well known to process would-be antibiotics as they make their way into the cell, so it is striking that there is no mention of this possibility of drug modification and how it might complicate the interpretation of their results.
3) A strength of this work is the ability to evaluate a multitude of different looking compounds, since the authors are untethered from antibacterial activity.
4) The authors seem to have done quite a bit of validation of their permeation competition assay. The biggest challenge with using this assay to draw conclusions is that positive compounds covalently append themselves to the bacteria, so, the natural cycle of in/out that would occur for (non-covalent) antibiotics is bypassed. This assay can then be viewed as good for assessing penetration, but less good (and perhaps poor) for truly assessing accumulation, which is the crux of the drug discovery problem. The authors do mention this caveat in the Discussion; but, it is a big one.
5) A weakness in the ML workflow is on the bottom of page 12, hand selection of 26 chemical features. This is a small fraction of the features that are available.
6) For Figure 4, testing within a series, it is hard to know how meaningful the differences are in terms a microbiologist/drug researcher might understand. What is the cellular concentration difference between a compound with a deltaLogCC50 of 1.25 and one with a value of 2 (for example)?
7) A major weakness is not validating any of the results with the LC/MS/MS method which is now standard in the field.

Overall, the authors present an interesting workflow for studying penetrance into Mtb. But, it is hard to make heads or tails of the data. It is hard to know what has been learned, perhaps best evident by the fact that the authors have not been able to apply any of the learnings to convincingly lead to compounds that accumulate better in Mtb and have better antibacterial activity.

(Remarks on code availability)

Decision Letter:

16th May 2025

*Please ensure you delete the link to your author homepage in this e-mail if you wish to forward it to your co-authors.

Dear Sloan,

Thank you very much for your patience while your manuscript "Identification of chemical features that influence mycomembrane permeation and antitubercular activity" was under peer-review at Nature Microbiology. It has now been seen by 3 referees, whose expertise and comments you will find at the end of this email. Although they find your work of some potential interest, they have raised a number of concerns that will need to be addressed before we can consider publication of the work in Nature Microbiology.

In particular, several of the referees raised a few concerns about aspects of the machine learning model applied. Referee #3 noted that some features were hand selected and had queries about this, while Referee #1 importantly noted that some additional analyses are needed to more fully demonstrate that the model has predictive function and has learned structure-function relationships. There were also requests for further validation, including dose-response curves for parent versus modified compounds, to more clearly demonstrate improved penetration with given scaffolds, as well as LC-MS/MS to validate some findings. Referee #1 also asked for more explanation as to why certain moieties were followed up and others not, and had doubts over whether the azide modification might influence molecule characteristics. The referees generally also noted that some reworking of the manuscript text and figures would be needed to make this more accessible for a more microbiology-focussed audience who may not be expert in some of the computational approaches used. In light of Referee #3's comments, we also felt it would be important for the manscript to better communicate what the key take home messages are for molecular scaffolds that confer better penetration capabilities, and if possible, to further explore or demonstrate how application of these principles can lead to better penetration/antimicrobial activity. We realise that this may be complicated, but think it would really strengthen the manuscript and emphasise the significant advance this approach and findings potentially provide.

Should further experimental data allow you to address these criticisms, we would be happy to look at a revised manuscript.

We are committed to providing a fair and constructive peer-review process. Please do not hesitate to contact us if there are specific requests from the reviewers that you believe are technically impossible or unlikely to yield a meaningful outcome.

We strongly support public availability of data. Please place the data used in your paper into a public data repository, if one exists, or alternatively, present the data as Source Data or Supplementary Information. If data can only be shared on request, please explain why in your Data Availability Statement, and also in the correspondence with your editor. For some data types, deposition in a public repository is mandatory - more information on our data deposition policies and available repositories can be found at https://www.nature.com/nature-research/editorial-policies/reporting-standards#availability-of-data.

Please include a data availability statement as a separate section after Methods but before references, under the heading "Data Availability". This section should inform readers about the availability of the data used to support the conclusions of your study. This information includes accession codes to public repositories (data banks for protein, DNA or RNA sequences, microarray, proteomics data etc…), references to source data published alongside the paper, unique identifiers such as URLs to data repository entries, or data set DOIs, and any other statement about data availability. At a minimum, you should include the

following statement: “The data that support the findings of this study are available from the corresponding author upon request”, mentioning any restrictions on availability. If DOIs are provided, we also strongly encourage including these in the Reference list (authors, title, publisher (repository name), identifier, year). For more guidance on how to write this section please see: http://www.nature.com/authors/policies/data/data-availability-statements-data-citations.pdf

If revising your manuscript:

* Include a “Response to referees” document detailing, point-by-point, how you addressed each referee comment. If no action was taken to address a point, you must provide a compelling argument. This response will be sent back to the referees along with the revised manuscript.

* If you have not done so already we suggest that you begin to revise your manuscript so that it conforms to our Article format instructions at http://www.nature.com/nmicrobiol/info/final-submission. Refer also to any guidelines provided in this letter.

* Include a revised version of any required reporting checklist. It will be available to referees (and, potentially, statisticians) to aid in their evaluation if the manuscript goes back for peer review. A revised checklist is essential for re-review of the paper.

When submitting the revised version of your manuscript, please pay close attention to our href="https://www.nature.com/nature-portfolio/editorial-policies/image-integrity">Digital Image Integrity Guidelines. and to the following points below:

-- that unprocessed scans are clearly labelled and match the gels and western blots presented in figures.
-- that control panels for gels and western blots are appropriately described as loading on sample processing controls
-- all images in the paper are checked for duplication of panels and for splicing of gel lanes.

EXTENDED DATA FIGURES

When re-submitting your manuscript, please ensure that any supplementary figures and tables that are crucial to the manuscript’s conclusions are converted into Extended Data figures and tables to increase visibility of these data. Extended Data figures and tables are online-only (present in the online PDF and full-text HTML versions of the paper), peer-reviewed display items that provide essential background to the article but are not included in the main article due to space constraints. A maximum of ten Extended Data display items (figures and tables) is permitted.

Finally, please ensure that you retain unprocessed data and metadata files after publication, ideally archiving data in perpetuity, as these may be requested during the peer review and production process or after publication if any issues arise.

Please use the link below to submit a revised paper:

Link Redacted

Note: This url links to your confidential homepage and associated information about manuscripts you may have submitted or be reviewing for us. If you wish to forward this e-mail to co-authors, please delete this link to your homepage first.

Nature Microbiology is committed to improving transparency in authorship. As part of our efforts in this direction, we are now requesting that all authors identified as ‘corresponding author’ on published papers create and link their Open Researcher and Contributor Identifier (ORCID) with their account on the Manuscript Tracking System (MTS), prior to acceptance. This applies to primary research papers only. ORCID helps the scientific community achieve unambiguous attribution of all scholarly contributions. You can create and link your ORCID from the home page of the MTS by clicking on ‘Modify my Springer Nature account’. For more information please visit please visit www.springernature.com/orcid.

If you wish to submit a suitably revised manuscript we would hope to receive it within 6 months. If you cannot send it within this time, please let us know. We will be happy to consider your revision, even if a similar study has been accepted for publication at Nature Microbiology or published elsewhere (up to a maximum of 6 months).

In the meantime we hope that you find our referees' comments helpful.

Yours sincerely,

****
Reviewer Expertise:

Referee #1: AI/ML, drug discovery
Referee #2: mycobacteria, membrane biology
Referee #3: chemistry, antibiotic development, antibiotic accumulation

Reviewer Comments:

Reviewer #1 (Remarks to the Author):

The manuscript is well written and focused on an important problem. I enjoyed reading it. The authors leverage a previously developed assay to assess the permeability of ~1500 small molecules through the cell wall of Gram-positive mycobacteria. Their analysis identifies chemical moieties that correlate with permeability, which are then used to optimize a previously reported compound.

Acceptance of the manuscript should be contingent upon addressing the following points.

MAJOR COMMENTS
1. I would like to better understand the rationale behind selecting imidazole, pyrazole, pyrrolidine, and cyclopentane for the optimization of JSF-2985. While Figure 2A highlights these groups, other moieties (such as indole) appear to show even stronger positive correlation with permeability but were not chosen. Similarly, many groups exhibit strong negative correlation. Please clarify how the final selection was made, and why certain promising scaffolds were excluded.

2. On line 246, the manuscript states that a molecule with a positively correlated group permeates better than the cyclopentane derivative. However, for a meaningful comparison, one would expect the derivative to be compared to the parent (unmodified) compound. If the cyclopentane group correlates negatively with permeability, it should perform worse. Am I misinterpreting the statement? I recommend showing dose–response curves for each derivative directly compared to the original molecule in Figure 4F. The current comparison against beads is informative, but this data could be moved to the Supplementary Information.

3. Figure 3B suggests the ML model predicts residuals well, but I am concerned it may simply be memorizing the data (i.e., overfitting) rather than learning meaningful structure–activity relationships. To better evaluate generalization, I recommend the authors repeat the ML analysis using their 80-10-10 train-validation-test splits, with the test set consisting of compounds that are significantly dissimilar (e.g., by Tanimoto distance) from those in the training set. One way to achieve this is by scaffold-based splitting. The performance (e.g., ρ, R^2) should be reported as mean ± SEM across multiple splits. If predictive performance under these conditions is poor, Figures 3C, 3E, and 3F should be toned down (or removed). That said, even in the absence of predictive value, the ML model may still serve as a useful tool to summarize structure–permeability relationships within the dataset. However, in this case, its role would overlap substantially with the chemoinformatics analysis, and this redundancy should be acknowledged.

MINOR COMMENTS
- In Figure 1A, the test molecule is represented by a large “R” (yes?) labeled “test azide,” presumably indicating it is bound to an N_3 group for TetD labeling. I assume “R” stands for “residue,” but this is not clearly stated in the figure or legend, and took some effort to deduce. Please clarify this notation explicitly.

- The assay readout is “competition,” which reflects both permeability and reactivity. The manuscript would benefit from a more explicit discussion of whether these two components can be disentangled. Are the authors primarily interested in permeability? If so, how do they control for reactivity effects?

- I would appreciate efforts to improve the clarity of Figure 2. The caption states that the scaffolds shown in panels A and B correspond to those listed on the y-axis of panel C, but there is no clear mapping between scaffold names and their chemical structures—making it difficult to identify, for example, which structure represents indole. One might assume the scaffolds are presented in the same order across panels, but this is made more confusing by inconsistencies in the compound counts. For instance, indole is reported to have 28 compounds in panel A but only 26 in panel C. I understand that panel B may contain fewer compounds due to selection criteria, but shouldn’t panels A and C include the same number of compounds per scaffold? Please clarify both the ordering and the source of these discrepancies.

- A natural question arises as to whether the azide modification affects antibacterial activity. Azides can be reactive and potentially alter compound potency. The discussion touches on this, but further clarification would be valuable. Ideally, the authors could provide dose–response curves for the azide-tagged versus parent compounds in at least one Mycobacterium strain. MIC or IC_{50} values would help clarify this point.

Reviewer #2 (Remarks to the Author):

In this work Lepori, Liu, Evbarunegbe, Feng et al. present the most comprehensive report to date on the application of their permeability assay PAC-MAN, which uses covalent trapping of azide test compounds by alkynes metabolically incorporated into peptidoglycan, to the determination of small molecule accumulation in mycobacteria, with a focus on M. tuberculosis (Mtb). Building on their recent work examining chemical features that modulate dye, antibiotic and peptide accumulation in the cell wall, the authors here present the screening of a far larger library of azide test compounds (>1500) and use these results to develop a predictive model.
This work stands apart from very recently reported complementary work on antibiotic accumulation in M. abscessus due to its far higher degree of generalizability and applicability. Crucially, the authors identify and validate chemical features that (dis)favor accumulation in their assay and show via the analysis of existing (non-azide-containing) datasets and their own test compound

SAR series that these relationships correlate with whole-cell activity. This is a revolutionary first for a field that has a history of target-based inhibitors failing to yield whole-cell activity and inhibitor optimization based on unvalidated assumptions regarding chemical features that promote permeation. This study provides among the first guidance that is based on experimental evidence; importantly, it also introduces and emphasizes accessible tools in the publicly available MycoPermeNet predictive model and the PACMAN assay, which, although it requires azide-modified compounds, is still on the whole more tractable than more widely reported but far more expensive mass spectrometry methods.

The study is especially commendable for its comprehensiveness and rigor and the strong dialogue between experiment and modeling. For example, the authors provide quantitative comparisons to analogous studies in E. coli and P. aeruginosa (showing that they do not predict accumulation in Mtb) and a negative control for their predictions by using data from not only from whole-cell screens, but also enzyme target-based screens where permeation is irrelevant. They also have the crucial insight to segment their results by chemical context (i.e., scaffold) rather than only analyzing the entire set for physicochemical correlates, as is more widely done. This makes their findings far more specific and useful with respect to chemical changes that promote permeation, as demonstrated in their two small molecule series highlighting Trp-Phe substitution and the introduction of nitrogen-containing heterocycles. Overall, the combination of informed (myco)bacterial perspective and deep chemical understanding makes this study an exemplar in this burgeoning area of predictive modeling for antibiotic development and optimization; we can only hope that similar teams come together to tackle related areas. Only a very few, very minor comments on a well written and presented manuscript that is set to be landmark study in the field.

MINOR COMMENTS

General comment: For the non-expert the coverage of the computation analysis and model building may be especially difficult to navigate and could benefit from more explanatory context, although we recognize that there may not be space to explain terms and provide background, in terms of norms that dictate methodological choices.

Line 152: “When analyzed as a whole, our Mtb dataset did not reveal obvious correlations.” The authors generally also summarize their results on Msm throughout, but not here. Was the same true for the Msm dataset?

Line 317-2138: “PAC-MAN primarily measures mycomembrane permeation, e.g., passive diffusion and facilitated transport” It is not clear from the cited studies what the evidence is that other processes (plasma membrane transport, as yet unknown mechanisms of mycomembrane export) do not also contribute or, at least, what assumptions are made by this statement (e.g., that chemical reactivity is appreciably faster than any process by which the test compound would exit the cell wall). Could the authors please clarify?

Reviewer #3 (Remarks to the Author):

This manuscript by Lepori et al takes on an important problem, that of trying to discern what chemical features might allow a would-be antibiotic to permeate into Mtb. Given the many differences between the membranes of Mtb and other bacteria, it is critical to determine Mtb-specific permeation traits.

I have read this manuscript closely and carefully considered its message. I do think the long-term goal is highly meritorious, and the work could potentially be of high interest. As of right now, however, there are questions about the method and what it is reading out. And more critically, there is no real conclusion about chemical traits and permeation in Mtb (for example, neither the abstract or discussion mentions anything that could guide drug development). The manuscript indeed is much more computationally oriented and in its present form would be better suited to a computational journal. Modification for Nature Microbiology would require significant re-thinking and re-casting of this work, and further experiments. Specific comments:

1) The hypothesis that Mtb permeation is a key determinant of whole cell anti-Mtb activity (mentioned a couple times) is self-evident and strikes as a straw man.
2) Mtb is well known to process would-be antibiotics as they make their way into the cell, so it is striking that there is no mention of this possibility of drug modification and how it might complicate the interpretation of their results.
3) A strength of this work is the ability to evaluate a multitude of different looking compounds, since the authors are untethered from antibacterial activity.
4) The authors seem to have done quite a bit of validation of their permeation competition assay. The biggest challenge with using this assay to draw conclusions is that positive compounds covalently append themselves to the bacteria, so, the natural cycle of in/out that would occur for (non-covalent) antibiotics is bypassed. This assay can then be viewed as good for assessing penetration, but less good (and perhaps poor) for truly assessing accumulation, which is the crux of the drug discovery problem. The authors do mention this caveat in the Discussion; but, it is a big one.
5) A weakness in the ML workflow is on the bottom of page 12, hand selection of 26 chemical features. This is a small fraction of the features that are available.
6) For Figure 4, testing within a series, it is hard to know how meaningful the differences are in terms a microbiologist/drug researcher might understand. What is the cellular concentration difference between a compound with a deltaLogCC50 of 1.25 and one with a value of 2 (for example)?
7) A major weakness is not validating any of the results with the LC/MS/MS method which is now standard in the field.

Overall, the authors present an interesting workflow for studying penetrance into Mtb. But, it is hard to make heads or tails of the data. It is hard to know what has been learned, perhaps best evident by the fact that the authors have not been able to apply any of the learnings to convincingly lead to compounds that accumulate better in Mtb and have better antibacterial activity.

****

Version 1:

Reviewer comments:

Reviewer #2

(Remarks to the Author)
All of our minor comments were addressed. We look forward to seeing this work finally published as is well deserved.

(Remarks on code availability)

Reviewer #3

(Remarks to the Author)
There are many improvements in this new version of the manuscript. It is a good start toward getting at the vexing Mtb permeation/accumulation challenge. There are still weaknesses – most notably the lack of any mechanistic basis for the effect of indoles and related moieties, and the lack of a convincing demonstration of this discovery in the generation of a novel antibiotic. A lot to ask, for sure, but will ultimately be needed for these observations to be widely adopted by the field.

That said, I do not wish to stand in the way of this study as there is lots of important info here. The impact of this work will be much higher if the authors keep their target audience (drug discoverers / microbiologiests) in mind, especially in the presentation of their data. A few specifics:

1) Many depictions of the data are non-intuitive and non-standard, so it is hard to understand some of the key effects and their meaning. The deltaLog10CC50, in particular, is puzzling. Is the decrease of this from ~1.75 to ~1.00 (as in Figure 4c and 4g) considered big? What are these values for high accumulating and low accumulating control compounds? This would be helpful.

2) Related, why are there not error bars and p-values for these experimental plots (Figure 4c and 4g), but there are error bars and p-values for the computational plots (4d and 4h)? Again, this is a puzzle to the non-computationalist.

3) Similar theme, Figure 6 talks about whole cell activity, but there is nothing in this figure that is familiar to the microbiologist. Why not express whole cell activity as an MIC value? A simple, easy-to-understand figure would be powerful: showing that addition of an indole (for example) takes a compound from one that is non-accumulating and inactive against Mtb, to one that now accumulates and is active against Mtb.

(Remarks on code availability)

Reviewer #4

(Remarks to the Author)
I think the focus of the study is important. Understanding the molecular features that govern intracellular accumulation is essential to make antibiotic discovery and optimization more efficient.

Given that the algorithmic methods are not new, there is not much to comment on for these parts of the paper. One minor comment here would be that with the relatively small size of their training dataset, I wonder whether a simpler binary classification method would have been more useful than a regressor; I do not expect the authors to address this at the current stage. Moreover, I'm unconvinced that their Chemprop model can generalize without being prospectively applied to a (somewhat) out of distribution chemical set. Again, I'm unsure whether this needs to be addressed for this study.

I do have one major comment for the authors to address. In my opinion, the most important contribution of modeling intracellular accumulation is to improve antibacterial potency, most commonly defined using MIC. However, as the authors note, ML-guided modification of JSF-2985 did not improve activity (it looks like it lost activity). Similarly, from what I can see from Figure 5, the increases in OctTri potency after modification are modest. In my opinion, this speaks to the difficulty of identifying "permeability-enabling" subgroups without considering whole cell antibacterial activity in parallel. I don't think that this is necessarily disqualifying for the study, but it is an essential observation to convey clearly to the field and a shortcoming of this study.

I think that the authors need to add a set of growth inhibition curves (OD as a function of concentration) to the main text figures, showing (a) the starting "parent" molecule and (b) the ML-guided modified analogs. This is essential to depict clearly, to highlight the disconnect between predicted (or experimentally validated) envelope permeability and whole cell antibacterial potency, which is what's being chased this this type of work.

(Remarks on code availability)

Decision Letter:

17th February 2026

*Please ensure you delete the link to your author homepage in this e-mail if you wish to forward it to your co-authors.

Dear Professor Siegrist,

Thank you for your patience while your manuscript "Identification of chemical features that influence mycomembrane permeation and antitubercular activity" was under peer-review at Nature Microbiology.

It has now been seen by 3 referees, whose expertise and comments you will find at the end of this email. Unfortunately referee #2 was unavailable to look at this again and so we recruited a new referee with overlapping expertise to assess your rebuttal (referee #4). You will see from their comments below that while they find your work of interest, some important points are raised. We are very interested in the possibility of publishing your study in Nature Microbiology, but would like to consider your response to these concerns in the form of a revised manuscript before we make a final decision on publication.

In particular, you will see that some additional text edits are required to tone down claims in line with referee comments. In line with these comments we also feel that this study fits our 'Resource' format given the potential utility of the dataset. The formatting is very similar to Articles. The rest referees' reports are clear and the remaining issues should be straightforward to address.

We are committed to providing a fair and constructive peer-review process. Do not hesitate to contact us if there are specific requests from the reviewers that you believe are technically impossible or unlikely to yield a meaningful outcome.

If you have not done so already please begin to revise your manuscript so that it conforms to our Resource format instructions at http://www.nature.com/nmicrobiol/info/final-submission/

The usual length limit for a Nature Microbiology Article is six display items (figures or tables) and 3,000 words. We have some flexibility, and can allow a revised manuscript at 3,500 words, but please consider this a firm upper limit. There is a trade-off of ~250 words per display item, so if you need more space, you could move a Figure or Table to Supplementary Information.

Some reduction could be achieved by focusing any introductory material and moving it to the start of your opening 'bold' paragraph, whose function is to outline the background to your work, describe in a sentence your new observations, and explain your main conclusions. The discussion should also be limited. Methods should be described in a separate section following the discussion, we do not place a word limit on Methods.

Nature Microbiology titles should give a sense of the main new findings of a manuscript, and should not contain punctuation. Please keep in mind that we strongly discourage active verbs in titles, and that they should ideally fit within 90 characters each (including spaces).

We strongly support public availability of data. Please place the data used in your paper into a public data repository, if one exists, or alternatively, present the data as Source Data or Supplementary Information. If data can only be shared on request, please explain why in your Data Availability Statement, and also in the correspondence with your editor. For some data types, deposition in a public repository is mandatory - more information on our data deposition policies and available repositories can be found at https://www.nature.com/nature-research/editorial-policies/reporting-standards#availability-of-data.

Please include a data availability statement as a separate section after Methods but before references, under the heading "Data Availability". This section should inform readers about the availability of the data used to support the conclusions of your study. This information includes accession codes to public repositories (data banks for protein, DNA or RNA sequences, microarray, proteomics data etc…), references to source data published alongside the paper, unique identifiers such as URLs to data repository entries, or data set DOIs, and any other statement about data availability. At a minimum, you should include the following statement: "The data that support the findings of this study are available from the corresponding author upon request", mentioning any restrictions on availability. If DOIs are provided, we also strongly encourage including these in the Reference list (authors, title, publisher (repository name), identifier, year). For more guidance on how to write this section please see: http://www.nature.com/authors/policies/data/data-availability-statements-data-citations.pdf

To improve the accessibility of your paper to readers from other research areas, please pay particular attention to the wording of the paper's opening bold paragraph, which serves both as an introduction and as a brief, non-technical summary in about 150 words. If, however, you require one or two extra sentences to explain your work clearly, please include them even if the paragraph is over-length as a result. The opening paragraph should not contain references. Because scientists from other sub-disciplines will be interested in your results and their implications, it is important to explain essential but specialised terms concisely. We suggest you show your summary paragraph to colleagues in other fields to uncover any problematic concepts.

If your paper is accepted for publication, we will edit your display items electronically so they conform to our house style and will reproduce clearly in print. If necessary, we will re-size figures to fit single or double column width. If your figures contain several parts, the parts should form a neat rectangle when assembled. Choosing the right electronic format at this stage will speed up the processing of your paper and give the best possible results in print. We would like the figures to be supplied as vector files - EPS, PDF, AI or postscript (PS) file formats (not raster or bitmap files), preferably generated with vector-graphics software (Adobe Illustrator for example). Please try to ensure that all figures are non-flattened and fully editable. All images should be at least 300 dpi resolution (when figures are scaled to approximately the size that they are to be printed at) and in RGB colour format. Please do not submit Jpeg or flattened TIFF files. Please see also 'Guidelines for Electronic Submission of Figures' at the end of this letter for further detail.

Figure legends must provide a brief description of the figure and the symbols used, within 350 words, including definitions of any error bars employed in the figures.

When submitting the revised version of your manuscript, please pay close attention to our href="https://www.nature.com/nature-research/editorial-policies/image-integrity">Digital Image Integrity Guidelines. and to the following points below:

-- that unprocessed scans are clearly labelled and match the gels and western blots presented in figures.
-- that control panels for gels and western blots are appropriately described as loading on sample processing controls
-- all images in the paper are checked for duplication of panels and for splicing of gel lanes.

EXTENDED DATA FIGURES

When re-submitting your manuscript, please ensure that any supplementary figures and tables that are crucial to the manuscript's conclusions are converted into Extended Data figures and tables to increase visibility of these data. Extended Data figures and tables are online-only (present in the online PDF and full-text HTML versions of the paper), peer-reviewed display items that provide essential background to the article but are not included in the main article due to space constraints. A maximum of ten Extended Data display items (figures and tables) is permitted.

Finally, please ensure that you retain unprocessed data and metadata files after publication, ideally archiving data in perpetuity, as these may be requested during the peer review and production process or after publication if any issues arise.

Please include a statement before the acknowledgements naming the author to whom correspondence and requests for materials should be addressed.

Finally, we require authors to include a statement of their individual contributions to the paper -- such as experimental work, project planning, data analysis, etc. -- immediately after the acknowledgements. The statement should be short, and refer to authors by their initials. For details please see the Authorship section of our joint Editorial policies at http://www.nature.com/authors/editorial_policies/authorship.html

When revising your paper:

* include a point-by-point response to any editorial suggestions and to our referees. Please include your response to the editorial suggestions in your cover letter, and please upload your response to the referees as a separate document.

* ensure it complies with our format requirements for Letters as set out in our guide to authors at www.nature.com/nmicrobiol/info/gta/

* state in a cover note the length of the text, methods and legends; the number of references; number and estimated final size of figures and tables

* resubmit electronically if possible using the link below to access your home page:

Link Redacted

*This url links to your confidential homepage and associated information about manuscripts you may have submitted or be reviewing for us. If you wish to forward this e-mail to co-authors, please delete this link to your homepage first.

Please ensure that all correspondence is marked with your Nature Microbiology reference number in the subject line.

Nature Microbiology is committed to improving transparency in authorship. As part of our efforts in this direction, we are now requesting that all authors identified as 'corresponding author' on published papers create and link their Open Researcher and Contributor Identifier (ORCID) with their account on the Manuscript Tracking System (MTS), prior to acceptance. This applies to primary research papers only. ORCID helps the scientific community achieve unambiguous attribution of all scholarly contributions. You can create and link your ORCID from the home page of the MTS by clicking on 'Modify my Springer Nature account'. For more information please visit please visit www.springernature.com/orcid.

We hope to receive your revised paper within three weeks. If you cannot send it within this time, please let us know.

We look forward to hearing from you soon.

Yours sincerely,

****

Reviewers Comments:

Reviewer #2 (Remarks to the Author):

All of our minor comments were addressed. We look forward to seeing this work finally published as is well deserved.

Reviewer #3 (Remarks to the Author):

There are many improvements in this new version of the manuscript. It is a good start toward getting at the vexing Mtb permeation/accumulation challenge. There are still weaknesses – most notably the lack of any mechanistic basis for the effect of indoles and related moieties, and the lack of a convincing demonstration of this discovery in the generation of a novel antibiotic. A lot to ask, for sure, but will ultimately be needed for these observations to be widely adopted by the field.

That said, I do not wish to stand in the way of this study as there is lots of important info here. The impact of this work will be much higher if the authors keep their target audience (drug discoverers / microbiologiests) in mind, especially in the presentation of their data. A few specifics:

1) Many depictions of the data are non-intuitive and non-standard, so it is hard to understand some of the key effects and their meaning. The deltaLog10CC50, in particular, is puzzling. Is the decrease of this from ~1.75 to ~1.00 (as in Figure 4c and 4g) considered big? What are these values for high accumulating and low accumulating control compounds? This would be helpful.

2) Related, why are there not error bars and p-values for these experimental plots (Figure 4c and 4g), but there are error bars and p-values for the computational plots (4d and 4h)? Again, this is a puzzle to the non-computationalist.

3) Similar theme, Figure 6 talks about whole cell activity, but there is nothing in this figure that is familiar to the microbiologist. Why not express whole cell activity as an MIC value? A simple, easy-to-understand figure would be powerful: showing that addition of an indole (for example) takes a compound from one that is non-accumulating and inactive against Mtb, to one that now accumulates and is active against Mtb.

Reviewer #4 (Remarks to the Author):

I think the focus of the study is important. Understanding the molecular features that govern intracellular accumulation is essential to make antibiotic discovery and optimization more efficient.

Given that the algorithmic methods are not new, there is not much to comment on for these parts of the paper. One minor comment here would be that with the relatively small size of their training dataset, I wonder whether a simpler binary classification method would have been more useful than a regressor; I do not expect the authors to address this at the current stage. Moreover, I'm unconvinced that their Chemprop model can generalize without being prospectively applied to a (somewhat) out of distribution chemical set. Again, I'm unsure whether this needs to be addressed for this study.

I do have one major comment for the authors to address. In my opinion, the most important contribution of modeling intracellular accumulation is to improve antibacterial potency, most commonly defined using MIC. However, as the authors note, ML-guided modification of JSF-2985 did not improve activity (it looks like it lost activity). Similarly, from what I can see from Figure 5, the increases in OctTri potency after modification are modest. In my opinion, this speaks to the difficulty of identifying "permeability-enabling" subgroups without considering whole cell antibacterial activity in parallel. I don't think that this is necessarily disqualifying for the study, but it is an essential observation to convey clearly to the field and a shortcoming of this study.

I think that the authors need to add a set of growth inhibition curves (OD as a function of concentration) to the main text figures, showing (a) the starting "parent" molecule and (b) the ML-guided modified analogs. This is essential to depict clearly, to highlight the disconnect between predicted (or experimentally validated) envelope permeability and whole cell antibacterial potency, which is what's being chased this this type of work.

****

Version 2:

Reviewer comments:

Reviewer #3

(Remarks to the Author)
This resubmission provides some clarity and is suitable for publication. There is lots of work here and it is a very important problem. That said, most of the methods are non-standard (for accumulation, inhibition of growth, etc) so readers will largely be unable to meaningfully scrutinize and understand these new methods, all the more reason that it will be important for the authors to demonstrate -- in future papers -- the actionability of their discoveries to the creation of novel antibiotics against TB.

(Remarks on code availability)

Reviewer #4

(Remarks to the Author)
I appreciate the additional experiments and text revisions made during this round of revisions. I have a few comments that should be addressed. Notably, I do not want to impede the publication of this study because it's a useful contribution to the field, but there are some claims that are beyond what I think the data directly supports.

1. In their responses, the authors state "The lack of correlation between mycomembrane permeation and growth inhibition for JSF-2985 suggested that the mycomembrane might not be an important barrier to its whole cell activity." However, in the manuscript text, they also state "Here, we found that JSF-2985 derivatives with imidazole, pyrazole, and pyrrolidine scaffolds permeate the mycomembrane better than the JSF-2985 derivative bearing cyclopentane, consistent with both cheminformatics analyses and MycoPermeNet predictions." My interpretation to these statements is that it is possible to improve JSF-2985 permeability, but doing so also can decrease its inherent antibacterial activity. I think this needs to be stated far more clearly. Simply increasing permeability is not a guaranteed method to improve potency.

2. This extends to the comment from Reviewer #3 "…and the lack of a convincing demonstration of this discovery in the generation of a novel antibiotic". Currently we do not have a detailed understanding of how generalizable this indole-dependent phenotype is across chemical space. I think to address these concerns, some of the language should be toned down in a manner that highlights the limitations of this set of observations.

3. If I am interpreting Figure 5e and Figure 5l correctly, their model predicts that OctTriA lives in a chemical space that is inherently less permeable than JSF-2985. Given that OctTriA permeability is correlated with antibacterial activity, this may indicate that the indole substitution approach is better suited for molecules that are inherently less permeable, as opposed to increasing the permeability of molecules that are already reasonably able to permeate the mycomembrane.

4. In Figure 3f, the authors use SHAP values as a pseudo-interpretability method. However, these insights are not really leveraged in the rest of the paper. What is the overall purpose of this analysis if it's not being used to guide molecular optimization in downstream experiments? Additional explanation around this point would help readers understand how SHAP relates to their other modelling efforts.

(Remarks on code availability)

Decision Letter:

Our ref: NMICROBIOL-25041182B

11th May 2026

Dear Dr. Siegrist,

Thank you for submitting your revised manuscript "Chemical features that influence mycomembrane permeation and antitubercular activity" (NMICROBIOL-25041182B). It has now been seen by the original referees and their comments are below. The reviewers find that the paper has improved in revision, and therefore we'll be happy in principle to publish it in Nature Microbiology, pending minor revisions to satisfy the referees' final requests and to comply with our editorial and formatting guidelines.

If the current version of your manuscript is in a PDF format, please email us a copy of the file in an editable format (Microsoft Word or LaTex)-- we can not proceed with PDFs at this stage.

We are now performing detailed checks on your paper and will send you a checklist detailing our editorial and formatting requirements in about a week. Please do not upload the final materials and make any revisions until you receive this additional information from us.

Thank you again for your interest in Nature Microbiology Please do not hesitate to contact me if you have any questions.

Sincerely,

Reviewer #3 (Remarks to the Author):

This resubmission provides some clarity and is suitable for publication. There is lots of work here and it is a very important problem. That said, most of the methods are non-standard (for accumulation, inhibition of growth, etc) so readers will largely be unable to meaningfully scrutinize and understand these new methods, all the more reason that it will be important for the authors to demonstrate -- in future papers -- the actionability of their discoveries to the creation of novel antibiotics against TB.

Reviewer #4 (Remarks to the Author):

I appreciate the additional experiments and text revisions made during this round of revisions. I have a few comments that should be addressed. Notably, I do not want to impede the publication of this study because it's a useful contribution to the field,

but there are some claims that are beyond what I think the data directly supports.

1. In their responses, the authors state "The lack of correlation between mycomembrane permeation and growth inhibition for JSF-2985 suggested that the mycomembrane might not be an important barrier to its whole cell activity." However, in the manuscript text, they also state "Here, we found that JSF-2985 derivatives with imidazole, pyrazole, and pyrrolidine scaffolds permeate the mycomembrane better than the JSF-2985 derivative bearing cyclopentane, consistent with both cheminformatics analyses and MycoPermeNet predictions." My interpretation to these statements is that it is possible to improve JSF-2985 permeability, but doing so also can decrease its inherent antibacterial activity. I think this needs to be stated far more clearly. Simply increasing permeability is not a guaranteed method to improve potency.

2. This extends to the comment from Reviewer #3 "…and the lack of a convincing demonstration of this discovery in the generation of a novel antibiotic". Currently we do not have a detailed understanding of how generalizable this indole-dependent phenotype is across chemical space. I think to address these concerns, some of the language should be toned down in a manner that highlights the limitations of this set of observations.

3. If I am interpreting Figure 5e and Figure 5l correctly, their model predicts that OctTriA lives in a chemical space that is inherently less permeable than JSF-2985. Given that OctTriA permeability is correlated with antibacterial activity, this may indicate that the indole substitution approach is better suited for molecules that are inherently less permeable, as opposed to increasing the permeability of molecules that are already reasonably able to permeate the mycomembrane.

4. In Figure 3f, the authors use SHAP values as a pseudo-interpretability method. However, these insights are not really leveraged in the rest of the paper. What is the overall purpose of this analysis if it's not being used to guide molecular optimization in downstream experiments? Additional explanation around this point would help readers understand how SHAP relates to their other modelling efforts.

Version 3:

Decision Letter:

5th June 2026

Dear Professor Siegrist,

I am pleased to accept your Resource "Identification of chemical features for improved outer membrane permeation in mycobacteria using machine learning" for publication in Nature Microbiology. Thank you for having chosen to submit your work to us and many congratulations.

Over the next few weeks, your paper will be copyedited to ensure that it conforms to Nature Microbiology style. We look particularly carefully at the titles of all papers to ensure that they are relatively brief and understandable.

Once your paper is typeset, you will receive an email with a link to choose the appropriate publishing options for your paper and our Author Services team will be in touch regarding any additional information that may be required. Once your paper has been scheduled for online publication, the Nature press office will be in touch to confirm the details.

You may wish to make your media relations office aware of your accepted publication, in case they consider it appropriate to organize some internal or external publicity. Once your paper has been scheduled you will receive an email confirming the publication details. This is normally 3-4 working days in advance of publication. If you need additional notice of the date and time of publication, please let the production team know when you receive the proof of your article to ensure there is sufficient time to coordinate. Further information on our embargo policies can be found here:
https://www.nature.com/authors/policies/embargo.html

After the grant of rights is completed, you will receive a link to your electronic proof via email with a request to make any corrections within 48 hours. If, when you receive your proof, you cannot meet this deadline, please inform us at rjsproduction@springernature.com immediately. You will not receive your proofs until the publishing agreement has been received through our system

Due to the importance of these deadlines, we ask you please us know now whether you will be difficult to contact over the next month. If this is the case, we ask you provide us with the contact information (email, phone and fax) of someone who will be able to check the proofs on your behalf, and who will be available to address any last-minute problems.

Acceptance of your manuscript is conditional on all authors' agreement with our publication policies (see https://www.nature.com/nmicrobiol/editorial-policies). In particular your manuscript must not be published elsewhere.

Authors may need to take specific actions to achieve compliance with funder and institutional open access mandates. If your research is supported by a funder that requires immediate open access (e.g. according to Plan S principles or the NIH public access policy) then you

should select the gold OA route, and we will direct you to the compliant route where possible. Because authors warrant under our subscription licensing terms that they haven't committed to licensing any version of their article under a licence inconsistent with the terms of our agreement – including the applicable embargo period – publication under the subscription model isn't suitable for authors whose funders require no embargo.

If you have any questions about our publishing options, costs, Open Access requirements, or our legal forms, please contact ASJournals@springernature.com

An online order form for reprints of your paper is available at https://www.nature.com/reprints/author-reprints.html. All co-authors, authors' institutions and authors' funding agencies can order reprints using the form appropriate to their geographical region.

You can now use a single sign-on for all your accounts, view the status of all your manuscript submissions and reviews, access usage statistics for your published articles and download a record of your refereeing activity for the Nature journals.

To assist our authors in disseminating their research to the broader community, our SharedIt initiative provides you with a unique shareable link that will allow anyone (with or without a subscription) to read the published article. Recipients of the link with a subscription will also be able to download and print the PDF.

As soon as your article is published, you will receive an automated email with your shareable link.

With kind regards,

P.S. Click on the following link if you would like to recommend Nature Microbiology to your librarian http://www.nature.com/subscriptions/recommend.html#forms

** Visit the Springer Nature Editorial and Publishing website at www.springernature.com/editorial-and-publishing-jobs for more information about our career opportunities. If you have any questions please click here.**

Open Access This Peer Review File is licensed under a Creative Commons Attribution 4.0 International License, which permits use, sharing, adaptation, distribution and reproduction in any medium or format, as long as you give appropriate credit to the original author(s) and the source, provide a link to the Creative Commons license, and indicate if changes were made.
In cases where reviewers are anonymous, credit should be given to 'Anonymous Referee' and the source.
The images or other third party material in this Peer Review File are included in the article's Creative Commons license, unless indicated otherwise in a credit line to the material. If material is not included in the article's Creative Commons license and your intended use is not permitted by statutory regulation or exceeds the permitted use, you will need to obtain permission directly from the copyright holder.

To view a copy of this license, visit https://creativecommons.org/licenses/by/4.0/

16th May 2025

Dear Sloan,

Thank you very much for your patience while your manuscript "Identification of chemical features that influence mycomembrane permeation and antitubercular activity" was under peer-review at Nature Microbiology. It has now been seen by 3 referees, whose expertise and comments you will find at the end of this email. Although they find your work of some potential interest, they have raised a number of concerns that will need to be addressed before we can consider publication of the work in Nature Microbiology.

In particular, several of the referees raised a few concerns about aspects of the machine learning model applied. Referee #3 noted that some features were hand selected and had queries about this, while Referee #1 importantly noted that some additional analyses are needed to more fully demonstrate that the model has predictive function and has learned structure-function relationships. There were also requests for further validation, including dose-response curves for parent versus modified compounds, to more clearly demonstrate improved penetration with given scaffolds, as well as LC-MS/MS to validate some findings. Referee #1 also asked for more explanation as to why certain moieties were followed up and others not, and had doubts over whether the azide modification might influence molecule characteristics. The referees generally also noted that some reworking of the manuscript text and figures would be needed to make this more accessible for a more microbiology-focussed audience who may not be expert in some of the computational approaches used. In light of Referee #3's comments, we also felt it would be important for the manscript to better communicate what the key take home messages are for molecular scaffolds that confer better penetration capabilities, and if possible, to further explore or demonstrate how application of these principles can lead to better penetration/antimicrobial activity. We realise that this may be complicated, but think it would really strengthen the manuscript and emphasise the significant advance this approach and findings potentially provide.

Should further experimental data allow you to address these criticisms, we would be happy to look at a revised manuscript.

SUMMARY

Thank you to the reviewers and editor for taking the time to read our manuscript and provide thoughtful comments and suggestions. To address, we have:

1) Explained our feature selection, **lines 234-237**

"… we used a surrogate XGBoost to predict the outputs of MycoPermeNet from 25 interpretable, hand-selected input features (23 calculated with RDKit and 2 calculated with Collaborative Drug Discovery [CDD] Vault) previously shown to be important for permeability across Gram-negative membranes."

2) Performed additional analyses for the machine learning models to demonstrate their predictive capabilities, **Fig. 2b, 2c, 2e, 2f**; **Fig. S4a-e**; **lines 194-198**

"To minimize overlap between the train and test sets, we used a Bemis-Murcko scaffold split to hold out 10% of the data for testing. We then performed model selection and hyperparameter tuning on the training and validation sets, together constituting 90% of the data. The two stages are trained independently but neither observe the test data."

3) Performed dose-response curves for permeation and activity (**Fig. 4b, 4f, S6a** and new experiments in **Fig. 5c, 5e, 5k, 5l, S7b, S7d, S7e**; see the end of the **Summary** section for full figures).

Fig. 4b

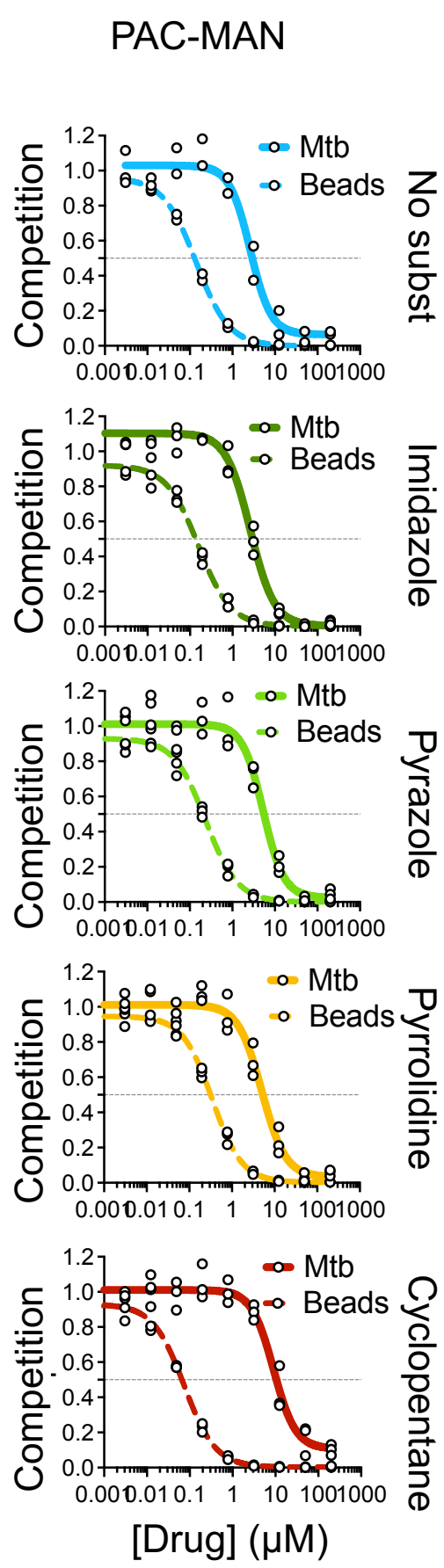

Fig. 4f (Mtb)

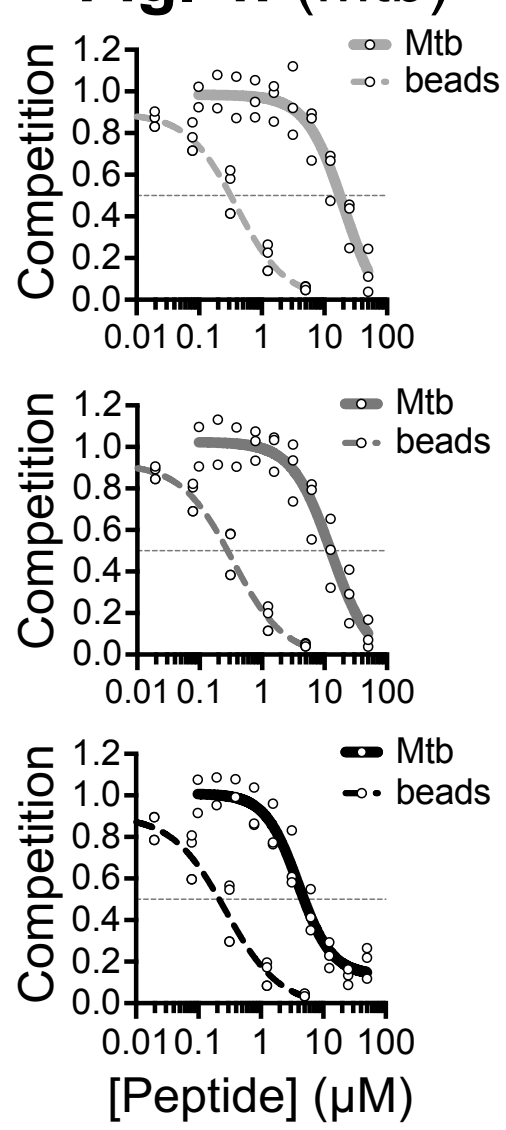

Fig. S6a (Msm)

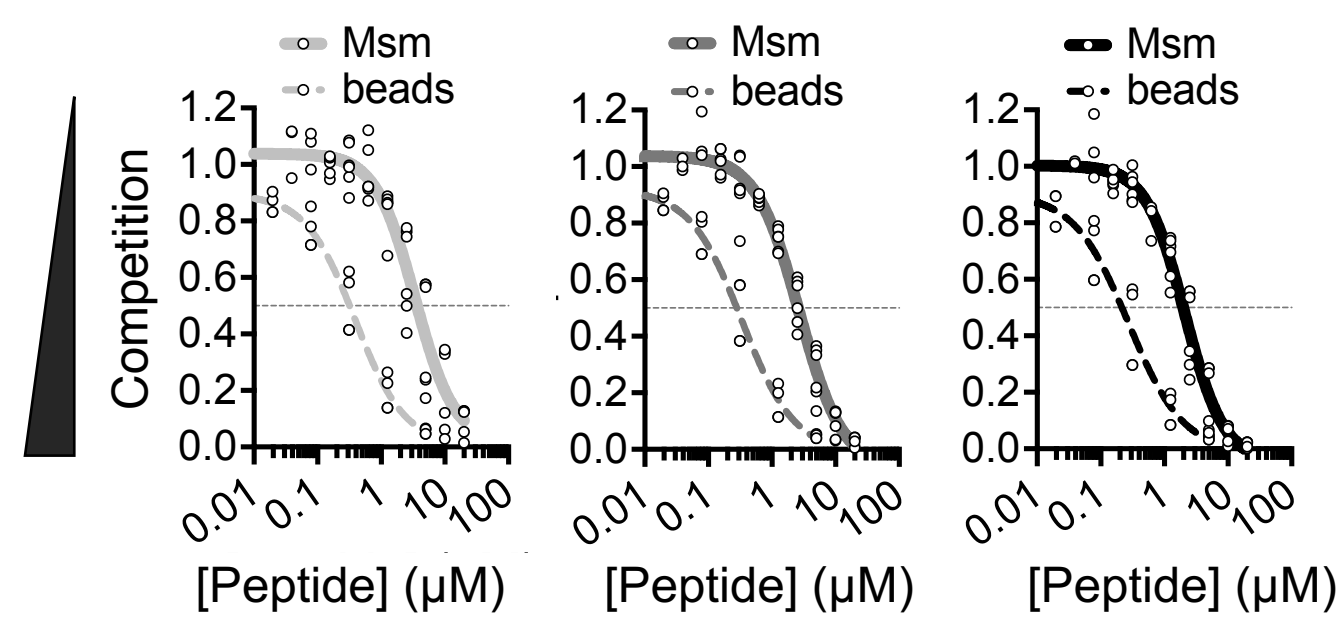

4) Tested effect of benzene to indole substitution on model peptide via LC-MS (new experiment in **Fig. S6d**). These results agree with both PAC-MAN data (**Fig. S6b**) and MycoPermeNet predictions (**Fig. S6c**).

Fig. S6d

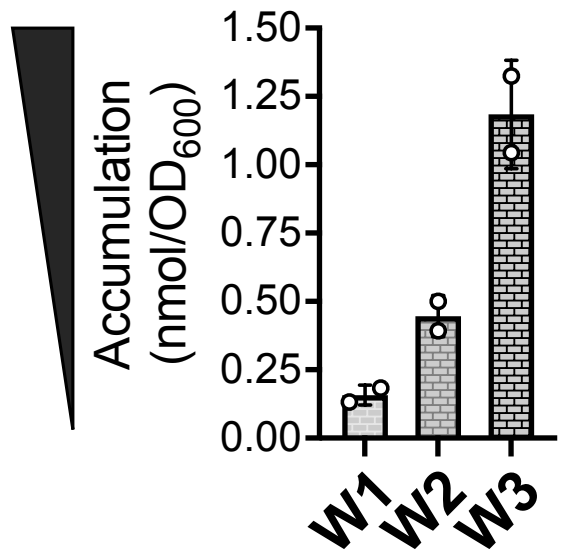

Fig. S6b

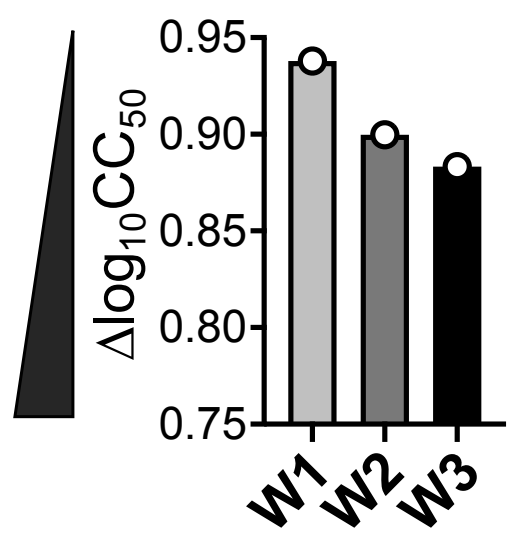

Fig. S6c

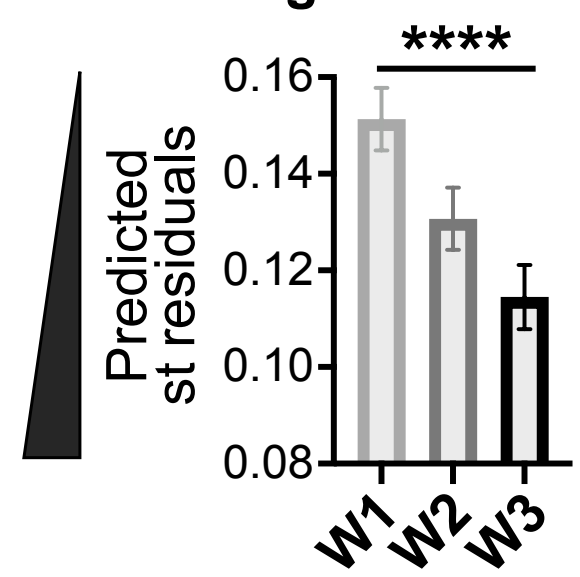

5) Tested effect of azide modification on accumulation via LC-MS (new experiment in **Fig. S8a**) and computational predictions (new experiment in **Fig. S8b**)

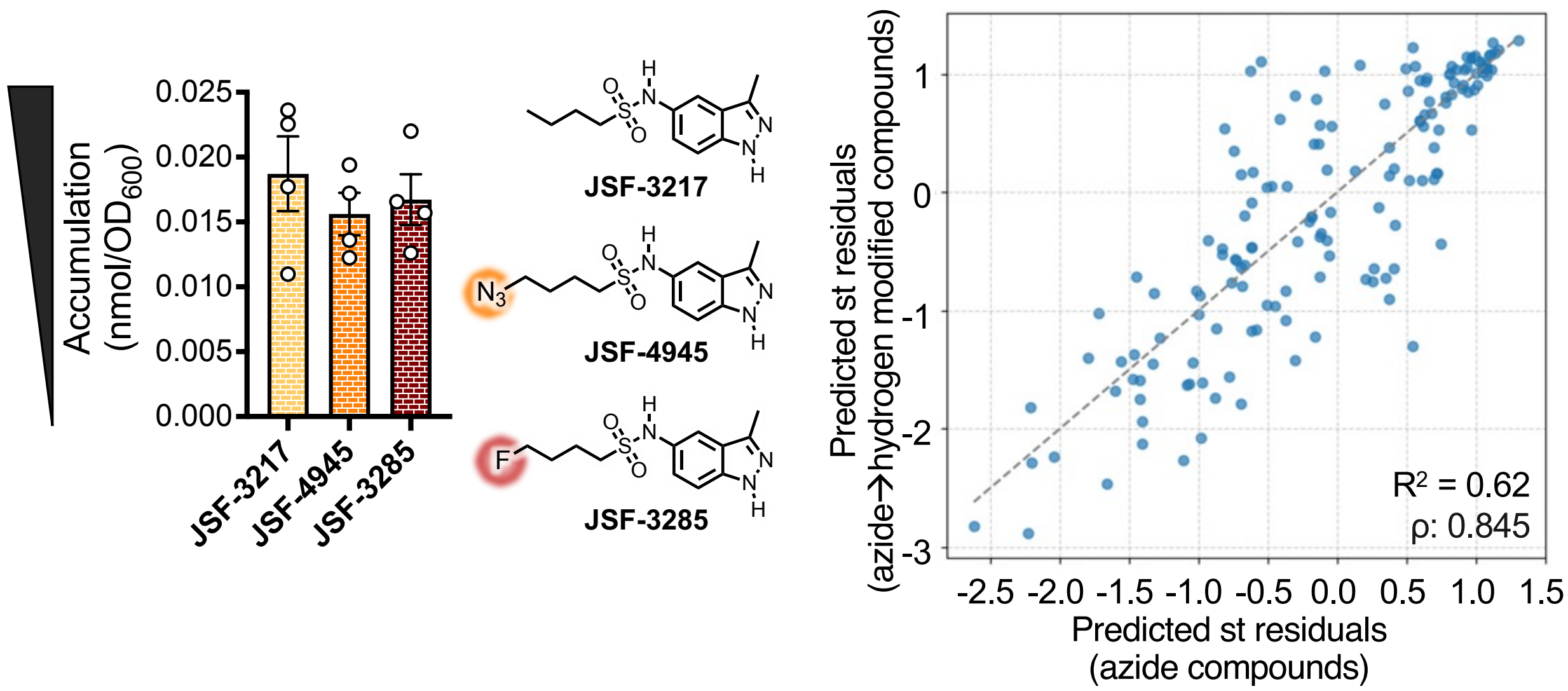

Figure S8. Effect of azide group on compound accumulation and mycomembrane permeation. (**a**) LC-MS whole cell accumulation for anti-Mtb drug candidate **JSF-3285** and analogs that have either an azide group (**JSF-4945**) or a hydrogen (**JSF-3217**) replacing the fluorine atom. Data represent mean of 4 technical replicates +/- SEM from one representative (of two) biological replicates. (**b**) Correlation between predicted standard residuals for azide-modified compounds (X-axis) and computationally-derived derivatives in which the azide is replaced with a hydrogen atom (Y-axis). Datapoints represent the compounds in the test set.

6) More comprehensively addressed limitations of our work, including potential effect(s) of azide tag (**lines 386-394** and **476-497**):

*“Because these molecules lack azide tags, we first tested by LC-MS how an azide might impact accumulation of a compound. We found that for **JSF-3285**, an antitubercular molecule related to **JSF-2985**, neither removal of fluorine nor substitution with an azide in the same location impacted whole cell accumulation (**Fig. S8a**). Since the effects of an azide tag may be molecule-specific, we next tested the performance of MycoPermeNet on matched compounds with and without the modification (-N_3 vs. -H). The concordance between predictions (**Fig. S8b**)*

suggested the utility of using the model, which was trained on azide-modified compounds, to predict the behavior of unmodified compounds."

and

*"By chemically trapping molecules in the periplasm, PAC-MAN enables subcellular localization while alleviating technical problems associated with non-specific association, e.g., with the mycomembrane, and loss of the molecule during sample processing. However, a caveat to the method is the requirement for an azide tag. Azides are widely recognized as ideal bioorthogonal tags because they are small and have minimal known impacts on the physicochemical properties of the parent compound. While the accumulation of an azide-tagged molecule was like that of unmodified compounds by LC-MS (**Fig. S8a**), we do not rule out potential effects of the azide on mycomembrane permeation or activity for other molecules. Indeed, azide functionalization of **JSF-2985** and octyl-tridecaptin derivatives modestly decreased activity (**Fig. S5b-c**, **Fig. 5j**), as we have observed for other antibiotics. However, we note that the chemical features that we identified as predictors of this phenotype from azide-tagged libraries are also correlates of whole cell activity for untagged compounds (**Fig. 6**, **Fig. S9**, **Fig. S10**). These data are consistent with the concordance of MycoPermeNet predictions for matched compounds modified or not with an azide (**Fig. S8b**) and with our previous finding that sensitivity of azide-modified drug permeation to envelope perturbation tracks closely with that of unmodified parent drug activity. The constancy of the azide across all the test molecules, the large number of test molecules, and the redundancy of some test molecules (i.e., compounds that differ only in the position of the azide) may contribute to our ability to predict mycomembrane permeation and functional associations with whole cell activity."*

7) Reworked the manuscript to enhance clarity and utility, including more focus on scaffolds in general and indole specifically as permeability- and activity-promoting moieties (e.g., **lines 50-53; 107-110; 157-163; 429-438** and new experiments in **Fig. 5** and **S7** [see the end of the **Summary** section for full figures] and related headings/text).

"Chemical predictors of mycomembrane permeation include scaffolds like indole and can promote anti-Mtb activity. Our data suggest a rational framework for improving mycomembrane permeation and whole cell activity of antituberculars."

and

"Finally, we showed that scaffolds like aromatic nitrogen heterocycles, especially indole, and other chemical predictors of mycomembrane permeation can promote anti-Mtb activity in different molecular contexts."

and

"For both Msm and Mtb, we found that aromatic nitrogen-containing scaffolds like indole, imidazole, or pyrazole correlate positively with permeation (negative medians for standardized

*residuals), while scaffolds like cyclopentane or cyclohexane correlate negatively (positive medians). Across our analyses, aromatic scaffolds, especially nitrogen heterocycles, positively associate with permeation while non-aromatic ring systems, including some heterocycles, negatively associate with permeation (**Fig. 2a**, **Fig. 2b, Fig. S3a**).”*

and

“Here we find that the effects of physicochemical properties on mycomembrane permeation are scaffold-dependent. Furthermore, scaffolds alone, particularly aromatic nitrogen heterocycles like indole, associate with and/or promote mycomembrane permeation in different molecular contexts. In pharmacology, indole is considered a privileged scaffold because it is highly represented across drug classes, can effectively engage with a variety of targets, and has useful chemical reactivity that allows versatile substitutions for derivatization. At present, several compounds under exploration as anti-Mtb drugs are indole-based. Our data suggest that, for mycobacterial species, indole may also be a privileged scaffold in terms of cell permeation.”

8) Reworked the manuscript with more attention to microbiology-focused audience (e.g., **227-231**).

“*Because neural networks have large numbers of parameters, additional techniques are needed to determine what changes in the input data will lead to changes in the output predictions. In our case, the input data are chemical structures, which are meaningful individually but difficult to summarize across our large and diverse dataset*.”

9) Demonstrated application of our learning with new data that show that benzene to indole substitution on an antimicrobial peptide promotes both mycomembrane permeability and whole cell activity (new experiments in **Fig. 5** and **S7** below).

We are excited to share our new data (**Fig. 5, S7, S8** and updates to **Fig. S3b** [below, in **Rev 2 Q2**], **Fig. S6d** [above]) and extensive text changes (marked in blue in main text), which we think have substantially strengthened the manuscript and made it more accessible to a microbiology-focused audience.

Finally, for clarity we point out that **Fig. 5** from the previous submission is now **Fig. 6** and some of the supplemental figures have been merged per editor request. We also number the references separately in this document and in the main text.

FULL FIGURE 5 and FIGURE S7

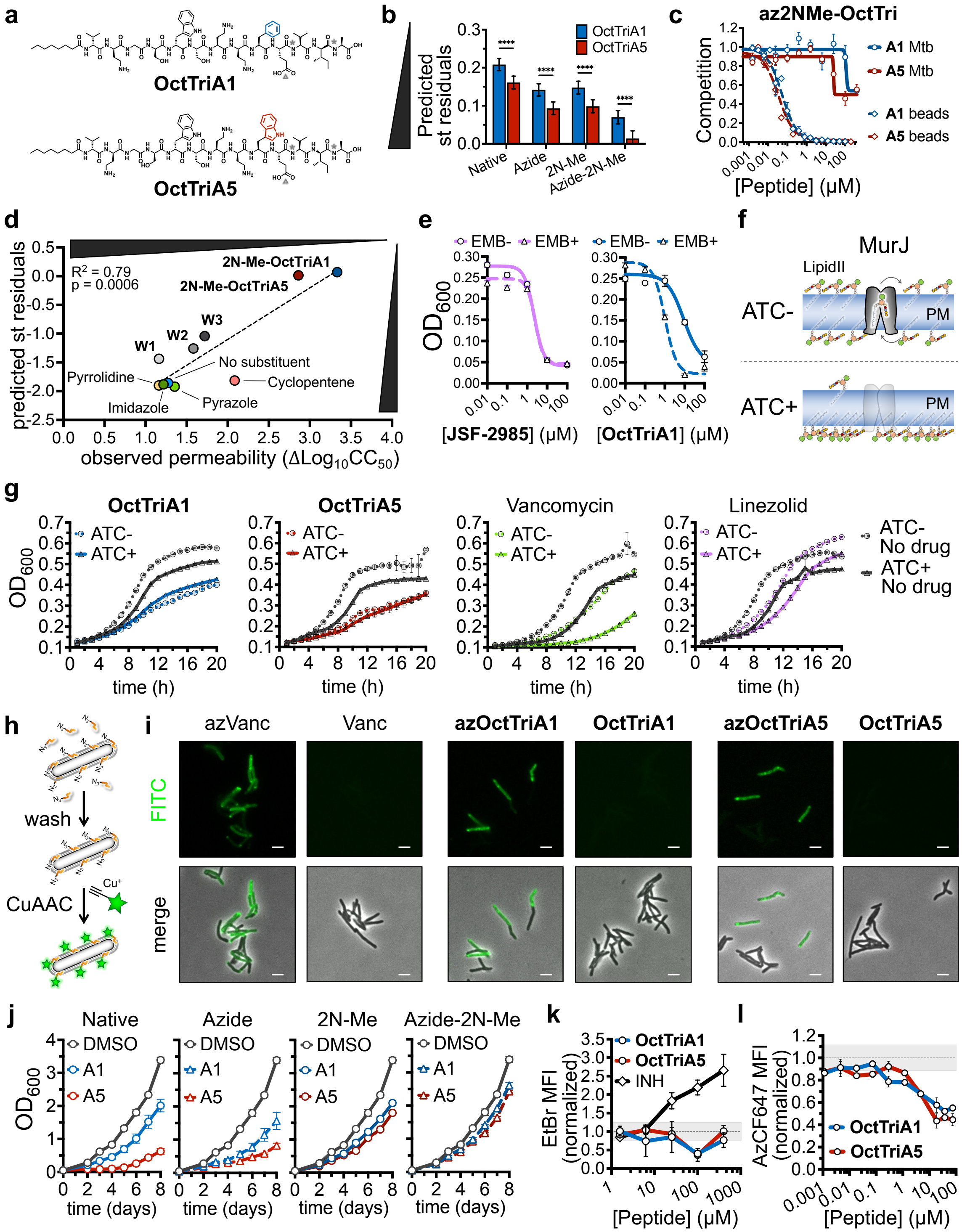

Figure 5. Benzene to indole substitution can promote mycomembrane permeability and anti-Mtb activity of octyl-tridecaptins. (**a**) Chemical structures of **OctTriA1** and **OctTriA5**. Grey asterisks, *N*-methylation sites; grey triangles, azide derivatization sites. See **Fig. S7a** for corresponding azide and methylated derivatives. (**b**) MycoPermeNet predictions for the **OctTriA1** and **OctTriA5** derivative pairs. Data are mean and SEM generated by 50 random states of the model. Wilcoxon rank-sum test: (****) p<0.0001. (**c**) PAC-MAN results for methylated, azide-modified octyl-tridecaptin derivatives for Mtb and beads (unmethylated derivatives in **Fig. S7b**). (**d**) Correlation between observed and ML-predicted mycomembrane permeability across three compound series. Observed permeability values for **OctTriA1** and **OctTriA5** are estimated, see **Methods** for details. (**e**) Mtb growth inhibition curves for **JSF-2985** and **OctTriA1** +/- sub-inhibitory ethambutol (EMB) co-treatment. (**f**) Schematic representation of MurJ depletion (in the presence of anhydrotetracycline [ATC]) and its effect on lipid II availability in the outer leaflet of the plasma membrane (PM). (**g**) MurJ was depleted (+ATC) or not (-ATC) in the presence of absence of **OctTriA1** (37.5 μM), **OctTriA5** (12.5 μM), vancomycin (3.1 μM), and linezolid (0.125 μM). Drug concentrations were titrated to achieve similar levels of Msm growth inhibition. For comparison of **OctTriA1** and **OctTriA5** at same concentration see **Fig. S7c**. (**h**) Schematic representation of the copper(I)-catalyzed azide-alkyne cycloaddition (CuAAC)-based assay to visualize azide-vancomycin, -**OctTriA5**, and -**OctTriA5** and (**i**) related fluorescent micrographs from Msm. Scale bars, 3 μm. (**j**) Mtb growth curves upon treatment with the four pairs of octyl-tridecaptin derivatives. (**k-l**) Mtb cell envelope permeabilization by octyl-tridecaptins: ethidium bromide (EtBr) uptake (**k**) and accessibility of DBCO-labeled peptidoglycan to an azide-fluorophore (**l**) performed as in[7] [4 in this file]. Data are technical replicates +/- SEM from one of three biological replicates.

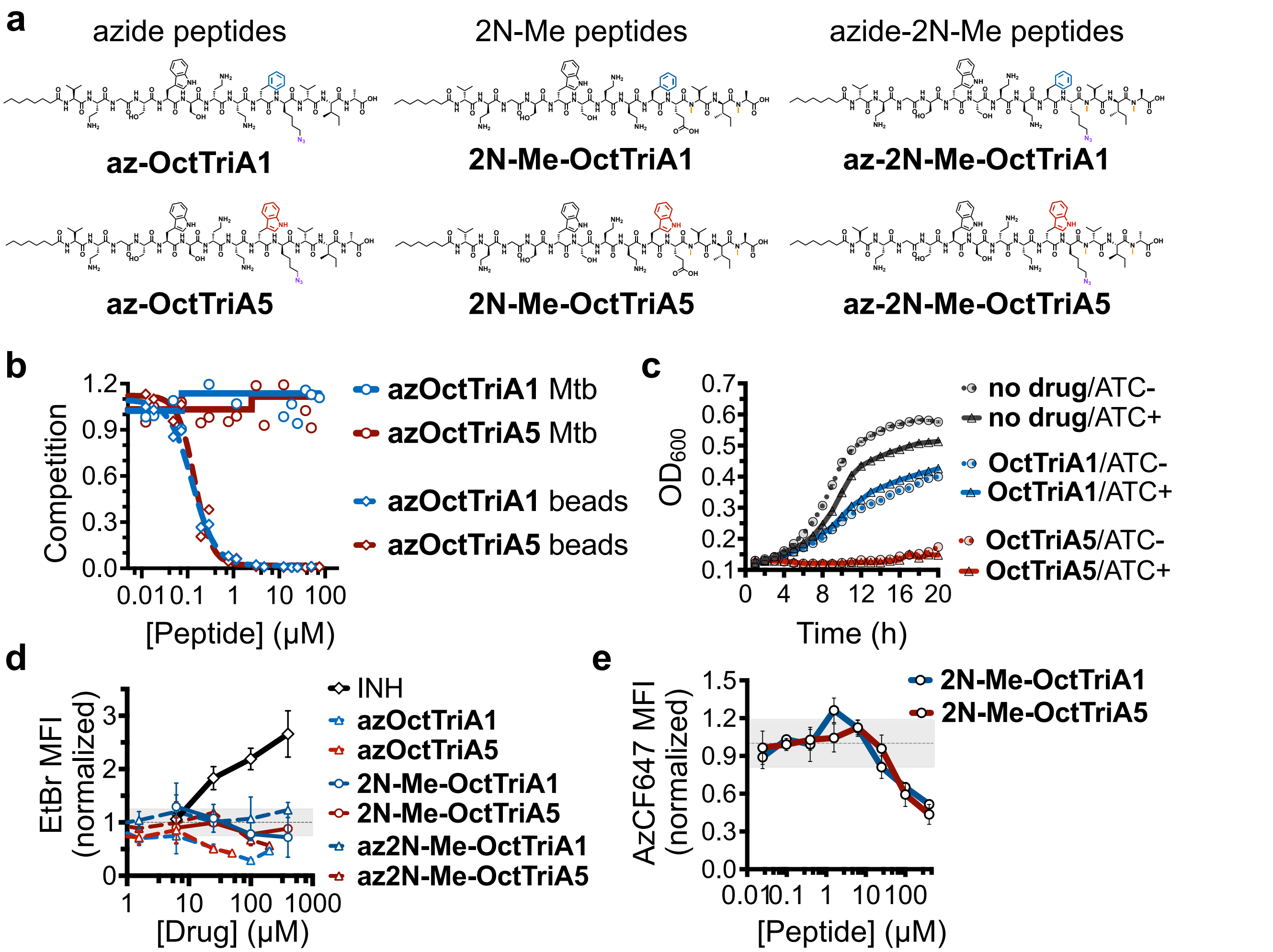

Figure S7. Extended data for Figure 5. (**a**) Chemical structure of the **azide-**, **2N-methyl-** and **azide-2N-methyl-** versions of **OctTriA1**and **OctTriA5**, antibacterial peptides. (**b**) PAC-MAN results for **azide-OctTriA1** and **azide-OctTriA5** peptides for both Mtb and DBCO-beads. (**b**) Msm MurJ^{-} mutant growth curves +/- ATC upon treatment with, **OcrTriA1** and **OcrTriA5** at 37.5 μM. (**c**) Mtb envelope permeabilization of **azOctTriA1**, **azOctTriA5**, and their 2N-methylated version measured by ethidium bromide (EtBr) assay. (**d**) Mycomembrane permeabilization of **2N-Me-OctTriA1** and **2N-Me-OctTriA5** assessed by accessibility of DBCO-labeled peptidoglycan to an azide-fluorophore (**e**). Data are 3 technical replicates +/- SEM from one of three biological replicates; panel **b**, individual datapoints for 2 biological replicates. MFI: mean fluorescence intensity.

Reviewer Expertise:

Referee #1: AI/ML, drug discovery
Referee #2: mycobacteria, membrane biology
Referee #3: chemistry, antibiotic development, antibiotic accumulation

Reviewer Comments:

Referee #1: AI/ML, drug discovery

Reviewer #1 (Remarks to the Author):

The manuscript is well written and focused on an important problem. I enjoyed reading it. The authors leverage a previously developed assay to assess the permeability of ~1500 small molecules through the cell wall of Gram-positive mycobacteria. Their analysis identifies chemical moieties that correlate with permeability, which are then used to optimize a previously reported compound.

Acceptance of the manuscript should be contingent upon addressing the following points.

MAJOR COMMENTS

Rev 1 Q1. I would like to better understand the rationale behind selecting imidazole, pyrazole, pyrrolidine, and cyclopentane for the optimization of JSF-2985. While Figure 2A highlights these groups, other moieties (such as indole) appear to show even stronger positive correlation with permeability but were not chosen. Similarly, many groups exhibit strong negative correlation. Please clarify how the final selection was made, and why certain promising scaffolds were excluded.

We aimed for the derivatives to be structurally similar to each other, reasoning that substituents of the same size would enable the most rigorous, apple-to-apple comparisons. Following this rationale, for **JSF-2985** we investigated a subset of the 5-membered rings shown in **Fig. 2a**. We opted for 5-membered rings for two reasons. First, we wanted substituents that were significantly smaller than the parent molecule to minimize potential effects on target engagement. In the case of **JSF-2985**, an indole substituent would have been almost the same size as the parent molecule, increasing the probability that we would change target engagement. Second, 5-membered rings are highly represented among our scaffolds, which enabled us to choose substituents with different observed permeability associations.

As the reviewer points out, indole had a strong positive association with mycomembrane permeability in our screen, so we opted to test for causation using larger parent molecules: a) the **W** peptide series, which do not have intrinsic antibacterial activity (synthesized by us); b) octyl-tridecaptin peptides, which in other species were known to use the peptidoglycan precursor lipid II as a receptor from which to inhibit growth (synthesized by us); c) two molecule

sets from MLSMR collection which inhibit Mtb growth via unknown mechanisms (retrospective analysis). The **W** peptide series was an important proof-of-concept to establish that indole not only correlates with permeability (per cheminformatics and ML) but can also promote this property (**Fig. 4e-h**). New to the revision, we have added LC-MS data that supports this claim (**Fig. S6d**, see **Summary** section above). The octyl-tridecaptin peptides (also new to this revision) and MLSMR molecule sets enabled us to test the hypothesis that indole promotes both permeability (observed and/or predicted; **Fig. 5b-c**, see **Summary** section above) and whole cell activity (**Fig. 5j** for Mtb and **Fig. S7c** for Msm, see **Summary** section above).

Rev 1 Q2. On line 246, the manuscript states that a molecule with a positively correlated group permeates better than the cyclopentane derivative. However, for a meaningful comparison, one would expect the derivative to be compared to the parent (unmodified) compound. If the cyclopentane group correlates negatively with permeability, it should perform worse. Am I misinterpreting the statement? I recommend showing dose–response curves for each derivative directly compared to the original molecule in Figure 4F. The current comparison against beads is informative, but this data could be moved to the Supplementary Information.

We performed dose-response curves for each derivative on Mtb. Because we have the additional variable of azide reactivity, for which we use dose-response curves on beads to control, we cannot directly compare dose-response Mtb curves to each other. Instead, we first must calculate $\log_{10}CC_{50}$ by comparing Mtb and bead curves (**Fig. 4b, 4f, Fig. 5c**, **and Fig. S6a**, see **Summary** section above), then compare via $\Delta\log_{10}CC_{50}$ (for further details see **Rev 1 Q5**).

To the reviewer's second point, we think that comparisons between structurally similar **JSF-2985** derivatives are more informative than comparisons between the derivatives and parent. Where the parent molecule has a 2-position primary amide, the derivatives have the amide functionalized with various 5-membered rings.

Rev 1 Q3. Figure 3B suggests the ML model predicts residuals well, but I am concerned it may simply be memorizing the data (i.e., overfitting) rather than learning meaningful structure–activity relationships. To better evaluate generalization, I recommend the authors repeat the ML analysis using their 80-10-10 train-validation-test splits, with the test set consisting of compounds that are significantly dissimilar (e.g., by Tanimoto distance) from those in the training set. One way to achieve this is by scaffold-based splitting. The performance (e.g., ρ, R^2) should be reported as mean ± SEM across multiple splits. If predictive performance under these conditions is poor, Figures 3C, 3E, and 3F should be toned down (or removed). That said, even in the absence of predictive value, the ML model may still serve as a useful tool to summarize structure–permeability relationships within the dataset. However, in this case, its role would overlap substantially with the chemoinformatics analysis, and this redundancy should be acknowledged.

We have made the following changes to better evaluate generalization of our ML model. First, we use a Bemis-Murcko scaffold split to divide the data into a 90-10 train/val-test split, with 90% for training and validation and 10% for testing. As before, model selection and hyperparameter

tuning was performed only on the training and validation sets (together constituting 90% of the data). Second, we evaluate the performance across both random model initialization seeds (7x) and across different splits of the train/val set (7x), for a total of 49 different models. Third, we calculate the R^2, RMSE, and Spearman across these 49 splits for both train and test. We now report these results (**Fig. 3b**) and summarize them (mean ± SEM) below:

Train R^2: 0.6579 ± 0.0072
Train RMSE: 0.5702 ± 0.0056
Train Spearman: 0.8138 ± 0.0028

Validation R^2: 0.6257 ± 0.0075
Validation RMSE: 0.5948 ± 0.0062
Validation Spearman: 0.7967 ± 0.0029

Test R^2: 0.5127 ± 0.0078
Test RMSE: 0.7493 ± 0.0058
Test Spearman: 0.7415 ± 0.0029

Then, to maximize the model's predictive capability on unseen data, we retrain it using the entire dataset, as is routinely done in cheminformatics studies[1-3]. This "full" version of the model is used in **Fig. 3c** and onwards. We now more clearly articulate these steps in both the Methods and **lines 194-198**:

"To minimize overlap between the train and test sets, we used a Bemis-Murcko scaffold split to hold out 10% of the data for testing. We then performed model selection and hyperparameter tuning on the training and validation sets, together constituting 90% of the data. The two stages are trained independently but neither observe the test data."

Finally, to more accurately reflect variation in the performance of the final model on new data, we repeat the model training on the entire dataset with 50 different random seeds and report the mean and confidence interval of model performance on the subsequent molecule series.

Reference 1: Seal, S. et al., Integrating cell morphology with gene expression and chemical structure to aid mitochondrial toxicity detection. *Commun Biol* 2022, *5* (1), 858.

Reference 2: Moradi, et al., Assessing the effects of therapeutic combinations on SARS-CoV-2 infected patient outcomes: A big data approach. *PLoS One* 2023, *18* (3), e0282587.

Reference 3: Puszkarska, et al., Machine learning designs new GCGR/GLP-1R dual agonists with enhanced biological potency. *Nat Chem* 2024, *16* (9), 1436-1444.

MINOR COMMENTS

Rev 1 Q4. In Figure 1A, the test molecule is represented by a large “R” (yes?) labeled “test azide,” presumably indicating it is bound to an N_3 group for TetD labeling. I assume “R” stands for “residue,” but this is not clearly stated in the figure or legend, and took some effort to deduce. Please clarify this notation explicitly.

We have now explained the meaning of “R” in **Fig. 1** legend:

“Mycobacteria are then exposed to an azide-modified test molecule (R = residue)…”

Rev 1 Q5. The assay readout is “competition,” which reflects both permeability and reactivity. The manuscript would benefit from a more explicit discussion of whether these two components can be disentangled. Are the authors primarily interested in permeability? If so, how do they control for reactivity effects?

The reviewer is correct that we are interested in permeability/accumulation, not in reactivity. We control for intrinsic differences in azide reactivity in two different ways. Both require DBCO-functionalized polystyrene beads that have bacteria-like dimensions but are devoid of a permeability barrier.

In the first method, we screen many azide-tagged molecules on DBCO-beads and on DBCO-labeled bacteria at a fixed concentration. Standardized residuals are then extracted from log (bead) **-** linear (bacterium) regression analyses (**Fig. 1** and **Fig. S2**). We think this is well-suited for high-throughput experiments, albeit less precise for compounds that have very low or very high reactivity. To this end, in the second method we perform dose-response curves for select azide-tagged molecules on DBCO-beads and on DBCO-labeled bacteria, then calculate the difference in concentration of azide-tagged molecule that competes fluorescence by 50% in the two systems ($\Delta log_{10}CC_{50}$; **Fig. 4, S6a, 5c,** and developed in [4]).

Please see **lines 137-150** for more discussion on this critical point:

*“To control for the intrinsic reactivities of azide test molecules toward DBCO, we also screened DBCO-functionalized polystyrene beads that have bacteria-like dimensions but are devoid of a permeability barrier (**Fig. 1b**). Previously we controlled for reactivity and calculated mycomembrane permeation by exposing DBCO-labeled beads and mycobacteria to different concentrations of azide test molecules, then calculating the difference in concentration of test azide molecule that competes fluorescence by 50% in the two systems ($\Delta log_{10}CC_{50}$). For higher-throughput calculation of mycomembrane permeation, we instead exposed DBCO-labeled beads and mycobacteria to fixed concentrations of test azide molecules then performed log-linear regression analyses to calculate the differences between observed and expected fluorescence (standardized residuals; **Fig. 1b**; **Fig. S2a**, **Supplemental File 1**). The standardized residuals for compounds shared between the Sharpless/Dong and Enamine libraries were consistent (**Fig. S2b**).”*

Reference 4: Lepori, I.; Jackson, K.; Liu, Z.; Chordia, M. D.; Wong, M.; Rivera, S. L.; Roncetti, M.; Poliseno, L.; Freundlich, J. S.; Pires, M. M.; Siegrist, M. S., The Mycomembrane Differentially and Heterogeneously Restricts Antibiotic Permeation. *ACS Infect Dis* **2025,** *11* (7), 1893-1906.

Rev 1 Q6. I would appreciate efforts to improve the clarity of Figure 2. The caption states that the scaffolds shown in panels A and B correspond to those listed on the y-axis of panel C, but there is no clear mapping between scaffold names and their chemical structures—making it difficult to identify, for example, which structure represents indole. One might assume the scaffolds are presented in the same order across panels, but this is made more confusing by inconsistencies in the compound counts. For instance, indole is reported to have 28 compounds in panel A but only 26 in panel C. I understand that panel B may contain fewer compounds due to selection criteria, but shouldn't panels A and C include the same number of compounds per scaffold? Please clarify both the ordering and the source of these discrepancies.

We have added scaffold names to **Fig. 2a** and updated the analysis to make the panels consistent.

Rev 1 Q7. A natural question arises as to whether the azide modification affects antibacterial activity. Azides can be reactive and potentially alter compound potency. The discussion touches on this, but further clarification would be valuable. Ideally, the authors could provide dose–response curves for the azide-tagged versus parent compounds in at least one Mycobacterium strain. MIC or IC_{50} values would help clarify this point.

Azides are ideal bioorthogonal tags because they are compact and inert to other functional groups in the cell[5, 6]. However, azide modification can indeed impact molecule activity, depending on the antibacterial of interest and site of modification. For example, we (M.M.P.) previously showed that some azide-modified antibiotics have higher MICs than parent compounds against *S. aureus*[7]. In the current manuscript, azide modification decreases **JSF-2985** activity (growth inhibition after 6 days in **Fig. S5b** and MIC in **Fig. S5c**) and more modestly affects octyl-tridecaptin activity (below are **Fig. 5j** data regraphed to highlight +/- azide differences). We think that the effects of azide modification on antimicrobial activity are most likely due to alterations in physicochemical properties (polarity, steric hindrance, etc.) that perturb target engagement rather than non-specific reactivity of the azide moiety.

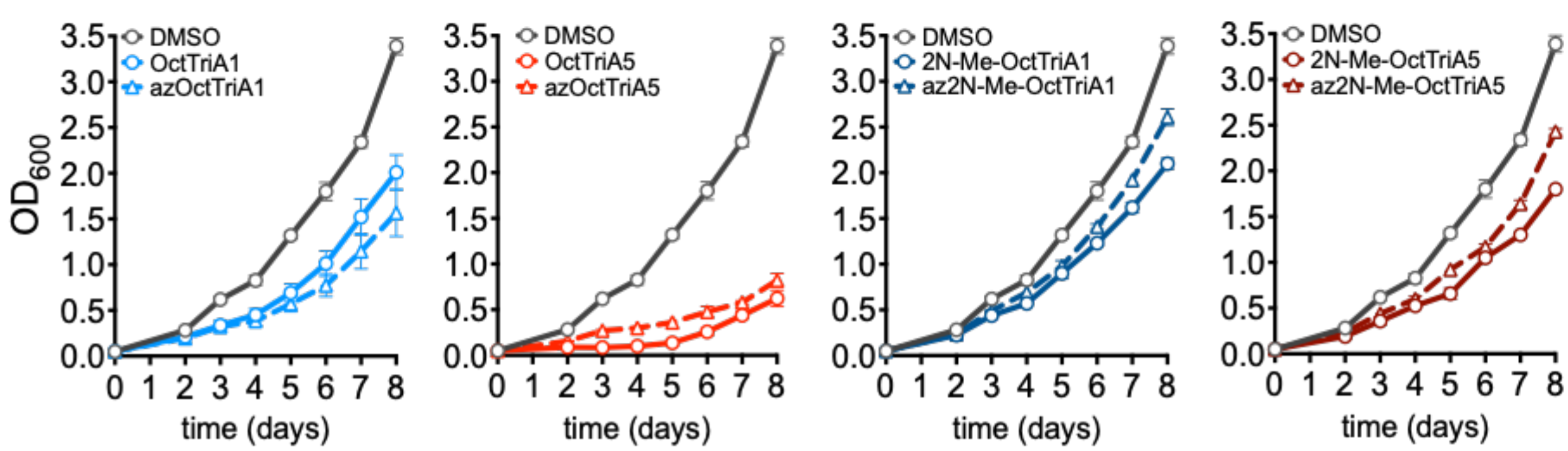

Reference 5: Agard, N. J.; Prescher, J. A.; Bertozzi, C. R., A strain-promoted [3 + 2] azide-alkyne cycloaddition for covalent modification of biomolecules in living systems. *J Am Chem Soc* **2004,** *126* (46), 15046-7.

Reference 6: Jewett, J. C.; Bertozzi, C. R., Cu-free click cycloaddition reactions in chemical biology. *Chem Soc Rev* **2010,** *39* (4), 1272-9.

Reference 7: Kelly, J. J.; Dalesandro, B. E.; Liu, Z.; Chordia, M. D.; Ongwae, G. M.; Pires, M. M., Measurement of Accumulation of Antibiotics to Staphylococcus aureus in Phagosomes of Live Macrophages. *Angew Chem Int Ed Engl* **2024,** *63* (3), e202313870.

Despite the drawbacks associated with tagging, we have shown that azide-modified drugs can yield useful information. For example, in [4] we show good concordance between the permeability of azide drugs in the presence or absence of different mycomembrane perturbations vs. whole cell activity of unmodified parent drugs. In that publication we did not query the activity of the azide drugs. Here, partially in recognition that the azide modification might have limited our ability to assess the effect(s) of permeability-impacting modifications on **JSF-2985**, for our new work on octyl-tridecaptins we synthesized derivatives both with and without azide modification (**Fig. 5** and **Fig. S7,** see **Summary** section above).

Reference 4: Lepori, I.; Jackson, K.; Liu, Z.; Chordia, M. D.; Wong, M.; Rivera, S. L.; Roncetti, M.; Poliseno, L.; Freundlich, J. S.; Pires, M. M.; Siegrist, M. S., The Mycomembrane Differentially and Heterogeneously Restricts Antibiotic Permeation. *ACS Infect Dis* **2025,** *11* (7), 1893-1906.

Azide modification may also impact permeability. In the one case where we have used LC-MS to directly compare the accumulation of a compound +/- azide, we found that whole cell accumulation of these compounds was similar (**Fig. S8a,** see **Summary** section above). However, this is an n=1 and we think that, as with activity, potential effect(s) of an azide on molecule permeability may depend on the molecule of interest and site of modification. We note that the chemical features that we identified as correlates of periplasm accumulation based on screening azide-tagged libraries are also correlates of whole Mtb cell activity for unmodified compounds (**Fig. 6, S9, S10**). As well, there is general concordance between MycoPermeNet predictions for the same set of compounds modified with either an azide or hydrogen (**Fig. S8b,** see **Summary** section above). We think these data, along with the similarity in behavior between azide-modified drugs and unmodified parent drugs mentioned above, suggest that tag-based methods can be useful with careful controls and interpretation.

In sum, we agree with the reviewer that this is a natural question. We now address potential effect(s) of the azide tag directly in **lines 386-394** and **476-497**:

"Because these molecules lack azide tags, we first tested by LC-MS how an azide might impact accumulation of a compound. We found that for ***JSF-3285****, an antitubercular molecule related to*

__JSF-2985__, neither removal of fluorine nor substitution with an azide in the same location impacted whole cell accumulation (__Fig. S8a__). Since the effects of an azide tag may be molecule-specific, we next tested the performance of MycoPermeNet on matched compounds with and without the modification (-N_3 vs. -H). The concordance between predictions (__Fig. S8b__) suggested the utility of using the model, which was trained on azide-modified compounds, to predict the behavior of unmodified compounds."

and

"By chemically trapping molecules in the periplasm, PAC-MAN enables subcellular localization while alleviating technical problems associated with non-specific association, e.g., with the mycomembrane, and loss of the molecule during sample processing. However, a caveat to the method is the requirement for an azide tag. Azides are widely recognized as ideal bioorthogonal tags because they are small and have minimal known impacts on the physicochemical properties of the parent compound. While the accumulation of an azide-tagged molecule was like that of unmodified compounds by LC-MS (__Fig. S8a__), we do not rule out potential effects of the azide on mycomembrane permeation or activity for other molecules. Indeed, azide functionalization of __JSF-2985__ and octyl-tridecaptin derivatives modestly decreased activity (__Fig. S5b-c__, __Fig. 5j__), as we have observed for other antibiotics. However, we note that the chemical features that we identified as predictors of this phenotype from azide-tagged libraries are also correlates of whole cell activity for untagged compounds (__Fig. 6__, __Fig. S9__, __Fig. S10__). These data are consistent with the concordance of MycoPermeNet predictions for matched compounds modified or not with an azide (__Fig. S8b__) and with our previous finding that sensitivity of azide-modified drug permeation to envelope perturbation tracks closely with that of unmodified parent drug activity. The constancy of the azide across all the test molecules, the large number of test molecules, and the redundancy of some test molecules (i.e., compounds that differ only in the position of the azide) may contribute to our ability to predict mycomembrane permeation and functional associations with whole cell activity."

Referee #2: mycobacteria, membrane biology

Reviewer #2 (Remarks to the Author):

In this work Lepori, Liu, Evbarunegbe, Feng et al. present the most comprehensive report to date on the application of their permeability assay PAC-MAN, which uses covalent trapping of azide test compounds by alkynes metabolically incorporated into peptidoglycan, to the determination of small molecule accumulation in mycobacteria, with a focus on M. tuberculosis (Mtb). Building on their recent work examining chemical features that modulate dye, antibiotic and peptide accumulation in the cell wall, the authors here present the screening of a far larger library of azide test compounds (>1500) and use these results to develop a predictive model.

This work stands apart from very recently reported complementary work on antibiotic accumulation in M. abscessus due to its far higher degree of generalizability and applicability. Crucially, the authors identify and validate chemical features that (dis)favor accumulation in their

assay and show via the analysis of existing (non-azide-containing) datasets and their own test compound SAR series that these relationships correlate with whole-cell activity. This is a revolutionary first for a field that has a history of target-based inhibitors failing to yield whole-cell activity and inhibitor optimization based on unvalidated assumptions regarding chemical features that promote permeation. This study provides among the first guidance that is based on experimental evidence; importantly, it also introduces and emphasizes accessible tools in the publicly available MycoPermeNet predictive model and the PACMAN assay, which, although it requires azide-modified compounds, is still on the whole more tractable than more widely reported but far more expensive mass spectrometry methods.

The study is especially commendable for its comprehensiveness and rigor and the strong dialogue between experiment and modeling. For example, the authors provide quantitative comparisons to analogous studies in E. coli and P. aeruginosa (showing that they do not predict accumulation in Mtb) and a negative control for their predictions by using data from not only from whole-cell screens, but also enzyme target-based screens where permeation is irrelevant. They also have the crucial insight to segment their results by chemical context (i.e., scaffold) rather than only analyzing the entire set for physicochemical correlates, as is more widely done. This makes their findings far more specific and useful with respect to chemical changes that promote permeation, as demonstrated in their two small molecule series highlighting Trp-Phe substitution and the introduction of nitrogen-containing heterocycles. Overall, the combination of informed (myco)bacterial perspective and deep chemical understanding makes this study an exemplar in this burgeoning area of predictive modeling for antibiotic development and optimization; we can only hope that similar teams come together to tackle related areas. Only a very few, very minor comments on a well written and presented manuscript that is set to be landmark study in the field.

MINOR COMMENTS

Rev 2 Q1. General comment: For the non-expert the coverage of the computation analysis and model building may be especially difficult to navigate and could benefit from more explanatory context, although we recognize that there may not be space to explain terms and provide background, in terms of norms that dictate methodological choices.

Thank you for pointing this out. Per reviewer suggestions, we have reworked the entire manuscript to enhance clarity and to make our findings more accessible to a microbiology audience without sacrificing necessary details, e.g., **line 227-231**:

"*Because neural networks have large numbers of parameters, additional techniques are needed to determine what changes in the input data will lead to changes in the output predictions. In our case, the input data are chemical structures, which are meaningful individually but difficult to summarize across our large and diverse dataset*."

Rev 2 Q2. Line 152: "When analyzed as a whole, our Mtb dataset did not reveal obvious

correlations." The authors generally also summarize their results on Msm throughout, but not here. Was the same true for the Msm dataset?

Yes, in both Msm and Mtb, the effects of physicochemical properties were scaffold-dependent, and generally in similar directions (compare new **Fig. S3b** [Msm data; below on the left] and **Fig. 2c** [Mtb data; below on the right, and in **Summary** section above]).

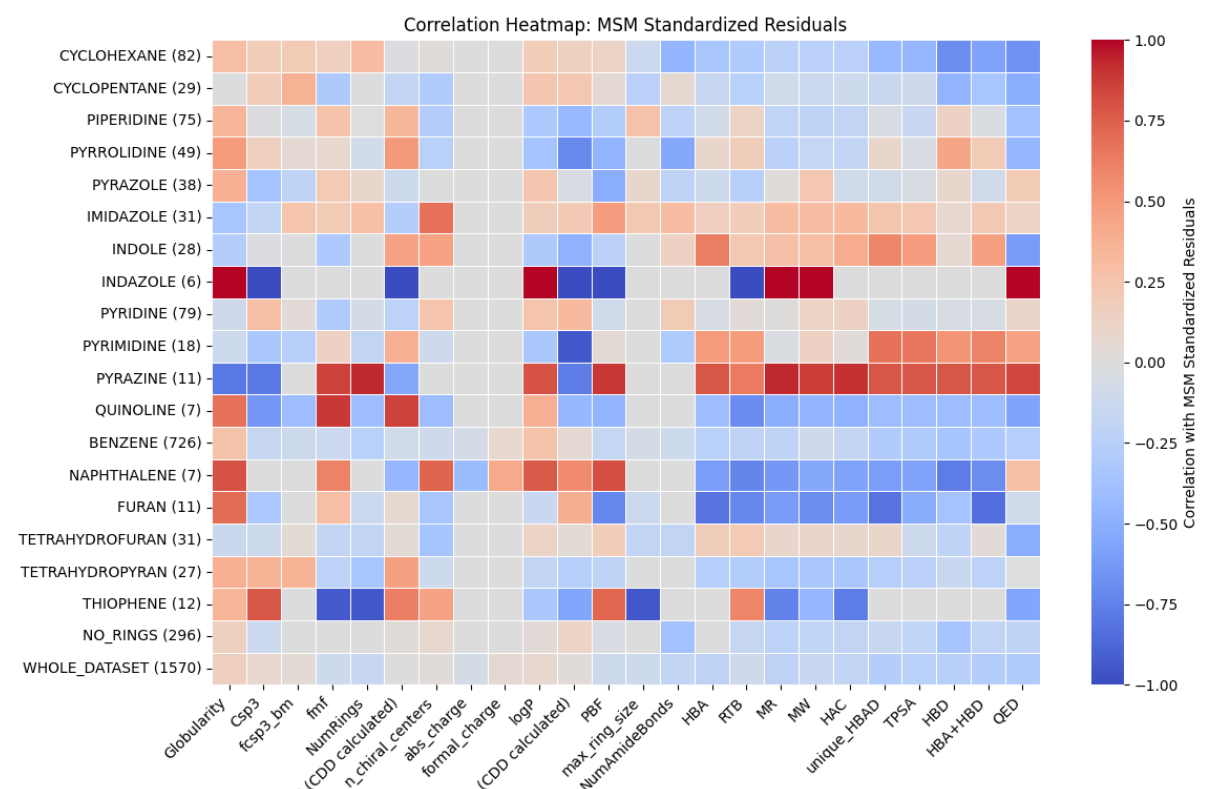

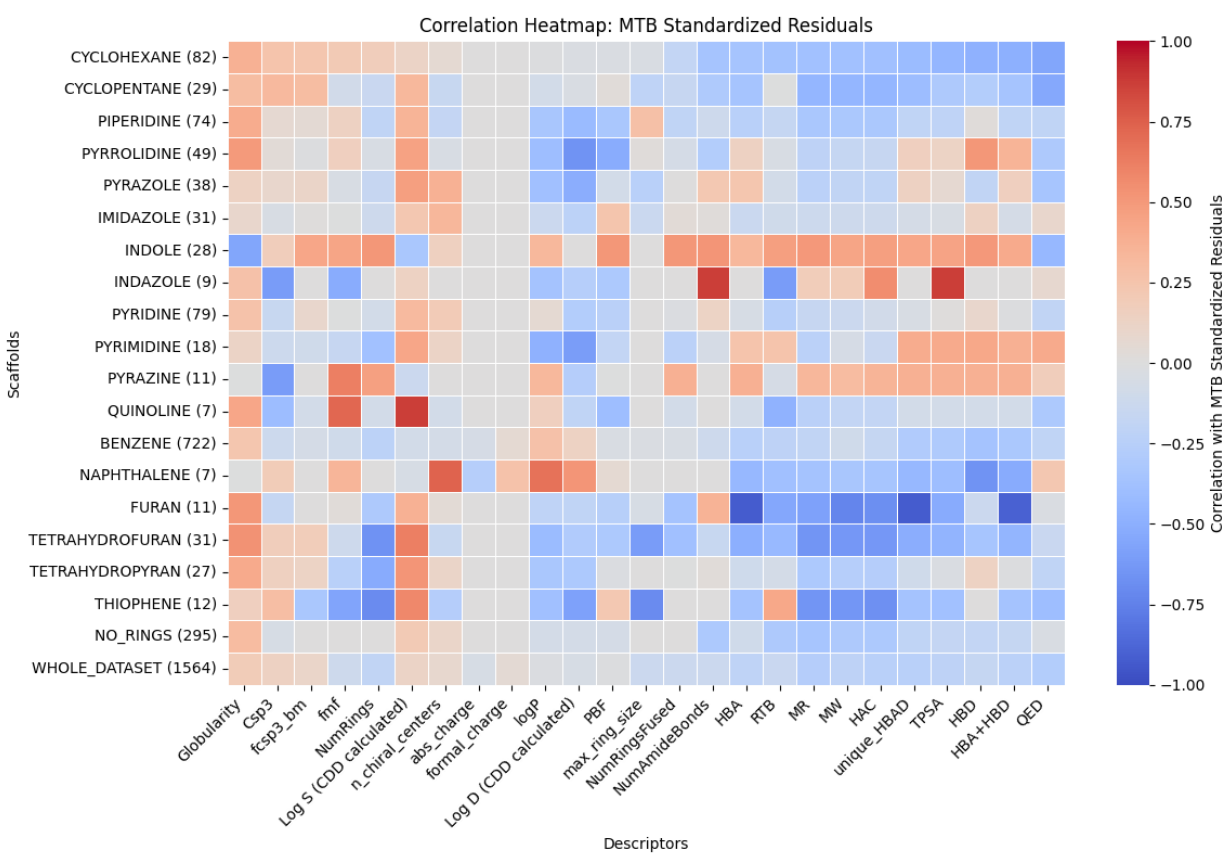

We have also updated the text to encompass these observations **line 164-165** we hope that the information will be useful for those who use Msm as a model organism:

*"We next investigated the correlation between mycomembrane permeation and physicochemical properties (**Fig. 2c** for Mtb and **S3b** for Msm)."*

Rev 2 Q3. Line 317-2138: "PAC-MAN primarily measures mycomembrane permeation, e.g., passive diffusion and facilitated transport" It is not clear from the cited studies what the evidence is that other processes (plasma membrane transport, as yet unknown mechanisms of mycomembrane export) do not also contribute or, at least, what assumptions are made by this statement (e.g., that chemical reactivity is appreciably faster than any process by which the test compound would exit the cell wall). Could the authors please clarify?

We based this claim in part on results from a previous paper [4], where we found that competition of a subset of our azide-modified antibiotics increased upon genetic (*M. smegmatis*) or chemical (*M. tuberculosis*) mycomembrane perturbation, and in part on the assumption that SPAAC-mediated anchoring of test molecules makes PAC-MAN less sensitive to in/out cycles (please see our response to **Rev 3 Q4**). The reviewer is correct, however, that we have not actually tested perturbations other than those to the mycomembrane, nor do we know the relative rates of SPAAC reaction vs. inner membrane permeation and/or efflux across either of the membranes. In the future we'd like to test these ideas by performing PAC-MAN +/- efflux mutants/inhibitors and by translating a cytoplasm-specific readout that we recently developed for *E. coli*[8]; recently accepted) into mycobacterial species. We have updated the Discussion, now **lines 509-513**, to capture this nuance.

"PAC-MAN covalently traps test molecules in the periplasm and is sensitive to genetic and chemical perturbations to the mycomembrane. Therefore, we speculate that the assay primarily measures one aspect of accumulation, mycomembrane permeation, although the slow reaction kinetics of SPAAC (second order rate constant of 0.2-0.5 $M^{-1}s^{-1}$) may also permit contributions from inner membrane permeation and efflux processes."

Reference 4: Lepori, I.; Jackson, K.; Liu, Z.; Chordia, M. D.; Wong, M.; Rivera, S. L.; Roncetti, M.; Poliseno, L.; Freundlich, J. S.; Pires, M. M.; Siegrist, M. S., The Mycomembrane Differentially and Heterogeneously Restricts Antibiotic Permeation. *ACS Infect Dis* **2025,** *11* (7), 1893-1906.

Reference 8: Ongwae, G. M.; Liu, Z.; Feng, S.; Chordia, M. D.; Sharifian Gh, M.; Dash, R.; Dalesandro, B. E.; Guo, T.; Sharpless, K. B.; Dong, J.; Siegrist, M. S.; Im, W.; Pires, M. M., Click-Based Determination of Accumulation of Molecules in Escherichia coli. *bioRxiv* **2025**.

Referee #3: chemistry, antibiotic development, antibiotic accumulation

Reviewer #3 (Remarks to the Author):

This manuscript by Lepori et al takes on an important problem, that of trying to discern what chemical features might allow a would-be antibiotic to permeate into Mtb. Given the many differences between the membranes of Mtb and other bacteria, it is critical to determine Mtb-specific permeation traits.

I have read this manuscript closely and carefully considered its message. I do think the long-term goal is highly meritorious, and the work could potentially be of high interest. As of right now, however, there are questions about the method and what it is reading out. And more critically, there is no real conclusion about chemical traits and permeation in Mtb (for example, neither the abstract or discussion mentions anything that could guide drug development). The manuscript indeed is much more computationally oriented and in its present form would be better suited to a computational journal. Modification for Nature Microbiology would require significant re-thinking and re-casting of this work, and further experiments. Specific comments:

We have addressed concerns, including those of Reviewer 3, with new experiments (**Fig. 5, S7, S8**, see **Summary** section above, and updates to **Fig. S8a**, **Fig. S6d**) and extensive text changes (marked in blue in main text). For details, please see below.

Rev 3 Q1. The hypothesis that Mtb permeation is a key determinant of whole cell anti-Mtb activity (mentioned a couple times) is self-evident and strikes as a straw man.

We were interested in an idea that is often repeated but rarely directly tested, which is that mycobacteria are intrinsically resistant to antibacterials because they have a unique, difficult-to-permeate outer membrane. While it is of course true that in most cases a molecule must permeate a cell in order to have whole cell activity, we and others have shown for mycobacteria

that the whole cell activities of some (but not all) antibacterials are enhanced by chemical and genetic perturbations to the mycobacterial cell envelope([4, 9, 10] and new **Fig. 5e**, see **Summary** section above). Where we have looked, the competition levels of the corresponding, azide-modified drugs in the PAC-MAN assay follow suit[4]. To us, these and other data suggest that the mycomembrane is a selective permeability and activity barrier. In this manuscript, we aim to understand the molecular basis and importance of mycomembrane selectivity. We appreciate the reviewer's candor and have updated the abstract, introduction, and discussion accordingly.

One example from the abstract:

Previous version: "Our data indicate that the mycomembrane is indeed a significant barrier to whole cell activity in Mtb and provide a rational framework for designing more effective compounds.

Updated version: "Our data suggest a rational framework for improving mycomembrane permeation and whole cell activity of antituberculars."

One example from the introduction:

Previous version: "Our work supports the long-standing hypothesis that the mycomembrane is a major barrier to whole cell activity in Mtb and suggests that improving the ability of a molecule to permeate the mycomembrane may aid the rational design or redesign of more effective drugs for tuberculosis."

Updated version: "Our work demonstrates that the mycomembrane is a major, albeit selective barrier to whole cell activity in Mtb and suggests that improving the ability of a molecule to permeate the mycomembrane can aid the rational design or redesign of more effective drugs for tuberculosis."

Reference 4: Lepori, I.; Jackson, K.; Liu, Z.; Chordia, M. D.; Wong, M.; Rivera, S. L.; Roncetti, M.; Poliseno, L.; Freundlich, J. S.; Pires, M. M.; Siegrist, M. S., The Mycomembrane Differentially and Heterogeneously Restricts Antibiotic Permeation. *ACS Infect Dis* **2025,** *11* (7), 1893-1906.

Reference 9: Li, S.; Poulton, N. C.; Chang, J. S.; Azadian, Z. A.; DeJesus, M. A.; Ruecker, N.; Zimmerman, M. D.; Eckartt, K. A.; Bosch, B.; Engelhart, C. A.; Sullivan, D. F.; Gengenbacher, M.; Dartois, V. A.; Schnappinger, D.; Rock, J. M., CRISPRi chemical genetics and comparative genomics identify genes mediating drug potency in Mycobacterium tuberculosis. *Nat Microbiol* **2022,** *7* (6), 766-779.

Reference 10: Sarathy, J.; Dartois, V.; Dick, T.; Gengenbacher, M., Reduced drug uptake in phenotypically resistant nutrient-starved nonreplicating Mycobacterium tuberculosis. *Antimicrob Agents Chemother* **2013,** *57* (4), 1648-53.

Rev 3 Q2. Mtb is well known to process would-be antibiotics as they make their way into the cell, so it is striking that there is no mention of this possibility of drug modification and how it might complicate the interpretation of their results.

We agree that this is an important point. We (J.S.F.) and others have reported on many examples of intrabacterial metabolism of small molecule antitubercular agents within *M. tuberculosis*[11-14]. We think the time window of our assay (2-hour exposure to azide-modified molecules) may limit intrabacterial metabolism as we tend to see this phenomenon in *M. tuberculosis* starting after 2 hours, e.g., [11]. We have touched on the potential for metabolic transformation in a previous paper[4] but agree it is helpful to the reader to articulate this and other limitations of our assay more clearly, which we now do in **lines 499-505**:

"Intrabacterial metabolism presents a challenge for any method measuring molecule accumulation in live cells. For PAC-MAN, modification or removal of the azide would prevent covalent trapping by SPAAC and thus our ability to detect the molecule, while transformations farther from the azide could alter permeability in more complex ways. We speculate that the two-hour window of molecule exposure may limit intrabacterial metabolism, as we have observed that transformation of other molecules in Mtb begins after two hours."

Reference 4: Lepori, I.; Jackson, K.; Liu, Z.; Chordia, M. D.; Wong, M.; Rivera, S. L.; Roncetti, M.; Poliseno, L.; Freundlich, J. S.; Pires, M. M.; Siegrist, M. S., The Mycomembrane Differentially and Heterogeneously Restricts Antibiotic Permeation. *ACS Infect Dis* **2025,** *11* (7), 1893-1906.

Reference 11: Wang, X.; Inoyama, D.; Russo, R.; Li, S. G.; Jadhav, R.; Stratton, T. P.; Mittal, N.; Bilotta, J. A.; Singleton, E.; Kim, T.; Paget, S. D.; Pottorf, R. S.; Ahn, Y. M.; Davila-Pagan, A.; Kandasamy, S.; Grady, C.; Hussain, S.; Soteropoulos, P.; Zimmerman, M. D.; Ho, H. P.; Park, S.; Dartois, V.; Ekins, S.; Connell, N.; Kumar, P.; Freundlich, J. S., Antitubercular Triazines: Optimization and Intrabacterial Metabolism. *Cell Chem Biol* **2020,** *27* (2), 172-185.e11.

Reference 12: Wang, X.; Perryman, A. L.; Li, S. G.; Paget, S. D.; Stratton, T. P.; Lemenze, A.; Olson, A. J.; Ekins, S.; Kumar, P.; Freundlich, J. S., Intrabacterial Metabolism Obscures the Successful Prediction of an InhA Inhibitor of Mycobacterium tuberculosis. *ACS Infect Dis* **2019,** *5* (12), 2148-2163.

Reference 13: Li, S. G.; Vilchèze, C.; Chakraborty, S.; Wang, X.; Kim, H.; Anisetti, M.; Ekins, S.; Rhee, K. Y.; Jacobs, W. R., Jr.; Freundlich, J. S., Evolution of a thienopyrimidine antitubercular relying on medicinal chemistry and metabolomics insights. *Tetrahedron Lett* **2015,** *56* (23), 3246-3250.

Reference 14: Awasthi, D.; Freundlich, J. S., Antimycobacterial Metabolism: Illuminating Mycobacterium tuberculosis Biology and Drug Discovery. *Trends Microbiol* **2017,** *25* (9), 756-767.

Rev 3 Q3. A strength of this work is the ability to evaluate a multitude of different looking compounds, since the authors are untethered from antibacterial activity.

Evaluating compounds that have activity has indeed been trickier than evaluating those without activity. When we do attempt the latter, we include experiments with unmodified, non-azide derivatives, for activity assays ([4] and new data with octyl-tridecaptins **Fig. 5j**, see **Summary** section above) and/or for PAC-MAN, as controls against which we normalize total fluorescence (e.g., **Figs. 4b-c, Fig 5c, Fig S7b**, see **Summary** section above). In the second approach, we acknowledge that unmodified, non-azide derivatives are not perfect controls (since, as we discuss in **Rev 1 Q7**, the azide modification may impact activity), but we think that they can be informative in concert with other control experiments.

Reference 4: Lepori, I.; Jackson, K.; Liu, Z.; Chordia, M. D.; Wong, M.; Rivera, S. L.; Roncetti, M.; Poliseno, L.; Freundlich, J. S.; Pires, M. M.; Siegrist, M. S., The Mycomembrane Differentially and Heterogeneously Restricts Antibiotic Permeation. *ACS Infect Dis* **2025,** *11* (7), 1893-1906.

Rev 3 Q4. The authors seem to have done quite a bit of validation of their permeation competition assay. The biggest challenge with using this assay to draw conclusions is that positive compounds covalently append themselves to the bacteria, so, the natural cycle of in/out that would occur for (non-covalent) antibiotics is bypassed. This assay can then be viewed as good for assessing penetration, but less good (and perhaps poor) for truly assessing accumulation, which is the crux of the drug discovery problem. The authors do mention this caveat in the Discussion; but, it is a big one.

Given that SPAAC is a rather slow reaction, with a second order rate constant of 0.2-0.5 $M^{-1}s^{-1}$ [15, 16] we do not yet know whether some in/out occurs. We agree with the reviewer that PAC-MAN likely mostly queries one aspect of accumulation (mycomembrane permeation) but, as we responded to **Rev 2 Q3** and now address in **lines 508-513**, we have not directly tested this assumption.

"PAC-MAN covalently traps test molecules in the periplasm and is sensitive to genetic and chemical perturbations to the mycomembrane. Therefore, we speculate that the assay primarily measures one aspect of accumulation, mycomembrane permeation, although the slow reaction kinetics of SPAAC (second order rate constant of 0.2-0.5 $M^{-1}s^{-1}$) may also permit contributions from inner membrane permeation and efflux processes."

Accumulation is a complex phenotype, requiring a molecule to cross membrane(s) and avoid efflux and metabolic transformation. Several landmark studies have used mass spectrometry to capture this process in its entirety, e.g., [17-19], and very recently, [20]. While in some cases, descriptors could be identified that correlate with or drive accumulation, in other cases there were no single and/or implementable features. The complexity is likely driven by chemical space, bacterial factors, and the interactions between them. To simplify the problem, many

groups have opted to investigate specific families of molecules and/or bacteria with defined defects in the outer membrane and/or efflux. We think that compartment-specific assays like PAC-MAN represent an orthogonal approach to isolating variables that are important to accumulation. Beyond enabling subcellular localization, covalent trapping by PAC-MAN also avoids technical problems associated with non-specific association, e.g., with the mycomembrane, and loss of the molecule during sample processing.

We are currently translating a cytoplasm-specific readout from *E. coli* ([8]; recently accepted) to *M. tuberculosis* and non-tuberculous mycobacteria (NTMs). We have also established collaborations to screen accumulation in these species by mass spectrometry, both from whole cells and from spent supernatants. We think the four assays–periplasm click, cytoplasm click, LC-MS in whole cells, and LC-MS in supernatants–have complementary strengths (see also **Rev 3 Q6**) and report overlapping but distinct phenotypes. By using multiple assays +/- genetic and chemical perturbations, our future goal is to build step-by-step models of how molecules transit into and out of mycobacterial cells.

Beyond enabling us and others to investigate fundamental biological processes (we are particularly interested in what *M. tuberculosis* eats and how it recycles components of their cell envelope) we anticipate that breaking down the problem of accumulation into its constituent parts (then building back up again) will aid antitubercular agent (re)design.

Our data strongly suggest that–while it is only one variable in accumulation–mycomembrane permeation alone is an important parameter for whole cell activity. We have shown that the mycomembrane can pose a permeability and activity barrier to both antibacterials with targets in the periplasm, e.g., vancomycin in [4] and octyl-tridecaptins that we now include in **Fig. 5**, as well as for those with targets in the cytoplasm, e.g., moxifloxacin in [4]. More broadly, we find good agreement between chemical predictors of mycomembrane permeation and whole Mtb cell activity (**Fig. 5** [see **Summary** section above], **Fig. 6, Fig. S9,** and **Fig. S10**).

Reference 8: Ongwae, G. M.; Liu, Z.; Feng, S.; Chordia, M. D.; Sharifian Gh, M.; Dash, R.; Dalesandro, B. E.; Guo, T.; Sharpless, K. B.; Dong, J.; Siegrist, M. S.; Im, W.; Pires, M. M., Click-Based Determination of Accumulation of Molecules in Escherichia coli. *bioRxiv* **2025**.

Reference 15: Rigolot, V.; Biot, C.; Lion, C., To View Your Biomolecule, Click inside the Cell. *Angew Chem Int Ed Engl* **2021,** *60* (43), 23084-23105.

Reference 16: Dommerholt, J.; van Rooijen, O.; Borrmann, A.; Guerra, C. F.; Bickelhaupt, F. M.; van Delft, F. L., Highly accelerated inverse electron-demand cycloaddition of electron-deficient azides with aliphatic cyclooctynes. *Nat Commun* **2014,** *5*, 5378.

Reference 17: Richter, M. F.; Drown, B. S.; Riley, A. P.; Garcia, A.; Shirai, T.; Svec, R. L.; Hergenrother, P. J., Predictive compound accumulation rules yield a broad-spectrum antibiotic. *Nature* **2017,** *545* (7654), 299-304.

Reference 18: Leus, I. V.; Weeks, J. W.; Bonifay, V.; Shen, Y.; Yang, L.; Cooper, C. J.; Nath, D.; Duerfeldt, A. S.; Smith, J. C.; Parks, J. M.; Rybenkov, V. V.; Zgurskaya, H. I., Property space mapping of Pseudomonas aeruginosa permeability to small molecules. *Sci Rep* **2022,** *12* (1), 8220.

Reference 19 :Geddes, E. J.; Gugger, M. K.; Garcia, A.; Chavez, M. G.; Lee, M. R.; Perlmutter, S. J.; Bieniossek, C.; Guasch, L.; Hergenrother, P. J., Porin-independent accumulation in Pseudomonas enables antibiotic discovery. *Nature* **2023,** *624* (7990), 145-153.

Reference 20: Le Goff, F.; Hazemann, J.; Christen, L.; Bourquin, G.; Pierlot, G.; Lange, R.; Panchaud, P.; Zumbrunn, C.; Peter, O.; Rueedi, G.; Ritz, D., Measurement and prediction of small molecule retention by Gram-negative bacteria based on a large-scale LC/MS screen. *Sci Rep* **2025,** *15* (1), 25431.

Rev 3 Q5. A weakness in the ML workflow is on the bottom of page 12, hand selection of 26 chemical features. This is a small fraction of the features that are available.

The ML model based on descriptors was created to interpret the predictions from MycoPermeNet and provide information readable by medicinal chemists. Our goal is to help guide structural changes to compounds of interest, previously characterized for SAR, to improve mycomembrane permeability without impacting target engagement.

With this goal in mind, we selected 25 physicochemical properties (CNS MPO was included by mistake and has been now removed) that have been previously shown to be important for Gram-negative accumulation[17-19, 21] and can be easily interpreted by experienced chemists and translated into drug modifications. Gratifyingly, since posting our manuscript on bioRxiv, another paper has used our findings to design a new antitubercular[22].

Before using this approach, we initially developed the method using ~1060 descriptors. The model had a similar performance as the one that employs 25 features but was impossible to read and translate into chemical modifications of a molecule by our team of experienced chemists. Furthermore, the redundancy of the descriptors led to the loss of important information as multiple top spots were occupied by very similar descriptors. We provide below an example of the interpretability graph for peptide **W1** (used in **Fig. 4e-h**) generated using the model trained with ~1060 descriptors. The obtained outcome conflicted with our goal of readability and accessibility, so we opted for the clearer, 25-descriptor model.

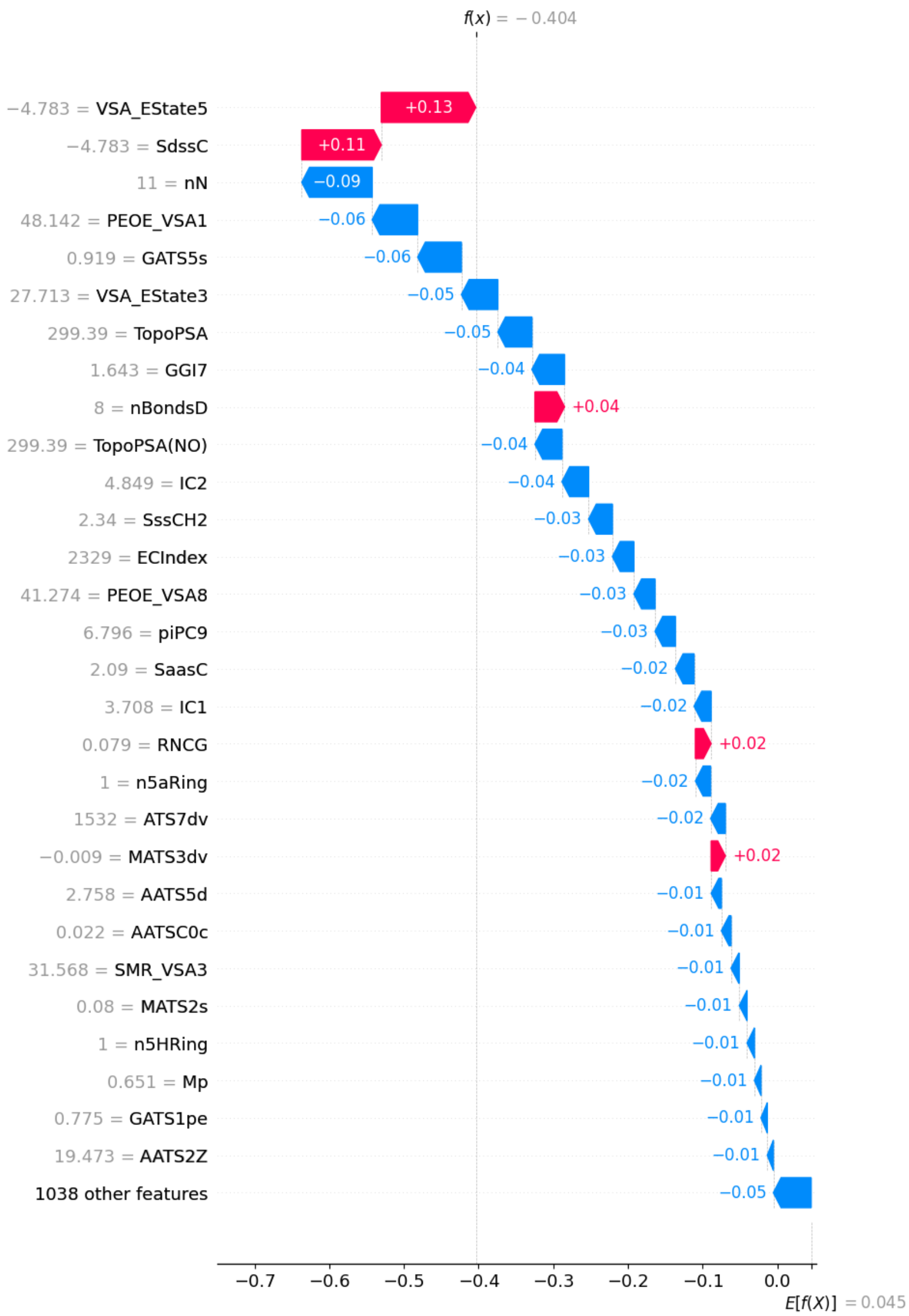

Example of explanation of descriptors with limited practical applicability:

1. VSA_Estate5: VSA (Van der Waals Surface Area) E-State Index 5: This descriptor combines Van der Waals surface area and electrotopological state (E-State) indices. It represents the sum of E-State values for atoms with a particular VSA range, corresponding to category 5 in a predefined range of atomic surface areas.

2. SdssC: Sum of Distance Sum Connectivity: This is a topological descriptor that sums up the distance between atom pairs, weighted by their connectivity. It provides information about the overall topological structure of the molecule.

Reference 17: Richter, M. F.; Drown, B. S.; Riley, A. P.; Garcia, A.; Shirai, T.; Svec, R. L.; Hergenrother, P. J., Predictive compound accumulation rules yield a broad-spectrum antibiotic. *Nature* **2017,** *545* (7654), 299-304.

Reference 18: Leus, I. V.; Weeks, J. W.; Bonifay, V.; Shen, Y.; Yang, L.; Cooper, C. J.; Nath, D.; Duerfeldt, A. S.; Smith, J. C.; Parks, J. M.; Rybenkov, V. V.; Zgurskaya, H. I., Property space mapping of Pseudomonas aeruginosa permeability to small molecules. *Sci Rep* **2022,** *12* (1), 8220.

Reference 19: Geddes, E. J.; Gugger, M. K.; Garcia, A.; Chavez, M. G.; Lee, M. R.; Perlmutter, S. J.; Bieniossek, C.; Guasch, L.; Hergenrother, P. J., Porin-independent accumulation in Pseudomonas enables antibiotic discovery. *Nature* **2023,** *624* (7990), 145-153.

Reference 21: Davis, T. D.; Gerry, C. J.; Tan, D. S., General platform for systematic quantitative evaluation of small-molecule permeability in bacteria. *ACS Chem Biol* **2014,** *9* (11), 2535-44.

Reference 22: Kumar, T. A.; Birua, S.; Mallojjala, S. C.; Mukherjee, P.; Singh, S.; Kaul, G.; Ramachandran, A.; Akhir, A.; Chopra, S.; Gadgil, C. J.; Hirschi, J. S.; Singh, A.; Chakrapani, H., A Mycobacteria-Specific Prodrug to Overcome Phenotypic AMR in Mycobacterium tuberculosis. *J Med Chem* **2025**.

Rev 3 Q6. For Figure 4, testing within a series, it is hard to know how meaningful the differences are in terms a microbiologist/drug researcher might understand. What is the cellular concentration difference between a compound with a deltaLogCC50 of 1.25 and one with a value of 2 (for example)?

We are still figuring this out. In theory, we could compare our measurements, e.g., **W** peptides in **Fig. 4e-h** and **JSF-2985** in **Fig. 4a-d**, to LC-MS analyses of whole bacteria. Given that PAC-MAN queries the periplasm, and LC-MS measures whole cell accumulation, we do not think that this comparison alone is an accurate way to assess. As mentioned above in **Rev 3 Q4**, we have established collaborations to do (more) LC-MS for whole cells as well as for spent supernatants. By subtracting what's associated with cells and what's left in the supernatant after PAC-MAN from the starting concentration of the test molecule, we may be able to map $\Delta log_{10}CC_{50}$ to biologically meaningful concentrations.

Lack of absolute quantitation may be a limitation for some applications of PAC-MAN. However, for our current work, we think a comparative approach is sufficient to achieve our goal, i.e., identify chemical features that correlate with and/or promote mycomembrane permeation.

Rev 3 Q7. A major weakness is not validating any of the results with the LC/MS/MS method which is now standard in the field.

Initially we did not compare LC-MS and PAC-MAN because of the inherent differences in what we think the two assays are reading out, i.e. whole cell accumulation/association[23] and mycomembrane permeation, respectively. Instead, we validated our results via other orthogonal methods, for example:

- In [4], we show good concordance between the permeation of azide drugs and activity of parent (non-azide) drugs +/- different mycomembrane perturbations.

- In this manuscript, we show that chemical features that correlate with and/or promote mycomembrane permeation also correlate with and/or promote whole cell activity (**Fig. 5** and **Fig. 6**). The former were identified using azide-modified compounds and the latter assessed with unmodified (non-azide) compounds.

However, we agree that LC-MS is a standard method for measuring accumulation in Gram-negatives, and that it is natural for a reader to want to know how our assay compares. We now show LC-MS for two experiments, one to compare accumulation of a compound +/- azide (**Fig. S8a**, see **Summary** section above) and the other to test the effect of the indole on permeation/accumulation of model **W** peptide (**Fig. S6d**, see **Summary** section above). In the latter case, there was good agreement with ML predictions (**Fig. S6c**, see **Summary** section above) and experimental PAC-MAN data (**Fig. S6a-b**, see **Summary** section above), although we acknowledge the limited sample size and chemical diversity.

Reference 23: Evaluating LC−MS/MS To Measure Accumulation of Compounds within Bacteria Ramkumar Iyer, Zhengqi Ye, Annette Ferrari, Leonard Duncan, M. Angela Tanudra, Hong Tsao,Tiansheng Wang, Hong Gao, Christopher L. Brummel, and Alice L. Erwin

Reference 4: Lepori, I.; Jackson, K.; Liu, Z.; Chordia, M. D.; Wong, M.; Rivera, S. L.; Roncetti, M.; Poliseno, L.; Freundlich, J. S.; Pires, M. M.; Siegrist, M. S., The Mycomembrane Differentially and Heterogeneously Restricts Antibiotic Permeation. *ACS Infect Dis* **2025,** *11* (7), 1893-1906.

Rev 3 Q8. Overall, the authors present an interesting workflow for studying penetrance into Mtb. But, it is hard to make heads or tails of the data. It is hard to know what has been learned, perhaps best evident by the fact that the authors have not been able to apply any of the learnings to convincingly lead to compounds that accumulate better in Mtb and have better antibacterial activity.

Our study suggests that the correlates of mycomembrane permeability are not simple, as physicochemical properties have scaffold-dependent effects. However, we do see that scaffolds alone are predictive, even without additional physicochemical descriptors, and that the indole scaffold performs particularly well, predicting both mycomembrane permeation and whole cell activity. To bring these findings to the fore, we have:

- Tested effect of benzene to indole substitution on model **W** peptide accumulation via LC-MS in **Fig. S6d** (new experiment, see **Summary** section above)

- Tested the effect of benzene to indole substitution on predicted and observed octyl-tridecaptin permeability via ML (**Fig. 5b**) and PAC-MAN (**Fig. 5c**). These are all new experiments (see **Summary** section above)

- Tested the effect of benzene to indole substitution on octyl-tridecaptin activity against Mtb in **Fig. 5j** and against Msm in **Fig. S7c** (new experiments, see **Summary** section above)

- Highlighted the association of indole in two larger molecule sets with ML-predicted permeation and observed whole cell activity (**Fig. 6a-b, Fig. S9a**)

- Reworked the manuscript to enhance clarity and utility, including more focus on scaffolds in general and indole specifically as permeability- and activity-promoting moieties (e.g., **lines 50-53; 107-110; 157-163; 429-438** and new experiments in **Fig. 5** and **S7** [see the end of the **Summary** section for full figures] and related headings/text).

"Chemical predictors of mycomembrane permeation include scaffolds like indole and can promote anti-Mtb activity. Our data suggest a rational framework for improving mycomembrane permeation and whole cell activity of antituberculars."

and

"Finally, we showed that scaffolds like aromatic nitrogen heterocycles, especially indole, and other chemical predictors of mycomembrane permeation can promote anti-Mtb activity in different molecular contexts."

and

*"For both Msm and Mtb, we found that aromatic nitrogen-containing scaffolds like indole, imidazole, or pyrazole correlate positively with permeation (negative medians for standardized residuals), while scaffolds like cyclopentane or cyclohexane correlate negatively (positive medians). Across our analyses, aromatic scaffolds, especially nitrogen heterocycles, positively associate with permeation while non-aromatic ring systems, including some heterocycles, negatively associate with permeation (**Fig. 2a**, **Fig. 2b, Fig. S3a**)."*

and

"Here we find that the effects of physicochemical properties on mycomembrane permeation are scaffold-dependent. Furthermore, scaffolds alone, particularly aromatic nitrogen heterocycles like indole, associate with and/or promote mycomembrane permeation in different molecular contexts. In pharmacology, indole is considered a privileged scaffold because it is highly

represented across drug classes, can effectively engage with a variety of targets, and has useful chemical reactivity that allows versatile substitutions for derivatization. At present, several compounds under exploration as anti-Mtb drugs are indole-based. Our data suggest that, for mycobacterial species, indole may also be a privileged scaffold in terms of cell permeation."

As the reviewer will note, we tried to tune the mycomembrane permeation and whole cell activity for two sets of compounds, one based on **JSF-2985** and the other on an octyl-tridecaptin. We were largely able to achieve the first goal with both sets, but the second goal only with the octyl-tridecaptin. We have two leading hypotheses for why this might be, not mutually exclusive: a) **JSF-2985** (309 Da) is much smaller than octyl-tridecaptin (1521 Da), which makes it difficult to add permeability-tuning moieties without impacting target engagement and b) the mycomembrane is a barrier to whole cell activity for octyl-tridecaptin but not **JSF-2985** (**Fig. 5e**, see **Summary** section above).

While we think these hypotheses are reasonable explanations for our failure to rationally tune **JSF-2985** activity and success with octyl-tridecaptin, we are hesitant to make broad claims from n=2 molecule series. It is for that reason that we close the manuscript with what we think are the more robust and compelling data, namely, the association between chemical predictors of mycomembrane permeation–which we define experimentally and computationally in this work–and whole cell (but not in vitro on purified enzyme) activity in large datasets.

We also now discuss applications of our learning both on the small scale (derivatization of lead compounds) and on the large scale (prioritization before or after screening for activity) in the Discussion, **lines 440-464**. As well, we note that a very recent anti-Mtb application[22] was supported by our preprinted work.

*"The identification of chemical features that influence mycomembrane permeation has the potential to benefit drug discovery efforts in several ways. On the small scale, MycoPermeNet and similar ML models may help guide derivatization of lead compounds. Our work here suggests that the utility of such models will depend on several factors. First, the mycomembrane is not always a significant barrier for anti-Mtb antimicrobials (**Fig. 5e**). Second, even for molecules for which the mycomembrane poses a major barrier to uptake, improving permeation via derivatization may come at the cost of other aspects of whole cell activity, namely, target engagement and susceptibility to metabolism and efflux. For example, N-methylation enhances observed and predicted mycomembrane permeation of the octyl-tridecaptins in Msm and Mtb but activity only against Msm (**Fig. 5b-c, Fig. 5j, S7b**). By contrast, benzene to indole substitution can enhance permeation and activity of these derivatives in both species (**Fig. 5b, 5c, 5j, S7c**). Third, we envision that interpretability studies of individual compounds (**Fig. 3f**) will be important for tailoring permeability-promoting modifications. For example, smaller molecules may be more amenable to subtle changes in physicochemical properties like TPSA and logP than to addition or subtraction of scaffolds, which are relatively large modifications. Beyond derivatization of individual compounds, on the large-scale, ML models for mycomembrane permeability could be applied to molecule collections before or after activity screening to prioritize candidates more efficiently. In this work, for example, MycoPermeNet enabled us to*

identify robust correlation between chemical predictors of mycomembrane permeation and whole cell (but not in vitro, against a purified enzyme) activity in large datasets, a relationship that was more complicated to interpret for individual molecule series, e.g., ***JSF-2985*** *(**Fig. 4c, 4d, S5**).”*

Reference 22: Kumar, T. A.; Birua, S.; Mallojjala, S. C.; Mukherjee, P.; Singh, S.; Kaul, G.; Ramachandran, A.; Akhir, A.; Chopra, S.; Gadgil, C. J.; Hirschi, J. S.; Singh, A.; Chakrapani, H., A Mycobacteria-Specific Prodrug to Overcome Phenotypic AMR in Mycobacterium tuberculosis. *J Med Chem* **2025**.

Complete list of references:

1. Seal, S.; Carreras-Puigvert, J.; Trapotsi, M. A.; Yang, H.; Spjuth, O.; Bender, A., Integrating cell morphology with gene expression and chemical structure to aid mitochondrial toxicity detection. *Commun Biol* **2022,** *5* (1), 858.
2. Moradi, H.; Bunnell, H. T.; Price, B. S.; Khodaverdi, M.; Vest, M. T.; Porterfield, J. Z.; Anzalone, A. J.; Santangelo, S. L.; Kimble, W.; Harper, J.; Hillegass, W. B.; Hodder, S. L., Assessing the effects of therapeutic combinations on SARS-CoV-2 infected patient outcomes: A big data approach. *PLoS One* **2023,** *18* (3), e0282587.
3. Puszkarska, A. M.; Taddese, B.; Revell, J.; Davies, G.; Field, J.; Hornigold, D. C.; Buchanan, A.; Vaughan, T. J.; Colwell, L. J., Machine learning designs new GCGR/GLP-1R dual agonists with enhanced biological potency. *Nat Chem* **2024,** *16* (9), 1436-1444.
4. Lepori, I.; Jackson, K.; Liu, Z.; Chordia, M. D.; Wong, M.; Rivera, S. L.; Roncetti, M.; Poliseno, L.; Freundlich, J. S.; Pires, M. M.; Siegrist, M. S., The Mycomembrane Differentially and Heterogeneously Restricts Antibiotic Permeation. *ACS Infect Dis* **2025,** *11* (7), 1893-1906.
5. Agard, N. J.; Prescher, J. A.; Bertozzi, C. R., A strain-promoted [3 + 2] azide-alkyne cycloaddition for covalent modification of biomolecules in living systems. *J Am Chem Soc* **2004,** *126* (46), 15046-7.
6. Jewett, J. C.; Bertozzi, C. R., Cu-free click cycloaddition reactions in chemical biology. *Chem Soc Rev* **2010,** *39* (4), 1272-9.
7. Kelly, J. J.; Dalesandro, B. E.; Liu, Z.; Chordia, M. D.; Ongwae, G. M.; Pires, M. M., Measurement of Accumulation of Antibiotics to Staphylococcus aureus in Phagosomes of Live Macrophages. *Angew Chem Int Ed Engl* **2024,** *63* (3), e202313870.
8. Ongwae, G. M.; Liu, Z.; Feng, S.; Chordia, M. D.; Sharifian Gh, M.; Dash, R.; Dalesandro, B. E.; Guo, T.; Sharpless, K. B.; Dong, J.; Siegrist, M. S.; Im, W.; Pires, M. M., Click-Based Determination of Accumulation of Molecules in Escherichia coli. *bioRxiv* **2025**.
9. Li, S.; Poulton, N. C.; Chang, J. S.; Azadian, Z. A.; DeJesus, M. A.; Ruecker, N.; Zimmerman, M. D.; Eckartt, K. A.; Bosch, B.; Engelhart, C. A.; Sullivan, D. F.; Gengenbacher, M.; Dartois, V. A.; Schnappinger, D.; Rock, J. M., CRISPRi chemical genetics and comparative genomics identify genes mediating drug potency in Mycobacterium tuberculosis. *Nat Microbiol* **2022,** *7* (6), 766-779.
10. Sarathy, J.; Dartois, V.; Dick, T.; Gengenbacher, M., Reduced drug uptake in phenotypically resistant nutrient-starved nonreplicating Mycobacterium tuberculosis. *Antimicrob Agents Chemother* **2013,** *57* (4), 1648-53.

11. Wang, X.; Inoyama, D.; Russo, R.; Li, S. G.; Jadhav, R.; Stratton, T. P.; Mittal, N.; Bilotta, J. A.; Singleton, E.; Kim, T.; Paget, S. D.; Pottorf, R. S.; Ahn, Y. M.; Davila-Pagan, A.; Kandasamy, S.; Grady, C.; Hussain, S.; Soteropoulos, P.; Zimmerman, M. D.; Ho, H. P.; Park, S.; Dartois, V.; Ekins, S.; Connell, N.; Kumar, P.; Freundlich, J. S., Antitubercular Triazines: Optimization and Intrabacterial Metabolism. *Cell Chem Biol* **2020,** *27* (2), 172-185.e11.

12. Wang, X.; Perryman, A. L.; Li, S. G.; Paget, S. D.; Stratton, T. P.; Lemenze, A.; Olson, A. J.; Ekins, S.; Kumar, P.; Freundlich, J. S., Intrabacterial Metabolism Obscures the Successful Prediction of an InhA Inhibitor of Mycobacterium tuberculosis. *ACS Infect Dis* **2019,** *5* (12), 2148-2163.

13. Li, S. G.; Vilchèze, C.; Chakraborty, S.; Wang, X.; Kim, H.; Anisetti, M.; Ekins, S.; Rhee, K. Y.; Jacobs, W. R., Jr.; Freundlich, J. S., Evolution of a thienopyrimidine antitubercular relying on medicinal chemistry and metabolomics insights. *Tetrahedron Lett* **2015,** *56* (23), 3246-3250.

14. Awasthi, D.; Freundlich, J. S., Antimycobacterial Metabolism: Illuminating Mycobacterium tuberculosis Biology and Drug Discovery. *Trends Microbiol* **2017,** *25* (9), 756-767.

15. Rigolot, V.; Biot, C.; Lion, C., To View Your Biomolecule, Click inside the Cell. *Angew Chem Int Ed Engl* **2021,** *60* (43), 23084-23105.

16. Dommerholt, J.; van Rooijen, O.; Borrmann, A.; Guerra, C. F.; Bickelhaupt, F. M.; van Delft, F. L., Highly accelerated inverse electron-demand cycloaddition of electron-deficient azides with aliphatic cyclooctynes. *Nat Commun* **2014,** *5*, 5378.

17. Richter, M. F.; Drown, B. S.; Riley, A. P.; Garcia, A.; Shirai, T.; Svec, R. L.; Hergenrother, P. J., Predictive compound accumulation rules yield a broad-spectrum antibiotic. *Nature* **2017,** *545* (7654), 299-304.

18. Leus, I. V.; Weeks, J. W.; Bonifay, V.; Shen, Y.; Yang, L.; Cooper, C. J.; Nath, D.; Duerfeldt, A. S.; Smith, J. C.; Parks, J. M.; Rybenkov, V. V.; Zgurskaya, H. I., Property space mapping of Pseudomonas aeruginosa permeability to small molecules. *Sci Rep* **2022,** *12* (1), 8220.

19. Geddes, E. J.; Gugger, M. K.; Garcia, A.; Chavez, M. G.; Lee, M. R.; Perlmutter, S. J.; Bieniossek, C.; Guasch, L.; Hergenrother, P. J., Porin-independent accumulation in Pseudomonas enables antibiotic discovery. *Nature* **2023,** *624* (7990), 145-153.

20. Le Goff, F.; Hazemann, J.; Christen, L.; Bourquin, G.; Pierlot, G.; Lange, R.; Panchaud, P.; Zumbrunn, C.; Peter, O.; Rueedi, G.; Ritz, D., Measurement and prediction of small molecule retention by Gram-negative bacteria based on a large-scale LC/MS screen. *Sci Rep* **2025,** *15* (1), 25431.

21. Davis, T. D.; Gerry, C. J.; Tan, D. S., General platform for systematic quantitative evaluation of small-molecule permeability in bacteria. *ACS Chem Biol* **2014,** *9* (11), 2535-44.

22. Kumar, T. A.; Birua, S.; Mallojjala, S. C.; Mukherjee, P.; Singh, S.; Kaul, G.; Ramachandran, A.; Akhir, A.; Chopra, S.; Gadgil, C. J.; Hirschi, J. S.; Singh, A.; Chakrapani, H., A Mycobacteria-Specific Prodrug to Overcome Phenotypic AMR in Mycobacterium tuberculosis. *J Med Chem* **2025**.

23. Iyer, R.; Ye, Z.; Ferrari, A.; Duncan, L.; Tanudra, M. A.; Tsao, H.; Wang, T.; Gao, H.; Brummel, C. L.; Erwin, A. L., Evaluating LC-MS/MS To Measure Accumulation of Compounds within Bacteria. *ACS Infect Dis* **2018,** *4* (9), 1336-1345.

Reviewer #2 (Remarks to the Author):

All of our minor comments were addressed. We look forward to seeing this work finally published as is well deserved.

Thank you!

Reviewer #3 (Remarks to the Author):

There are many improvements in this new version of the manuscript. It is a good start toward getting at the vexing Mtb permeation/accumulation challenge. There are still weaknesses – most notably the lack of any mechanistic basis for the effect of indoles and related moieties, and the lack of a convincing demonstration of this discovery in the generation of a novel antibiotic. A lot to ask, for sure, but will ultimately be needed for these observations to be widely adopted by the field.

To Rev 3's first point, understanding how the indole chemotype can promote mycomembrane permeation, and in what molecular contexts, are major future goals. We now acknowledge this more transparently:

"Although we do not yet know the mechanism, our data indicate that benzene to indole substitution can promote ML-predicted and observed mycomembrane permeation in two molecule series."

"Given that benzene to indole substitution can promote ML-predicted and observed mycomembrane permeation, albeit via an unknown mechanism, we wondered whether the modification might also enhance whole cell activity."

To Rev 3's second point, we agree that generating a novel antibiotic is indeed a challenge. Towards this end, we plan to develop models that account for parameters in addition to permeability:

"Expansion of the chemical space covered by our azide libraries, along with whole cell mass spectrometry, adaptation of PAC-MAN for the cytoplasm, and the use of strains with defined membrane, efflux, or metabolism defects will collectively enable the generation of more comprehensive accumulation models. These, in turn, may be integrated with models for target inhibition, pharmacokinetics, and synthetic feasibility to build comprehensive, biologically- and chemically-interpretable pipelines that aid antibacterial development."

That said, I do not wish to stand in the way of this study as there is lots of important info here. The impact of this work will be much higher if the authors keep their target audience (drug discoverers / microbiologists) in mind, especially in the presentation of their data.

To enhance the accessibility of the work to our target audience, we have simplified the machine learning portion of the manuscript, moving non-essential details to figure legends and methods.

A few specifics:

1) Many depictions of the data are non-intuitive and non-standard, so it is hard to understand some of the key effects and their meaning. The deltaLog10CC50, in particular, is puzzling. Is the decrease of this from ~1.75 to ~1.00 (as in Figure 4c and 4g) considered big? What are these values for high accumulating and low accumulating control compounds? This would be helpful.

The $\Delta\log_{10}CC_{50}$ parameter, which we developed in our previous work (*Lepori and Jackson, 2025, ACS Inf Dis*), is a relative measurement that can span zero (left; compound #40) to infinity (right; compound #42):

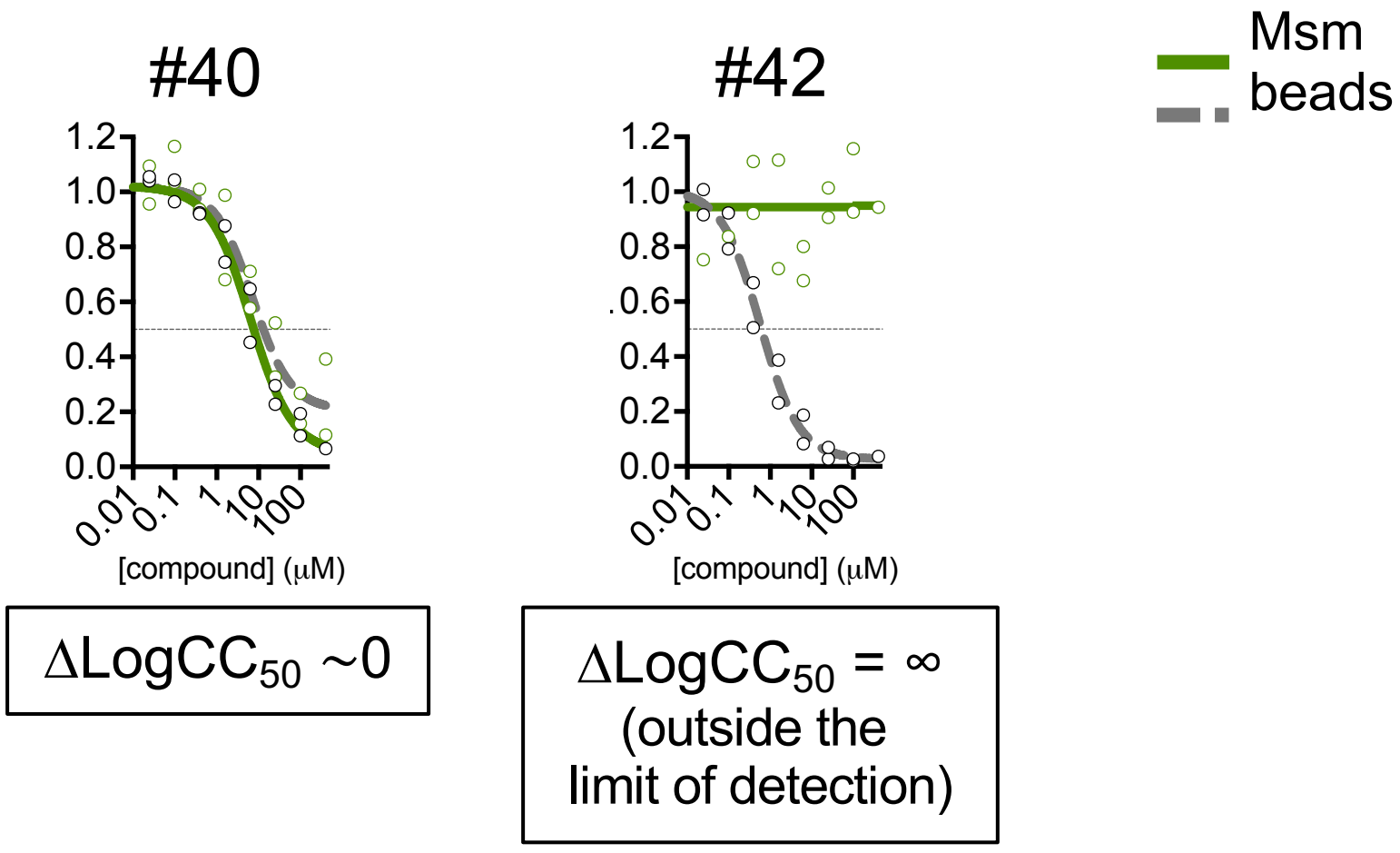

The interpretation of the parameter is complex as the limit of detection varies with a compound's reactivity (above, in dotted grey line). However, for most compounds our data suggest that even small differences in $\Delta log_{10}CC_{50}$ can be significant. In published (*Lepori and Jackson, 2025, ACS Inf Dis*) and unpublished work, we have used antibiotics to benchmark $\Delta log_{10}CC_{50}$. To clarify this important point:

"To control for azide reactivity, we exposed DBCO-labeled beads and mycobacteria to different concentrations of azide test molecules, then calculated the difference in concentration of test azide molecule that competes fluorescence by 50% in the two systems ($\Delta log_{10}CC_{50}$). We previously developed the $\Delta log_{10}CC_{50}$ parameter as a relative measurement that ranged from approximately 0.8 to 2 for more- and less-permeable azide-antibiotics, respectively."

2) Related, why are there not error bars and p-values for these experimental plots (Figure 4c and 4g), but there are error bars and p-values for the computational plots (4d and 4h)? Again, this is a puzzle to the non-computationalist.

For the experimental plots, we calculate the CC_{50} for azide compound competition on beads and on Mtb from 2-3 independent biological replicates, then take the delta.

For the computational plots, however, we do have error bars and p values. To be trained, neural network models require a starting point that is randomly generated. This "starting point" is the set of initial values of the parameters of the neural network. A model that is well suited to the data and trained appropriately will reach virtually the same result (parameter values, and thus output prediction) regardless of the value of the random starting point. We therefore analyzed the robustness of our model by accounting for the effect of different "starting points" on the model output. To make final predictions on the test compounds in the figure, we trained the ML model 50 times, inputting 50 different random "starting points," which are called random seeds. The fact that all the models trained starting from different random seeds provided a very similar result validate the robustness of the model. We then performed the Wilcoxon rank-sum test to generate p-values and define the significance of the predictions for different compounds.

We now include the above explanation in the Methods section.

3) Similar theme, Figure 6 talks about whole cell activity, but there is nothing in this figure that is familiar to the microbiologist. Why not express whole cell activity as an MIC value? A simple, easy-to-understand figure would be powerful: showing that addition of an indole (for example) takes a compound from one that is non-accumulating and inactive against Mtb, to one that now accumulates and is active against Mtb.

Unfortunately, the analyses in **Fig. 6** are retrospective, so we only have access to the screening data where compound activity was measured as % inhibition of Mtb growth at a fixed concentration of compound. The closest graph to what the reviewer is asking for is **Fig. 6a**. Here the majority (87 out of 90) of the molecules without indole are less active than the majority (9 out of 11) of the molecules with indole.

In response to this comment and one from Rev 4 below, we now include **Fig. 5l** for the **OctTriA** series to illustrate the effect of the benzene to indole substitution on predicted permeability and observed activity. We use Mtb growth at a fixed, low concentration of **OctTriA** as our readout to avoid problems with compound precipitation/aggregation.

Figure 5I

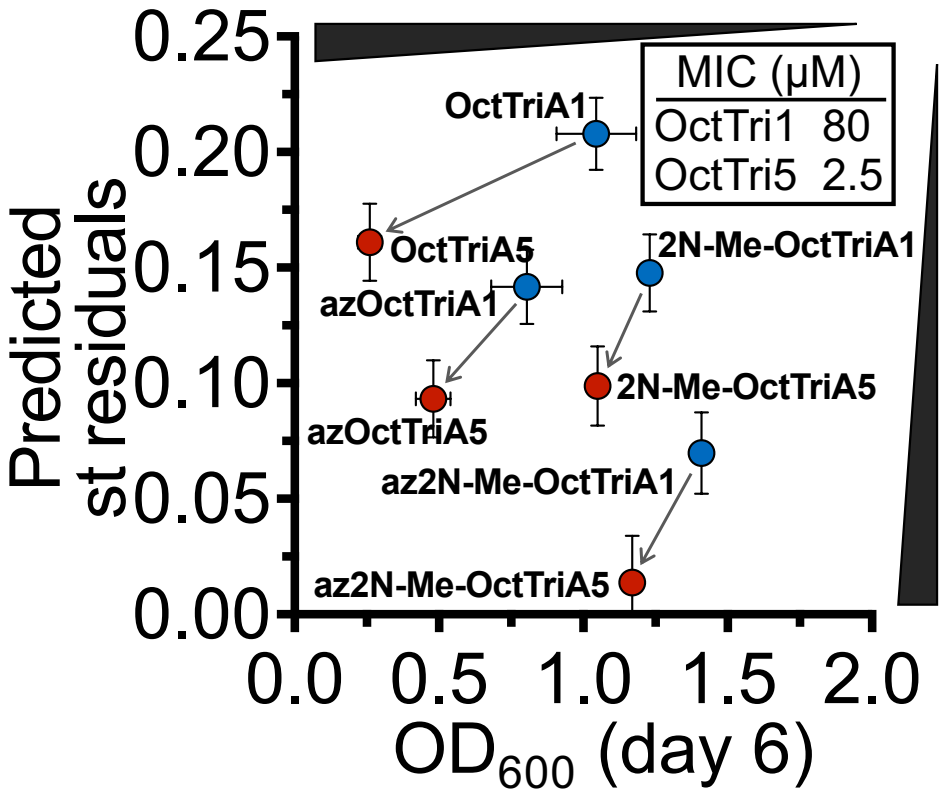

Reviewer #4

I think the focus of the study is important. Understanding the molecular features that govern intracellular accumulation is essential to make antibiotic discovery and optimization more efficient.

Given that the algorithmic methods are not new, there is not much to comment on for these parts of the paper. One minor comment here would be that with the relatively small size of their training dataset, I wonder whether a simpler binary classification method would have been more useful than a regressor; I do not expect the authors to address this at the current stage.

One of the strengths of the method we developed is the capability to provide an experimental readout that is continuous rather than binary, which is why we opted for a regression framework for our analysis. Even though in some of our experiments we made a simplification, defining a molecule as permeability-promoting or permeability-preventing (binary classification), this is applicable only when the distinction is neat, which in most cases is not true. We don't have a clear experimental threshold that definitively separates permeable from non-permeable compounds, so we decided to avoid introducing a computational threshold to artificially separate continuous data into categorical bins to retain as much information as possible on mycomembrane permeability.

Moreover, I'm unconvinced that their Chemprop model can generalize without being prospectively applied to a (somewhat) out of distribution chemical set. Again, I'm unsure whether this needs to be addressed for this study.

We agree it would be exciting to evaluate the model on newly obtained data, but as this is a new study with a new dataset, there was no out-of-distribution data available. We are currently in the process of procuring and screening a more diverse collection of ~2000 azide-tagged compounds to improve the model's generalizability. In the interim, here we have used rigorous methods to test the model's generalizability, including the "hard" scaffold splits (the field's current best approximation of an out-of-distribution evaluation), in addition to demonstrating our application to newly synthesized and tested compounds.

I do have one major comment for the authors to address. In my opinion, the most important contribution of modeling intracellular accumulation is to improve antibacterial potency, most commonly defined using MIC. However, as the authors note, ML-guided modification of JSF-2985 did not improve activity (it looks like it lost activity). Similarly, from what I can see from Figure 5, the increases in OctTri potency after modification are modest. In my opinion, this speaks to the difficulty of identifying "permeability-enabling" subgroups without considering whole cell antibacterial activity in parallel. I don't think that this is necessarily disqualifying for the study, but it is an essential observation to convey clearly to the field and a shortcoming of this study.

To address this shortcoming, in the future we plan to develop models that account for parameters in addition to permeability:

"Expansion of the chemical space covered by our azide libraries, along with whole cell mass spectrometry, adaptation of PAC-MAN for the cytoplasm, and the use of strains with defined membrane, efflux, or

metabolism defects will collectively enable the generation of more comprehensive accumulation models. These, in turn, may be integrated with models for target inhibition, pharmacokinetics, and synthetic feasibility to build comprehensive, biologically- and chemically-interpretable pipelines that aid antibacterial development."

We also address the shortcoming explicitly:

*"However, we found that Mtb growth inhibition by the **JSF-2985** derivatives did not reflect their mycomembrane permeation profiles (**Fig. 5e, Extended Data Fig. 5b-c**). We and others have found that the cell envelope, including the mycomembrane, is a differential barrier that restricts permeation and whole cell activity for some antibacterials, e.g., vancomycin and moxifloxacin, more than others, e.g., linezolid and ciprofloxacin. The lack of correlation between mycomembrane permeation and growth inhibition for **JSF-2985** suggested that the mycomembrane might not be an important barrier to its whole cell activity. Indeed, we found that a sub-inhibitory dose of ethambutol, an antibiotic that indirectly disrupts the mycomembrane does not potentiate **JSF-2985** activity against Mtb (**Fig. 5f**)."*

Fig. 5e is a new plot requested by the reviewer (see below). As well:

*"…even for molecules for which the mycomembrane poses a major barrier to uptake, improving permeation via derivatization might compromise other aspects of whole cell activity, e.g., target engagement or susceptibility to metabolism and efflux. For example, N-methylation enhances observed and predicted mycomembrane permeation of the octyl-tridecaptins in Msm and Mtb but activity only against Msm (ref, **Fig. 5, Extended Data Fig. 7**). By contrast, benzene to indole substitution can enhance permeation and activity of these derivatives in both species (**Fig. 5, Extended Data Fig. 7**)."*

I think that the authors need to add a set of growth inhibition curves (OD as a function of concentration) to the main text figures, showing (a) the starting "parent" molecule and (b) the ML-guided modified analogs. This is essential to depict clearly, to highlight the disconnect between predicted (or experimentally validated) envelope permeability and whole cell antibacterial potency, which is what's being chased this this type of work.

We were able to perform the requested experiment for **OctTriA1** and **OctTriA5** (**Extended Data Fig. 7d**) and, towards a similar end, measure MICs (**Fig. 5l**, top right inset). In these assays, as in our original growth curves (**Fig. 5k**), **OctTriA5** (two indoles) has better activity than **OctTriA1** (one indole).

For some of the **JSF-2985** and **OctTriA** compounds we were limited by precipitation/aggregation and/or compound availability. Thus, we compared activity within the two series by growing Mtb +/- lower, fixed concentrations. To address the reviewer's larger concern—inconsistent relationships between mycomembrane permeability and activity in different compound series—we have now included graphs of predicted mycomembrane permeability vs. OD_{600} at 30 μM (**Fig. 5e**) or 5 μM (**Fig. 5l**) in the main text:

Extended Data Figure 7d

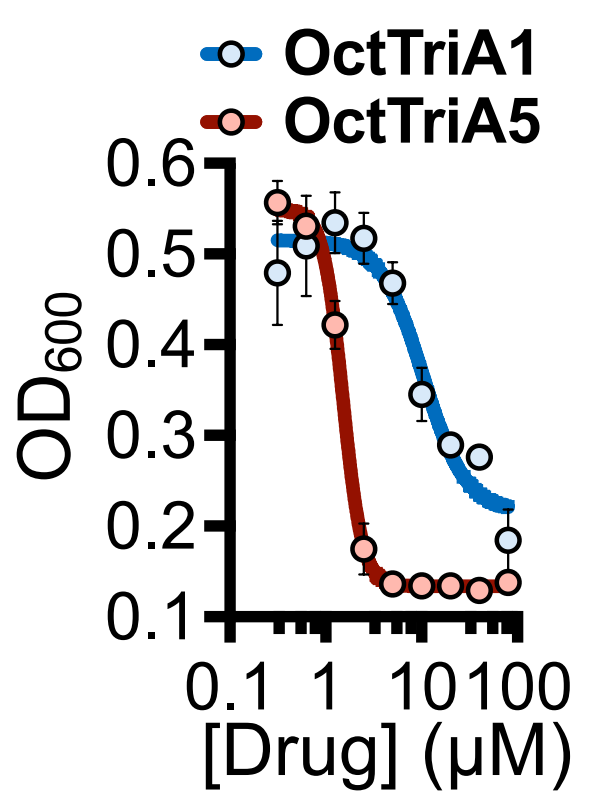

Figure 5e

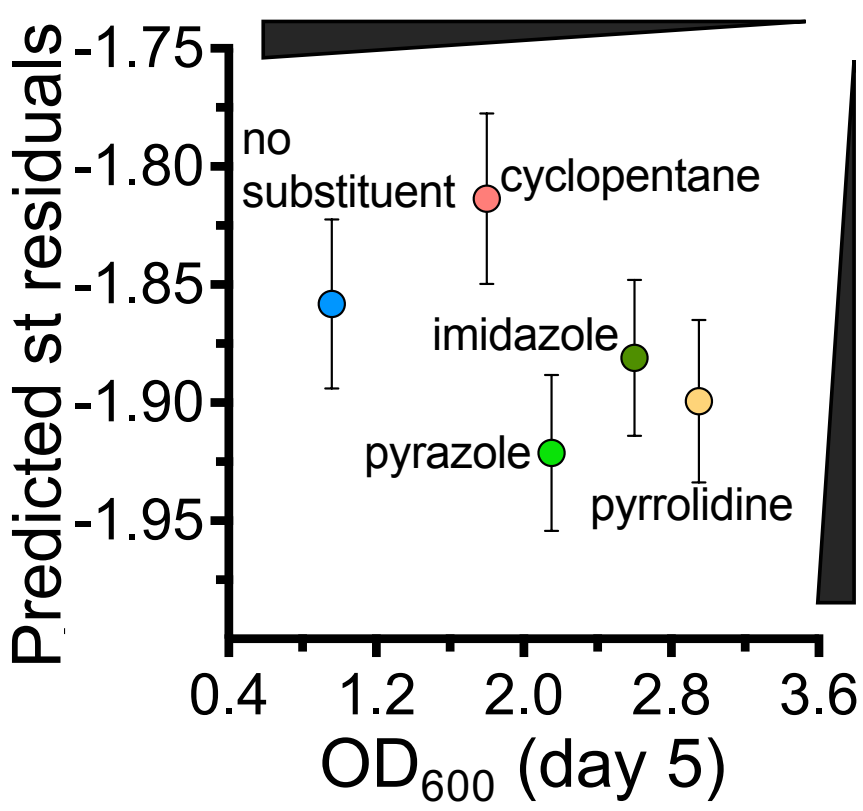

Figure 5l

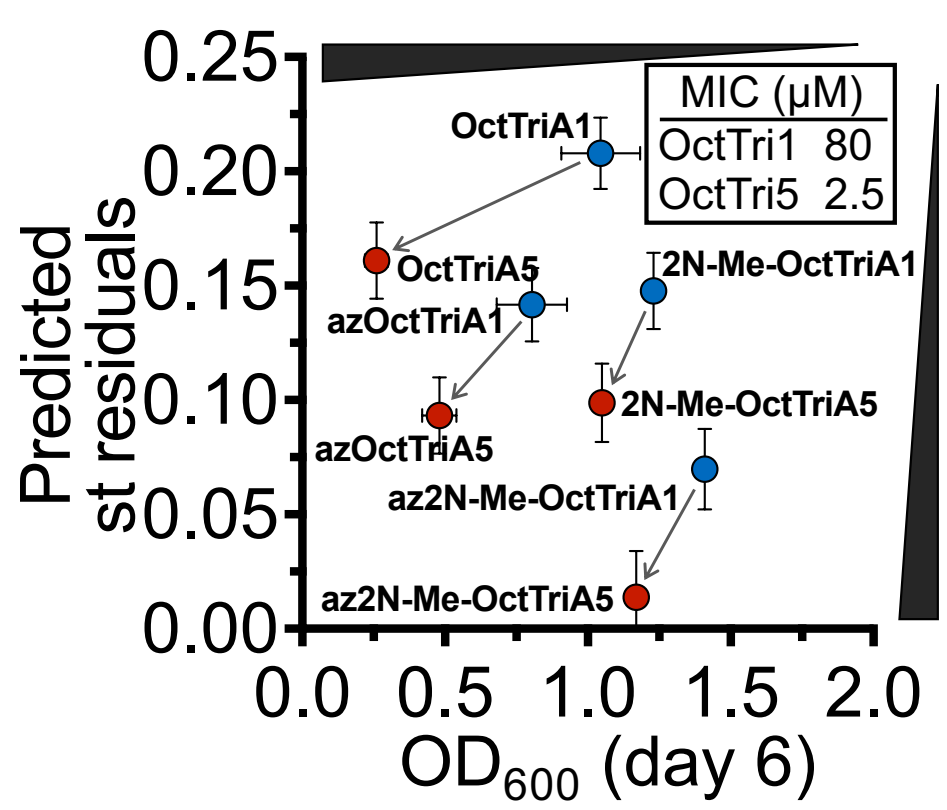

Reviewer #3 (Remarks to the Author):

This resubmission provides some clarity and is suitable for publication. There is lots of work here and it is a very important problem. That said, most of the methods are non-standard (for accumulation, inhibition of growth, etc) so readers will largely be unable to meaningfully scrutinize and understand these new methods, all the more reason that it will be important for the authors to demonstrate -- in future papers -- the actionability of their discoveries to the creation of novel antibiotics against TB.

Reviewer #4 (Remarks to the Author):

I appreciate the additional experiments and text revisions made during this round of revisions. I have a few comments that should be addressed. Notably, I do not want to impede the publication of this study because it's a useful contribution to the field, but there are some claims that are beyond what I think the data directly supports.

1. In their responses, the authors state "The lack of correlation between mycomembrane permeation and growth inhibition for JSF-2985 suggested that the mycomembrane might not be an important barrier to its whole cell activity." However, in the manuscript text, they also state "Here, we found that JSF-2985 derivatives with imidazole, pyrazole, and pyrrolidine scaffolds permeate the mycomembrane better than the JSF-2985 derivative bearing cyclopentane, consistent with both cheminformatics analyses and MycoPermeNet predictions." My interpretation to these statements is that it is possible to improve JSF-2985 permeability, but doing so also can decrease its inherent antibacterial activity. I think this needs to be stated far more clearly. Simply increasing permeability is not a guaranteed method to improve potency.

The reviewer's interpretation is correct. We have observed that certain drugs don't increase their whole cell activity even when permeability increases, for example, when the mycomembrane is disrupted. Because the relationship between drug activity and permeability is complicated, we analysed these properties across large datasets (total of ~300k compounds, Figure 6).While we observed the predicted scaffold-specific relationships, we certainly agree that the effect of permeability on drug activity is context- and molecule-dependent.

We acknowledge this complexity throughout the paper:

*"Activity is an indirect proxy for uptake[17]; collateral metabolic dysfunction from cell envelope perturbation[18-21] may also impact drug sensitivity. To clarify, we recently tested activity in parallel with Peptidoglycan Accessibility Click-Mediated AssessmeNt (PAC-MAN[7, 22]; **Fig. 1a**)–an assay that measures the arrival of azide-modified molecules to the periplasm independent of their activity–to show that the mycomembrane is a selective permeability barrier and differentially restricts access of drugs."*

"Our work demonstrates that the mycomembrane is a major, albeit selective barrier to whole cell activity in Mtb"

"We and others have found that the cell envelope, including the mycomembrane, is a differential barrier that restricts permeation and whole cell activity for some antibacterials, e.g., vancomycin and moxifloxacin, more than others, e.g., linezolid and ciprofloxacin[7, 15, 43]."

*"Thus, this scaffold swap simultaneously promotes mycomembrane permeation and, in the context of the octyl tridecaptins, enhances whole cell anti-Mtb activity (**Fig. 5k-l**)."*

*"On the small scale, MycoPermeNet and our interpretability studies on individual molecules (**Fig. 3f**) may help guide derivatization of lead compounds, although their utility will depend on several factors. First, the mycomembrane is not always a significant barrier for anti-Mtb antimicrobials[7, 15,*

[43] (**Fig. 5f**). Second, even for molecules for which the mycomembrane poses a major barrier to uptake, improving permeation via derivatization might compromise other aspects of whole cell activity, e.g., target engagement or susceptibility to metabolism and efflux."*

2. This extends to the comment from Reviewer #3 "…and the lack of a convincing demonstration of this discovery in the generation of a novel antibiotic". Currently we do not have a detailed understanding of how generalizable this indole-dependent phenotype is across chemical space. I think to address these concerns, some of the language should be toned down in a manner that highlights the limitations of this set of observations.

In addition to the statements above, we have modified the manuscript title and sub-headings to tone down our claims about activity. For example:

Title:
from:
Chemical features that influence mycomembrane permeation and antitubercular activity
to:
Identification of chemical features for improved accumulation past the mycobacterial outer membrane

Sub-headings:
from:
Benzene to indole substitution can promote whole cell activity of an antimicrobial peptide
to:
Indole association with whole cell activity

Removed:
Indole predicts mycomembrane permeability and whole cell activity

from:
Scaffolds and other chemical predictors of mycomembrane permeability also predict whole cell activity in large datasets
to:
Permeability predictors correlate with whole cell activity

3. If I am interpreting Figure 5e and Figure 5l correctly, their model predicts that OctTriA lives in a chemical space that is inherently less permeable than JSF-2985. Given that OctTriA permeability is correlated with antibacterial activity, this may indicate that the indole substitution approach is better suited for molecules that are inherently less permeable, as opposed to increasing the permeability of molecules that are already reasonably able to permeate the mycomembrane.

We agree! In the manuscript we state:

*We hypothesized that the larger size of **OctTriA1** (1521 Da) might render the molecule more sensitive to membrane barriers than **JSF-2985** as well as enable structure activity relationship (SAR)-informed chemical modifications that do not directly interfere with on-target activity[39, 41]. In support of the first conjecture, we found that sub-MIC ethambutol sensitizes Mtb to **OctTriA1**, unlike **JSF-2985** (**Fig. 5f**).*

4. In Figure 3f, the authors use SHAP values as a pseudo-interpretability method. However, these insights are not really leveraged in the rest of the paper. What is the overall purpose of this analysis if it's not being used to guide molecular optimization in downstream experiments?

Additional explanation around this point would help readers understand how SHAP relates to their other modelling efforts.

There were indeed many paths not taken post-SHAP analysis.. We opted to focus mostly on chemical scaffolds because of their unexpected and striking effects in both our cheminformatic and ML analyses.

We now highlight explicitly the potential of the mentioned interpretability analysis in the discussion:

*"On the small scale, MycoPermeNet and our interpretability studies on individual molecules (**Fig. 3f**) may help guide derivatization of lead compounds [...]"*

We are now in the process of creating a web interface that will be broadly accessible. While beyond the scope of the current study, we hope that this tool will enable the community to use our interpretability model to understand and improve the mycomembrane permeability for molecules of interest.
